# Bimetallic Sites for Catalysis: From Binuclear Metal Sites to Bimetallic Nanoclusters and Nanoparticles

**DOI:** 10.1021/acs.chemrev.2c00733

**Published:** 2023-03-27

**Authors:** Lichen Liu, Avelino Corma

**Affiliations:** † Department of Chemistry, Tsinghua University, Beijing 100084, China; ‡ Instituto de Tecnología Química, Universitat Politècnica de València−Consejo Superior de Investigaciones Científicas (UPV-CSIC), Avenida de los Naranjos s/n, Valencia 46022, Spain

## Abstract

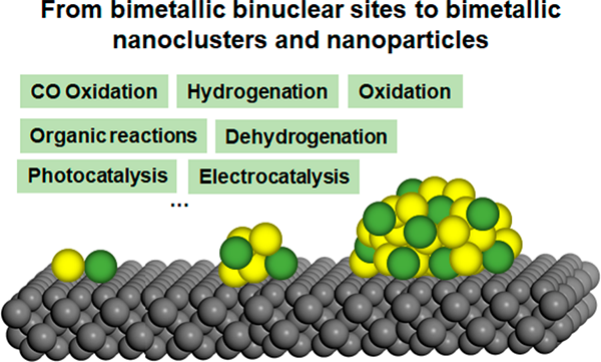

Heterogeneous bimetallic catalysts have broad applications in industrial processes, but achieving a fundamental understanding on the nature of the active sites in bimetallic catalysts at the atomic and molecular level is very challenging due to the structural complexity of the bimetallic catalysts. Comparing the structural features and the catalytic performances of different bimetallic entities will favor the formation of a unified understanding of the structure–reactivity relationships in heterogeneous bimetallic catalysts and thereby facilitate the upgrading of the current bimetallic catalysts. In this review, we will discuss the geometric and electronic structures of three representative types of bimetallic catalysts (bimetallic binuclear sites, bimetallic nanoclusters, and nanoparticles) and then summarize the synthesis methodologies and characterization techniques for different bimetallic entities, with emphasis on the recent progress made in the past decade. The catalytic applications of supported bimetallic binuclear sites, bimetallic nanoclusters, and nanoparticles for a series of important reactions are discussed. Finally, we will discuss the future research directions of catalysis based on supported bimetallic catalysts and, more generally, the prospective developments of heterogeneous catalysis in both fundamental research and practical applications.

## 1 Introduction

The application of bimetallic particles is widely practiced in industrial catalysis.^1,2^ The solid catalysts comprising two metals, either in the form of alloys or particulate mixture, can be more efficient than the corresponding monometallic catalysts.^3^ For instance, the industrial naphtha reforming catalysts based on Pt-based bimetallic particles (e.g., PtIr and PtRe) supported on Al_2_O_3_ have played a crucial role in the conversion of alkanes into aromatics. The introduction of the second metal (i.e., Ir and Re) to Pt can remarkably improve the selectivity of aromatics and prolong the catalyst’s lifetime.^4,5^ In another example, bimetallic AuPd catalysts are used for the production of vinyl acetate monomer by the reaction between acetic acid and ethylene in the presence of oxygen.^6^ Pd is the main functional component, while adding Au can improve Pd particles’ stability by protecting them from sintering. In another example, the Lindlar catalyst, which is made by PdPb alloys, is used for the selective hydrogenation of alkynes to alkenes.^7^ The role of Pb is generally proposed for poisoning the Pd sites to avoid the overhydrogenation of alkynes into alkanes. Moreover, the industrial catalysts used in hydrotreating processes rely on the bimetallic PdPt particles because of their superior sulfur-resistant capability over the monometallic counterparts.^8^ These widely employed bimetallic catalysts in the chemical industry demonstrate the importance of the construction of active sites through the combination of different metals.

Although tremendous efforts have been devoted to mechanistic studies on bimetallic nanoparticles, the reasons the bimetallic particles can deliver better or different performances than the corresponding monometallic particles are still unclear. In some reactions, the bimetallic catalysts show enhanced catalytic performances (higher activity and/or selectivity), while in some others, the bimetallic catalysts are less active or show lower selectivities toward desired products. For instance, AuPd alloy nanoparticles show significantly higher activity for oxidation of primary alcohols to aldehydes and oxidation of toluene to benzyl benzoate.^9,10^ Moreover, AuPd bimetallic nanoparticles also show dramatically enhanced activity for hydrogenation of levulinic acid to γ-valerolactone.^11^ The addition of Pt-group metals to Au for promoting the activity for hydrogenation reactions has also been found with AuPt catalysts for chemoselective hydrogenation of nitroaromatics.^12^ However, in some cases like CO oxidation, water–gas shift, and decomposition of formic acid to CO_2_ and H_2_, AuPd bimetallic nanoparticles are less active than pure Au nanoparticles.^13^ It is well-known that the electronic structures and the geometric structures of bimetallic nanoparticles are different from the corresponding monometallic nanoparticles. However, the correlation between reactivity and structural factors is still unknown, or at least, not fully elaborated. The two metals may both participate in a catalytic reaction by accounting for different elementary steps. In another scenario, only one metal works as the functional component while the other metal is added to modify the structure of the functional component.

**Figure 1:**
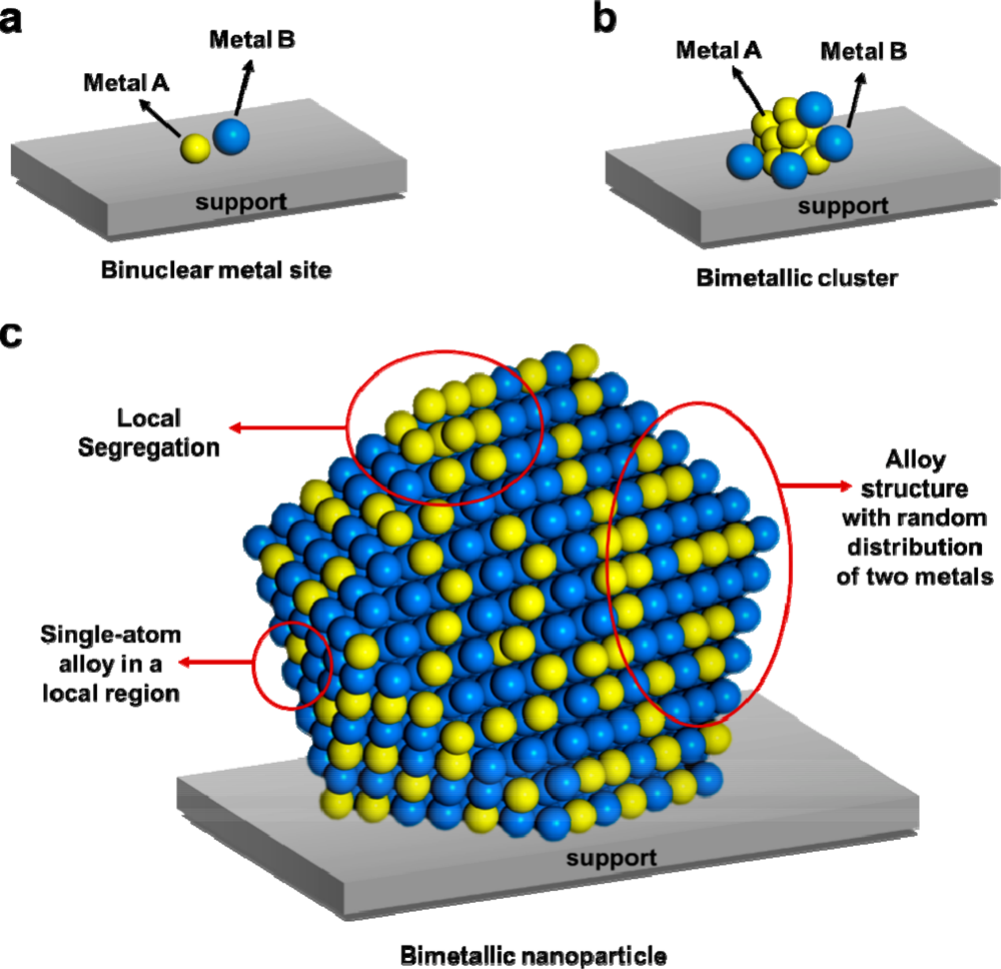
Schematic illustration of three types of supported bimetallic sites. (a) The supported binuclear metal site, (b) supported bimetallic nanocluster, and (c) supported bimetallic nanoparticle. It should be noted that currently there is no definite size boundary between “nanocluster” and “nanoparticle”. In our opinion, metal clusters whose particle sizes are ≤1 nm (subnanometer clusters) could show distinct catalytic properties in comparison to metal nanoparticles with sizes >1 nm because subnanometer clusters show molecule-like electronic structures and nearly all the metal atoms in the clusters are exposed to reactants. In this review, we mainly refer to “nanoclusters” as metal species with sizes ≤1 nm.

The difficulty to clarify the so-called “synergistic effects” in supported bimetallic catalysts could be caused by the complexity of the structures of bimetallic nanoparticles.^14^ Depending on the atomic arrangements of the two metal elements, a bimetallic catalyst could be in the form of a random alloy, intermetallic compound, segregated structure, or mixture. Even in a single bimetallic nanoparticle (as depicted in Figure 1c), multiple types of metal sites can be formed, depending on the spatial distribution of the two elements and their catalytic functions could be quite different. Therefore, it is very difficult to rationalize the structures of the active sites within the practical bimetallic nanoparticulate catalysts.

In recent years, the emerging works on catalysts comprising isolated metal atoms or subnanometer metal clusters as active species have brought new insights into the size-dependent catalytic properties of supported metal catalysts, which have already been extensively studied for decades based on supported metal nanoparticles with sizes above 1 nm. By comparing the catalytic performance of isolated metal atoms, subnanometer metal clusters, and nanoparticles and correlating their structural features (geometric and electronic structures) with catalytic behaviors, a deeper understanding of the size effect in supported metal catalysts has been achieved.^15^ In this direction, we have witnessed the explosion of works, in which people are striving to control the size and coordination environment of supported subnanometer metal catalysts and to understand the structures of the active sites at the molecular and atomic levels.

By translating this concept from monometallic systems to bimetallic systems, it can be anticipated that the size of the bimetallic sites will also have a significant impact on their catalytic behaviors. For instance, it has already been found that subnanometer PtSn clusters can show different geometric and electronic structures compared to the bimetallic PtSn nanoparticles.^16^ Such structural differences are also reflected in the structural evolution behavior at high-temperature redox treatments^17^ and the catalytic performance for propane dehydrogenation.^16^

From a structural point of view, compared to the conventional bimetallic nanoparticles (>1 nm), the bimetallic sites are made by binuclear metal sites (two neighboring metal atoms on the solid carrier, as depicted in Figure 1a) or bimetallic nanoclusters (≤1 nm, as depicted in Figure 1b) have relatively less complicated structures. Indeed, with the advanced characterization techniques, it is possible to fully resolve the structure of bimetallic nanoclusters.^18^ In this sense, the bimetallic sites, either in the form of binuclear atoms or small clusters, can be model systems to understand the elusive “synergistic effects” in the large bimetallic nanoparticles.

In this review, we aim to make a comprehensive overview of the recent progress on supported bimetallic catalysts, covering the range of binuclear metal sites to bimetallic nanoclusters and nanoparticles. Considering the abundant publications and reviews on bimetallic nanoparticles, we will focus on the bimetallic sites in the subnanometer regime in terms of materials synthesis and characterizations. Moreover, we will make critical comparisons of the three types of bimetallic entities in terms of their structural features and catalytic behaviors. By summarizing the results obtained in different systems, we may gain a more unified and global understanding of the working mechanism and potential of bimetallic systems in heterogeneous catalysis.

## 2 Structural Features of Different Types of Bimetallic Entities

The interaction between the bimetallic entities and substrate molecules will be greatly influenced by the geometric and electronic structures of the bimetallic entities. In this section, we will discuss and make a critical comparison in terms of the structural features of different types of bimetallic entities.

### 2.1 Binuclear Bimetallic Sites

#### 2.1.1 Geometric Structures of Binuclear Bimetallic Sites

In binuclear complexes, the two metal atoms are coordinated by ligands and the full structures can be fully resolved by proven techniques developed in molecular science. However, when the binuclear metal species are immobilized on a surface, the binding sites on the surface can partially or fully serve as “solid ligands” to coordinate with the two metal atoms. In this sense, the geometric structure of the binuclear bimetallic sites can be determined by elucidating the local structures of the binding sites on the surface and the ligand/atom directly bonding with the metal atoms. To improve the stability of the binuclear species, the bonding interaction between the metal species and the support needs to be enhanced by appropriate thermal treatment, resulting in binuclear species free of organic ligands. Taking the carbon-based support as an example, the two metal atoms are shown to be preferentially located at the vacant sites with heteroatoms (N, S, O, or P) at the binding sites, as revealed by experimental measurements and theoretical modeling.^19,20^

**Figure 2:**
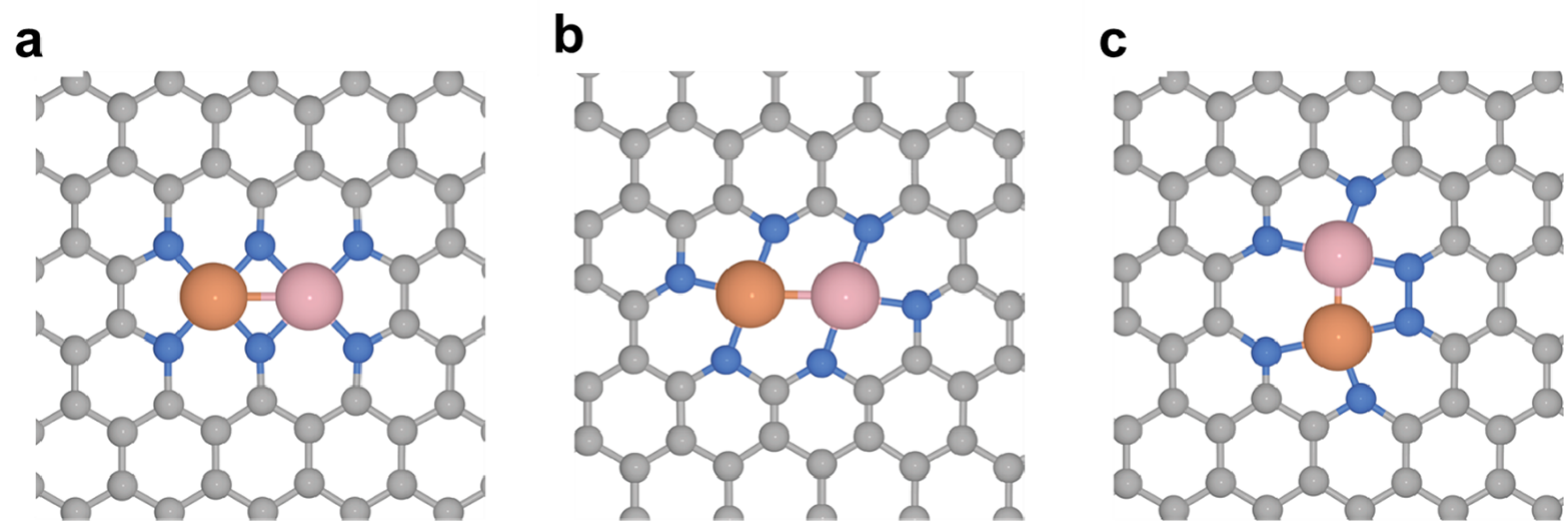
Several possible structural configurations of binuclear bimetallic sites are embedded in the carbon matrix. In the three cases, the two metal atoms are stabilized by the heteroatoms doped in the carbon matrix and the coordination environment of the binuclear bimetallic sites depends on the configuration of the bonding between the metal atoms and the heteroatoms. In the practical materials, the coordination configuration of the binuclear sites will be more complicated and disordered than the depicted models and multiple types of configurations may exist in an individual sample.

It is anticipated that the distance between the two metal atoms is dependent on the structure of the vacant site for accommodating the binuclear bimetallic species. According to the models presented in the reported works, the most stable configurations of binuclear bimetallic sites are formed in the vacant sites in the carbon matrix by bonding with the carbon atoms or heteroatoms (N, O, S, etc.) in the carbon matrix.^21^ As shown in Figure 2, several typical configurations of binuclear bimetallic sites embedded in carbon matrix show distinct local structural features, resulting in different coordination environments. One critical issue related to the structure of binuclear bimetallic sites is the interaction between the two metal atoms. In binuclear bimetallic complexes, the two metal atoms can have direct metal–metal bonding or be bridged through ligands.^22^ In the cases of supported binuclear bimetallic sites, the formation of metal–metal bonding is rarely observed. Instead, the two metal atoms are usually separated by the atom (such as O, C, N, S, P, etc.) in the support, exhibiting a longer metal–metal distance than that in conventional metallic nanoparticles.

#### 2.1.2 Electronic Structures of Binuclear Bimetallic Sites

In principle, the electronic structures can be fully elaborated if the coordination environment of the binuclear sites can be fully resolved, although as mentioned before, it is a very challenging task for a practical solid catalyst. The coordination environment of the metal atoms on the support is not as well-defined as the ligand in the molecular complex. According to the reported examples in the literature, the metal atoms in the binuclear sites are usually positively charged with interaction through the metal–heteroatom (O, C, N, S, P, etc.) interaction. The electronic structure of the metal atoms depends on their bonding with the support and the distance between the two metal atoms. For instance, in the Pt–Co binuclear sites, both metals show a chemical state of +2, and coupling between the d orbitals of both atoms may occur, according to DFT calculations.^24^

**Figure 3:**
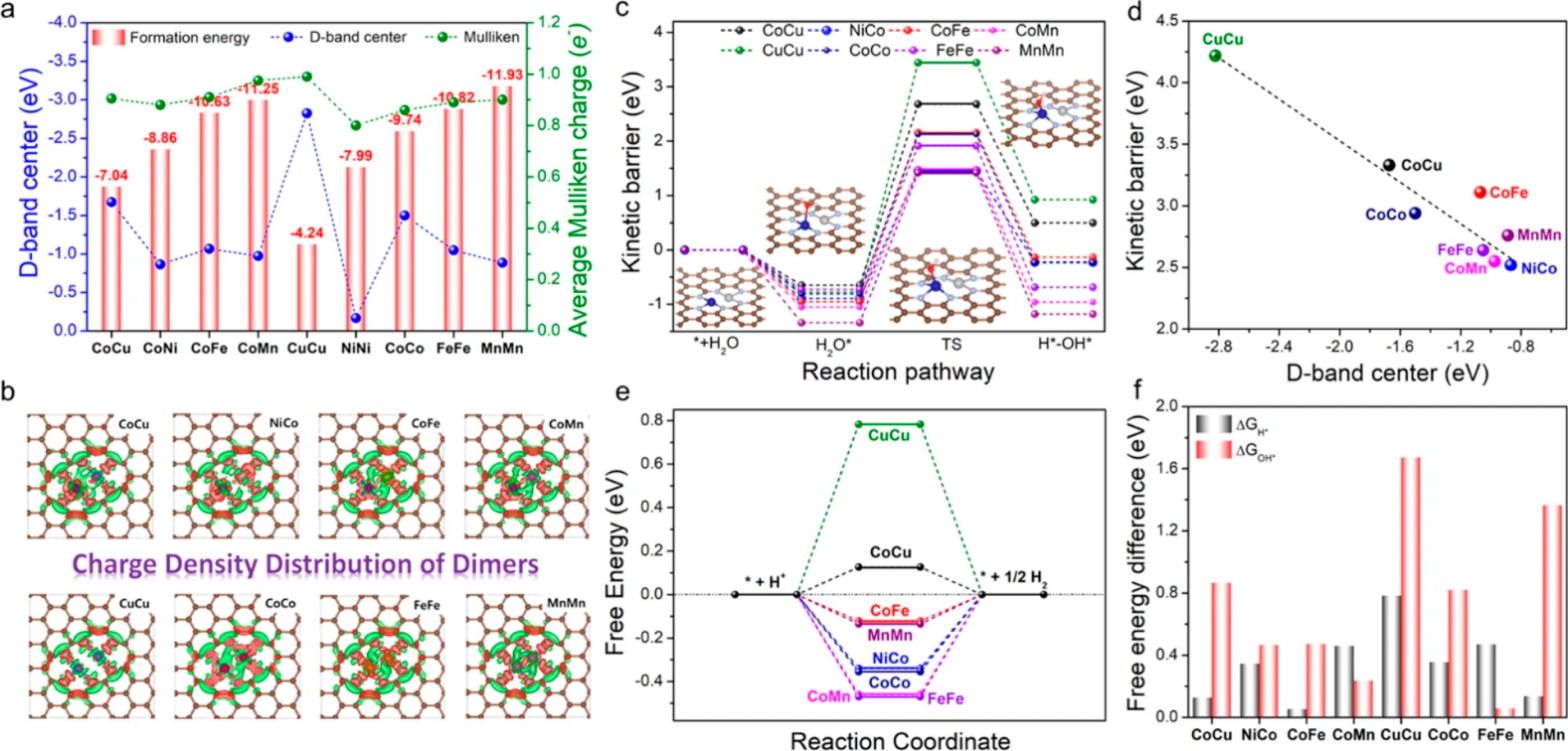
High-throughput screening of various binuclear transition metal entities for electrocatalytic hydrogen evolution reaction. (a) Formation energies corresponding to the charge depletion (Mulliken charge) and d-band center of various binuclear metal entities embedded in the N-doped carbon matrix. (b) Charge density distributions of binuclear metal species embedded in N-doped carbon matrix. The charge depletion and accumulation are marked by green and red colors, respectively. (c) Reaction pathways with the minimum energy barrier of water splitting reactions on binuclear metal entities embedded in N-doped carbon matrix. (d) Linear correlation between the kinetic energy barrier and d-band center. (e) Free energy diagram of hydrogen adsorption and (f) free energy changes of the hydronium (Δ*G*_H*_) and hydroxide (Δ*G*_OH*_) desorption step for binuclear metal entities embedded in N-doped carbon matrix. Reproduced with permission from ref (23). Copyright 2022 Springer Nature under CC-BY license (https://creativecommons.org/licenses/by/4.0/).

Active sites based on binuclear bimetallic species offer the opportunity to establish the structure–reactivity relationship in a relatively straightforward approach. For instance, it has been revealed by density functional theory (DFT) that some fundamental parameters such as electron affinity, electronegativity, and the radii of the metal atoms embedded in the carbon matrix can be used to predict the catalytic performance for the oxygen reduction reaction.^25^ In another example, the charge depletion (Mulliken charge) and d-band center are employed as a descriptor for screening catalysts of binuclear bimetallic sites stabilized in N-doped carbon matrix for electrocatalytic H_2_ evolution reaction.^23^ As presented in Figure 3, by varying the metal element in the binuclear sites, the NiCo bimetallic site can upshift the d-band center and thereby facilitate rapid water dissociation (lower kinetic barrier) and proton adsorption, resulting in improved electrocatalytic activity for water splitting to H_2_.

### 2.2 Bimetallic Nanoclusters

#### 2.2.1 Geometric Structures of Bimetallic Nanoclusters

Numerous works have studied the structural features of bimetallic nanoclusters via theoretical and/or experimental approaches. For instance, according to density functional theory (DFT), Au atoms in bimetallic Au_*m*_Ag_*n*_ clusters (2 ≤ *m*+*n* ≤ 8) tend to occupy the surface sites because of the charge transfer from Ag to Au, which is consistent with the experimental observation with bimetallic Au*_m_*Ag*_n_* (*m*+*n* = 34) nanoclusters.^26,27^ The local segregation has also been observed with other bimetallic nanoclusters, and the situation is greatly related to the composition of the bimetallic clusters, even the reported works only consider the geometric structures of “naked” bimetallic nanoclusters.^28^

It can be expected that the real situations will be more complicated after the introduction of ligands or solid carriers. The presence of organic ligands, especially those with strong interaction with the bimetallic nanoclusters, will block part or all of the surface sites, resulting in stable nanoclusters with low reactivity. On the other hand, by proper activation means, the ligands bonded to the bimetallic nanoclusters could be removed or replaced by some weakly bonded ligands, leaving open sites for substrate molecules.^29^

In the case of bimetallic nanoclusters supported on a solid carrier, the three-dimensional configuration of the bimetallic nanoclusters will determine the exposed metal sites. For relatively large nanoclusters (with more than 20 atoms), only quite a few atoms will not be accessed by the reactants unless the nanoclusters show a two-dimensional configuration on the support. Indeed, two-dimensional “raft-like” structures have been observed with bimetallic PtPd nanoclusters on CeO_2_.^30^ By varying the composition of the support, the geometric structures of the bimetallic nanoclusters will also change accordingly. In some cases, segregation may occur due to the different metal–support affinity of the two metal elements in the bimetallic nanoclusters.

#### 2.2.2 Electronic Structures of Bimetallic Nanoclusters

**Figure 4:**
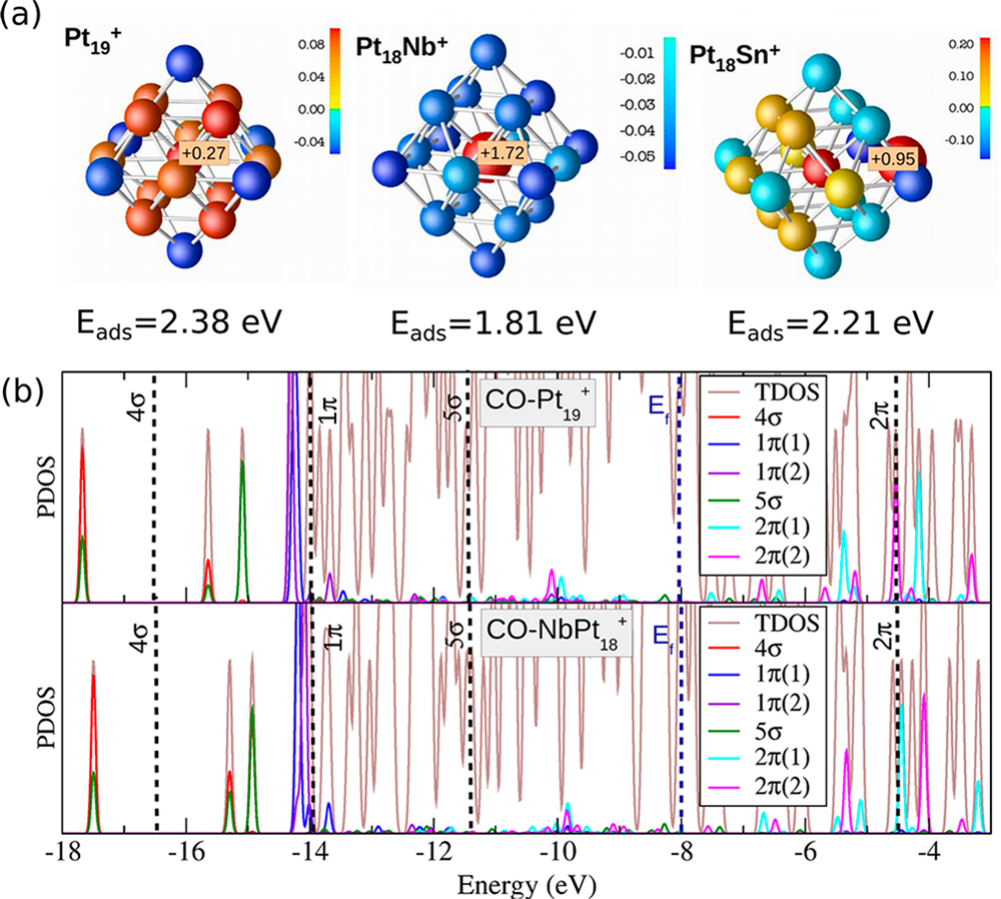
Electronic structures of bimetallic nanoclusters. (a) Bader charge distributions on MPt_18_^+^ clusters (M = Pt, Nb, Sn). Positively and negatively charged atoms are colored in red and blue, respectively. The labeled charge indicates the value (in e) of the corresponding dopant atom. (b) Densities of states of Pt_19_^+^–CO and NbPt_18_^+^–CO projected on the CO molecular orbitals. The brown line shows the total density of states. Dashed vertical lines indicate the position of free CO molecular orbitals. Reproduced with permission from ref (31). Copyright 2016 Wiley-VCH.

The electronic structures of bimetallic nanoclusters also depend on many factors including the particle size, composition of the clusters, and metal–support interaction.^32^ For instance, Ag*_n_*Ni*_n_* (*n* ≤ 7) clusters show size-dependent electronic structures according to the DFT calculations. Small Ag_*n*_Ni_*n*_ (*n* ≤ 4) clusters show Ag-like properties, while large Ag_*n*_Ni_*n*_ (5 ≤ *n* ≤ 7) show Ni-like properties.^33^ The impacts of tunable electronic structures are also reflected in the interaction between the bimetallic nanoclusters and substrate molecules. By replacing one Pt atom in the Pt_8_ cluster with one Ru atom, the activation energy for O_2_ will be decreased.^34^ Electron donation from Ru atom to neighboring Pt atoms occurs because of the lower electronegativity of Ru. As a result, charge polarization in the Ru_1_Pt_7_ cluster leads to a shift of the d-band center close to the Fermi level, resulting in the enhanced capability for O_2_ and CO activation than the Pt_8_ cluster. As shown in Figure 4, the profound influences of dopants in the electronic structures of bimetallic nanoclusters have also been demonstrated with the Pt_19_^+^ cluster. The doping of a single Nb atom leads to electron transfer from Nb to Pt, giving to negatively charged Pt atoms. Consequently, CO adsorption on the Nb-doped Pt cluster is weakened due to the lower capability for accepting the electrons from CO.^31^ In contrast, the electron transfer between Sn and Pt is much weaker, resulting in a minor modification of the electronic properties of the Pt_19_^+^ cluster.

The variation in metal–molecule interaction caused by the formation of bimetallic nanoclusters could be the consequence of changes in both electronic and geometric structures. For instance, the adsorption energy of CO on Au clusters is increased after the introduction of Rh to the Au clusters, which is ascribed to the electron transfer between Au and Rh and the occupation of less coordinated sites by Au atoms.^35^ The geometric and electronic effects are usually interconnected, as found in other systems.^36,37^

The metal–support interaction also has a profound influence on the electronic structures of bimetallic nanoclusters because of the charge transfer between the nanoclusters and the support. For example, after deposition of Pt atoms on SnOx/TiO_2_ surface, in which the SnOx species exist as single-layer structures, SnOx is partially transformed into metallic Sn, giving to the formation of Pt–Sn bonding.^38^ The electron transfer from Sn species to the TiO_2_ support causes the dipole–dipole repulsion of Sn atoms and the sintering of the PtSn clusters is therefore suppressed. When the support is changed from inorganic to organic carrier (e.g., polymers, metal–organic frameworks), the electron-transfer from the functional groups to the bimetallic nanoclusters will be largely different, although the electronic features of the polymer-encapsulated and MOF-encapsulated bimetallic nanoclusters have not been systematically compared with those supported on conventional inorganic carriers.^39,40^

### 2.3 Bimetallic Nanoparticles

#### 2.3.1 Geometric Structures of Bimetallic Nanoparticles

The degree of complexity for the geometric structures of bimetallic nanoparticles further increases in comparison with bimetallic nanoclusters. The bimetallic nanoclusters usually show disordered and flexible geometric structures because of the lack of enough metal–metal bonding. In the case of bimetallic nanoparticles, ordered geometric structures with crystalline features are formed, which leads to the higher stability of bimetallic nanoparticles than the nanoclusters. Of course, there are also some reports on bimetallic nanoparticles with an amorphous structure, in which the spatial arrangements of the metal atoms are not as ordered as the crystalline structure. It can be expected that the structural stability of the amorphous bimetallic nanoparticles will lie between the crystalline nanoparticles and the nanoclusters.

**Figure 5:**
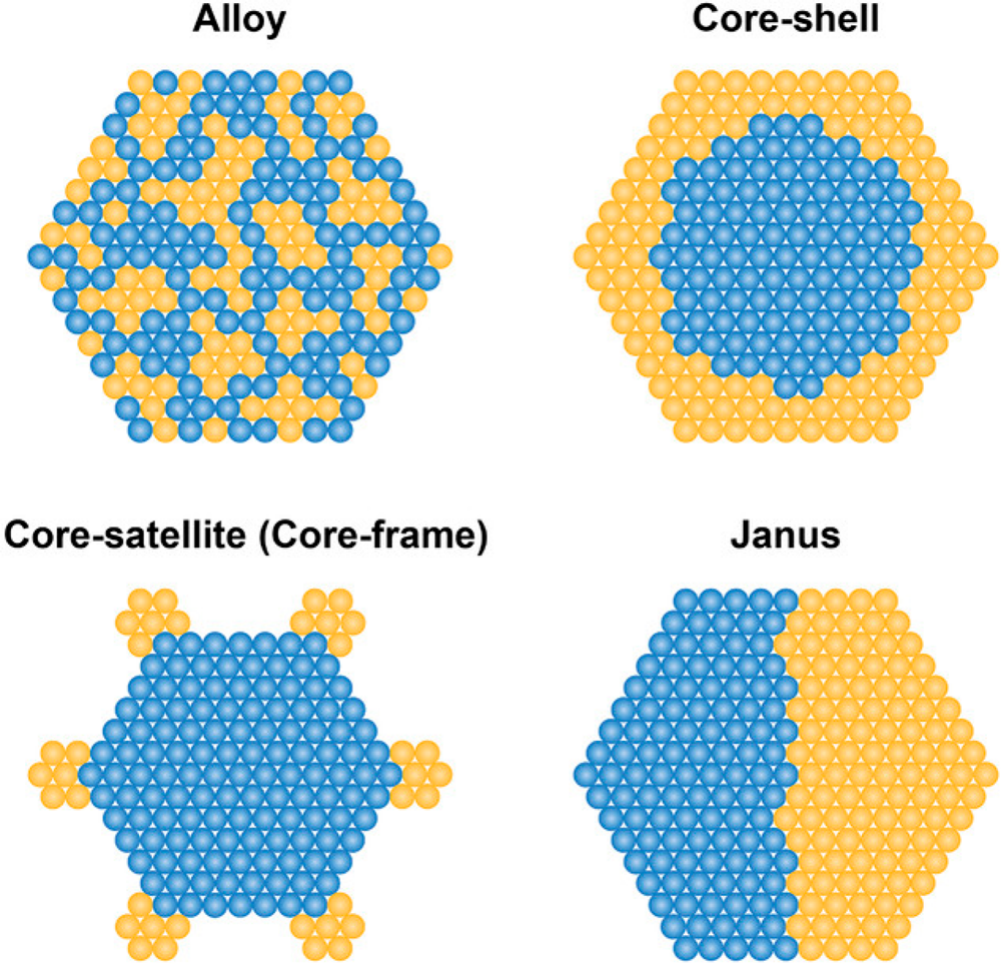
Schematic illustration of several types of bimetallic nanoparticles with different spatial distributions of the two metal elements within the individual bimetallic nanoparticles. Reproduced with permission from ref (41). Copyright 2021 Wiley-VCH.

As illustrated in Figure 5, the presence of more atoms in bimetallic nanoparticles also leads to the complexity in terms of the spatial distribution of the two metal elements within the nanoparticles.^41^ In an ideal scenario, the location of the two metal elements in intermetallic nanoparticles can be determined according to the crystallographic structure. However, in most of the real systems comprising bimetallic nanoparticles, the two metal elements may form various types of spatial distribution, such as random alloy, core–shell structure, and Janus-type structure.^42^ Moreover, a mixture of the above-mentioned distribution pattern may coexist in a single bimetallic nanoparticle, as observed in experimental and theoretical studies, making it a great challenge to precisely describe the spatial distribution of the metal elements.^43,44^

To simplify the situation, one may focus on the surface or subsurface structural features of the bimetallic nanoparticles because these regions are more relevant in catalysis. In practical catalysts, the surface of a bimetallic nanoparticle is usually rough, with multiple types of surface structures such as terrace, corner, flat facet, and defective sites. The coordination number of the surface atoms has been proposed as a key factor that determines the activity, because the adsorption of reactant and intermediate on the surface atoms is influenced by the neighboring atoms.^45^ It has been revealed in the literature that, for a specific catalytic reaction, it may follow different reaction pathways when the reactant is adsorbed on different surface sites.^46^ The site-specific catalytic behavior has already been established in some monometallic system,^47^ and it can be expected that such behavior/phenomenon will be more complicated when dealing with the bimetallic system.

Nevertheless, the combination of bimetallic nanoparticles with a solid carrier will further increase the complexity of the geometric structural features of bimetallic nanoparticles due to the formation of metal–support interface. The chemical bonding between the bimetallic nanoparticles and the support is related to the chemical properties of the two components. For instance, metal–O bonding can be present in oxide-supported bimetallic nanoparticles. When the support is changed to carbon-based materials, the metal–C/N/S bonding may serve as the linkage at the metal–support interface. Besides, due to the difference in metal–support affinity of the two metal elements, local segregation may appear at the interface between bimetallic nanoparticles and the support.

**Figure 6:**
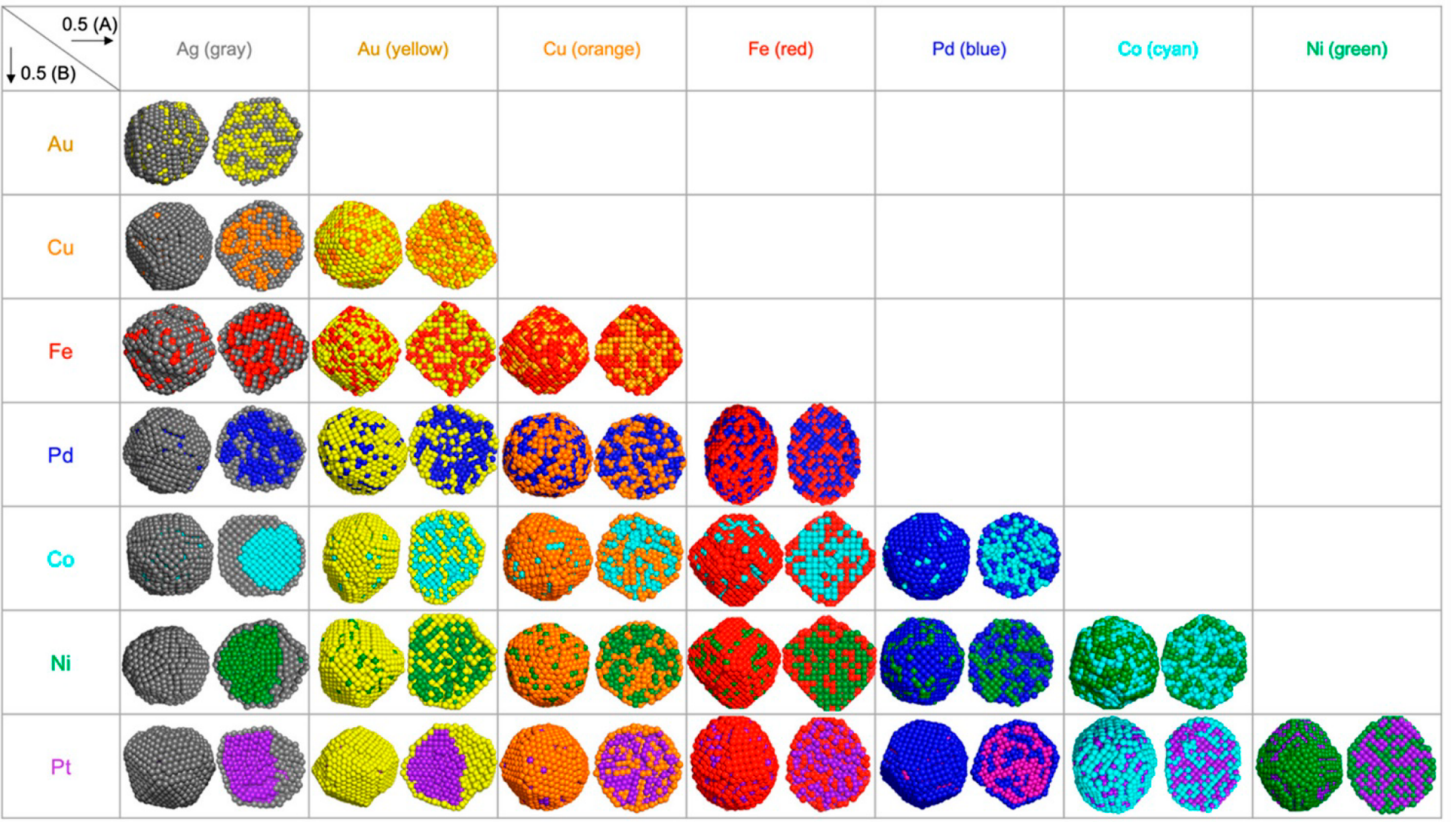
Results of the combined simulations of molecular dynamics (MD) and Monte Carlo (MC) for 0.5(A):0.5(B) composition for 28 combinations of bimetallic nanoparticles. For different combinations of two metals, the thermodynamically stable configurations give different spatial distributions of the metal elements, spanning from alloy structure to core–shell structure. Reproduced with permission from ref (50). Copyright 2021 American Chemical Society.

Because of the above reasons, the theoretical modeling of a bimetallic nanoparticle is not an easy task. There are already some studies on modeling of the morphology and possible segregation of the bimetallic nanoparticles under various conditions.^48^ For instance, a genetic algorithm is employed to predict the composition of thermodynamically stable bimetallic nanoparticles comprising tens of thousands of atoms.^49^ Because it is very difficult to measure the three-dimensional atomic structures of bimetallic nanoparticles, a fast modeling approach is helpful to give an estimation of the plausible geometric structure and composition ordering (see Figure 6).^50^ Moreover, the simulated structures of bimetallic particles can be used as models to correlate with the proposed structures derived from the experimental characterizations, as will be further discussed later in this review.

#### 2.3.2 Electronic Structures of Bimetallic Nanoparticles

Different to bimetallic nanoclusters whose electronic structures can be varied markedly by changing one atom, the variation of the electronic structures of bimetallic nanoparticles will be less sensitive to the change of particle size because their energy diagram become continuous while the small nanoclusters are not. Usually, noticeable changes are observed with bimetallic nanoparticles comprising plasmonic metals (such as Au, Ag, and Cu) when modulating the particle size because the d-band centers of these metals are far than the Fermi energy level.^51^ Such effects are not significant for platinum group metals because their d-band centers are close to the Fermi level. The influence of the composition is, to some extent, more remarkable than particle size. As shown with AuAg nanoparticles, the peak of the adsorption band in UV–vis spectra can be modulated from 400 nm (pure Ag) to 550 nm (pure Au) when tuning the composition of the bimetallic nanoparticles.^52^ The shift of the band positions in UV–vis adsorption spectra is caused by the shift of the d-band center of the bimetallic nanoparticles, which is induced by mixing Au and Ag.

Besides, the influence of the morphology of the bimetallic nanoparticles on electronic structures cannot be neglected, especially for plasmonic metals.^53^ The position of the plasmonic adsorption band of AuPd nanoparticles will shift when tuning the shape of the bimetallic nanoparticles from sphere to nanorods.^54^ The morphology-dependent electronic structures and related optical properties will be reflected in the catalytic applications, which will be further discussed in this review.

To correlate the electronic structures of bimetallic nanoparticles with the metal–reactant interaction or performance in catalytic tests, some descriptors are proposed to establish a concise relationship, among which the d-band model is widely used for evaluating the formation and stabilization of intermediates on the metal surface.^55^ The center of the d-band electron energy diagram (density of states) can correlate with the adsorption of the reactant/intermediate on a specific metal surface.

### 2.4 Comparison between Different Types of Bimetallic Entities

The geometric and electronic structures of the three types of bimetallic entities are greatly different from each other. Herein, we would like to emphasize the difference in terms of the number of exposed metal sites and the chemical states.

Limited by the atomicity, the number of metal atoms for adsorption and activation of reactants in the isolated binuclear sites will be quite limited. Therefore, in many cases, it is proposed that the support will participate and play a critical role in the catalytic process. Because there are multiple metal atoms exposed in the nanoclusters and nanoparticles, the adsorption and activation of reactants can be achieved by the synergy of multiple metal atoms. Certainly, the support in the metal catalysts comprising bimetallic nanoclusters or nanoparticles can also make a significant impact, but their role is not as indispensable as that of the catalyst based on binuclear sites.

As a result of the bonding interaction with the support for stabilization, binuclear bimetallic sites are usually positively charged due to the charge transfer from the metal atoms to the support. For instance, Pt(II) and Zn(II) are formed in the supported binuclear Pt–Zn sites prepared via a surface organometallic approach. In the case of bimetallic nanoclusters, the chemical states may vary from one atom to another within a single cluster, depending on the atom’s coordination environment. Due to the presence of metal–metal bonding, the majority of the atoms will be or close to the metallic state, while the metal atoms at the interface between the cluster and the support/ligand may become positively charged due to the electron transfer from the cluster to support/ligand. For bimetallic nanoparticles, the vast majority of the metal atoms are in the metallic state, except for those on the surface or at the metal–support interface, whose chemical states could be modified by the ligands/reactants/support.

In addition to metal–support interactions, the structural features of bimetallic entities are also greatly influenced by the metal–reactant interactions, which will be reflected in their stability and associated structural transformations under the conditions of catalytic reactions. For instance, it has been shown in numerous systems made by bimetallic nanoparticles that segregation of the two metal elements occurs when varying the atmosphere because of the discrepancy in the interactions of the two metal elements with the reactant.^56−58^ Furthermore, the high structural flexibility of subnanometer monometallic species (isolated metal atoms and clusters) have also been demonstrated in experimental and theoretical works, which show even higher sensitivity than the nanoparticulate counterparts in response to the variation of the atmosphere. The particle size and coordination environment of metal atoms and clusters can exhibit dramatic changes when altering the atmosphere/solvent/ligand.^59−61^ In this sense, when discussing the structural features of bimetallic entities, the discussion should be made in the context of the support/ligand and the atmosphere/solvent. The potential structural transformations during treatments or under reaction conditions should be paid special attention to, as will also be emphasized in other sections of this Review.

## 3 Synthesis of Different Types of Bimetallic Entities

### 3.1 Synthesis of Binuclear Bimetallic Sites

Selective generation of binuclear bimetallic sites on the surface is quite challenging because of the difficulty to control the proximity and coordination environment of the two metal atoms.^62^ In this section, we will summarize several methods that allow the formation of binuclear sites on a solid carriers.

#### 3.1.1 Direct Immobilization

A straightforward strategy for generating supported binuclear metal sites is to load two metal precursors on the support simultaneously. For instance, using a defective C_3_N_4_ material as the support, Ru and Pt atoms were loaded on the C_3_N_4_ support by a photoassisted deposition process.^63^

Different from the preparation of supported isolated monometallic species, it is recommended to use a bimetallic metal complex as the precursor to improve the possibility of forming binuclear bimetallic sites. Fu et al. have shown the generation of Mo–Ir species on TiO_2_ by grafting organometallic Mo–Ir complex and subsequent calcination treatment for removal of the organic ligands.^64^ In principle, this method can also be applied to the preparation of a wide scope of binuclear species if the bimetallic organometallic complex can be well dispersed on the support and the sintering of the metal species can be avoided during the thermal treatments.

#### 3.1.2 Atomic Layer Deposition

Atomic layer deposition (ALD) is a method that allows controlling the growth of metal species on solid carried with high precision. It has been applied for the preparation of numerous supported single-atom catalysts by using an organometallic complex as the precursor and solid carrier with anchoring groups as the support.^65^ To form binuclear bimetallic sites by ALD, one straightforward strategy is to employ a bimetallic organometallic complex as the precursor of the ALD process so that the resultant material comprises binuclear sites on the support.

Another approach is to perform consecutive deposition of the two metals, which requires the selective anchoring of the secondary metal near to the primary metal species. For example, after the deposition of isolated Pt atoms on N-doped carbon nanotubes, Ru atoms were selectively anchored to the neighboring sites of the Pt atoms by ALD, resulting in the formation of Pt–Ru binuclear sites.^66^

#### 3.1.3 Surface Organometallic Approach

**Figure 7:**
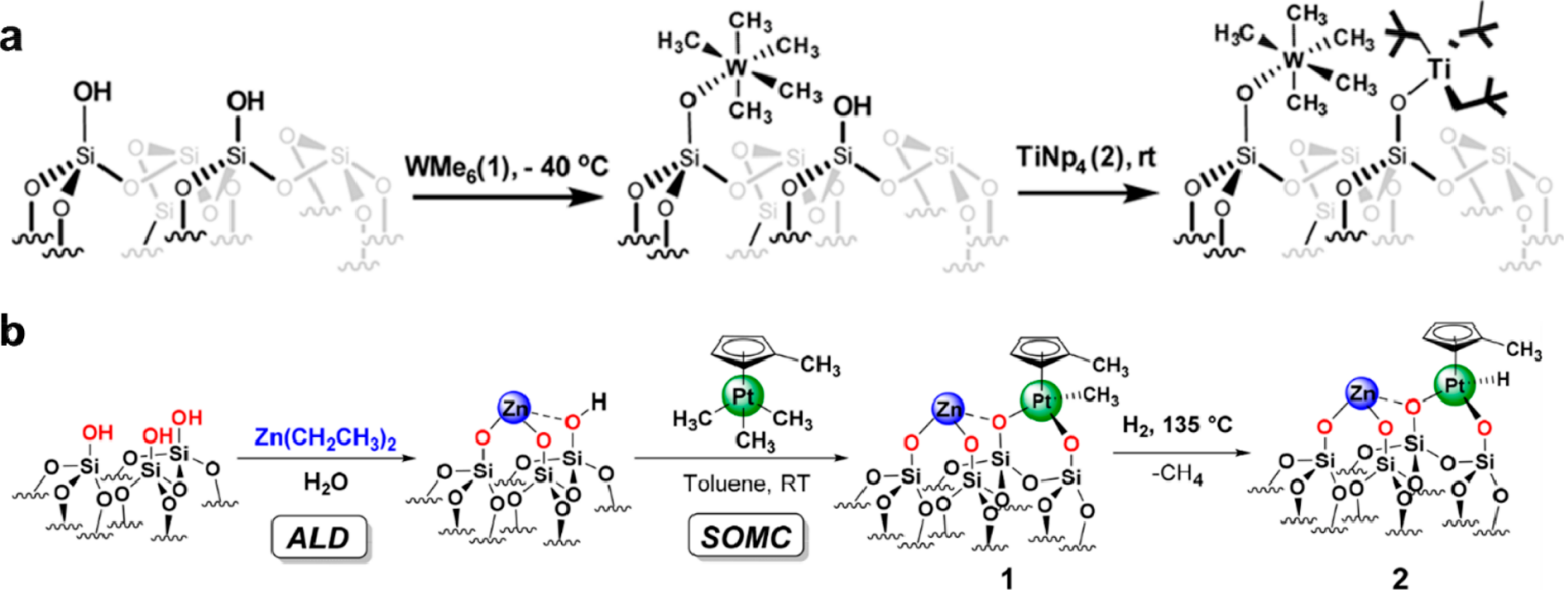
Generation of binuclear bimetallic sites on solid carrier via the reactions of surface organometallic precursors. The organometallic complexes can be grafted on silica through the reaction condensation reaction with the −OH groups on the silica surface. Generation of bimetallic W–Ti sites (a) and Pt–Zn sites (b) on silica. Reproduced with permission from ref (67). Copyright 2017 American Chemical Society. Reproduced with permission from ref (68). Copyright 2018 American Chemical Society.

Surface organometallic chemistry has been demonstrated as an effective approach for the generation of analogue species as molecular organometallic complexes in order to transform the homogeneous catalytic reaction into a heterogeneous process. By taking dehydrated silica as the support, organometallic complexes are grafted on silica by a condensation reaction between the hydroxyl group on silica and the organometallic complex. In principle, this strategy can also be extended to bimetallic systems which can be achieved by consecutive grafting of the organometallic precursor (see Figure 7). For instance, a stepwise anchoring of organometallic complexes (W(Me)_6_ and Ti(Np)_4_, Np = neopentyl) on silica resulted in the formation of neighboring W and Ti species on silica.^67^ The presence of neighboring W and Ti species is confirmed by the ^1^H–^1^H multiple-quantum NMR spectroscopy. In another work, a bimetallic Pt–Zn catalyst based on the surface grafting of organometallic Zn and Pt complex was prepared via a combination of ALD (deposition of isolated Zn species) and surface grafting of Pt species.^68^

It can be expected that the consecutive grafting approach cannot guarantee the homogeneity of the formation of neighboring W and Ti species because of the random distribution of the grafted W and Ti species. To ensure the formation of binuclear metal species on the support, a bimetallic complex is employed as the precursor for the grafting process so that the final support catalyst comprises abundant proximate binuclear species. Taking bimetallic Ta–Ir complex as the precursor, Camp et al. have developed a supported Ta–Ir catalyst for H–D exchange reactions, which showed much higher activity than the homogeneous Ta–Ir complex and supported monometallic single-site Ta species.^69^

#### 3.1.4 Solid-State Transformation Approach

**Figure 8:**
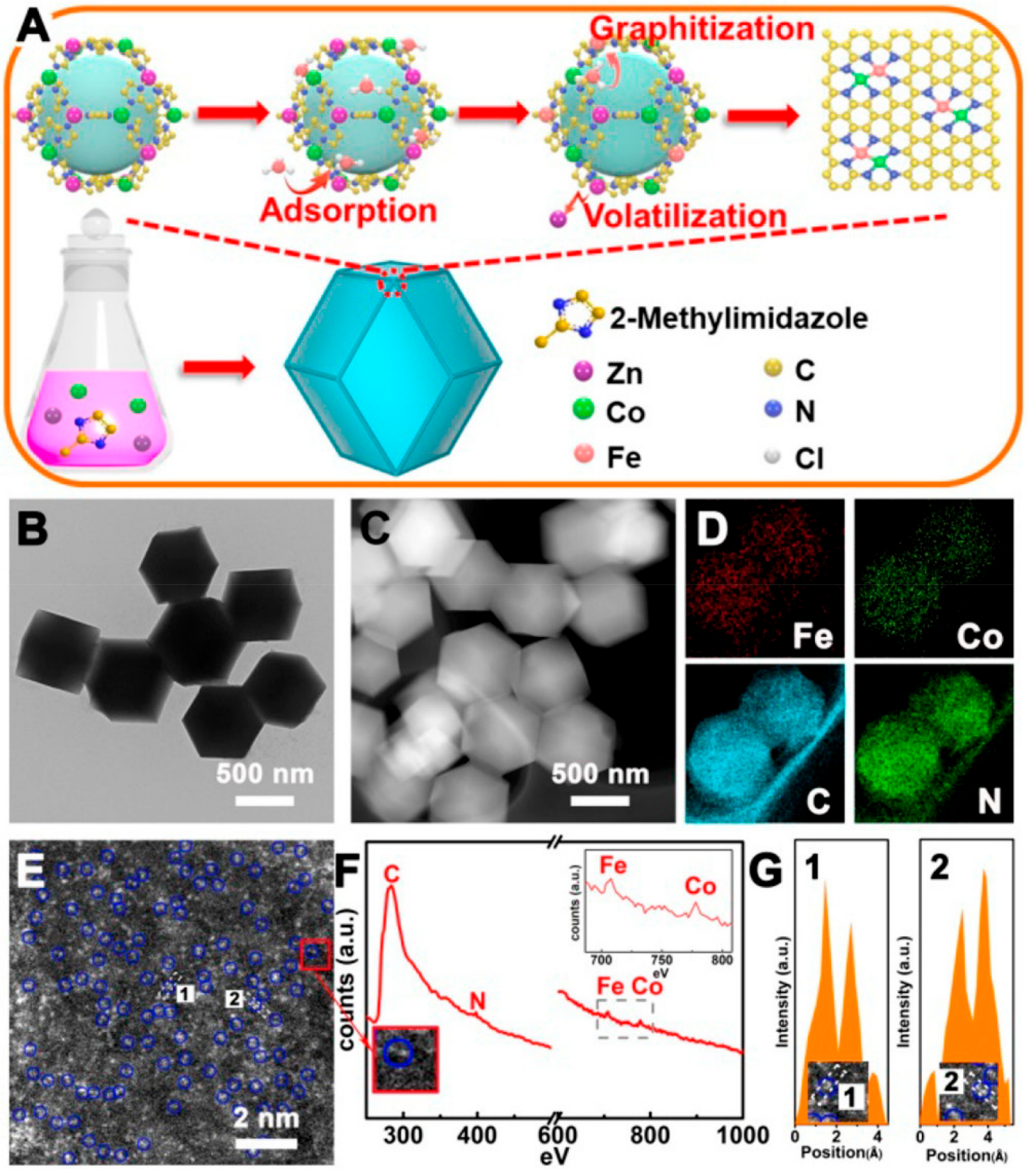
Generation of binuclear FeCo sites via the pyrolysis of CoFe-MOF precursor. (A) Illustration of the formation process of FeCo binuclear sites on N-doped carbon. (B–G) Structural characterizations of the FeCo sites on the N-doped carbon support. (B) TEM image and (C) STEM image of FeCo binuclear sites on N-doped carbon, and (D) corresponding EELS mapping images of Fe, Co, C, and N. (E) High-resolution HAADF-STEM image of FeCo binuclear sites on N-doped carbon, (F) EELS spectrum taken on the red rectangle region indicated in (E), and (G) corresponding contrast profiles across the enlarged areas indicated in (E). Reproduced with permission from ref (72). Copyright 2020 American Chemical Society.

In addition to the immobilization and surface organometallic strategy described above, binuclear metal sites can be formed via solid-state transformation, in which the metal species are introduced and stabilized on the solid carrier during treatments such as high-temperature annealing or pyrolysis. By pyrolyzing a composite material comprising two metal precursors, it is possible to form binuclear bimetallic sites on the solid carrier (usually carbon-based materials), as demonstrated with the Co–Pt–N–C and Ni–Fe–N–C materials.^70,71^ As demonstrated in Figure 8, binuclear FeCo sites are formed in N-doped carbon matrix by pyrolysis treatment of a MOF structure comprising the metal precursor compounds.^72^ The decomposition of MOF structure with zinc nodes will cause the formation of defective sites in the N-doped carbon because of the evaporation of Zn atoms, which can serve as the anchoring sites for binuclear sites.^73^ Due to the difficulties to control the pyrolysis process, it is highly challenging to achieve a high homogeneity in terms of the spatial distribution of the two metal species. In other words, the direct pyrolysis strategy may result in the generation of a broad scope of species in the final materials, including binuclear bimetallic sites, bimetallic nanoclusters/nanoparticles, and monometallic nanoclusters/nanoparticles.

The formation of binuclear bimetallic sites on carbon support can also be achieved by a subsequent anchoring strategy.^74^ The metal atoms anchored primarily on the support can serve as the binding sites for the secondary metal atoms during the high-temperature pyrolysis process through the formation of metal–metal bonding, which facilitates the selective generation of binuclear bimetallic sites. Taking Co-MOF material comprising isolated Co atoms in the MOF matrix as the starting material, binuclear Co–Fe species are formed after the introduction of Fe precursor into the Co-MOF and subsequent high-temperature pyrolysis treatment.^75^

**Figure 9:**
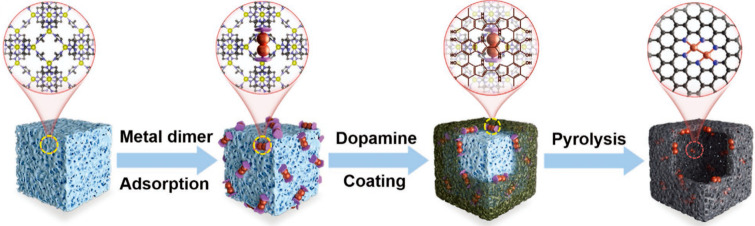
Schematic illustration for generation of binuclear bimetallic sites embedded in carbon-based solid carrier via the solid-transformation approach. Binuclear bimetallic complexes were loaded on porous metal–organic framework (ZIF-8) as the precursor and then a polydopamine layer was coated on the ZIF-8 particles, before being subjected to high-temperature pyrolysis treatment. Reproduced with permission from ref (76). Copyright 2022 Wiley-VCH.

In the above-mentioned approaches, there is always a considerable amount of monometallic binuclear sites generated on the carbon matrix. To improve the probability of generation of bimetallic binuclear sites in the final materials, binuclear bimetallic complexes can be employed as the precursors and loaded on the solid carrier before the high-temperature pyrolysis treatment (as illustrated in Figure 9).^76^ In this way, the intimacy of the two metal atoms will be greatly improved in comparison to the methods in which the metal species are introduced separately. In principle, this strategy can also be extended to generation of bimetallic nanoclusters on the carbon matrix by using multinuclear organometallic complex as the precursor.

#### 3.1.5 Binuclear Sites in Porous Materials

Porous materials such as zeolites and metal–organic frameworks (MOFs) with well-defined crystalline structures have been widely employed as the host for single-site metal species.^77^ In principle, by introducing extra-framework species into a porous matrix with isolated framework, metal species will lead to the formation of binuclear metal sites if the two metal atoms can have close contact. This scenario has been achieved with metal-exchanged zeolites, in which the introduced metal species have close contact with the framework Al species. Moreover, it can also occur with heteroatom zeolites such as Sn-zeolite and Ti-zeolite materials, in which the external metal species can be located at the neighboring position of the framework Sn and Ti species. However, due to the difficulty in materials synthesis and structural characterization, this type of binuclear metal site has not been carefully explored yet.

It will be more versatile and facile to generate binuclear metal sites in MOFs because of their structural diversity.^78^ By incorporating the metal species onto the organic linkers or nodes, two metal atoms with proximity can be formed, which can work as binuclear metal sites for catalysis. For instance, The Zn–O–Zr sites are produced by the reaction between the Zr_6_(μ_3_-O)_4_(μ_3_-OH)_4_ nodes of MOF-808 and ZnEt_2_, followed by a mild thermal treatment for removal of the ligands.^79^ In another work, Rh–Cu binuclear sites are formed in Cu-MOFs by replacing one Cu atom with a Rh atom in the binuclear paddlewheel nodes of the framework through transmetalation reaction.^80^

Another approach for generation of binuclear metal species in crystalline porous materials is to grafting the two metal entities into the microporous cavities. For instance, Cu^2+^ and Ni^2+^ ions were introduced into MOF-808 because of the coordination interaction between the metal ions and the ligands in the MOF-808 structure.^81^ The proximate Cu and Ni atoms form binuclear metal sites in the microporous cavity of MOF-808 with flexible distance in the ranges of 0.4–0.5 nm. If can be expected that the distance of the two metal atoms can be modulated by the size of the cavity and the binding sites for the metal species, as demonstrated with the generation of bifunctional Pd–Cu catalysts in MOF structure.^82^

### 3.2 Synthesis of Bimetallic Nanoclusters

In this section, we will summarize the methodologies for synthesis of bimetallic nanoclusters by presenting the general principle of each synthesis method and discussing the structural features of some representative materials generated accordingly. Different methods will be compared in terms of a set of criteria including the versatility, scalability, homogeneity, scope of materials, and their merits for green chemistry principles (cost, waste, toxicity, etc.).

#### 3.2.1 Size-Selected Clusters

Bimetallic nanoclusters can be generated in gas phase by size-selected method and then deposited on a substrate for subsequent studies. Their size and chemical components can be precisely controlled by the mass spectrometer and the size-selected metal clusters can be deposited on a substrate, resulting in formation of well-defined nanoclusters as model materials. It should be noted that the nanoclusters generated by size-selected method are free of capping agents, making them as ideal samples to study the intrinsic physicochemical properties of the metal clusters. For instance, Ag_9_Pt_2_ and Ag_9_Pt_3_ clusters were generated in gas phase and then deposited on an ultrathin alumina support to elucidate how Pt and Ag can work in a synergistic way for CO oxidation reaction.^83^ Although this method can be theoretically applied to all the metal elements to generate metal clusters with desired size and compositions, the yield of the target materials is quite low because of the limited flux of ion beam. Therefore, up to this date, this method can only be used for preparing model catalysts for fundamental studies.^84^

Size-selected monometallic nanoclusters can serve as the binding sites for the second metal, leading to the formation of bimetallic nanoclusters with precise composition. For instance, the addition of Sn to size-selected Pt clusters was achieved by a reaction between reduced Pt clusters and SnCl_4_.^85^ Although the resultant bimetallic nanoclusters derived from the modification may not show high uniformity as the ones from direct size selection, it can effectively broaden the scope of bimetallic nanoclusters by chemical modifications of size-selected monometallic clusters.

##### 3.2.2 Liquid Phase Synthesis

Wet-chemistry synthesis is one of the most widely used and versatile methods for the production of bimetallic nanoclusters. The general principle for this method is usually to reduce the two metal precursors simultaneously in the presence of solvent and/or capping agents (polymers or ligands) to form monodispersed bimetallic nanoclusters.^86,87^ The growth kinetics of the two metal elements should be carefully controlled to obtain the desired materials with narrow size distribution and high uniformity in chemical compositions. By tuning the synthesis parameters (precursor, solvent, capping agent, reductant, temperature, reaction time, etc.), the particle size and the chemical composition can be controlled in a fairly wide range. Especially, by using organic ligands with functional groups (such as thiol groups) that can deliver strong metal–ligand interaction, bimetallic nanoclusters with tunable chemical components and geometric structures have been prepared and their well-defined crystallographic structures are more regular than the bimetallic nanoclusters synthesized in the presence of capping agents with less strong metal–ligand coordination interaction (such as glycol and polyvinylpyrrolidone).^88^ Nevertheless, dendrimers with well-fined molecular structures can also serve as the host for bimetallic nanoclusters. The sizes of the nanoclusters encapsulated in the dendrimers can be modulated by the synthesis conditions and the structures of the dendrimers.^89^ However, the high costs and low yields of dendrimers limits their applications for large-scale preparation of dendrimer-encapsulated bimetallic nanoclusters.

High yields of bimetallic nanoclusters can be achieved in relatively short reaction times (from minutes to hours), and the scope of the wet-chemistry synthesis is quite wide. The resultant monodispersed bimetallic nanoclusters can be directly used for catalytic results or further deposited on solid carriers, although their well-defined structures may change after being subjected to reaction conditions, which will be discussed later in this review.

One drawback of liquid-phase synthesis is the use of capping agents and/or ligands, which may block the surface sites of the metal clusters. This blocking effect is particularly significant, with clusters stabilized by strongly coordinated ligands, such as AuPd, AuAg, and AuCu clusters protected by thiolate groups. Although the capping agents can be removed or partially removed by postsynthesis treatments, undesired structural reconstruction (sintering or change of the spatial distribution of the chemical components) may occur. Nevertheless, for large-scale production of bimetallic nanoclusters for practical applications, the cost and the treatment of the solvent used in the synthesis should be taken into account.

##### 3.2.3 Impregnation

For the preparation of bimetallic nanoclusters, direct impregnation of the metal precursors on the solid carrier can work when the growth kinetics of the two metals are matched. Otherwise, it may cause the segregation of the two elements and the formation of separated monometallic clusters. By in situ IR and EXAFS spectroscopic characterization, formation of bimetallic OsRu clusters via Os–Ru bonding has been observed during the reduction of MgO-supported Os_3_(CO)_12_ and Ru_3_(CO)_12_ complex by H_2_.^90^ At 333–398 K, the Ru–CO bonding is dissociated due to the reduction by H_2_ and the formation of Ru–Os bonding is observed at temperature above 398 K. The EXAFS data indicate the formation of Ru_2_Os clusters after reduction treatment at 423 K. The formation of bimetallic clusters depends on the decomposition kinetics of the two metal precursors and the stability of the tiny metal clusters on the support. In the case of Rh and Os, the formation of bimetallic RhOs clusters is not observed when applying the H_2_ reduction treatment to MgO-supported Os_3_(CO)_12_ and Rh(C_2_H_4_)(acac) (acac = acetylacetonate) complex at temperature up to 393 K. Because of the lower stability of Rh(C_2_H_4_)(acac), the formation of Rh_4–6_ clusters is observed after reduction treatment at 393 K while the Os_3_(CO)_12_ species remain intact under the same conditions.^91^ Consequently, the formation of small RhOs clusters will be more difficult than the case of RuOs. Therefore, one strategy to facilitate the contact between the two metals is to use bimetallic complexes as the precursor for impregnation. After the decomposition of the bimetallic complexes, bimetallic nanoclusters with uniform chemical components are formed on the solid support. To avoid the segregation and/or sintering of the two elements, the postimpregnation treatments should be carefully proceeded to ensure the formation of bimetallic nanoclusters on the support.

In order to facilitate the formation of bimetallic nanoclusters, a potential strategy is to employ consecutive impregnation of metal precursors on the solid carrier because the first impregnated metal can serve as the binding site for the second metal, especially when the impregnated metal species are reduced. This method has already been successfully demonstrated for the preparation of bimetallic nanoparticles with core–shell structures.^92,93^

##### 3.2.4 Strong Electrostatic Adsorption

**Figure 10:**
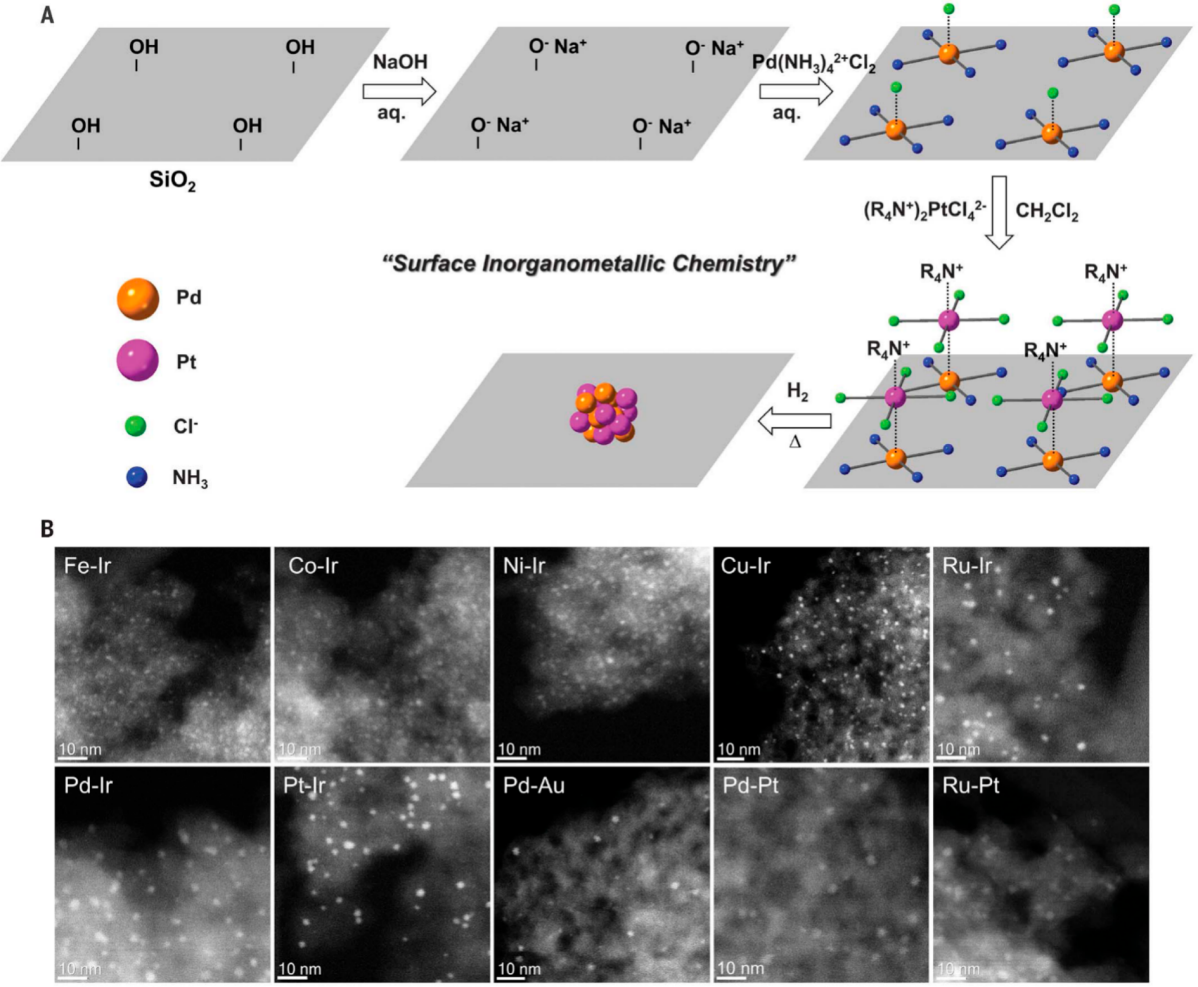
Generation of bimetallic nanoclusters on silica by surface reaction of the molecular metal precursors. (A) Schematic illustration of the preparation procedure of bimetallic nanoclusters by loading metal precursors on modified silica support. (B) HAADF-STEM images of 10 types of supported bimetallic NPs synthesized by this approach. All scale bars are 10 nm. Reproduced with permission from ref (94). Copyright 2018 The American Association for the Advancement of Science.

Because the precursors for synthesis metal nanoclusters are mostly cationic or anionic complexes, the precursors can be adsorbed by the solid carrier by strong electrostatic adsorption. As depicted in Figure 10, by properly choosing the metal precursors and controlling the support’s surface charge property, the metal precursors are adsorbed and stabilized by the support and then transformed into bimetallic nanoclusters in subsequent calcination/reduction treatments.^95^ To achieve a more homogeneous mixing of the two elements, the two metal precursors with opposite charges were loaded on the support via strong electrostatic adsorption. Consequently, the two metal precursors formed a new bimetallic complex on the support, which can be further transformed into bimetallic nanoclusters.^94^

##### 3.2.5 Ion Exchange Method

By conventional liquid-phase ion exchange reactions, the metal precursors can be grafted on the solid carrier and then transformed into bimetallic species in postsynthesis treatments. Because of their high porosity and regular structure, zeolites are excellent hosts for generation and stabilization of bimetallic nanoclusters. In principle, bimetallic nanoclusters can be formed when performing the ion exchange reactions in the presence of two metal precursors if the two metals can be adsorbed and stabilized evenly by the host. However, the different exchange kinetics of the metal precursors may result in the random distribution and spatial segregation in the final metal-zeolite materials.^96^ To control the proximity of the two metals, the introduction of two metals is carried in a consecutive way. For instance, PtZn bimetallic nanoclusters are generated in MFI zeolite by introducing Pt^2+^ cations into a zincosilicate zeolite in order to anchor Pt species to the nearby sites surrounding framework Zn species, which are further converted to PtZn nanoclusters calcination and reduction treatments.^97^ This strategy has also been demonstrated with generation of PtSn nanoclusters in beta zeolites^98^ and should be in principle applicable for preparation of a broad scope of materials by taking metal-zeolite materials as the support.

Ion exchange reactions can occur between monodispersed particles and molecular metal complex, as widely demonstrated with the preparation of multicomponent colloid nanoparticles.^99^ By galvanic replacement, the chemical composition of metal nanoclusters can be altered in a controllable manner.^86^ For example, the galvanic reaction between Pd_147_ cluster and Au^3+^ ions lead to the formation of isolated Au sites on Pd cluster.^100^ Of course, this method can also be applied to clusters with smaller sizes, and the doping level of the second metal into the parent metal cluster can be tuned by controlling the galvanic reaction.^101^ Furthermore, by employing ligand-protected metal clusters as the seed, the generation of bimetallic nanoclusters via a controllable galvanic replacement by varying the types of ligands, which has already demonstrated by galvanic replacement reactions with metal nanoparticles.^102^

##### 3.2.6 Synthesis in Confined Environment

The impregnation and static adsorption are versatile and effective methods for generation of bimetallic nanoclusters on solid surface. However, the resultant metal clusters may suffer severe sintering during treatments under harsh conditions. One way to address this challenge is to impose an external constraint on the bimetallic nanoclusters to restrict their mobility. Porous supports (usually with pore sizes below 10 nm) can be the ideal carrier to host bimetallic nanoclusters if the metal species can be introduced into the porous environment.

By impregnation or ion-exchange method, part of the metal precursors can be introduced into the porous environment. For instance, small PtSn and PtZn nanoclusters are generated by introducing molecular Pt precursor into Sn-β zeolite or zincosilicate zeolite structure, respectively. The Sn and Zn species in the zeolite framework can serve as the binding sites for Pt and the rigid zeolite framework also protect the PtSn and PtZn nanoclusters from sintering into large particles.^97,98^ This strategy has already been demonstrated with the synthesis of PtZn and PtSn nanoclusters in MFI and beta zeolites.^104,105^ However, the ion-exchange method will produce a large portion of the metal species may be located on the surface or subsurface of the porous materials.

**Figure 11:**
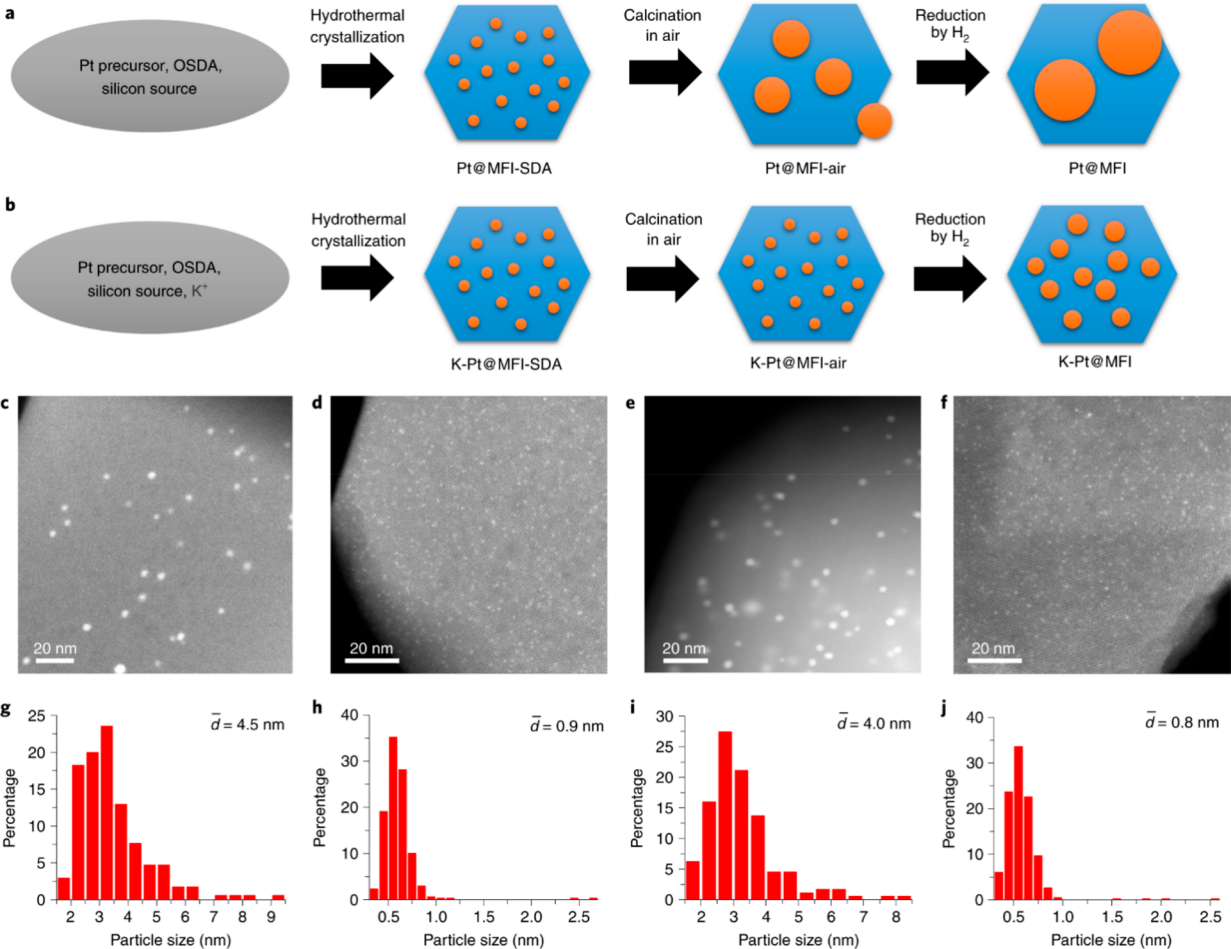
One-pot synthesis of Pt-zeolite materials. (a,b) Schematic illustration of the formation process of Pt@MFI and K-Pt@MFI samples by one-pot synthesis. (c–f) STEM images of Pt-zeolite samples after reduction by H_2_ at 600 °C: K-free Pt-MFI (c), K-Pt@MFI (d), K-free PtSn@MFI (e), and K-PtSn@MFI (f). (g–j) The size distributions of Pt particles in different Pt-zeolite materials: K-free Pt-MFI (g), K-Pt@MFI (h), K-free PtSn@MFI (i), and K-PtSn@MFI (j). The average particle size is calculated according to *d* = Σ*n*_*i*_*d*_*i*_^3^/Σ*n*_*i*_*d*_*i*_^2^. *n*_*i*_ stands for the number of the particle with a size of *d*_*i*_. Reproduced with permission from ref (103). Copyright 2019 Springer Nature Limited.

To improve the efficiency for encapsulating the metal species by the porous scaffold, the introduction of metal species can be performed during the synthesis of the porous materials. This one-pot synthesis approach has been demonstrated effectively with the preparation of bimetallic metal clusters in microporous zeolites. For instance, bimetallic PtPd nanoclusters with average particle sizes of ∼1.5 nm in ZSM-5 zeolite (MFI-type aluminosilicate zeolite).^106^ In another work, as shown in Figure 11, bimetallic PtSn clusters with sizes of 0.5–0.6 nm are formed in the sinusoidal channels of pure silica MFI zeolite by introducing alkali metals into the synthesis mixture for stabilizing the atomically dispersed metal species during high-temperature calcination and reduction treatments.^103^ This strategy should also be applicable for the synthesis of bimetallic nanoclusters within metal–organic framework (MOF) structures because the encapsulation of Pt and Pd nanoclusters in MOF crystallites have already been realized.^107^

Thanks to the porous scaffold surrounding the bimetallic nanoclusters, size-selective catalysis can be achieved with those confined metal catalysts. However, the accessibility of the metal clusters is also limited to small molecules such as light alkanes, CO/CO_2_, and NOx, which will be further discussed in the sections on catalytic applications of confined metal catalysts in this review.

##### 3.2.7 Postsynthesis Treatments

As reported in the literature, the morphology, particle size, chemical components, and metal–support interface of metal nanoparticles can show dynamic structural transformations as responses to the change of the external environment (temperature, atmosphere, pressure etc.). For instance, the exposed facet of the PtCo bimetallic nanoparticles and the arrangements of Pt and Co within the nanoparticles evolves during the high-temperature annealing treatment.^108^ On one hand, such environment-induced structural changes should also occur with bimetallic nanoclusters, and the consequences should be carefully considered when using the as-synthesized bimetallic nanoclusters for catalytic applications, because the bimetallic nanoclusters should be more sensitive to the environment than the bimetallic nanoparticles. However, the environment-induced structural transformations have not received enough attention in the works focused on synthesis and/or catalysis with bimetallic nanoclusters. In many cases, the organic ligands capped on the surface of ligand-protected bimetallic nanoclusters need to be removed in order to liberate the metal sites for catalytic reactions. However, during the removal of the ligands, the structural configuration of the bimetallic nanoclusters could be modified, which should be carefully followed.

On the other hand, further modifications on the structures of the bimetallic nanoclusters are also possible when appropriate postsynthesis treatments are employed. One example of this strategy is shown in the modulation of bimetallic PtSn clusters by high-temperature reduction treatment.^16^ With longer reduction time with H_2_ or increasing the reduction temperature, a more intimate contact between the Pt and Sn species can be achieved, resulting in the formation of Pt clusters covered by Sn species in the microporous channel of MFI zeolite. In this regard, the coverage of Sn species on Pt clusters can be tuned by the postsynthesis reduction conditions.

In another example, PtCo bimetallic nanoclusters with tunable size and chemical compositions can be generated by reducing Pt/Co_3_O_4_ catalyst under desired conditions.^109^ Initially, the Pt species are atomically dispersed on the Co_3_O_4_ support and the Pt atoms agglomerate into Pt clusters after exposure to H_2_. Once the Pt clusters are formed, the reduction of the Co_3_O_4_ support will be promoted, and the reduced Co atoms can migrate into the Pt clusters as dopants, giving to the formation of PtCo bimetallic nanoclusters. This strategy could be extended for other noble metals (Pd, Rh, Ru etc.) supported on reducible solid carriers (FeOx, MnOx, NiOx, SnOx, etc.). Mild treatment conditions are recommended for generation of bimetallic nanoclusters via this approach in order to avoid the formation of large nanoparticles.

##### 3.2.8 Perspective on Precise Synthesis of Bimetallic Nanoclusters

The preparation of supported bimetallic nanoclusters with high uniformity in particle size and composition has been a long-standing goal in the field of heterogeneous catalysis. As discussed above, the following approaches have been reported in the literature for the generation of bimetallic clusters with given size and compositions: (1) size-selected clusters by physical method, (2) bimetallic clusters from organometallic precursors, and (3) ligand-protected bimetallic clusters. The bimetallic clusters generated from these strategies can be used as model systems for the fundamental understanding of the reactivities of the bimetallic clusters. However, currently, none of the above approaches can meet the requirements of preparation of supported bimetallic clusters for practical applications. In the case of the generation of bimetallic clusters from the size-selected method, the yields of the bimetallic clusters are too low for scalable production. In the case of the bimetallic clusters from organometallic precursors and ligand-protected bimetallic clusters, the decompositions of ligands are usually mandatory for liberating the active sites. However, during the removal of the organic ligands, sintering of the metal particles or segregation of the metal elements probably occur, resulting in the formation of bimetallic particles with a relatively broad distribution of particle size and composition.

To mitigate the structural deformation of the organometallic precursors and ligand-protected clusters during the ligand removal procedure, porous materials with anchoring sites can be used as the support for hosting the bimetallic precursors and protect them from sintering/segregation. This strategy can also be applied for the precise synthesis of bimetallic nanoclusters made by a metallic component and an oxide component. By using crystalline porous materials (zeolites and metal–organic frameworks) for the accommodation of subnanometer metal clusters in the microporous channels/cavities, the oxyphilic metal species can be located in the framework materials (e.g., node positions in MOFs) and then the metal clusters are anchored at these oxyphilic metal sites, giving to the formation of well-defined noble metal–oxyphilic metal combinations.

#### 3.3 Synthesis of Bimetallic Nanoparticles

The synthesis of bimetallic nanoparticles has been covered by numerous comprehensive reviews.^111−114^ As reflected in the literature, bimetallic nanoparticles can be readily prepared in controllable ways in terms of the particle size, chemical composition, spatial distribution of the two elements, and morphology. There are already mature synthesis methodologies reported in the literature for a wide scope of metal elements and for a broad range of bimetallic nanoparticles with various. The choice of the synthesis approach depends on the structural features of the target materials and the potential applications. In this section, we would like to highlight some representative works reported in recent years in terms of the synthesis of bimetallic nanoparticles/nanostructures with novel structural features.

##### 3.3.1 Single-Atom Alloys

**Figure 12:**
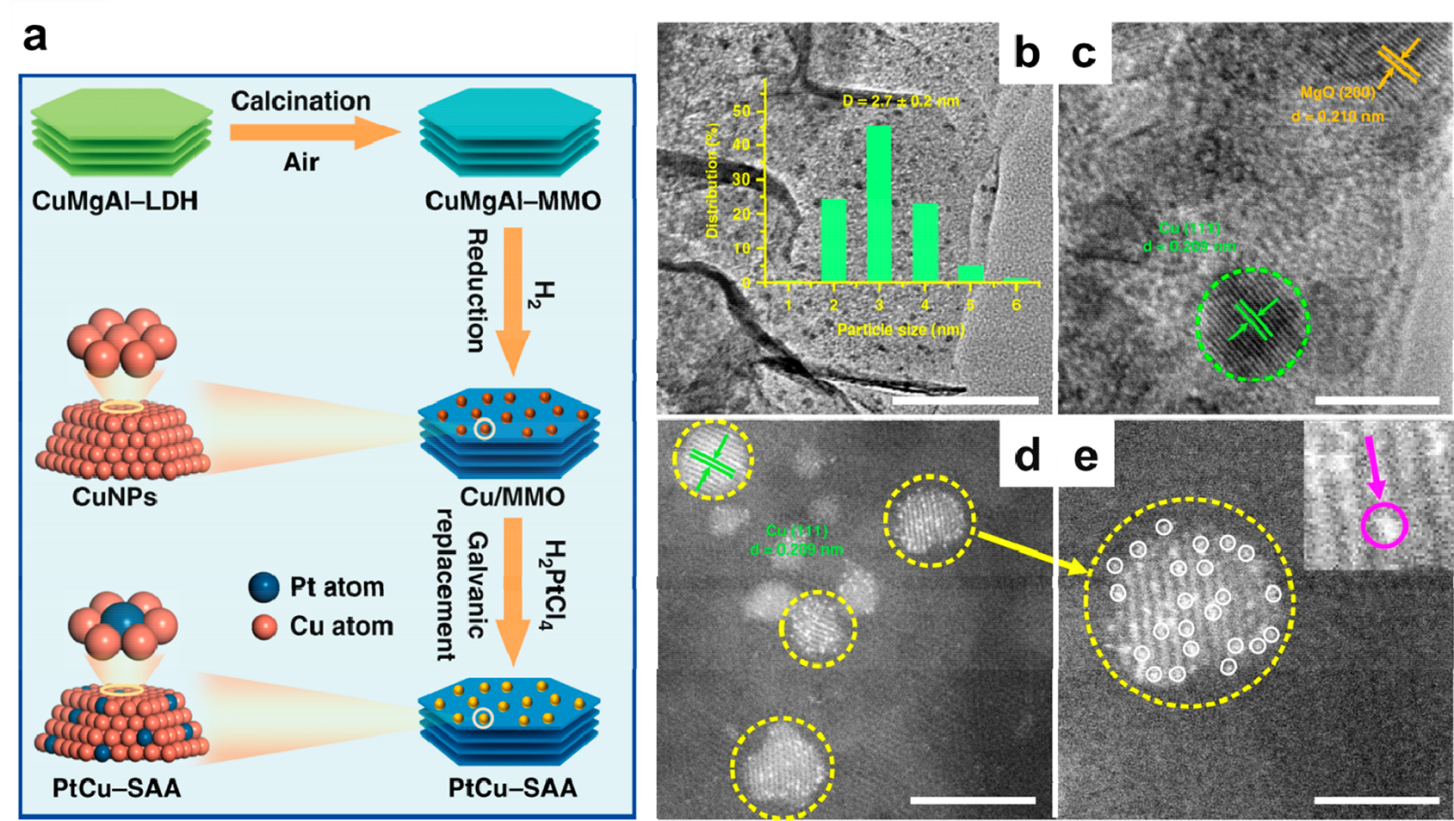
Formation of single-atom PtCu alloy nanoparticles by galvanic replacement between H_2_PtCl_6_ and metallic Cu nanoparticles. (a) Illustration of the synthesis procedure of support PtCu single-atom alloy nanoparticles. (b) TEM image, (c) HRTEM image, (d) High-resolution HAADF-STEM image of PtCu single-atom alloy nanoparticles, and (e) corresponding enlarged images. Scale bars: (b) 100 nm, (c) 5 nm, (d), and (e) 2 nm. Reproduced with permission from ref (110). Copyright 2019 Springer Nature under CC-BY license (https://creativecommons.org/licenses/by/4.0/).

In conventional alloy nanoparticles, the two metals form a mixture and show random distribution in a bimetallic nanoparticle.^115,116^ The percentage of the two metals are close to each other or at the same magnitude, and for each metal atom, it will be surrounded by both elements. By decreasing the percentage of one metal to a very low level (usually below 5%), each metal atom of the minor composition will be separated by the other metal in the bimetallic particles, giving to the formation of single-atom alloy nanoparticles. Generally, the preparation of single-atom alloy nanoparticles is similar to conventional bimetallic nanoparticles. For instance, isolated metal sites can be formed by coreduction of the two metal precursors by diluting one metal in another. Besides, preparation of single-atom alloy nanoparticles can also be achieved by galvanic replacement reaction, as demonstrated with the synthesis of PdCu and PtCu single-atom alloy nanoparticles.^110,117^ As shown in Figure 12, Cu nanoparticles formed on mixed metal oxides can reduce H_2_PtCl_6_, resulting in the formation of isolated Pt atoms in the Cu matrix. Controlling the ratio of Pt/Cu will be critical to ensure the atomic dispersion of Pt in Cu nanoparticles.

##### 3.3.2 Core–Shell Nanoparticles with Precise Thickness Control

In terms of bimetallic nanoparticles with core–shell structures, the uneven spatial distribution of the two elements causes the lattice strain at the interface of “core” and “shell” due to the mismatch of the lattice parameters of the two metallic components. The magnitude of the lattice strain depends on the degree of the mismatch of the lattice parameters and, moreover, it can be expected that the interfacial lattice strain will strongly dependent on the thickness of the “shell”, which is confirmed by a study based on high-resolution electron microscopy study.^119^ In the case of Pd@Pt nanoparticles, the lattice strain of Pt shell will be not significant once the shell is thicker for more than 5–6 monolayers. Beyond the geometric, charge redistribution between the “core” and “shell” components will occur, resulting in the variation of the electronic properties.^120^

**Figure 13:**
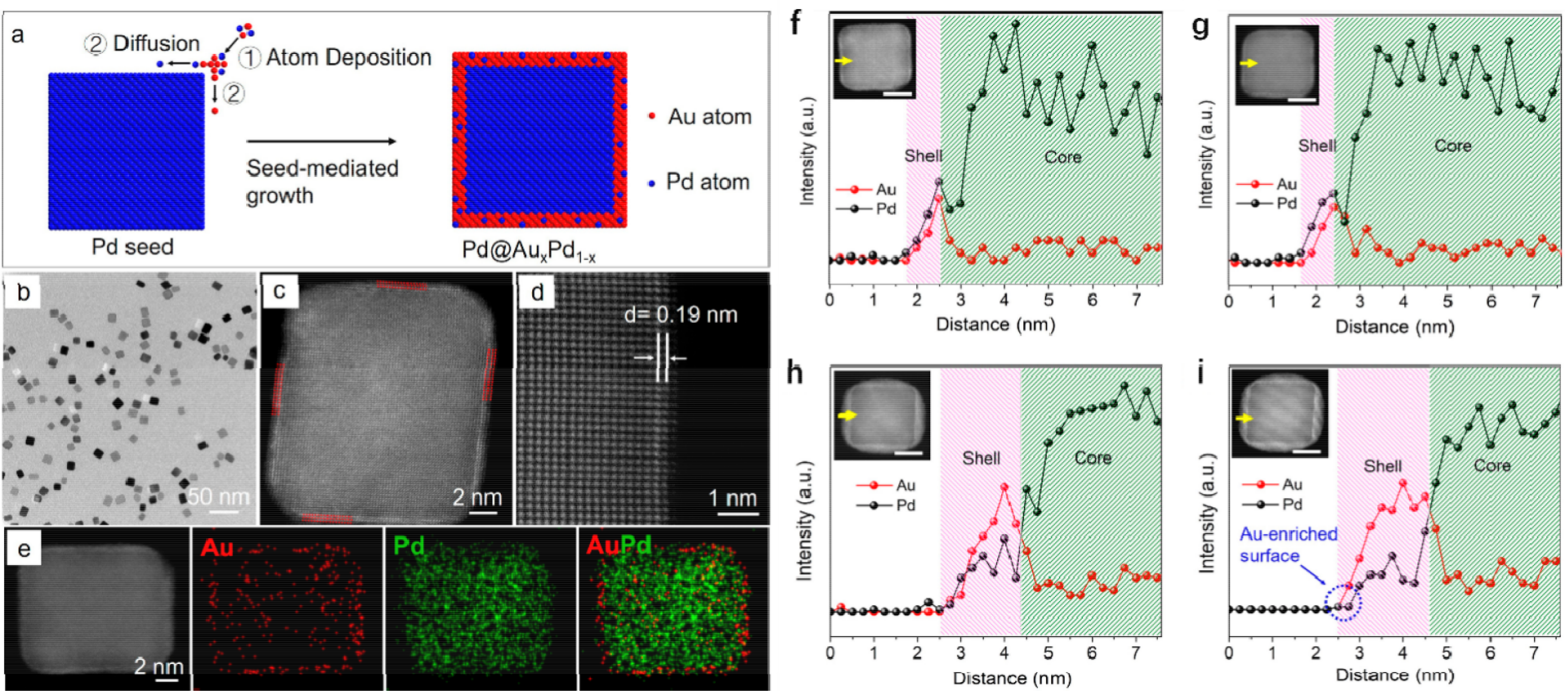
Synthesis and characterizations of Pd@AuPd nanocubes. (a) Preparation process of Pd@AuPd core–shell structure, involving the codeposition of Au and Pd atoms on Pd nanocubes. (b) Representative TEM image of the Pd@AuPd nanocubes, which exhibit very similar morphology as the Pd cubic seeds. (c) HAADF-STEM image, indicating a shell thickness of about three atomic layers. (d) Atomic-resolution HAADF-STEM image, showing a fringe spacing of 0.19 nm. (e) EDX mapping of Au (red) and Pd (green) in the core–shell structure. (f–i) Structural characterizations of Pd@AuPd core–shell structure by electron microscopy. Line scan profiles of the Pd@AuPd nanocubes for analysis of the chemical composition. (f) before and (g) after stability test for 10 000 reaction cycles and Pd@AuPd-Thick (h) before and (i) after 2000 cycles. The insets show the corresponding HAADF-STEM images of the nanocubes with yellow arrows indicating the scanning direction (scale bar: 5 nm) Reproduced with permission from ref (118). Copyright 2021 Wiley-VCH.

The shell thickness can be modulated by controlling the deposition rate of the “shell” composition on the “core” nanoparticles. For example, with a slow introduction rate of the Pt precursor to the synthesis mixture, the deposition of Pt is achieved on the entire surface of Pd nanocubes, resulting in the formation of uniform thin Pt shells of 1–6 monolayers.^121^ With the help of synthesis methodologies developed based on flow chemistry, it is feasible to achieve large-scale production of core–shell nanoparticles with precise control of the thickness of the shell component.^122^ Diffusion of metal atoms from the “core” to the “shell” during the deposition process may occur, leading to the formation of bimetallic shells, as observed with the preparation of Pd@AuPd nanoparticles (see Figure 13).^118^ The thickness of the “shell” can affect the stability of the core–shell structure under the reaction conditions for electrocatalytic production of H_2_O_2_. The sample with thin AuPd “shells” exhibit higher stability than the sample with thick “shells”.

##### 3.3.3 Intermetallic Nanoparticles

Intermetallic nanoparticles are bimetallic nanoparticles with ordered structures at atomic level and the formation of intermetallic nanoparticles usually requires high-temperature synthesis conditions.^123,124^ Though there are some works on wet-chemistry synthesis of intermetallic nanoparticles, the majority of the reported works involves high-temperature annealing treatments.^125^ For instance, Pt_3_Co intermetallic nanoparticles are formed after annealing treatment with the platinum/cobalt–carbon precursor at 900 °C, as a result of the migration of cobalt species from the carbon support to Pt nanoparticles.^126^ To avoid severe sintering of the metal particles during the high-temperature treatments, sulfur-doped carbon is employed as the support for stabilizing small bimetallic PtM intermetallic nanoparticles (<5 nm).^127^ Furthermore, core–shell Pt/PtM (M = Fe, Ni, Cu etc.) nanoparticles with intermetallic PtM core and thin Pt shell can be prepared by the combination of high-temperature annealing treatment and acid leaching of M element.^128^

The intermetallic nanoparticles comprising early transition metals (such as Fe, Co, Ni, etc.) and main group metals (such as Sn, Ga, etc.) have been intensively studied while the rare-earth elements are much less explored because of the difficulty to incorporate rare-earth elements into metallic particles.^129^ It has been found that PtCe intermetallic nanoparticles are formed after reducing Pt/CeO_2_ catalyst by H_2_ at above 900 °C. The resultant PtCe intermetallic nanoparticles are protected by thin layers of CeOx, as a result of strong-metal support interaction.^130^ Molten-salt synthesis methods have been developed to facilitate the formation of rare-earth intermetallic nanoparticles under relatively mild conditions (annealing at 650 °C).^131^ The choice of precursors for efficient incorporation of rare-earth elements into metallic particles is important, because the presence of H_2_O and Cl during the high-temperature annealing process assist the formation of bonding between noble metals and rare-earth elements.^132^

Another strategy to promote the stability of intermetallic nanoparticles is to confine them in a porous support. For example, PtRE (RE = Y, Ce and La) nanoparticles (<5 nm) can be synthesized after reducing the Pt and RE precursors at 700 °C and stabilized in hierarchical MFI-type zeolite.^133^ This work indicates that intermetallic nanoparticles with unconventional chemical compositions could be obtained by tuning the environment surrounding the nanoparticles.

##### 3.3.4 Novel Bimetallic Nanostructures

Tremendous efforts have been dedicated to tailoring the morphology of bimetallic nanoparticles and assembling the particles by design for the construction of novel bimetallic nanostructures. For instance, by acid leaching and annealing treatments, hollow PtNi nanoframeworks and one-dimensional PtNi nanowires with jagged surface have been prepared and used for electrocatalytic oxygen reduction reaction.^134,135^ These materials show enhanced activity and stability against sintering in comparison with conventional Pt/C catalyst, which are associated with the abundant undercoordinated sites on the surface and the unique morphologies. Besides, two-dimensional bimetallic nanostructures are also interesting catalytic materials.^136,137^ These thin materials with a few atomic-layer thickness can be considered as intermediate materials between nanoclusters and nanoparticles because they are made with the highly undercoordinated metal atoms at the edge and the coordinated metal atoms at planar positions. In section 7, we will discuss further the novel catalytic properties of the low-dimensional bimetallic nanostructures, such as ultrathin nanosheets or nanowires.

Substituting noble metal catalysts with non-noble metal catalysts represent an important direction for achieving sustainable processes. However, for most of 3d transition metals, their high affinity to oxygen causes the low stability of their metallic nanoparticles in air due to the oxidation of 3d metal nanoparticles into oxides. By using organometallic compounds as precursor and organic ligands as stabilizer, a series of bimetallic nanoparticles based on 3d metals have been prepared, which exhibit narrow size distributions and uniform compositions.^138−140^ In order to further improve the stability of non-noble metal catalysts, it is proposed to cover them with thin carbon layers to minimize their contact with air, which leads to the preparation of highly stable and active CoNi and CuFe bimetallic nanoparticles for a variety of hydrogenation reactions.^141−143^ The use of thin carbon layers for protecting metal nanoparticles from degradation/deactivation/leaching has also been shown with noble metals, such as the synthesis of bimetallic PtCo nanoparticles stabilized in graphene nanopockets.^144^

##### 3.3.5 Amorphous Bimetallic Nanostructures

**Figure 14:**
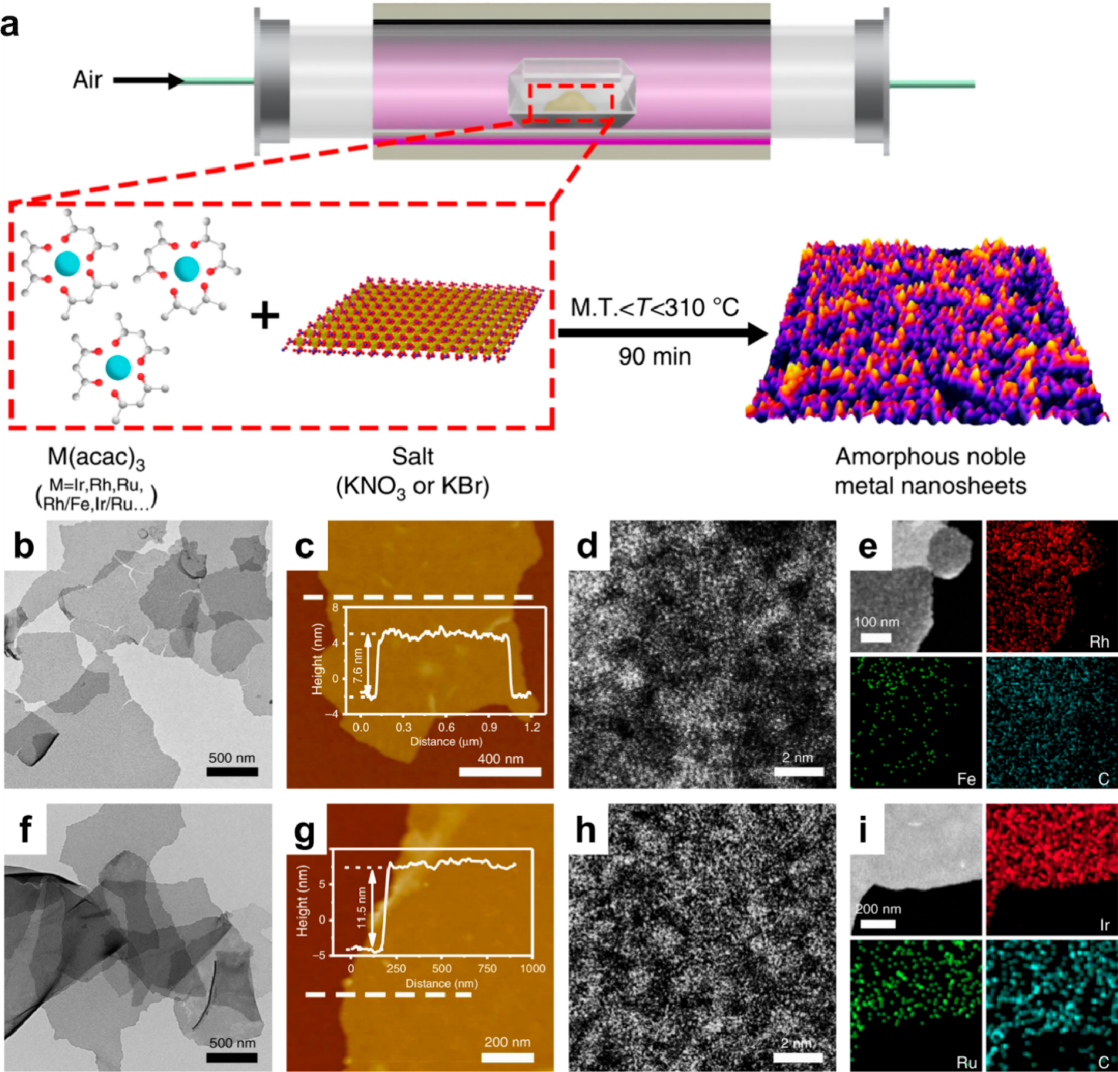
Synthesis of amorphous noble metal nanosheets. (a) Schematic illustration of the general synthetic process for amorphous noble metal nanosheets by low-temperature annealing the mixture of metal precursors and salt. (b–e) Structural characterization of amorphous RhFe nanosheets prepared by the salt-assisted annealing method. (f–i) Structural characterization of amorphous IrRu nanosheets prepared by the salt-assisted annealing method. Reproduced with permission from ref (145). Copyright 2019 Springer Nature under CC-BY license (https://creativecommons.org/licenses/by/4.0/).

In the above-mentioned bimetallic nanoparticulate materials, they are crystalline structures with long-range ordering of the metal atoms, although the occupation of a specific position in the crystalline structure could be either of the two metal elements as the scenario of alloy nanoparticles. Actually, bimetallic nanostructures can be formed in amorphous phase, in which the metal atoms do not show long-range ordering. For instance, the doping of B into crystalline PdRu nanoparticles will cause the crystalline-to-amorphous transition, which could be caused by the interruption of Pd–Ru bonding in the presence of B.^146^ To avoid the atomic ordering driven by thermodynamics, the synthesis of amorphous nanostructures is usually performed under mild conditions. As displayed in Figure 14, a general strategy for preparation of amorphous bimetallic nanosheets (thickness below 10 nm) by annealing mixture of metal acetylacetonates and alkali salt (KNO_3_ or KBr) at below 310 °C.^145^ In these structures, the metal–metal bonding is usually longer than that in the corresponding crystalline system.

##### 3.3.6 Bimetallic Nanoparticles by Overcoming Immiscibility

**Figure 15:**
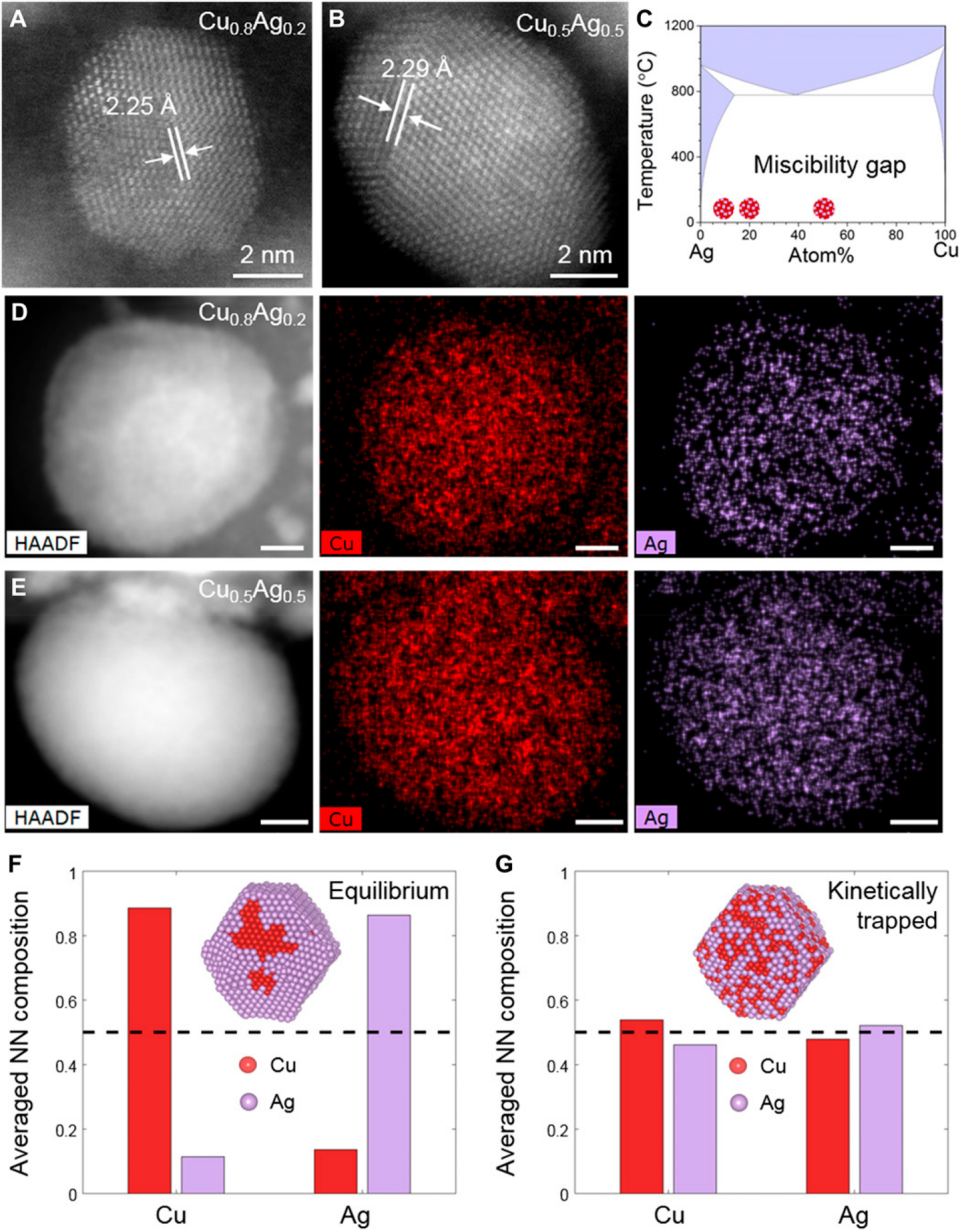
Alloyed Cu–Ag bimetallic nanoparticles with different compositions. High-resolution STEM image of typical (A) Cu_0.8_Ag_0.2_ and (B) Cu_0.5_Ag_0.5_ nanoparticles. (C) Bulk phase diagram of Cu–Ag, in which the Cu_1–*x*_Ag_*x*_ bimetallic nanoparticles in this work fall in the miscibility gap. HAADF-STEM images and EDS elemental mapping of (D) Cu_0.8_Ag_0.2_ and (E) Cu_0.5_Ag_0.5_ nanoparticles. Scale bars, 5 nm. Structure modeling of the Cu_0.5_Ag_0.5_ nanoparticle and the statistical analysis of the averaged nearest-neighbor (NN) composition surrounding the Cu and Ag atoms after MD/MC simulation at 25 °C, in which one MC trial step was attempted (F) every 1 fs to simulate sufficient diffusion for thermodynamic equilibrium and (G) every 10 ps to simulate limited diffusion and kinetic trapping. Reproduced with permission from ref (147). Copyright 2020 AAAS under CC-BY license (https://creativecommons.org/licenses/by/4.0/).

In a bulk bimetallic system, the composition of the bimetallic structures is dominated by the phase diagram. In nanoscale, the phase diagram still works, although it may differ from the conventional bulk phase diagram.^50^ To achieve the formation of thermodynamically metastable bimetallic structures, the mixing of the two metal elements need to be proceeded rapidly to avoid the segregation. For example, as shown in Figure 15, CuAg alloy nanoparticles are formed after heating the Cu and Ag precursors supported on carbon nanofibers to 1600 °C in less than 1 s via Joule heating, leading to the very fast decomposition of the precursors and the mixing of Cu and Ag atoms.^147^ Another approach to achieve the fast agglomeration of the metal atoms into metastable alloy nanoclusters/nanoclusters is using laser as the energy input source for creating local hot spots in the sample.^148^ For instance, the metal ions supported on graphene are reduced by laser propulsion and then the metal–metal bonding are formed, leading to the formation of multimetallic nanoparticles on the graphene support.^149^

It should be noted that the conventional phase diagrams are established under standard state conditions. By the introduction of metal–support interactions, it is possible to generate alloy nanoclusters/nanoparticles with compositions beyond the conventional phase diagram. In this regard, the use of solid carriers with rich defective sites can facilitate the capture and stabilization of metal atoms for overcoming immiscibility.

#### 3.4 Deactivation and Regeneration of Bimetallic Catalysts

Deactivation is a ubiquitous for solid catalysts, which is also commonly observed with bimetallic catalysts. Depending on the structural features of the bimetallic catalysts and the reaction conditions, the several types of deactivation mechanism may occur, such as the sintering of bimetallic particles, coking, leaching of metal species, segregation of the two metal elements, poisoning of the active sites, etc.^150−153^ Because of the complexity in deactivation mechanism and diversity in the structural features of bimetallic catalysts, the regeneration of deactivated bimetallic catalysts should be proceeded and optimized in a case-by-case fashion.^154^ For each particular case, the regeneration procedure of the deactivated catalyst needs to be carefully investigated based on the deactivation mechanism and the physicochemical properties of the bimetallic catalysts. In principle, the lessons learned in the studies of synthesis of various types of bimetallic catalysts and the structural transformation behaviors of bimetallic catalysts can be translated into the regeneration of the bimetallic catalysts. For instance, the regeneration of bimetallic PtSn nanoparticles supported on alumina can be achieved by proper oxychlorination treatment and subsequent high-temperature reduction treatment.^155,156^

#### 3.5 Summary

The ultimate goal of the synthesis of bimetallic entities (from binuclear species to nanoclusters and nanoparticles) is to control the geometric structure and composition of the metal species with atomic precision. This goal is achieved in several specific examples, such as the generation of binuclear bimetallic surfaces via surface organometallic approach and ligand-coordinated bimetallic nanoclusters. However, these as-synthesized well-defined species will undergo a structural transformation in catalytic reactions. Therefore, it is critical to maintain the initial structures of the as-synthesized well-defined species or to control the reaction-induced structural transformation, resulting in solid catalysts comprising uniform bimetallic species.

The presence of many different anchoring sites in the conventional solid carriers (high-surface-area oxides and carbon materials) hinders the precise control of the coordination environment of the bimetallic species. One promising approach is to utilize ordered porous materials such as zeolites and MOFs as the host for subnanometer bimetallic species. Although there are exciting progress in the last 10 years in this direction, the regioselective generation of highly uniform bimetallic metal species in the microporous environment is hindered by the difficulty of controlling the spatial distribution of the metal species inside the microporous crystallites and determining the location of the metal species with atomic precision. Considering that the use of an open-structure solid carrier is indispensable, the development of high-surface-area supports with uniform morphology, particle size, and porosity is also critical for the preparation of bimetallic catalysts with the well-defined metal–support interface.

Moreover, it is very difficult to achieve a good balance between structural uniformity and metal loading. In most scenarios, for the synthesis of subnanometer bimetallic catalysts, the metal loadings have to be kept below 0.5 wt % to avoid the presence of multiple types of metal entities, limiting the practical applications of the materials developed in the fundamental studies. It has been shown that the use of MOF as the host for isolated metal atoms or clusters can achieve metal loading up to 20 wt %.^157^ This approach relies on the use of a crystalline host with an open porous structure and controllable introduction of metal species into the porous framework and adequate postsynthesis treatments.

Fundamental understanding on the growth mechanism is vital for rational design of synthesis methodologies of bimetallic entities. Currently, there are already systematic studies on the formation and growth mechanism of monometallic and bimetallic nanoparticles, while the fundamental insights into the formation and growth mechanism of binuclear bimetallic sites and bimetallic nanoclusters on solid carriers are quite limited in the literature to date.^158,159^ In the future, we anticipate that more efforts dedicated to the mechanistic studies of the formation and evolution of supported subnanometer bimetallic entities will contribute to the rational synthesis of bimetallic catalysts based on binuclear sites and bimetallic nanoclusters.

## 4 Characterization of Bimetallic Sites

In this section, we will discuss the tools/techniques for characterizing geometric and electronic structures of bimetallic species from binuclear sites to bimetallic nanoclusters and nanoparticles. Especially, for each tool/technique, we will analyze the applicability to different types of bimetallic entities and discuss their advantages and limitations.

### 4.1 Characterization of Morphology

The morphology is a key structural feature that should be carefully studied if one attempts to establish the structure–reactivity relationship of a bimetallic entity. The morphological properties of a bimetallic entity include the size, geometric shape, and metal–support interfacial structure. The most frequently used technique for measuring the morphology of bimetallic species is aberration-corrected scanning transmission electron microscopy (AC-STEM). By working in the high-angle annular dark-field (HAADF) mode, the bimetallic species can be easily distinguished from the support with low *Z*-contrast (such as alumina and carbon materials). Moreover, if the difference in the atomic numbers of the two metal elements is large enough (for instance, Pt and Ru), it is possible to differentiate the binuclear species according to their contrast difference.^66^ By extracting the contrast and location of the metal atoms from the high-resolution HAADF-STEM images, one can determine the abundance of the binuclear bimetallic sites in the whole sample, based on an automated statistical analysis of a large number of images.^160^ In the case of bimetallic nanoclusters, it is also possible to resolve the three-dimensional structure of small clusters (usually with less than 20 atoms) by quantitative analysis of the AC-STEM images, although it relies on complicated instrumental setup and image analysis.^161^

Compared to subnanometer bimetallic species, it is easier to describe the general morphology of bimetallic nanoparticles with nanometric precision. Features such as particle size, exposed facet, distribution of the two metal elements, and particle–support interface can be readily measured by current electron microscopy techniques. However, if one needs to know further detailed information with atomic resolution, such as the three-dimensional structure of a bimetallic nanoparticle, it will be more challenging than the case with a bimetallic nanocluster.^18,162^ The recently developed atomic electron tomography technique can resolve the three-dimensional structure of a PtFe nanoparticle, although its realization requires a complicated experimental setup and image processing.^163^

**Figure 16:**
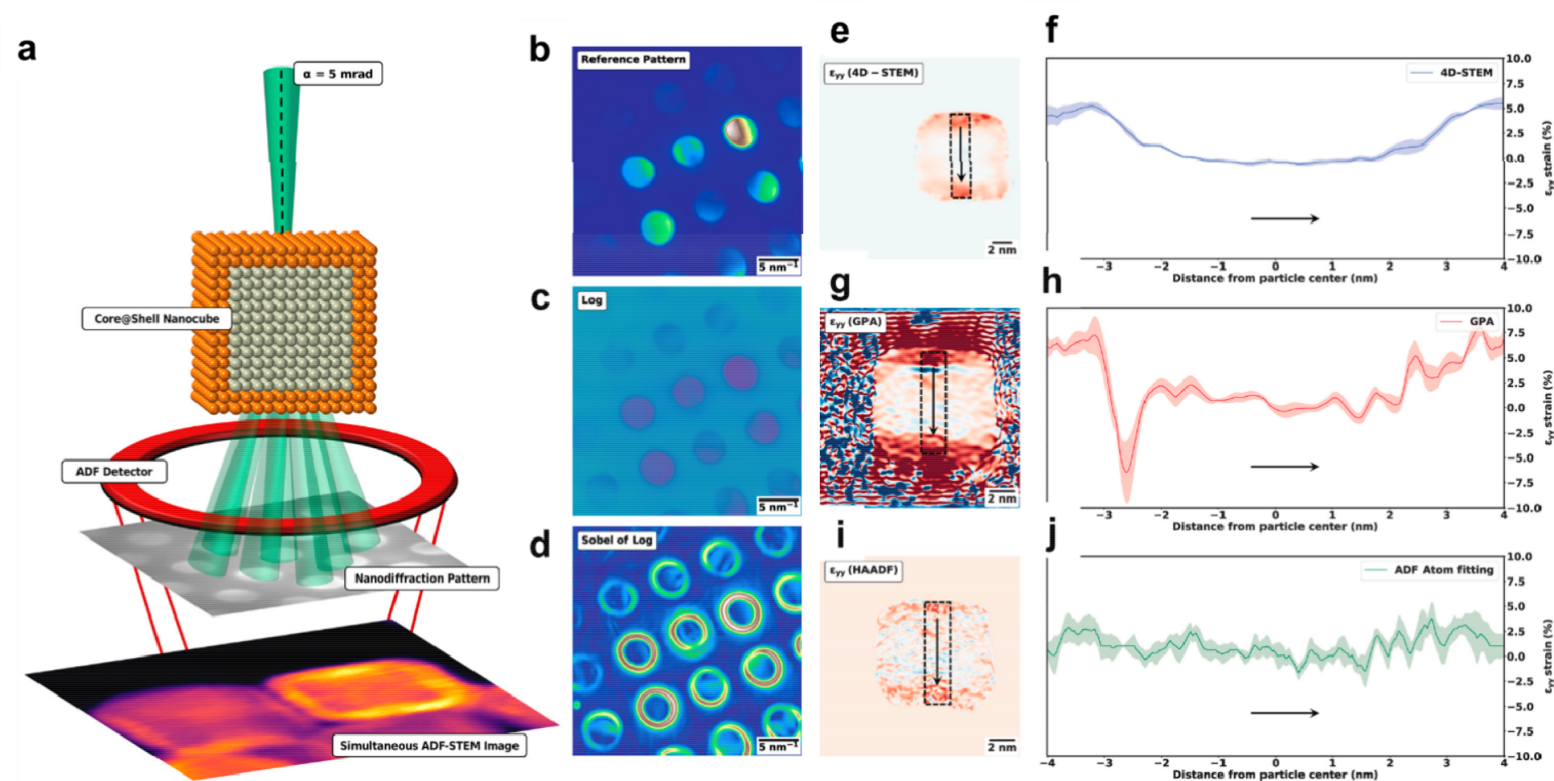
4D-STEM experimental setup and data preconditioning for measurement of the local strain in Rh@Pt nanoparticles. (a) Schematic of the experimental setup with an electron probe semiangle (α) of 5 mrad to avoid the overlapping of the diffraction disks. An ADF detector present above the 4D-STEM camera captures an ADF image. (b) Raw reference CBED pattern without preconditioning. (c) The logarithm of the diffraction pattern in (b). (d) The magnitude of the Sobel filtered image of the logarithm of the diffraction pattern is shown in (c). Comparison of measured strain through multiple techniques. (e) ϵ_*yy*_ strain measured through 4D-STEM disk fitting. (f) Variation of ϵ_*yy*_ strain in the region marked by the black dashed rectangle in (e) along the arrow direction. (g) ϵ_*yy*_ strain measured through the classic geometric phase analysis. (h) Variation of ϵ_*yy*_ strain in the region marked by the black dashed rectangle in (g) along the arrow direction. (i) ϵ_*yy*_ strain measured through two-dimensional Gaussian fitting of individual atom columns in a HAADF-STEM image. (j) Variation of ϵ_*yy*_ strain in the region marked by the black dashed rectangle in (i) along the arrow direction. Reproduced with permission from ref (164). Copyright 2020 American Chemical Society.

As mentioned before in this review, due to the mismatch in lattice parameters, lattice strain will be introduced into the bimetallic particles with core–shell structures. Ideally, the lattice strain in the core–shell bimetallic nanoparticles can be measured by X-ray diffraction because the lattice parameters can be directly calculated from the diffraction pattern.^165^ However, this technique requires a sample with high structural homogeneity, which is usually challenging to be achieved with bimetallic nanoparticles prepared by wet-chemistry methods. The local lattice strain can be calculated through the geometric phase analysis based on the atomic-level structural features extracted from high-resolution HAADF-STEM images, although errors may be introduced by image aberrations.^166^ As shown in Figure 16, The use of 4D-STEM can greatly improve the accuracy of strain analysis of core–shell nanoparticles because it allows quantification of the unit cell size as a function of distance from the core–shell interface due to the higher precision and absence of scan distortions that are afforded by 4D-STEM in comparison to aberration-corrected atomic-resolution STEM imaging technique.^164^ As an emerging electron microscopy technique, 4D-STEM can also be used to probe the electronic interaction between the metal particles and the support. For instance, the charge transfer from the SrTiO_3_ (STO) support to Au nanoparticle is directly visualized, which is supported by DFT calculations.^167^ This technique can be extended to the characterization of bimetallic nanoparticles supported on solid carriers, which may provide insights on the charge redistribution in these materials.

It is always argued by the researchers in catalysis community that high-resolution TEM/STEM images cannot reflect the averaged information on the measured samples because the images can only provide information in very local areas. This argument is valid when the sample comprises a mixture of different types of metal entities with a broad range of particle size distribution and chemical composition. However, if the metal entities in the sample exhibit high uniformity and a large number of representative images are collected, statistical information regarding the structural features of the metal entities can be obtained by performing automated analysis of the experimental images.^168^ The recent progress in employing machine-learning methods for processing TEM/STEM images has greatly improved the efficiency and precision for analysis of the experimental images, leading to the revealing of detailed structural features in the samples.

### 4.2 Characterization of Chemical Components

If the material containing bimetallic nanocluster is highly uniform, the chemical components of bimetallic clusters can be easily determined by conventional analysis such as inductively coupled plasma (ICP). Besides, due to their small sizes, the whole composition of the bimetallic clusters can also be measured by X-ray photoelectron spectroscopy (XPS), which cannot be achieved with large bimetallic nanoparticles (>5 nm) because of the limited penetration of X-ray. However, both XPS and ICP can only give an average value on the whole sample while they cannot provide the spatial distribution of the two metal elements in the solid catalyst in nanoscale or subnanometer regime.

**Figure 17:**
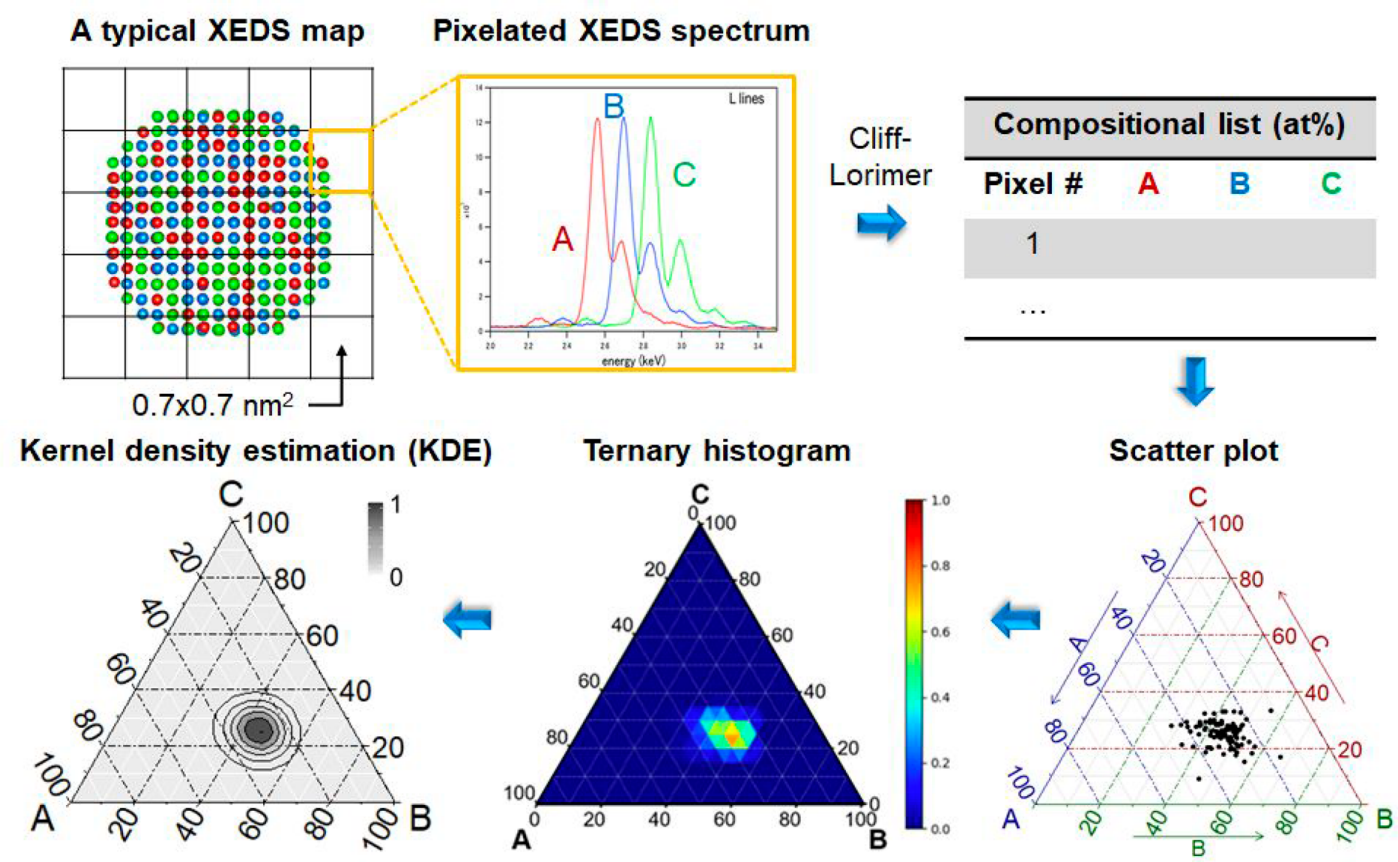
Schematic workflow for the statistical representation of local compositional distribution in arbitrary A–B–C ternary nanoalloys from an experimental two-dimensional X-ray EDS map. Reproduced with permission from ref (169). Copyright 2021 American Chemical Society.

As complementary tool to ICP and XPS, electron energy loss spectroscopy (EELS) and energy dispersion spectroscopy (EDS) can directly measure the chemical composition of a bimetallic entity. By measuring multiple bimetallic entities, one can give a preliminary conclusion on the uniformity/homogeneity of the distribution of the two metal elements, which cannot be achieved by ICP or XPS. As described in Figure 17, by grouping the raw EDS mapping data into two-dimensional data sets, the statistical representation of local compositional distribution in bimetallic or even multimetallic nanoparticles can be obtained, which can tell the homogeneity of the compositions with spatial resolution of ∼1 nm.^169^ Nevertheless, this workflow can be automatically applied to numerous EDS maps, leading to a statistical understanding on the composition of the bimetallic and multimetallic nanoparticles.

Furthermore, the three-dimensional composition analysis based on EELS and EDS tomography can provide three-dimensional structural information on bimetallic nanoparticles. With high-quality spectroscopic data, it is possible to reconstruct the spatial distribution of a bimetallic nanoparticle with spatial resolution less than 1 nm.^170^ The recent developments of image analysis methods and algorithms based on machine learning can greatly improves the precision of the 3D reconstruction with less input images than the conventional methodology, which will be very useful for characterization of beam-sensitive materials.^171^

In the case of bimetallic nanoclusters, the direct analysis of their chemical components by EELS/EDS is quite challenging because of the low signal/noise ratio of a single nanocluster. One indirect approach to resolve the structure of a bimetallic nanocluster is to perform detailed image analysis on high-quality AC-STEM image, as demonstrated with the K-means analysis with PtSn nanoclusters.^172^ By correlating the experimental and simulated HAADF-STEM images, it is possible to distinguish the Pt and Sn species and therefore to give the spatial distribution of the two elements in the zeolite support. In principle, this method can be a general approach for analyzing the structural features of supported bimetallic nanoclusters, especially when the spatial resolutions of the experimental HAADF-STEM images of challenging samples are compromised by the experimental conditions. It should be noted that, the reliability of this approach depends on the quality of the image simulation process, which should be as close to the experimental samples as possible.^173^

**Figure 18:**
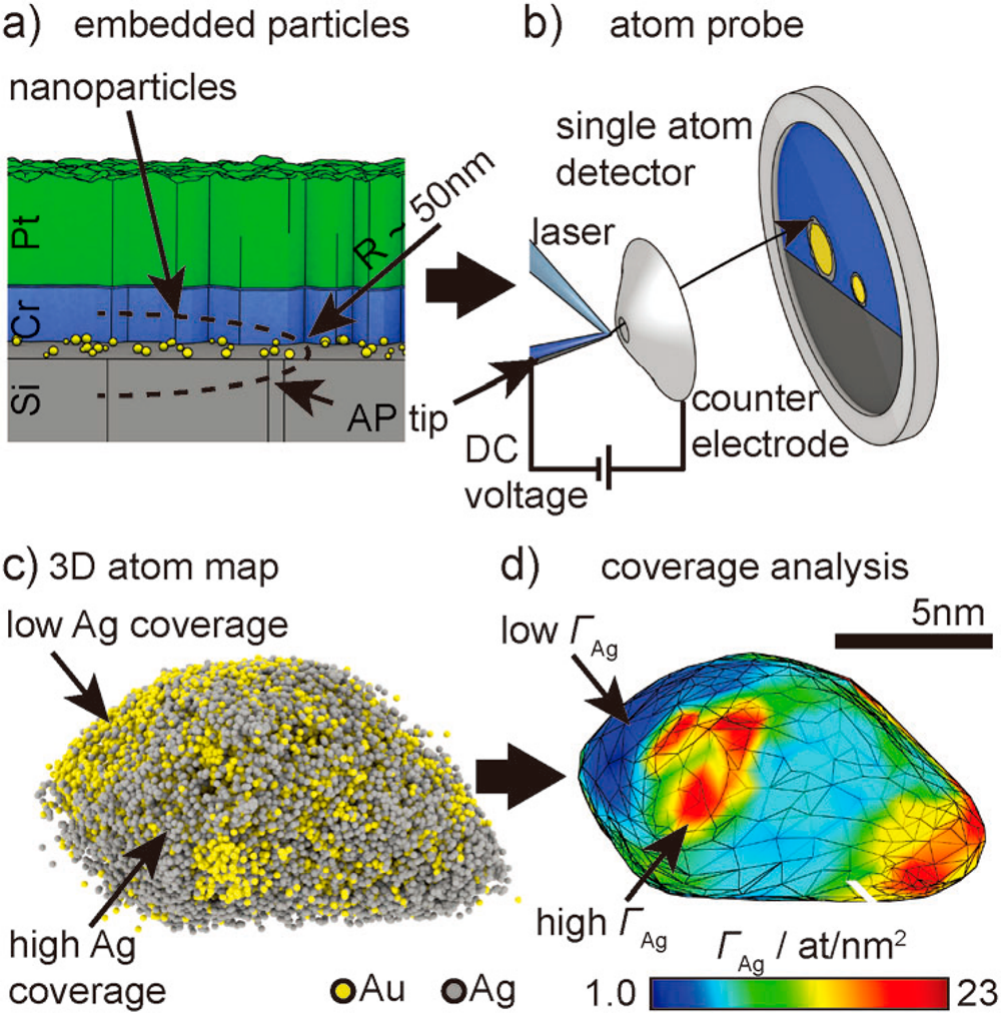
Characterization of the composition of bimetallic nanoparticles by atom probe tomography. (a) Schematic illustration of the preparation of the specimen. The nanoparticles are incorporated in between the Si and Cr layers (see text for details). (b) Set-up of a laser atom-probe experiment. (c) 3D view of an Au@Ag nanoparticle after sequential reconstruction from 2D detector coordinates and (d) quantification of the surface coverage (number of Ag atoms per nm^2^). Reproduced with permission from ref (175). Copyright 2014 Wiley-VCH.

The atom-probe tomography (APT) technique has been developed to achieve a high spatial resolution in regarding the composition of bimetallic nanoparticles in three-dimensional space.^174^ For instance, as can be seen in Figure 18, the location of Ag and Au atoms in core–shell AuAg nanoparticles can be directly resolved, giving a quantitative evaluation of the exposed surface atoms in bimetallic nanoparticles.^175^ After continuous improvements, the spatial resolution of APT can be achieved at the level of ∼0.5 nm or even smaller scale, which also depends on the physicochemical properties of the materials. Although the works on employing APT for structural characterization of supported metal catalysts are reported more than 10 years ago, this technique is still not widely applied in catalysis community, which could be caused by the requirements in professional experiences in sample preparation and data analysis.^176^ We believe that the commercial APT instruments will become more and more user-friendly, and therefore this technique can be employed by the catalysis community for a broad scope of catalytic materials because it can provide indispensable information on the three-dimensional structures of the solid catalysts.

### 4.3 Characterization of Electronic Properties

X-ray photoelectron spectroscopy is the most frequently used technique for probing the chemical states and electronic properties of supported metal catalysts.^177^ In the case of bimetallic entities, the chemical states of each metal elements and the potential charge transfer between the metals and the metal–support interaction can be revealed by XPS.^178^ The developments of ambient-pressure XPS based on synchrotron light source allow to follow the evolution of the chemical states of metal elements in bimetallic nanoclusters and nanoparticles as well as the structural transformation driven by the reactants.^179^

Besides XPS, the electronic properties of bimetallic entities are critical features because the interaction between the metal entities with the reactant molecules will be greatly determined by the electronic properties. As practiced with molecular complexes, in principle, the electronic properties of binuclear metal sites should also be able to be measured by UV–vis spectroscopy. However, due to the low metal loadings and the interfere from the support, the spectroscopic features of the binuclear metal sites may be difficult to be captured.

In the case of bimetallic nanoclusters with well-defined structures prepared by wet-chemistry synthesis, UV–vis spectroscopy is a powerful and facile tool to elucidate their electronic properties. The doping of one atom into a metal cluster can be clearly differentiate from the monometallic cluster because of the marked changes in the UV–vis spectrum, as proved with the study of bimetallic M-doped Au clusters (M = Pt, Pd).^88^ By employing gas-phase photoelectron spectroscopy, the electronic structures of bimetallic nanoclusters can be revealed with details of the orbital information, providing insights to understand the molecular-like properties of the nanoclusters with precise structures.^180^ Moreover, the changes in electronic structures of bimetallic clusters induced by adsorption of reactants can be captured by in situ UV–vis spectroscopy.^181^ The unique electronic properties of metal clusters are also reflected in their fluorescence spectra. Bimetallic nanoclusters can be usually easily differentiated from the monometallic counterparts by fluorescence spectroscopy.^182^ The electron affinities of the ligand-protected bimetallic nanoclusters can be measured by gas-phase photoelectron spectroscopy, which gives more precise information on the electronic structures of the metal clusters.^183^

The electronic properties of bimetallic nanoparticles are also reflected in the UV–vis spectra, although it is not straightforward to correlate the spectra with their electronic structures because of the broad bands in the spectra. Due to their plasmonic properties, bimetallic nanoparticles comprising Au, Ag, or Cu show size-dependent absorption band in UV–vis spectra. The variation of particle size and chemical components will be reflected in the UV–vis spectra.^184^

### 4.4 Characterization of Surface Properties

From a structural point of view, for metal nanoclusters of sizes around 1 nm, almost all the metal atoms can be considered as surface atoms, although capping agents or ligands may coordinate to the metal atoms (at least part of them, if not all), resulting in the separation of those atoms from the external environment. Probing the surface properties of the exposed metal atoms of the bimetallic clusters can help to understand the amount of exposed surface sites and their chemical reactivity. One commonly used approach is to employ a probe molecule to interact with the metal nanoclusters and then to measure the impacts of metal–molecule interaction by chemisorption, vibrational, and/or electronic spectroscopy.

**Figure 19:**
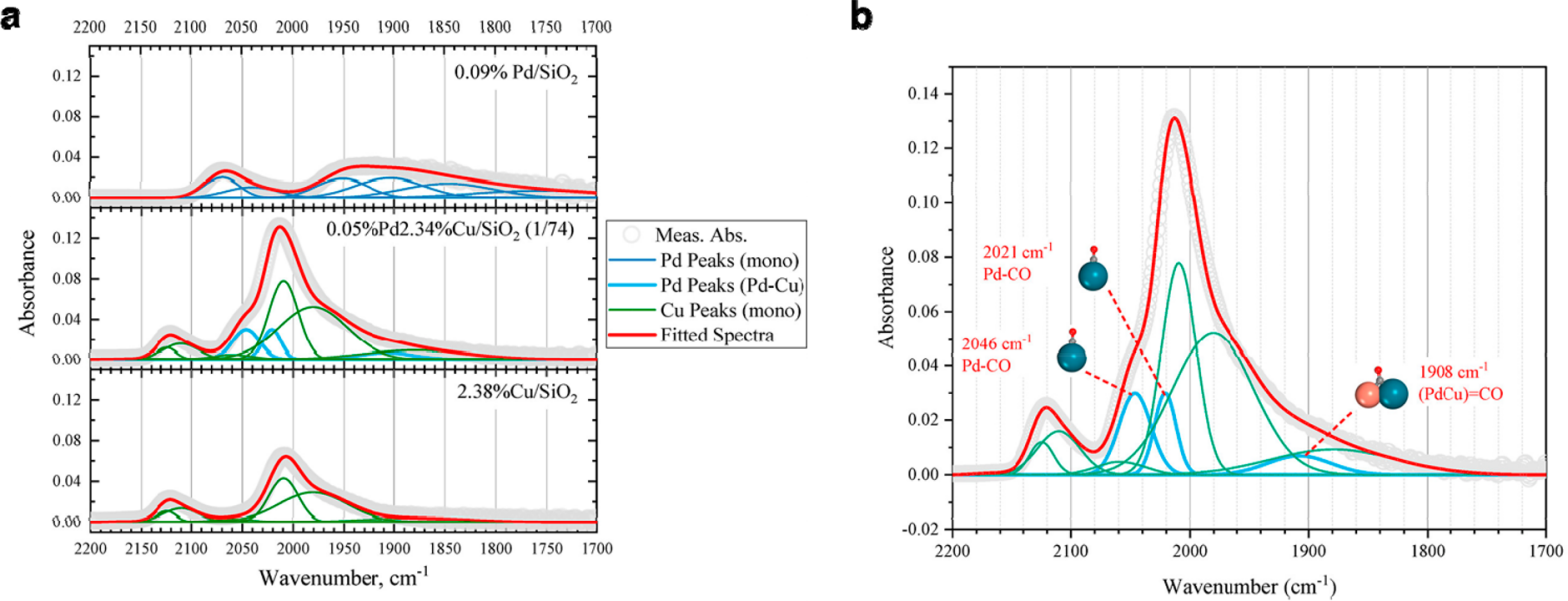
(a) Fitted spectra (final N_2_ purging) for monometallic Pd and Cu and bimetallic Pd–Cu systems and (b) three additional peak assignments for isolated palladium atom sites on diluted Pd–Cu single-atom alloy. Reproduced with permission from ref (185). Copyright 2022 American Chemical Society.

For instance, using CO or H_2_ as a probe molecule, the number of the exposed surface sites can be measured reliably by chemisorption methods, which actually has already been widely practiced with conventional nanoparticulate metal catalysts. By employing CO as probe molecule, the surface composition of bimetallic entities can be quantitative measured when only one metal can absorb CO while the other cannot. For example, the number of surface Pd sites in the dilute PdAu bimetallic nanoparticles is determined by quantified pulses of CO by assuming all the Pd sites are equal for adsorption of CO.^186^ However, it is inferred by a comparative study that multiple types of adsorption configurations could exist in the dilute single-atom PdCu alloy nanoparticles, which gives different CO/Pd ratios in the adsorption models (see Figure 19).^185^ Therefore, the precise measurements of the metal sites in bimetallic nanoparticles relies on the identification of the adsorption configuration (mono- or dicarbonyl configuration) of the probe molecule at the measurement conditions (temperature, partial pressure of the probe molecule etc.) by performing rigorous control experiments on the monometallic samples.^187^

It should be noted that due to the low stability of metal nanoclusters, the activation conditions used before the chemisorption measurements should be carefully selected to avoid sintering or other types of structural transformation of the nanoclusters. Nevertheless, when the probe molecules interact with binuclear bimetallic sites and bimetallic nanoclusters, the adsorption configuration of the probe molecules could be different to the classic metal nanoparticles. For example, CO may be adsorbed on small metal clusters via the bridged configuration instead of the terminal configuration. In the case of binuclear bimetallic sites, CO or H_2_ may not be adsorbed on those sites. Consequently, the well-established method may not be able to give quantitative results when applying the chemisorption measurement to subnanometer bimetallic sites because of the nonstoichiometric adsorption pattern, although the qualitative results can also provide useful insights. Nevertheless, the CO-induced reconstruction and/or segregation of bimetallic nanoclusters/nanoparticles should not be neglected, as a result of the different affinity of the metals to CO in the bimetallic entities.^188^ For this reason, it is recommended to collect the CO-IR spectra at different CO coverages at various temperature to evaluate the influences of the reconstruction of the bimetallic entities.

CO-infrared (CO-IR) spectroscopy is employed to acquire further information about the surface structure of the bimetallic nanoclusters, which gives detailed information on the electronic properties and coordination environment. The shape and position of the CO adsorption bands can tell the information regarding the surface composition of the bimetallic nanoclusters and the distribution of the two metal elements.^189^ Taking PtSn nanoparticles as an example, the introduction of Sn species to Pt nanoparticles will weaken the adsorption of CO on the Pt sites, as reflected on the decrease of chemisorption capacity of CO. Nevertheless, the full width at half-maximum of the linear configuration of CO adsorbed on PtSn nanoparticles are usually narrower than that on Pt because of the preferential coverage of Sn on the highly undercoordinated Pt sites.^190^ Therefore, the combination of CO-IR and XPS is quite useful for elucidating the spatial distribution of the two metal elements in bimetallic nanoclusters and nanoparticles.

### 4.5 Characterization of Coordination Environment

**Figure 20:**
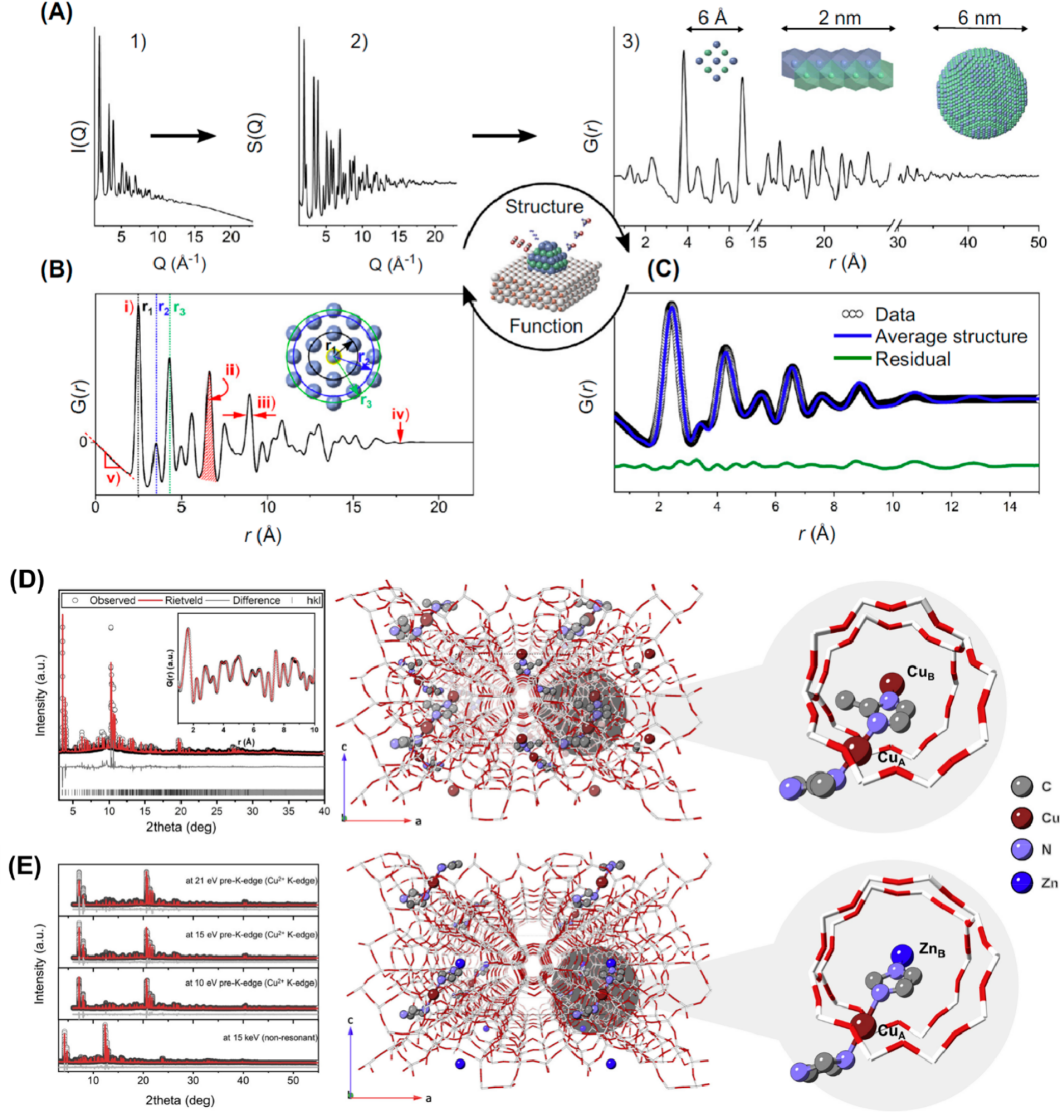
Characterization of supported metal catalysts by Pair distribution function (PDF) and synchrotron-based X-ray diffraction. (A) Schematic procedure to obtain a PDF from total scattering data and the different ranges of structural order covered by PDF for the example of a nanoparticle. (B) Key features of a PDF spectrum and the structural information contained: (i) peak positions correspond to interatomic distances, (ii) peak areas are proportional to coordination numbers, (iii) peak widths relate to static/dynamic disorder, (iv) the maximum *r* above which there are no more PDF peaks is an estimate of the ordered domain size, and (v) the initial slope of the PDF is related to the atomic density. (C) Exemplary PDF fitting to resolve local disorder as the mismatch between the experimental data (black curve) and an average structure model (blue curve). (D–E) Atomic and structural elucidation of monometallic and bimetallic CuZn species in ZSM-5 by PDF. (D) Rietveld refinement of SXRD data and the refined crystal structures of Cu_2_ species in ZSM-5 (inset: X-ray pair distribution function analysis profile). (E) Resonant SXRD measurements of Cu-Zn-ZSM-5 sample. Atoms are represented in balls and sticks; white and red sticks represent Al/Si and O, respectively. The refined crystal structures have been verified by DFT calculations. (A–C) Reproduced with permission from ref (191). Copyright 2022 Elsevier Inc. (D–E) Reproduced with permission from ref (192). Copyright 2022 Elsevier Inc. under CC-BY license (https://creativecommons.org/licenses/by/4.0/).

The coordination environment of a metal entity includes the bonding between the metal atoms inside the metal entity and the bonding with the external environment (solid carrier, support, and reactant). The atomic structures of bimetallic nanoclusters can be resolved by single-crystal X-ray diffraction, as practiced with well-defined bimetallic nanoclusters.^194^ However, this approach relies on the formation of single crystals for collection of high-quality X-ray diffraction patterns, which is usually not feasible with practical heterogeneous metal catalysts. To improve the data quality of X-ray diffraction, the powder samples can be measured with synchrotron X-ray sources which give high brightness and high resolution. As shown in Figure 20, the position of binuclear Cu–Zn species confined in ZSM-5 crystallites and the organic ligands between the Cu and Zn ions has been resolved by Rietveld refinement of synchrotron X-ray diffraction.^192^ Moreover, pair distribution function (PDF) analysis is a powerful tool to elucidate the atomic structures of the solid materials at multiple length scales (from a few angstroms to several nanometers), which is based on the total scattering analysis of the long-range order measured by Bragg peaks in XRD patterns and the short-range order in the less well-defined features in the diffraction patterns.^195^ In this regard, PDF is suitable for resolving the distribution of the two metal elements and their bonding information in bimetallic nanoclusters and nanoparticles.^196^

X-ray absorption spectroscopy (XAS) is the most commonly used technique for charactering the coordination environment of the metal species in solid catalysts without long-range crystalline structures, which has been widely applied for elaborating the bonding information on isolated metal atoms and metal clusters on solid carriers.^197−199^ In the case of subnanometer bimetallic metal entities (binuclear sites and bimetallic clusters), both the metal–metal bonding and the metal–environment bonding can be captured by XAS based on data of fairly good quality.^200^ However, in some materials (e.g., low metal loading, low uniformity), the data interpretation will be not straightforward and only qualitative speculation can be obtained, due to the low sensitivity of XAS to the disorder structures of subnanometer bimetallic species.^201^ In these cases, the combination of multiple characterization techniques will be helpful to study the local structures of the subnanometer bimetallic species.

In principle, the coordination environment of the metal atoms in bimetallic nanoparticles can also be clarified with XAS technique, although it will be challenging to obtain as detailed information as the cases of subnanometer bimetallic species, due to the more complicated structures of bimetallic nanoparticles.^202^ As a bulk technique, EXAFS can also be a surface-sensitive technique for characterization of metal nanoparticles by careful experimental design. For instance, by subtracting the EXAFS spectra of the reduced Pt nanoparticles and the same sample after exposure to air can give the difference, which could be associated with the oxidized Pt species on the surface.^203^

**Figure 21:**
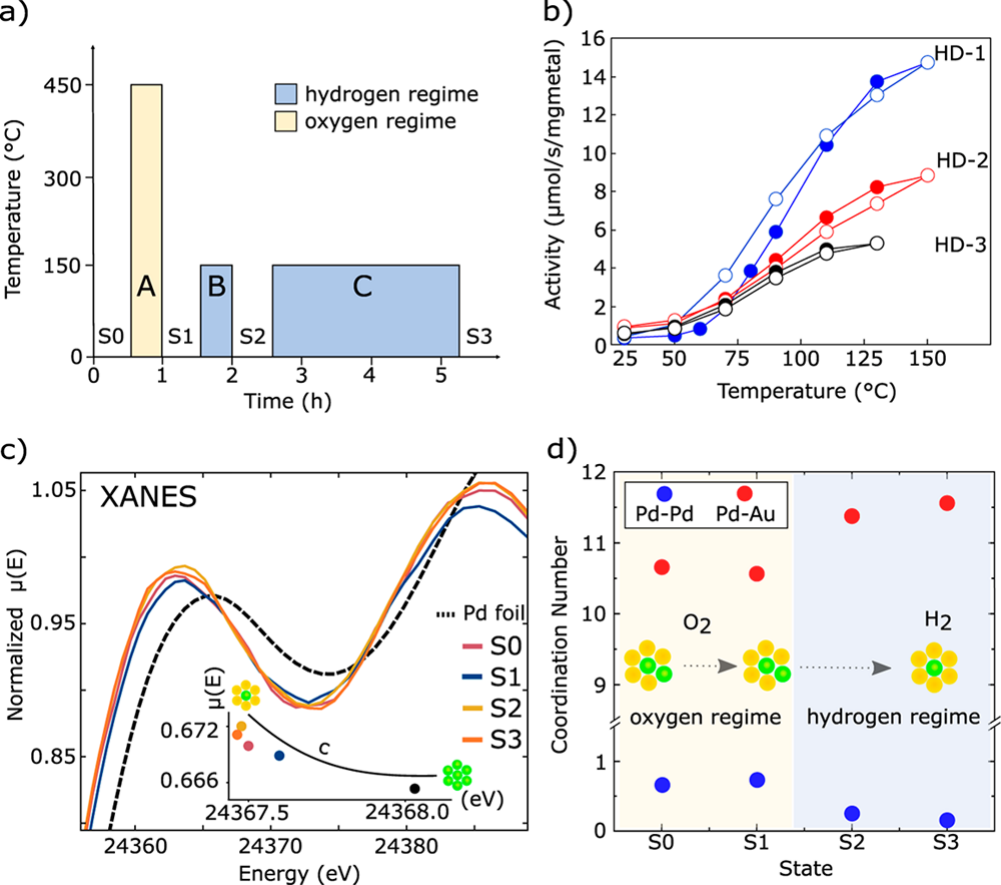
Evolution of the distribution of Pd and Au in PdAu alloy nanoparticles. (a) Initial state S0 undergoes O_2_ treatment to starting state S1 (treatment A), followed by H_2_ treatments to starting states S2 and then S3 (treatments B and C, respectively). (b) HD exchange experiments, labeled HD-1 to HD-3, corresponding to three different starting states (S1–S3). The solid and open circles indicate the heating and cooling regimes, respectively. (c) Normalized XANES spectra collected for the different samples (pink = S0, blue = S1, yellow = S2, orange = S3, and dashed black = Pd foil). The inset shows a shift in the spectral center of mass “*c*”. (d) Coordination numbers (blue = Pd–Pd bonds and red = Pd–Au bonds) from neural network-XANES are consistent with Pd atoms having more Pd neighbors after O_2_ treatment (yellow shade) and fewer Pd neighbors after H_2_ treatment (blue shade). Atomic Pd ensembles in (c) and (d) are depicted with bird’s-eye-view illustrations (yellow = Au; green = Pd). Reproduced with permission from ref (193). Copyright 2022 Springer Nature under CC-BY license (https://creativecommons.org/licenses/by/4.0/).

By applying supervised machine learning methods to the analysis of the XAS data (including both the X-ray absorption near edge structure (XANES) and extended X-ray absorption fine structure (EXAFS)) and theoretical modeling, it is possible to elaborate the distribution of the metal elements in bimetallic nanoparticles.^204,205^ The progress in this direction is very useful for elucidating the coordination environment of the bimetallic particles. As shown in Figure 21, after consecutive oxidation and reduction treatments, the Pd_2_ dimers in the PdAu alloy nanoparticles (Pd/Au = 8/92) tend to become separated due to the rearrangement of the spatial distribution of Pd and Au atoms in the alloy nanoparticles, as inferred by the analysis of the XANES and EXAFS spectra based on the neural network-assisted XANES inversion method.^193^ Thanks to the advanced data analysis method, the subtle structural changes can be captured and analyzed, leading to new insights on the coordination environment of the metal species and the metal–metal bonding (average coordination number and metal–metal bond length) in the alloy nanoparticles.^206^

A critical issue related to the artifacts introduced in the data fitting procedure, especially for the spectra with relatively low signal-to-noise ratios. To improve the accuracy of data interpretation in analysis of EXAFS spectra, it is proposed to employ the models derived from theoretical calculations for fitting the experimental EXAFS spectra. The theoretical models can provide insights for the possible bonding information in the sample and give useful hits for setting up the fitting parameters. This suggestion is practiced with analysis of the EXAFS spectra of supported isolated metal atoms and should be employed for measurements of supported bimetallic entities, from binuclear bimetallic species to bimetallic nanoclusters and nanoparticles.^207,208^

Another important issue associated with the characterizations of supported subnanometer metal catalysts by synchrotron-based X-ray techniques is the stability of the tiny metal species under high-flux X-ray irradiation, which is already well-documented for metal complexes grafted on solid carriers.^209^ It is found that the reduction of Rh species supported on Al_2_O_3_ is greatly enhanced by high-flux X-ray, resulting in the agglomeration of Rh species.^210^ Taking into account that long-time measurements are frequently required for supported subnanometer metal catalysts for accumulating enough signal, the beam-induced damage to the sample and the associated effects should be considered and mitigated. When performing in situ XAS measurements with supported metal catalysts, leaching of metal species from the support to the solvent/electrolyte could occur due to the high X-ray flux. In this sense, the flux of X-ray for liquid-phase reactions should be even lower than those used in gas-phase reactions to avoid the potential catalyst loss through dissolution.^211^

Beside the bonding information between the metal elements, the metal–ligand and metal–support bonding information is also critical, which can be acquired by solid-state NMR.^212^ For instance, by collecting the ^195^Pt NMR spectra of the bimetallic PtM (M = Pd, Sn) nanoparticles and a monometallic Pt reference sample, it is possible to clarify whether the Pt and the other element is well mixed in the bimetallic nanoparticles.^213,214^ After the introduction of sensitive spy nucleus (^1^H or ^31^P), it is possible to achieve the rapid acquisition of ^195^Pt magic angle spinning NMR spectra with good data quality. This new technique can characterize the coordination environment of organometallic Pt complex grafted on solid carrier and probe the metal species neighboring the Pt complex.^215^ Solid-state NMR can be considered as averaged information in the whole sample, while the TEM-based EDS mapping can only provide very local information on the spatial distribution of the two elements. Moreover, in terms of the bimetallic nanoclusters or nanoparticles with ligands on the surface, the bonding between the ligands and the metal atoms could be directly probed by ^13^C NMR spectra.

### 4.6 Characterizations of Surface Reactivity

Most of the above-mentioned techniques are used to probe the structural features of the bimetallic entities. However, there are still gaps between the structural features and catalytic behavior, which can be partly filled by the characterizations of surface reactivity. This approach is usually achieved by measurements based on temperature-programmed desorption (TPD) and temperature-programmed surface reaction (TPSR).^216^ For instance, the reactivities of bimetallic nanoclusters generated by physical approaches such as size-selection method are mostly measured by these techniques. For instance, the TPD data for methanol adsorbed on the surface of AuPt clusters supported on TiO_2_(110) surface can not only indicate the reactivity of the AuPt clusters, but also provide insights on the exposed surface sites of the bimetallic clusters according to the variation of the desorption temperature and the final products.^217^

**Figure 22:**
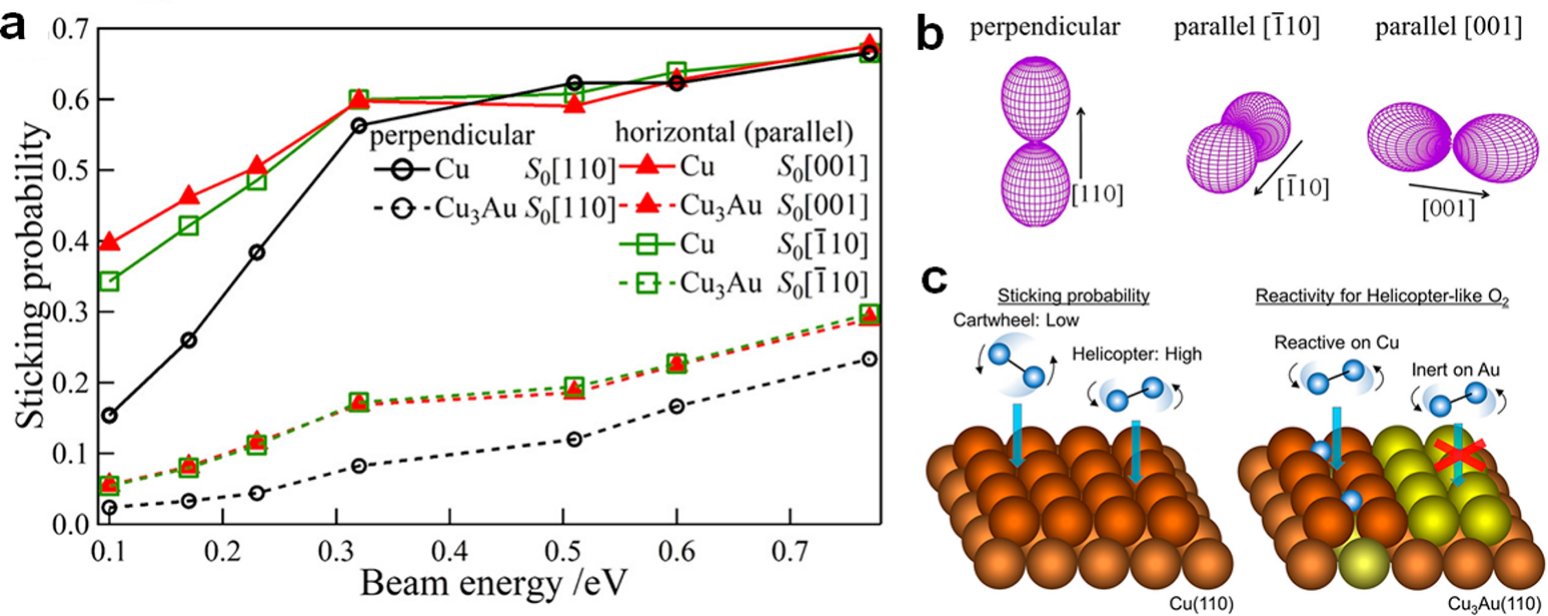
Probing copper and copper–gold alloy surfaces with space-quantized oxygen molecular beam. (a) Angular distributions of the O_2_ molecular axis (O–O bond axis) oriented perpendicular (along [110]) and parallel (along [1̅1̅0] and [001]) to the corresponding surfaces. (c) Initial sticking probability contributions from the O–O bond axis oriented perpendicular and parallel to Cu(110) and Cu_3_Au(110) as indicated in (b). (c) Illustration of the reactivity of O_2_ molecule on Cu(110) and Cu_3_Au(110) surface. Reproduced with permission from ref (218). Copyright 2022 American Chemical Society.

As can be seen in the abundant literature, measurements of surface reactivity of bimetallic catalysts are desirable to be performed on bimetallic model surface to give molecular- and atomic-level insights on the reaction mechanism and structures of the active sites. For instance, as displayed in Figure 22, using space-quantized oxygen molecular beam as reactant, the activation on Cu(110) and Cu_3_Au(110) alloy surface is measured, respectively, which indicates that O_2_ molecule with helicopter configuration can be activated on Cu(110) surface, while the O_2_ activation is prohibited on Cu_3_Au(110) alloy surface.^218^ Another typical example is the study of geometric structure of PdAu surface for acetoxylation of ethylene to vinyl acetate, which suggests that only the Pd atoms deposited on Au(100) surface are close enough for catalyzing the coupling reaction, while the Pd atoms on Au(111) surface are not active due to their large distance.^219,220^ The lessons accumulated in surface science studies can be translated to practical catalysts based on bimetallic nanoclusters/nanoparticles for fundamental understanding of the structure–reactivity relationships.

### 4.7 Dynamic Structural Evolution

**Figure 23:**
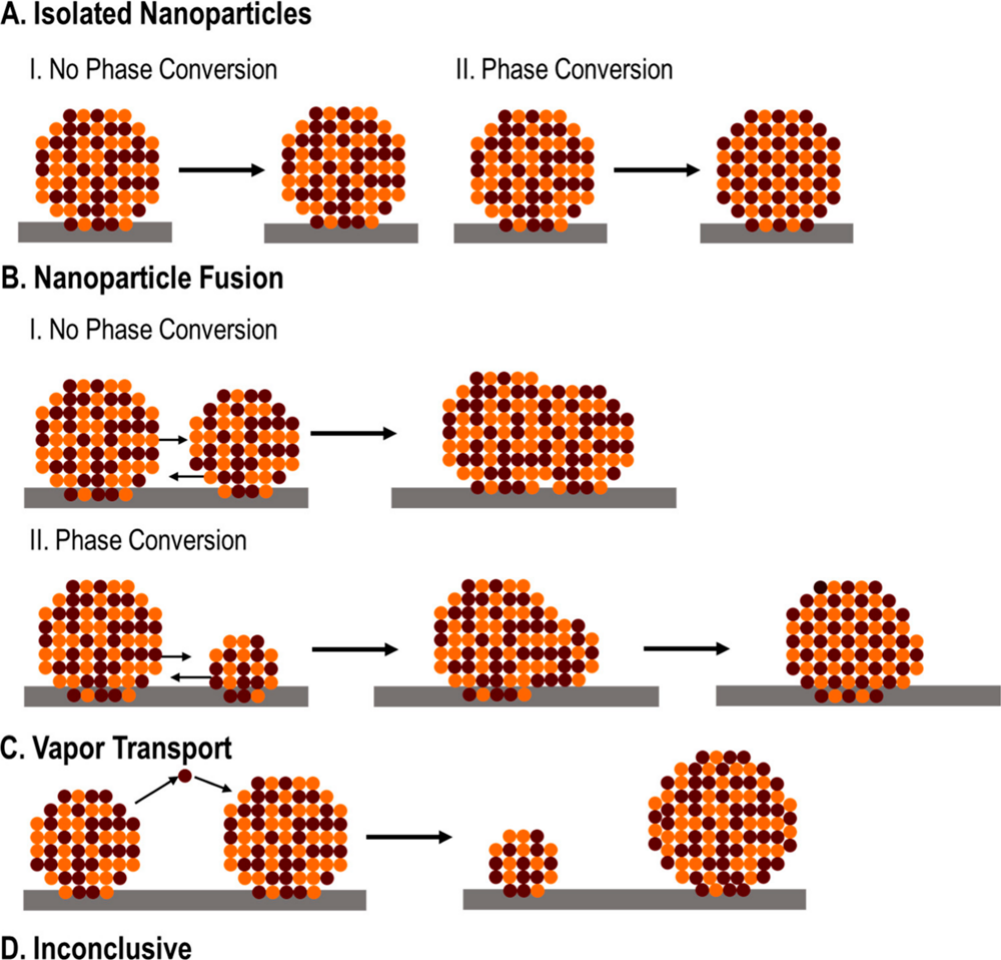
Schematic illustration of the evolution process of bimetallic nanoparticles observed during in situ annealing TEM experiments. (A) In scenario I, bimetallic nanoparticles remain stable during the annealing treatment. In scenario II, the bimetallic nanoparticles are transformed into ordered structures, which indicates the phase conversion of the bimetallic particles. (B) Sintering of bimetallic nanoparticles with and without phase conversion. (C) Migration of metal atoms from one particle to another, leading to the broadening of the particle size distribution. (D) Inconclusive situation with the above-mentioned structural evolution. Reproduced with permission from ref (221). Copyright 2021 American Chemical Society.

Heterogeneous catalytic reactions normally involve the dynamic structural transformation of the solid catalysts under reaction conditions, as widely observed in both surface model systems and practical catalysts based on particles.^222,223^ With advanced characterization techniques such as in situ TEM and in situ XPS, the dynamic structural evolution in bimetallic nanoparticles has already been revealed, including the rearrangement of the spatial distribution of the two elements, change of the exposed surface and interfacial structure, and change of chemical states and particle sizes.^224^ For instance, as revealed by in situ TEM, Ni@Au core–shell nanoparticles show reversible temperature-dependent structural transformation under CO_2_ hydrogenation conditions. Au shells are present at low reaction temperatures, while at high reaction temperatures, bimetallic AuNi alloy shells are formed due to the migration of Ni atoms into Au lattice.^225^ Beside the segregation under reductive atmosphere, oxidation-induced structural reconstruction is also widely observed in numerous systems. Specifically, the oxyphilic metals such as 3d metals have higher affinities than the noble metals such as Pt and Au, and therefore, upon exposure to oxidative atmosphere, surface segregation of oxyphilic metals will occur, resulting in the formation of oxide patches or layers on the surface of bimetallic nanoparticles. In some cases (see summary in Figure 23), the treatment-induced structural transformation is reversible, while in some cases, the transformations are irreversible, causing gradual segregation or sintering of the bimetallic entities, which can probably be associated with the variation of catalytic performances.^221,226^

**Figure 24:**
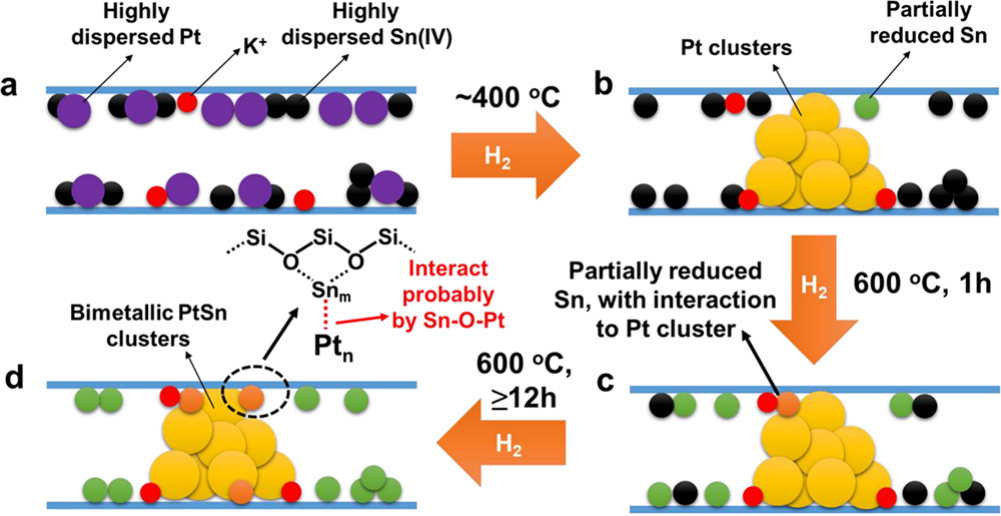
Structural evolution of K-PtSn@MFI during the reduction treatment with H_2_. (a) In the pristine K-PtSn@MFI sample, both Pt and Sn exist as atomically dispersed species in the sinusoidal channels of MFI zeolite. (b) After reduction by H_2_ at 400 °C, Pt single atoms will be reduced and form subnanometer Pt clusters, while only a few of the Sn(IV) species will be reduced. (c) When the temperature is increased to 600 °C, most of the Sn(IV) species are reduced to Sn(II), but those reduced Sn species mainly remain separated from Pt clusters. (d) After being kept in H_2_ flow for long time (≥12 h), part of the reduced Sn species migrate to Pt clusters and bimetallic PtSn clusters with reduced Sn species interacting with the external surface of Pt clusters are formed in the sinusoidal 10R channels of MFI zeolite. It should be noted that, due to the complexity of the structures of PtSn clusters, the models proposed in this figure could be oversimplified. PtSn clusters with different structures could exist in the working catalyst, as indicated by the CO-IR spectra. Reproduced with permission from ref (16). Copyright 2020 Springer Nature Limited.

Regarding binuclear species and bimetallic nanoclusters, their structure evolution behavior will be more elusive than nanoparticulate counterparts due to their smaller sizes.^227^ Indeed, limited by characterization techniques, there are few reported works on structural transformation of subnanometer bimetallic species. The prior works on structural evolution of subnanometer monometallic species already show that metal entities with smaller sizes will be more sensitive to the environment than the larger ones. It can be expected that when varying the reaction conditions, the coordination environment, morphology, and chemical states of the subnanometer bimetallic species will change accordingly.^228^ To capture these subtle structural changes, characterization techniques with high spatial and temporal resolution are needed to reveal the nature of the active sites under reaction conditions. For instance, as illustrated in Figure 24, the reduction and migration of Sn species along the microporous channels of MFI zeolite to interact with Pt clusters to form bimetallic PtSn clusters has been directly followed by quasi in situ TEM and CO-IR spectroscopy.^16,229^

The dynamic structural transformation could occur during the characterization procedure (e.g., the activation process of the sample or the high-vacuum environment inside the instrument), and the structural information obtained under those conditions may not be able to reflect the representative structures under reaction conditions. Specially, this point should be taken into account when dealing with bimetallic binuclear sites and nanoclusters, whose structures are quite flexible.

### 4.8 Summary and Suggestions

Each characterization technique has its advantage for measuring or detecting some featured properties of the bimetallic entities but also has its limitations in clarifying the detailed structures and distinguishing one type of entity from others. The low sensitivity of EXAFS to disorder metal species, especially the oxidized metal species, may lead to controversial conclusions in comparison with the characterization results obtained with high-resolution transmission electron microscopy.^230^ Therefore, it is highly recommended to employ various characterization techniques in a concerted way in order to obtain structural information on the bimetallic entities in a global view. Furthermore, by correlating the information derived from different techniques, it is helpful to avoid the pitfalls that maybe encountered in the process of characterizing binuclear species or bimetallic nanoclusters, because some techniques are established on nanoparticles but not directly applicable to subnanometer metal species.^230^

Resolving the full structure (i.e., precise characterization of the particle size, composition, and three-dimensional geometric structure) of supported bimetallic clusters is highly challenging. This goal has been achieved in quite a few examples by the combination of advanced electron microscopy (aberration-corrected electron microscopy) and spectroscopy (high-resolution energy dispersive spectroscopy and electron energy loss spectroscopy) techniques. However, these techniques can only give the three-dimensional structures of bimetallic clusters in a very local region, which may not well represent the whole sample. In our opinion, there are two prerequisites for the resolution of the full structure of bimetallic clusters in a supported bimetallic catalyst: (1) a sample comprising bimetallic clusters with highly uniform structural features (particle size, composition, etc.) and (2) a combination of advanced electron microscopy and spectroscopy techniques. By fulfilling the prerequisites, the structural information derived from the characterization can represent the properties of the supported bimetallic clusters and can be further used for establishing the structure–reactivity relationship.

## 5 Catalytic Applications of Binuclear Bimetallic Sites

The use of binuclear metal sites for catalysis has been studied by researchers in the field of inorganic and organometallic chemistry for a long time. In this section, we will briefly summarize the catalytic applications of binuclear bimetallic sites in natural enzymes and binuclear bimetallic complexes prepared by chemists and then discuss the development of supported binuclear bimetallic sites for various catalytic reactions.

### 5.1 Binuclear Bimetallic Sites for Hydrogenation Reactions

Hydrogenation reactions generally involve the activation of H_2_ and the transfer of the activated H species to the target molecule.^231^ In the natural [NiFe] hydrogenase, the neighboring Ni and Fe atoms will active H_2_ by forming a Ni···H···Fe intermediate and then reduce the substrate molecule.^232^ The idea of using binuclear bimetallic sites for hydrogenation reactions has also been practiced with organometallic complexes. For instance, a binuclear PtRh complex shows remarkable reactivity for the reduction of N_2_O to N_2_ with H_2_.^233^ The high reactivity of the binuclear PtRh complex relies on the synergy of Rh and Pt sites for activation of N_2_O and H_2_. More examples can be referred to some review articles in this field.^234^ A key lesson that can be learned from these enzymatic and homogeneous catalysts is that the dissociation of H_2_ is achieved by the involvement of two metal atoms, as proved by detailed mechanistic studies.

**Figure 25:**
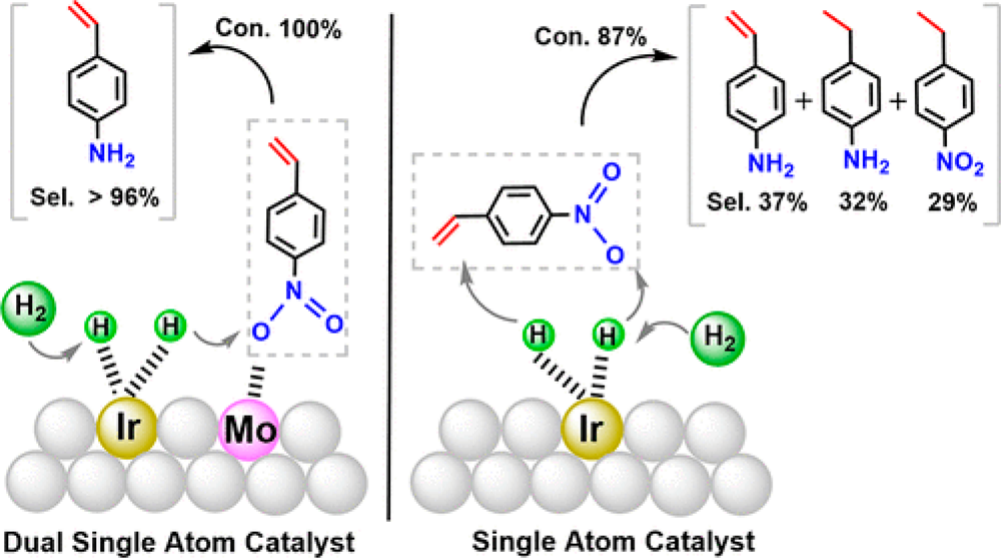
Binuclear bimetallic IrMo catalyst supported on TiO_2_ for chemoselective hydrogenation of 4-nitrostyrene. Reproduced with permission from ref (64). Copyright 2021 American Chemical Society.

By immobilizing metal complex precursors on solid carriers, binuclear bimetallic sites can be generated which can show enhanced stability than the enzymatic and homogeneous counterparts and then can be used for catalyzing hydrogenation reactions. For example, binuclear PtZn sites are formed after depositing organometallic Pt complex on Zn-modified silica by ALD and the resultant bimetallic sites show good activity for both selective hydrogenation of 1,3-butadiene and nitroaromatics.^68^ Besides, the promoting role of the metal atom to its neighboring atom has been demonstrated with a supported binuclear IrMo catalyst for selective hydrogenation of functional nitroaromatics.^64^ As shown in Figure 25, the Ir and Mo atom can serve as the active site for H_2_ activation and adsorption of the nitro group, respectively, and the cooperation of the two sites leads to enhanced activity and selectivity for the production of substitute aniline. In another example, binuclear PdCu sites generated on nanodiamond graphene show enhanced activity for selective hydrogenation of acetylene to ethylene than the isolated Pd and Cu sites due to optimized electronic feature of Pd atoms modified by the neighboring Cu atoms in the binuclear PdCu sites.^235^

Isolated metal atoms supported on solid carriers (such as Co atoms supported on N-doped carbon, Pt atoms supported on FeOx, etc.) have been reported as catalysts for selective hydrogenation reactions.^236,237^ In principle, the formation of binuclear bimetallic sites may further improve the catalytic performance because the H_2_ dissociation should be more favorable on two metal atoms than that on an isolated metal atom, and the chemoselectivity may also be modulated after the introduction of a neighboring metal atom because the adsorption configuration of the reactant can be influenced by the surrounding environment of the active sites, leading to chemoselective adsorption of the target groups.^238^ Therefore, we anticipated that more works in this direction will appear in the near future.

### 5.2 Binuclear Bimetallic Sites for Dehydrogenation Reactions

As the reverse process of hydrogenation, dehydrogenation reactions also widely exist in nature. For instance, alcohol metabolism relies on the participation of alcohol dehydrogenase, in which isolated Zn(II) sites are coordinated to the active site to assist the adsorption and activation of alcohol.^239^ In terms of homogeneous systems, both mononuclear and binuclear metal complexes have been employed for various dehydrogenation reactions, and in some cases, the binuclear bimetallic complexes give remarkable reactivities.^240^ Similar to the enzymes, dehydrogenation of alcohols to aldehydes can also be catalyzed by supported isolated metal species, such as Rh and Pt.^241,242^ For example, it is found that isolated Rh atoms supported on carbon are much more active than Rh nanoparticles for dehydrogenation of cyclohexanol to cyclohexanone. Following the above discussion, it will be interesting to check the possibility to incorporate another metal element as the neighboring site to Rh to further enhance the activity.

In heterogeneous systems, one of the most important dehydrogenation processes should be the dehydrogenation of light alkanes (propane, butane, and isobutane) for the production of light alkenes.^243^ Currently, supported CrOx and Pt catalysts are used in industrial processes. In the case of supported Pt catalysts, the active sites are generally considered to be small Pt clusters, while in the case of supported CrOx, atomically dispersed Cr(III) species are most likely to be the active species.^228,244^

**Figure 26:**
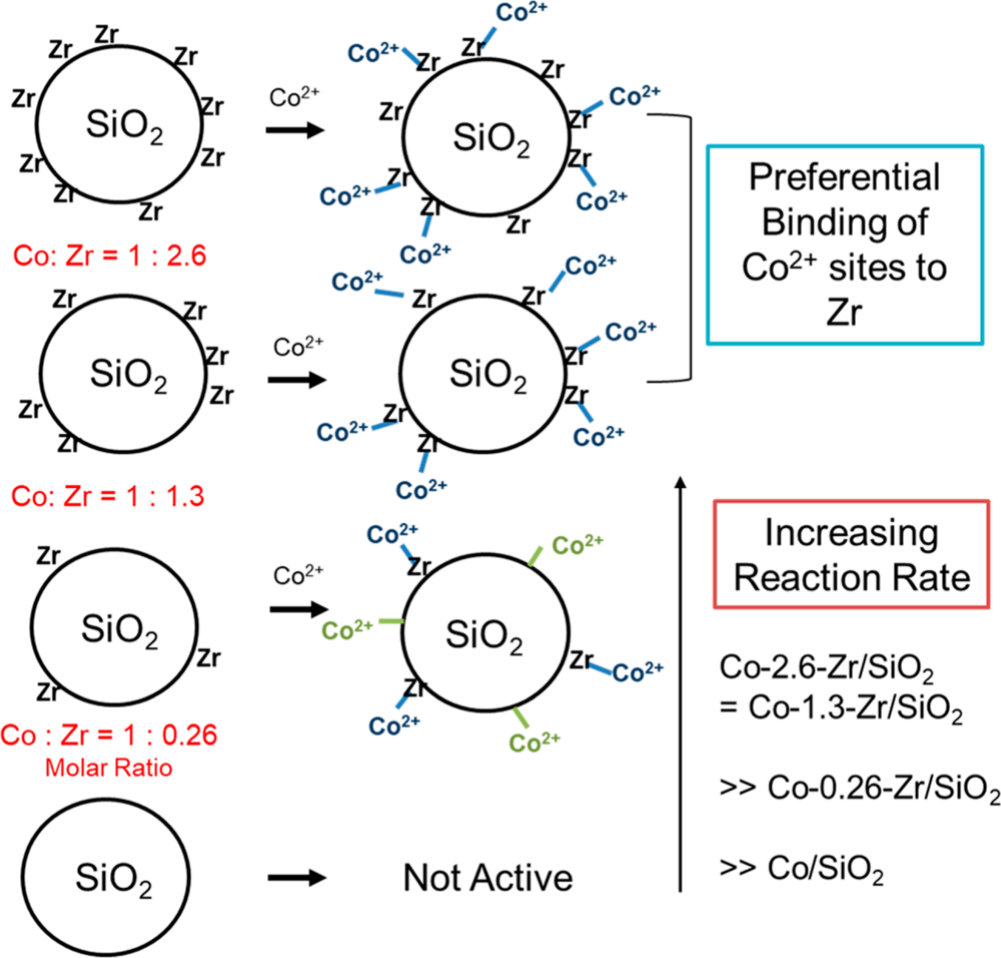
Schematic illustration of the ratio of Zr–Co in catalyst materials and Co–Zr/SiO_2_ catalyst site distribution. The Co–X lines (SiO_2_ or Zr) represent Co–O–X bonds. Reproduced with permission from ref (245). Copyright 2018 American Chemical Society.

Besides the materials based on supported mononuclear metal species, binuclear bimetallic species have also been reported as efficient catalysts for the dehydrogenation of alkanes. For instance, as described in Figure 26, the formation of binuclear CoZr sites is markedly more active than the monometallic Co and Zr sites for propane dehydrogenation, which is ascribed to the modification of the electronic structure of Co by Zr due to the metal-to-metal charge transfer.^245^ This enhancement effect has also been found with binuclear ZnAl sites, which are more active than isolated Zn species.^246^ Taking into account that a large number of supported isolated metal species (Cr, Fe, Ga, In, Sn, etc.) have been suggested as active sites for alkane dehydrogenation,^247,248^ the combination of these candidate elements could lead to the discovery of highly active catalysts comprising binuclear bimetallic sites.

### 5.3 Binuclear Bimetallic Sites for Oxidation Reactions

In natural enzymes, the oxidation of CO to CO_2_ can be realized by CO dehydrogenase, which contains MoCu bimetallic sites to perform the oxidation of CO with water (CO + H_2_O → CO_2_ + 2H^+^ + 2e^–^). The Mo and Cu atoms are bridged by S, and Mo is considered as the site for performing the redox process (between Mo^IV^/Cu^I^ and Mo^VI^/Cu^I^), while Cu is responsible for the CO capture and catalytic turnover.^250^ After the oxidation of CO to CO_2_, the Mo^VI^ site is regenerated by extracting oxygen from water.^251^ Solid catalysts made by mixed Cu-based oxide nanoparticles have been widely employed as excellent catalysts for CO oxidation, such as Cu–Mn mixed oxides, and the active sites in these solid catalysts are proposed to be associated with the Cu–O–Mn species,^252,253^ which could also be considered as motifs as binuclear bimetallic sites in CO dehydrogenase. The critical role of forming bridged M1–O–M2 species in mixed oxide catalysts for CO oxidation reaction has been validated by surface science study, in which the isolated Ni atoms deposited on CuO surface can catalyze the CO oxidation through the Eley–Rideal mechanism.^254^

**Figure 27:**
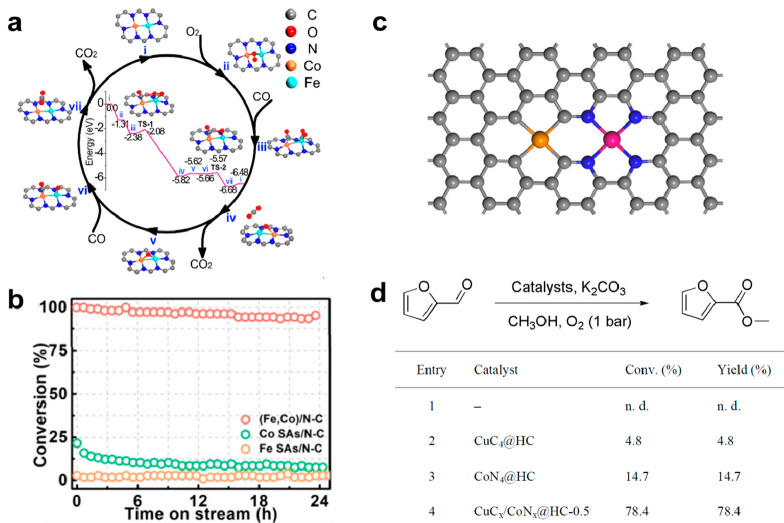
Supported binuclear bimetallic sites for oxidation reactions. (a,b) Binuclear CoFe sites embedded in N-doped carbon matrix for CO oxidation. The proposed reaction mechanism is shown in (a) and the catalytic performance for low-temperature CO oxidation reaction at −73 °C is shown in (b). The CoFe sites are much more active than the monometallic Co and Fe sites. (c,d) Binuclear CoCu sites embedded in N-doped carbon for oxidative esterification of furfural to methyl 2-furoate. The atomic structure of the bimetallic CoCu sites is displayed in (c) and the catalytic results are shown in (d). The CoCu sites give a higher yield than the monometallic Co and Cu sites, although all the catalysts are quite selective for this reaction. (a,b) Reproduced with permission from ref (72). Copyright 2020 American Chemical Society. (c,d) Reproduced with permission from ref (249). Copyright 2021 American Chemical Society.

By using N-doped carbon as the support, binuclear CoFe sites can be stabilized for mimicking the isolated CuMo sites in natural enzymes. As shown in Figure 27, CO is adsorbed on Co atoms, while O_2_ is activated on Fe atoms and the cooperation of the binuclear CoFe sites leads to much higher activity for CO oxidation reaction at a very low temperature (−73 °C) than the monometallic Co and Fe sites.^72^ Because low-temperature CO oxidation has also been achieved with Co_3_O_4_ nanoparticles,^255^ it will be interesting to study the similarity and dissimilarity between the Co atoms at the exposed facet of Co_3_O_4_ nanocrystals and the binuclear CoFe sites embedded in N-doped carbon matrix. Other applications of binuclear bimetallic sites embedded in N-doped carbon matrix have been demonstrated with CoCu sites for oxidative esterification of furfural to methyl 2-furoate and oxidative coupling–dehydrogenation cascade reaction of benzyl alcohol^249^ and 2′-hydroxyacetophenone for the synthesis of flavones.^256^ It is proposed that the neighboring Cu atom can promote the O_2_ activation on the Co atom and thus improve the yields of esters for the oxidative esterification of aldehydes.

By comparing the atomic structures of the binuclear sites and the reaction mechanism, we can find that the models proposed in the two works are different (metallic bonding is formed in the CoFe sites, while the Co and Cu atoms are not directly bonded in the other case). This discrepancy could be related to the difference in materials preparation procedure, but also could be caused by the difficulty of precisely elucidating the atomic structures of the binuclear bimetallic sites on the solid carrier, as discussed before in this review. Nevertheless, the two atoms in CoFe sites are proposed to account for the activation of CO and O_2_, respectively, while the Co atoms in CoCu sites seem to play a predominant role in oxidative esterification of aldehydes.

The activation of O_2_ is the critical step for oxidation reactions and the involvement of two or more atoms in the O_2_ activation step is necessary, as suggested by theoretical studies. However, during the past decade, isolated metal atoms supported on solid carriers have also been proposed as active sites for numerous oxidation reactions.^257,258^ From a mechanistic point of view, the investigations on supported binuclear bimetallic sites for oxidation reactions can help us clarify the reaction mechanism of the emerging single-atom catalysts based on isolated metal atoms. Moreover, in the reported works, the role of support in the supported binuclear bimetallic sites is not fully elucidated. The study with the model system based on Pt_2_ dimer supported on Fe_3_O_4_ surface indicate that the support together with the Pt_2_ dimer show reversible structural transformation during the active O species.^259^ These insights could be translated to the bimetallic systems, although the binding sites of O_2_ and the detailed mechanism could be more complicated than the monometallic systems.

Besides the activation of O_2_, the selective oxidation of organic molecules may invoke other key steps during the catalytic cycle. For instance, the binuclear IrCu sites supported on In_2_O_3_ exhibit superior performance for selective oxidation of biomass-derived isoeugenol to vanillin.^260^ The presence of an adjacent Cu atom to the Ir atom can promote the activation of O_2_ on the Ir site, and the Cu site accounts for the subsequent oxidation of the C=C bond to the epoxy group and then facilitates the hydrolysis of the epoxy group and the oxidative cleavage of C–C bond to generate the vanillin product.

### 5.4 Binuclear Bimetallic Sites for Other Organic Reactions

A large portion of the literature works on the catalytic application of binuclear metal sites are related to the transformation of organic molecules, and therefore, the activation of C–H bonds will be a critical step in many of these catalytic reactions. The use of supported TaIr bimetallic sites for activation of C–H bonds in the aromatic ring has been tested by performing an isotopic exchange experiment.^69^ The TaIr bimetallic sites are quite active for catalyzing hydrogen/deuterium exchange between C_6_D_6_ and C_6_H_5_F, while monometallic Ta sites are much less active. Moreover, the TaIr sites with Ta–H and Ir–H bonding are the active sites, while the Ta and Ir fully coordinated with metal–carbon bonding are not.

**Figure 28:**
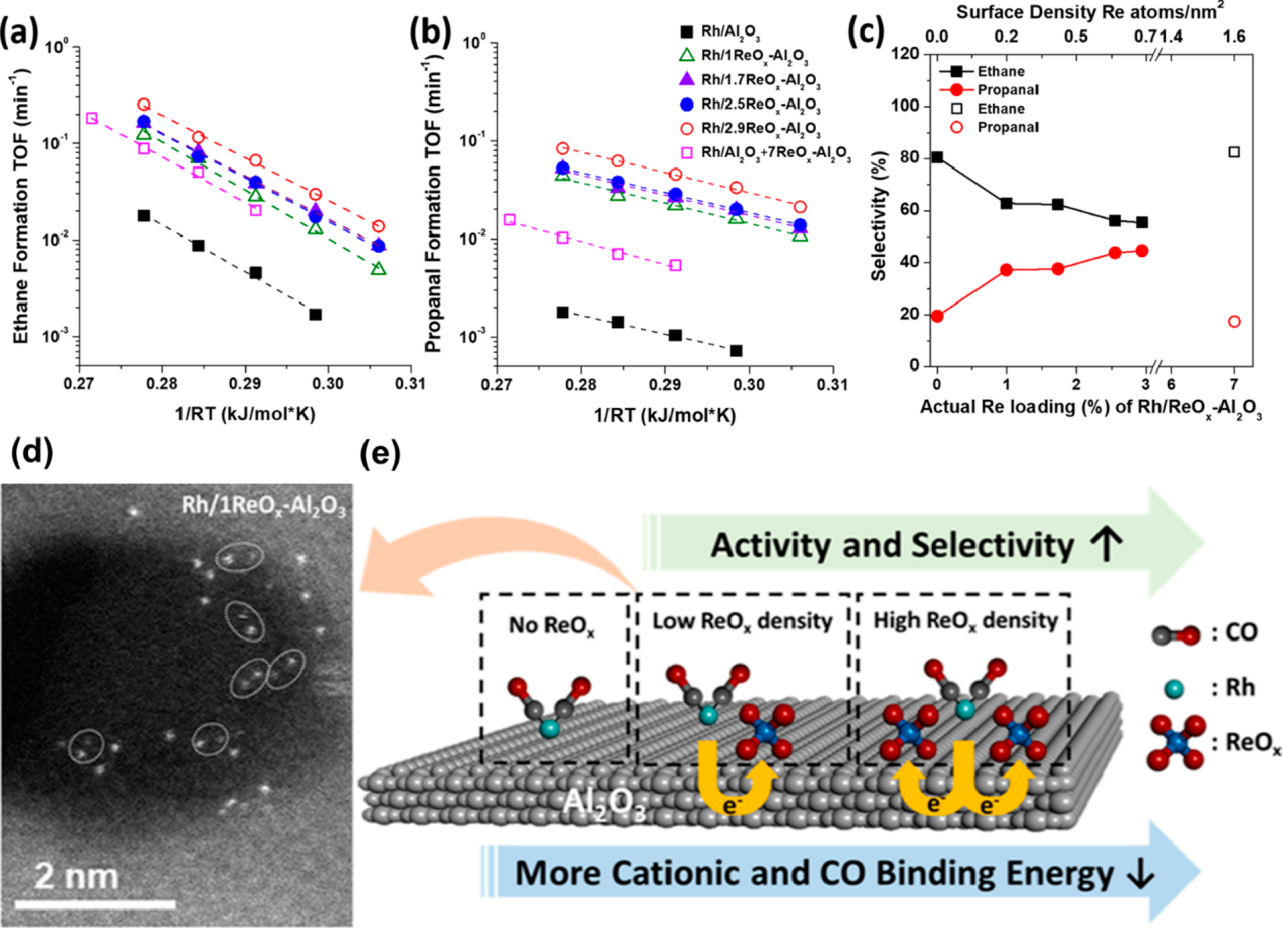
Supported RhRe binuclear sites for hydroformylation of ethylene. Normalized (a) ethane and (b) propanal formation rates at from 393 to 434 K. (c) Selectivity toward ethane (■) and propanal (red ●) as a function of actual ReOx loading in the Rh/ReOx–Al_2_O_3_. (Selectivity toward ethane (□) and propanal (red ○) over a physical mixture of Rh/Al_2_O_3_ and 7ReOx–Al_2_O_3_). (d) HAADF-STEM image of Rh/ReOx–Al_2_O_3_ after ex situ calcination at 623 K, showing the presence of binuclear species on Al_2_O_3_. (e) Schematic illustration of the promotion effect of Re to Rh for the hydroformylation of ethylene. Reproduced with permission from ref (261). Copyright 2019 American Chemical Society.

Hydroformylation of olefins is a very important process in the current chemical industry for the production of aldehydes. Currently, molecular Rh catalysts are employed in industrial processes and the addition of another metal can promote the reactivity of the mononuclear Rh catalyst because the binuclear bimetallic complex can facilitate the production of aldehyde through the binuclear elimination reaction within the RhRe complex.^262,263^ Following the works in the homogeneous systems, binuclear RhRe sites are supported on Al_2_O_3_ as heterogeneous binuclear bimetallic catalysts for the hydroformylation of ethylene.^261^ The introduction of atomically ReOx species in the neighboring site of isolated Rh atoms can cause the modification of the electronic structure of Rh atoms, as proved by the shift of the CO stretch frequency of the Rh(CO)_2_*gem*-dicarbonyl species. A dedicated theoretical study on the supported Rh–Re binuclear species suggests that the presence of Re at the neighboring site of Rh can weaken the Rh–CO bonding and the rate-determining step for the hydroformylation reaction is also switched from the acylation step to the requisite CO coordination before the insertion step.^264^ The mechanism for the promotion effect of Re to Rh catalyst is different in the homogeneous and heterogeneous systems, and the difference is proposed to be caused by the different reaction pathways, which should be related to the geometric and electronic differences between molecular binuclear complex and supported binuclear species. As shown in Figure 28, due to the modification of the electronic properties of Rh sites, the selectivity toward propanal is greatly improved in comparison to the monometallic Rh catalyst in hydroformylation reaction of ethylene, because the hydrogenation of ethylene is suppressed. The concept of constructing bimetallic sites for hydroformylation reaction has also been extended to Rh–WOx pair sites, which catalyzes ethylene hydroformylation reaction through a mechanism involving Rh-assisted WOx reduction, transfer of ethylene from WOx to Rh atom, and H_2_ activation and dissociation at the Rh–WOx interface.^265^

Another important reaction involving the activation of CO is the carbonylation of methanol for the production of acetic acid, which is catalyzed by a homogeneous Rh complex in the industrial process.^266^ Additionally, Ir-based catalysts have also been developed for this process and the addition of La to the Ir/C catalyst has been quite effective to enhance the performance. Mechanistic studies reveal that La can facilitate the normally rate-limiting CO insertion step in Ir-catalyzed carbonylation of methanol.^267,268^ Furthermore, the presence of La as the neighboring site to the Ir atom will suppress the leaching and sintering of atomically dispersed Ir species.^269^

**Figure 29:**
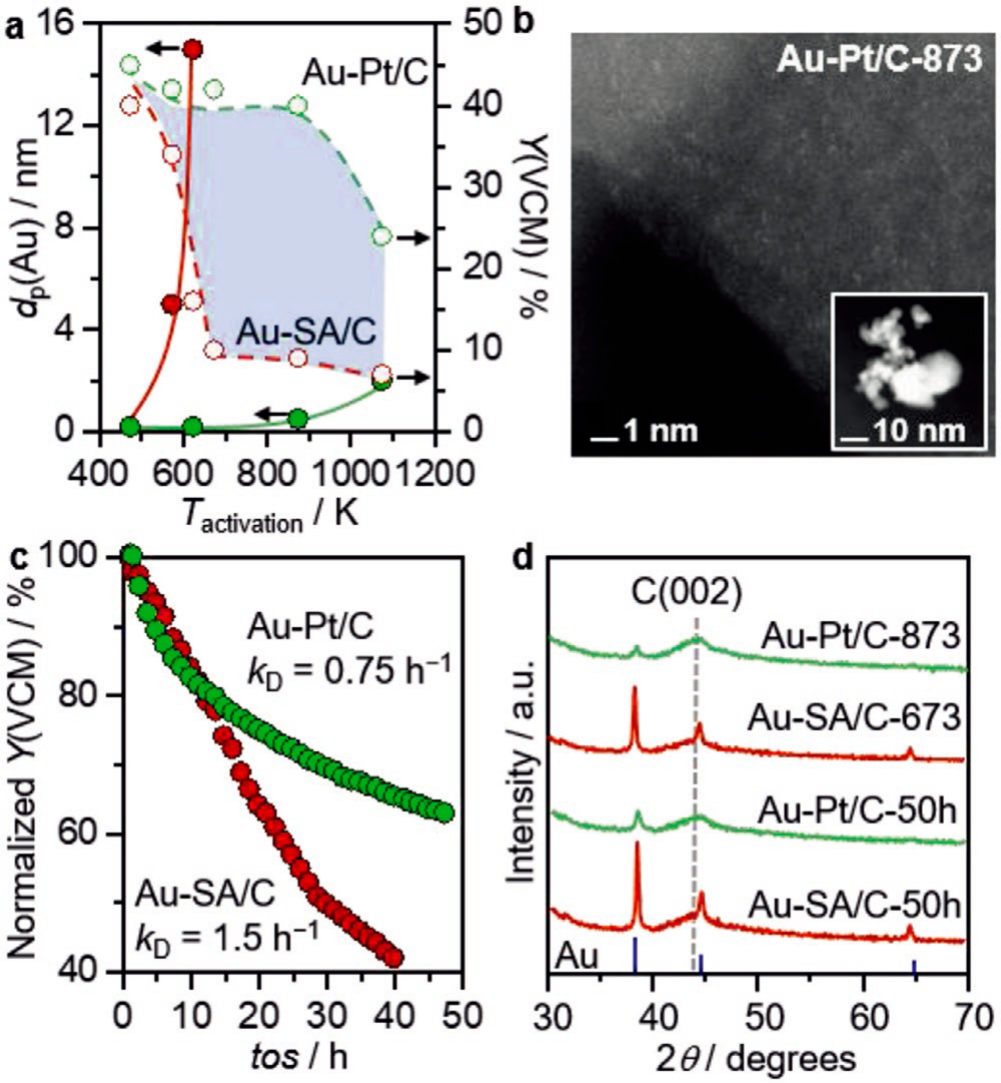
Supported AuPt bimetallic sites with good stability for acetylene hydrochlorination reaction. (a) Evolution of the Au particle size according to the HAADF-STEM measurements in the absence or presence of Pt atoms as a function of the thermal activation temperature and corresponding acetylene hydrochlorination activity, expressed as the yield of vinyl chloride monomer (VCM). (b) STEM image of Au–Pt/C after thermal activation at 873 K, showing the presence of many isolated metal atoms and occasional Au nanoparticles. (c,d) Comparison of the stability of Au–SA/C (comprising isolated Au atoms) and Au–Pt/C (comprising AuPt bimetallic sites) in acetylene hydrochlorination, accompanied by the respective deactivation constants (*k*_D_), and XRD patterns of both catalysts after 50 h time-on-stream (*tos*). Diffraction patterns of metallic Au and graphitic carbon are observed. Reproduced with permission from ref (271). Copyright 2021 Wiley-VCH.

The stability of binuclear bimetallic sites under the conditions for organic transformations could be a critical issue for IB group metals (such as Ag and Au) with higher reducibility than the VIII group metals, which limits their practical applications. For instance, isolated Au atoms supported on carbon suffer the sintering issue during the hydrochlorination of acetylene, due to the reduction of oxidized Au species (Au(I) and Au(III) species) into Au nanoparticles.^270^ As shown in Figure 29, the addition of isolated Pt atoms to the Au/C catalyst can effectively mitigate the sintering of Au atoms into Au nanoparticles because the migration and reduction of Au species are suppressed.^271^ Beside the addition of a noble metal, the introduction of alkali metals can also improve the stability of highly dispersed Au species supported by solid carriers. For example, the addition of cesium can also effectively enhance the stability of atomically dispersed Au species under the conditions of acetylene hydrochlorination reaction, because the Cs^+^ can inhibit the reduction and sintering of catalytically active Au(III) species to metallic Au.^272^ However, in both of the above examples, there is no direct experimental evidence of the formation of metal–metal bonding in the bimetallic catalysts. Instead, it is proposed that the second metal is anchored on the neighboring site of the Au(III) atoms embedded in the carbon support, which increases the energy barrier of the reduction and migration of Au atoms into Au nanoparticles on the carbon support. The distance between the two types of metal sites seems to be longer than the bimetallic binuclear sites discussed before in this section for electrocatalytic ORR reaction. Therefore, it is interesting to carry out further studies with these bimetallic catalysts to figure out the influence of the distance between the Au and the promotor metal species on the stability and catalytic reactivity, and thereby elucidate the structural features of the Au-based bimetallic sites.

**Figure 30:**
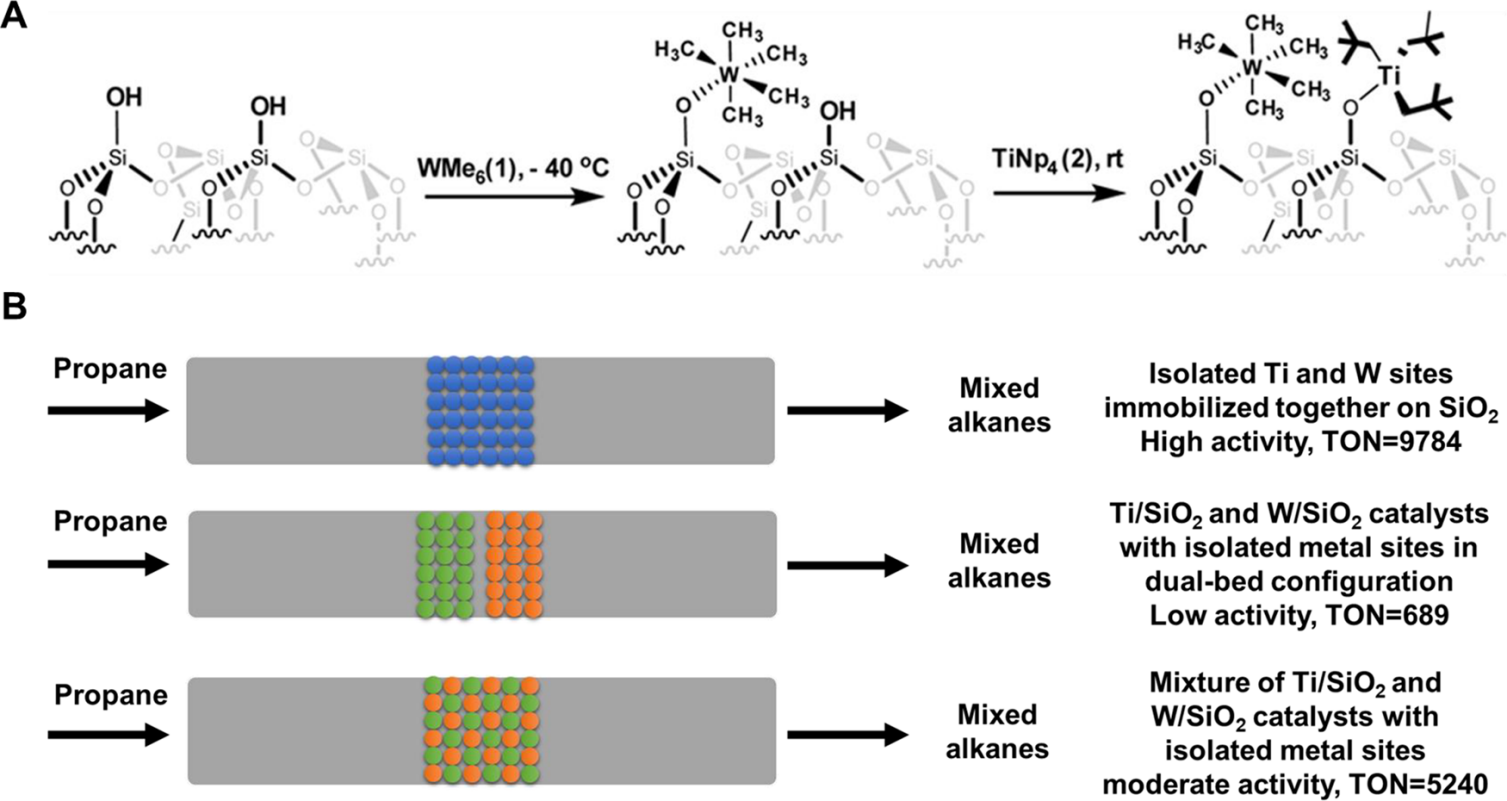
Metathesis reaction of propane with bifunctional supported organometallic catalysts. (A) Preparation of isolated Ti and W sites on amorphous silica support via surface grafting of organometallic complexes. (B) Different types of catalyst packing methods for continuous metathesis of propane. (C) Influence of the spatial distribution of the Ti and W sites on the catalytic performance of metathesis of propane. When the W and Ti sites were closely generated in the solid carrier, high activity for propane metathesis reaction could be obtained. On the contrary, when the two types of sites are separated, very low activity was observed. Reproduced with permission from ref (67). Copyright 2017 American Chemical Society.

The activation of alkanes has also demonstrated the critical role of binuclear bimetallic sites. Alkane metathesis, a very challenging reaction for adjusting the chain length of the inert alkanes, is considered to proceed via several elementary steps involving C–H activation and olefin metathesis.^273^ Monometallic sites such as isolated Zr, W, and Ta species supported silica show activity for metathesis of propane.^274,275^ To further improve the catalytic efficiency of alkane metathesis reaction, two types of isolated metal sites (W and Ti) are grafted on the support to work synergistically for the C–H activation and subsequent β-elimination step (see Figure 30), resulting in a significant improvement in activity for propane metathesis when increasing the intimacy of the two metal sites.^67^ However, according to the synthesis method and the structural characterization of the supported organometallic W–Ti catalyst, the distance between the W and Ti sites is still unknown. The surface organometallic chemistry allows the precise control of the spatial proximity of the two types of metal sites on silica surfaces by employing different organometallic complexes as the precursor.^276^ It will be interesting to directly graft the bimetallic W–Ti complex on the silica support to generate neighboring W–Ti sites to verify whether the alkane metathesis reaction requires bifunctional sites with atomic proximity.

### 5.5 Binuclear Bimetallic Sites for Radical Reactions

Supported isolated metal atoms have been reported as motifs homogeneous Fenton reagents, which can efficiently initiate the production of radicals in solution for radical reactions. For instance, isolated Fe atoms supported on N-doped carbon have been shown as active catalysts for activating peroxymonosulfate or H_2_O_2_ to produce ·OH species, which are responsible for the degradation of organic compounds and oxidation of benzene to phenol.^279−281^ The catalytic performance of the isolated Fe atoms is strongly dependent on the coordination environment. When increasing the coordination number of Fe–N bonding in the Fe–N–C catalyst, the reactivity for benzene oxidation reaction is greatly promoted due to the generation of highly active O=FeN_4_=O species.^280^

From a mechanistic point of view, the formation of radical species involves the activation of the precursor compound (e.g., H_2_O_2_, peroxymonosulfate, etc.) on the metal sites and subsequent dissociation of these compounds to form radical species. The electronic structures of the metal sites have profound impacts on the reactivity because of the electron transfer between the metal sites and the reactants. The advantages of binuclear bimetallic sites over the isolated metal sites for the generation of reactive radical species have been exhibited with MoZn and CuFe sites supported on carbon, which is ascribed to the enhanced capability for cleavage of O–O bonds in the peroxy compounds and the modification of the adsorption energy of hydroxyl.^282,283^

Beyond the reported works, the binuclear bimetallic sites are also promising catalysts for selective oxidation of light alkanes to oxygenates. Theoretical calculations suggest that FeNi and FeCo sites give much lower activation energies for C–H activation in methane than that on the Fe_2_ site.^284^ Taking into account that Fe_2_ sites stabilized in ZSM-5 can catalyze the conversion of methane to CH_3_COOH, which involves the coupling of CH_4_, CO, and ·OH produced from H_2_O_2_.^285^ Moreover, isolated Fe sites at the node of Fe-MOF structure can catalyze the oxidation of ethane to ethanol by using N_2_O as oxidant, which involves the Fe(IV)-oxo species as active species.^286^ Therefore, it will be interesting to explore the oxidation of light alkanes (ethane, propane etc.) with highly active radical species on binuclear bimetallic catalysts, to improve the activity and improve the selectivity of desired products. In the reported works, oxidation of ethane with H_2_O_2_ can be catalyzed by solid catalysts and a variety of products (including acetic acid, CO_2_, formic acid etc.) are formed with low turnover numbers.^287^ The employment of solid catalysts with regular binuclear sites may offer the opportunity to perform the oxidation of light alkanes with high chemoselectivity and regioselectivity.

### 5.6 Binuclear Bimetallic Sites for Photocatalytic Reactions

Molecular bimetallic complexes have been reported as photocatalysts for a variety of reactions, such as water oxidation and CO_2_ reduction. For example, the binuclear CoZn complex gives a much higher turnover number than monometallic CoCo and ZnZn complexes for photocatalytic reduction of CO_2_ to CO.^288^ The advantage of the bimetallic complex over the monometallic complex is ascribed to the enhanced absorption of OH- on Zn^II^ sites, which facilitate the proton-coupled electron transfer and subsequent cleavage of C–O in CO_2_ for production of CO.

**Figure 31:**
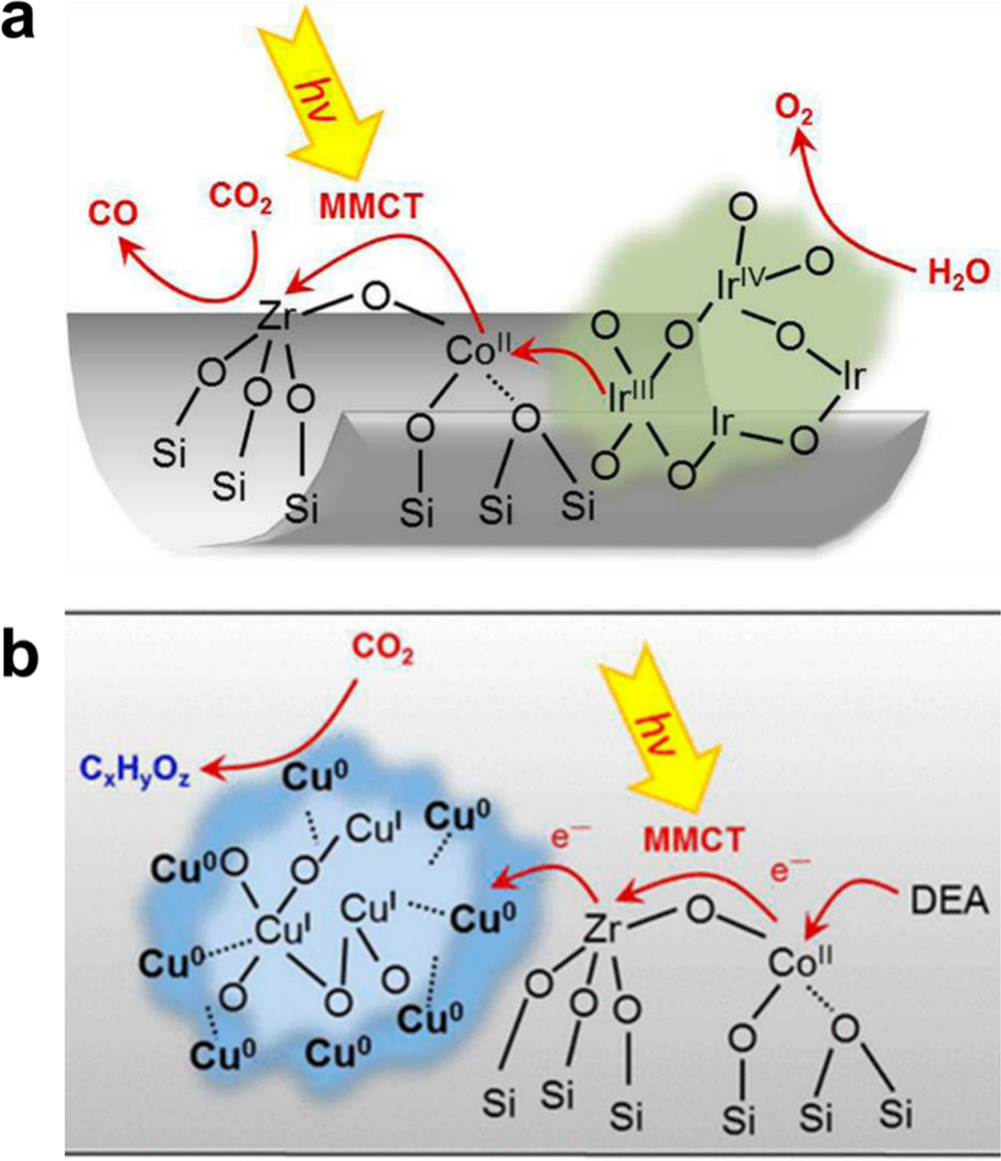
Photocatalytic reduction of CO_2_ with binuclear ZrCo sites and cocatalyst. (a) IrOx nanoclusters are employed as the cocatalyst to enhance the oxidation of water. (b) CuOx nanoclusters are employed as the cocatalyst to enhance the reduction of CO_2_. (a) Reproduced with permission from ref (277). Copyright 2014 American Chemical Society. (b) Reproduced with permission from ref (278). Copyright 2015 American Chemical Society.

As shown in Figure 31, by immobilizing the bimetallic complexes on solid carriers, they can work directly as the functional components for photocatalytic reactions or can serve as the cocatalysts of the light absorbents (e.g., semiconductors, plasmonic metals, etc.). In the former case, binuclear ZrCo sites are generated on mesoporous silica by grafting the organometallic Zr and Co precursor consecutively and the resultant binuclear ZrCo sites show activity for photocatalytic reduction of CO_2_ to CO in the presence of the sacrificial agent.^289^ The charge transfer between the Co^II^ and Zr^IV^ atoms is responsible for the light absorption and charge separation. Under light irradiation, the metal-to-metal charge transfer from Co^II^ to Zr^IV^ and then to the organic molecule has been directly followed by transient spectroscopy, while the reversed charge transfer pathway is prohibited, showing a rectified charge transfer behavior.^290^ Moreover, by combining the ZrCo sites with IrO_2_ nanoclusters, the photocatalytic reduction of CO_2_ to CO can be achieved directly with H_2_O in the absence of a sacrificial agent, thanks to the electron transfer from IrOx nanoclusters to the binuclear ZrCo sites and the enhanced oxidation of water on IrOx.^277^ The combination of ZrCo sites with CuOx nanoclusters can lead to an active system for photocatalytic reduction of CO_2_ to CO, as a result of the electron donation from ZrCo sites to CuOx nanoclusters and the favored reduction of CO_2_ on CuOx.^278^

In the latter case, the binuclear bimetallic sites supported on semiconductors can be efficient cocatalysts for photocatalytic H_2_ evolution, as demonstrated with CoPt sites supported on TiO_2_ nanosheets.^24^ The TiO_2_ nanosheets modified with CoPt sites are more active than those modified with isolated Pt atoms and Pt nanoclusters as cocatalysts. The electronic structure of the Pt atoms is modulated by the neighboring Co atoms, giving to promoted activity for the H_2_ evolution reaction. As demonstrated with CuIn sites supported on polymeric carbon nitride, the synergy of Cu and In sites contribute to the photocatalytic reduction of CO_2_ to ethanol via performing the C–C coupling of the intermediate CO adsorbed on the neighboring Cu and In site, respectively.^291^

Because of their versatile structures, MOFs can serve as a class of platform materials for installing different functional components to achieve catalytic transformations involving multiple steps. For instance, by incorporating isolated metal sites through the coordination interaction with the organic linker, both photosensitizer and catalytically active sites can be constructed in MOF structure for photocatalytic transformations, such as CO_2_ reduction and C–N coupling reactions.^292,293^ Although it is proposed that the photosensitizer and the transition metal sites are located inside the same cavity of the MOF structure, the two types of functional sites are separated in the MOF framework structure. Considering that photocatalytic transformations require the electron transfer from the photosensitizer to the transition metal sites, the energy transfer efficiency should be improved when the distance between the two functional sites is minimized. One possible approach is to incorporate binuclear bimetallic sites at the nodes of the MOF structure or binuclear complex at the linker.

### 5.7 Binuclear Bimetallic Sites for Electrocatalytic Reactions

As discussed in section 3 on the generation of binuclear bimetallic sites, a large portion of the reported catalysts is based on the binuclear species anchored on carbon-based materials, which are potential catalysts for electrocatalytic reactions because the carbon supports serve as the electron transport media. Developing non-noble metals as substitutes for Pt catalysts for fuel cells has attracted enormous attention among the electrocatalysis community in the last decades. According to many reported works, isolated metal atoms embedded in the N-doped carbon matrix (M–N–C species, M = Fe, Co, Ni, etc.) are considered the most promising candidate materials.^295,296^ Because the coordination environment of the isolated M species has profound impacts on the catalytic performance, the incorporation of a neighboring metal to the M to form binuclear bimetallic sites should also influence the reactivity of the M sites, as inferred by the theoretical calculations.^297^ Specifically, in the model constructed based on adjacent Co and Zn sites anchored on nitrogen-doped graphene, the d-band electronic structure of the Co site is influenced by the neighboring Zn site, and therefore, the bonding strength of the reaction intermediates for electrocatalytic oxygen evolution and reduction reactions are altered to enhance the catalytic performance.

**Figure 32:**
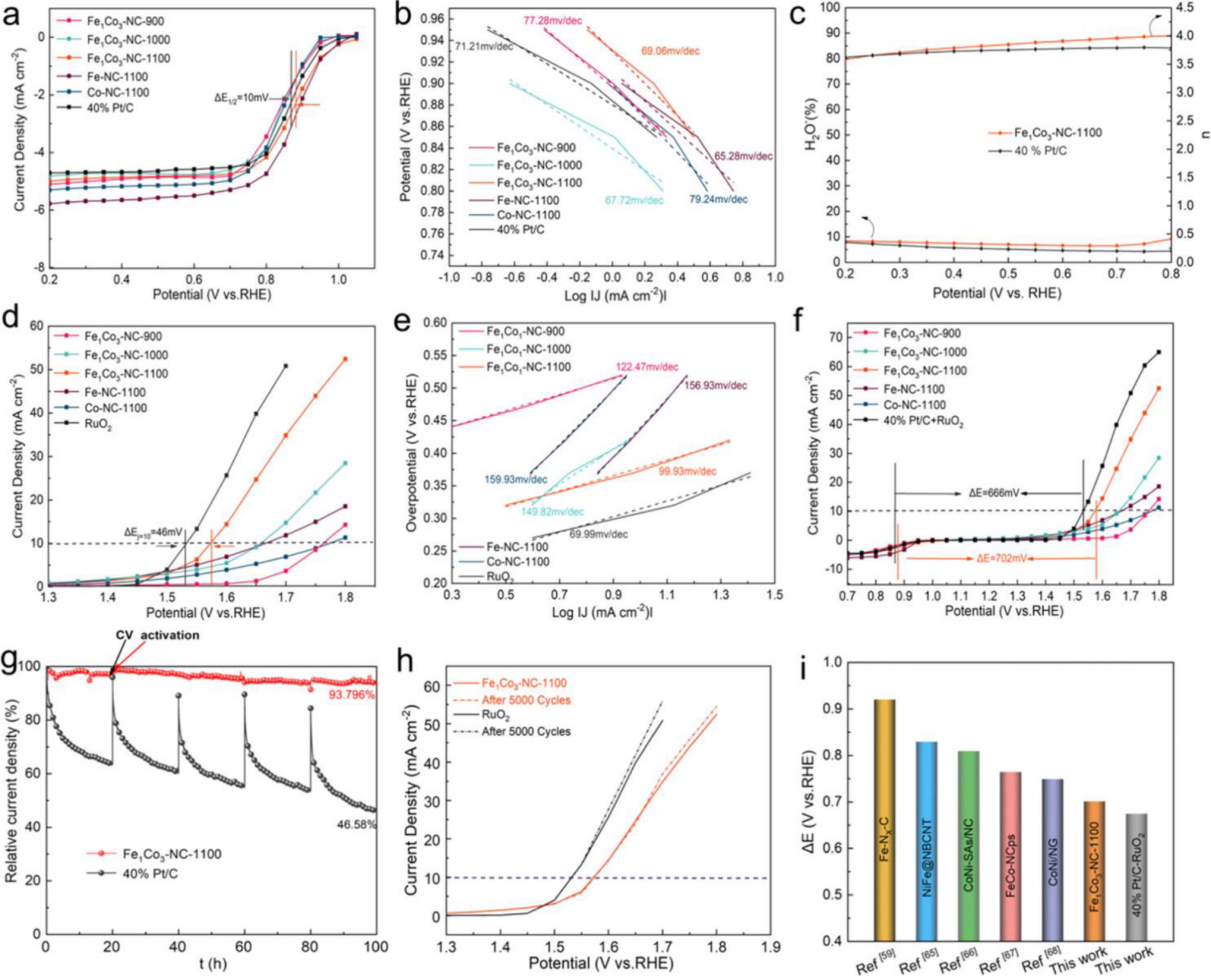
Catalytic performance of binuclear CoFe sites for electrocatalytic ORR. (a) Electrochemical measurements of the carbon-supported metal catalysts and 40% Pt/C. (b) Tafel plots of the ORR for various catalysts. (c) Rotating ring-disk electrode tests with the percentage of peroxide and electron transfer number of the Fe_1_Co_3_–NC-1100 and the 40% Pt/C catalysts. (d) Oxygen evolution reaction polarization curves of different catalysts. (e) Tafel plots of the OER for various catalysts. (f) Overall linear sweep voltammetry curves for the ORR and OER of various catalysts. (g) ORR stability test for the Fe_1_Co_3_–NC-1100 sample and 40% Pt/C catalysts. (h) OER stability test of the Fe_1_Co_3_–NC-1100 and RuO_2_ catalysts. (i) Δ*E* comparison of the Fe_1_Co_3_–NC-1100 and other reference catalysts. Reproduced with permission from ref (294). Copyright 2022 American Chemical Society.

In terms of the experimental studies, binuclear CoFe sites embedded in N-doped carbon derived from the pyrolysis of a CoFe-MOF precursor are found to be more active than the mononuclear metal sites prepared in a similar procedure for electrocatalytic oxygen reduction reaction (ORR).^298^ According to the structural characterization, it is proposed that the bimetallic FeCoN_5_–OH sites are ∼20 times more active than the monometallic FeN_4_ site because the Co and Fe atoms can work synergistically to enhance the cleavage of O–O bonding in O_2_. In another work, the binuclear CoFe sites are also generated by pyrolysis of a CoFe-MOF precursor, and the atomic structure of the active site is proposed to be CoFe-N_6_ moieties, which are more active than the monometallic counterparts for both oxygen reduction and evolution reactions (see Figure 32).^294^ The performance of the optimized catalyst with CoFe binuclear sites show more superior performances than the benchmark RuO_2_ and Pt/C catalysts for OER and ORR, respectively. The discrepancy between the above two works in terms of the structures of the active sites could be caused by the differences in the experimental procedure for catalyst synthesis. Nevertheless, it should be emphasized again that the determination of the coordination environment of the binuclear bimetallic sites on solid carriers is still a very difficult task, limited by the characterization techniques.

Thanks to the progress made in terms of controllable synthesis of binuclear bimetallic sites, it is possible to systematically study and compare the reactivity of different combinations of transition metals for electrocatalytic reactions. For instance, by in situ activation of a Ni–N–C catalyst comprising atomically dispersed Ni species in the electrolyte containing Fe^3+^, binuclear NiFe sites could be generated under the conditions for electrocatalytic reaction, as probed by in situ EXAFS.^299^ The in situ generated binuclear NiFe species are much more active than the isolated Ni and Fe atoms. It should be noted that, compared to the above-mentioned works, the NiFe sites generated under in situ conditions have more open coordination space than those embedded in the N-doped carbon matrix. By varying the metal ions in the electrolyte and the metal in the carbon matrix, the catalytic performances of various binuclear bimetallic sites have been studied, among which CoFe sites deliver the best performance for electrocatalytic oxygen evolution.

The advantages of binuclear bimetallic sites for electrocatalytic oxygen reduction/evolution reactions have been demonstrated with other binuclear bimetallic sites embedded in N-doped carbon matrix, such as the IrCo, CoFe, NiFe, RuCo, and IrFe sites.^300−305^ In most of the proposed atomic structures of the binuclear sites, the two metal atoms are coordinated by six N atoms and bridged by two N atoms, although there are differences in the synthesis methods among these works. Despite the numerous reports on application of binuclear bimetallic sites for electrocatalytic oxygen reduction/evolution reactions and the similarities in the structure of the active sites, benchmarking of the catalytic performances of various binuclear bimetallic sites under the same testing conditions is urgently needed to achieve a unified understanding on the structure–reactivity relationship in this direction.

Although some works show the enhanced catalytic performance of binuclear bimetallic species over the isolated metal sites, the long-term stability of the binuclear bimetallic sites under electrocatalytic conditions are not fully explored and compared with the classic systems. From a mechanistic point of view, a major reason causing the deactivation of the classic M–N–C materials is related to the degradation of the carbon support and the subsequent leaching of metal species into electrolyte under electrocatalytic conditions.^306^ In this sense, the stability of the binuclear bimetallic species against leaching and sintering should be carefully examined and compared with the materials comprising isolated metal species. It is possible that, by using binuclear bimetallic sites, the degradation of the carbon support caused by the radicals from Fenton reaction could be suppressed.^307^ Despite the tremendous efforts made to clarify the nature of the active sites in non-noble-metal based electrocatalysts (most of the works are focused on the M–N–C systems),^308^ there are still some arguments on the active sites due to the complicated composition of the M–N–C materials derived from pyrolysis treatments and the role of agglomerated metal species (in the form of metallic nanoparticles or carbide nanoparticles encapsulated in thin carbon layers) in the M–N–C catalyst is still not fully unveiled.^309^

Moreover, the superior electrocatalytic performance of binuclear bimetallic sites have also been observed for other electrocatalytic reactions. For example, WMo bimetallic sites exhibit excellent activity for electrocatalytic H_2_ evolution in a large pH range.^310^ By extensive theoretical studies, it is proposed that, the W and Mo atom are located in the defective sites of N-doped graphene and stabilized by Mo–O and W–O bonding. The W–O–Mo–O–C ensembles are responsible for the high activity for H_2_ evolution in pH-universal electrolysis. RhFe bimetallic sites presents enhanced activity for electrocatalytic H_2_ evolution in acid medium than the reference materials comprising Rh or Fe nanoparticles, which is ascribed to the bonding between Rh and Fe atom.^311^ The important role of chemical bonding between the two metal atoms is also reflected in the bimetallic AuRu sites for electrocatalytic H_2_ evolution reaction.^312^ Because Au is more electronegative than Ru, electron donation from Ru to Au may occur, which facilitates the H^+^ adsorption according to the d-band center theory.

**Figure 33:**
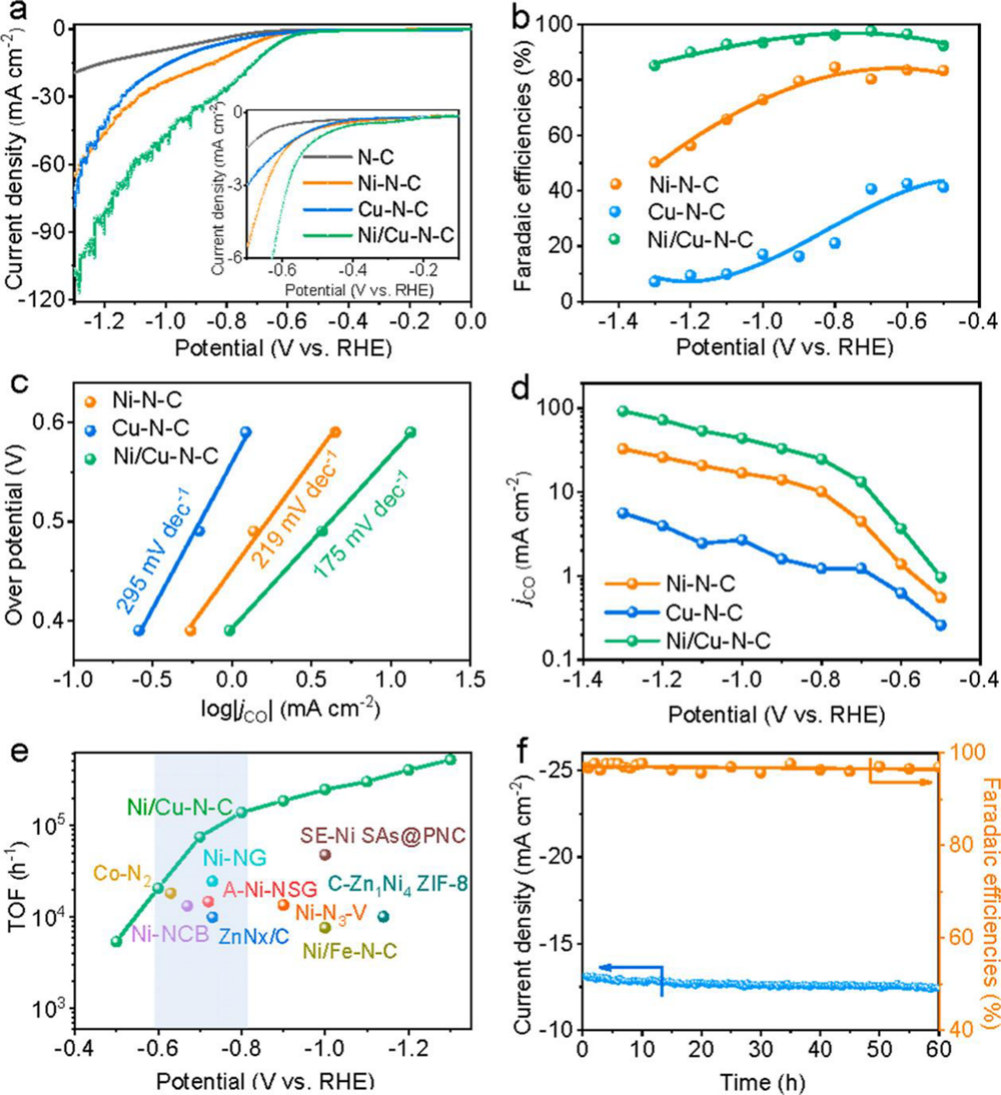
NiCu binuclear sites for electrocatalytic CO_2_ reduction. (a) Linear sweep voltammetry curves acquired in CO_2_-saturated 0.5 M KHCO_3_ solution on a rotating disc electrode at a rotating speed of 1600 rpm. The inset highlights the LVS curves in the potential range from −0.1 to −0.7 V. (b) Faradaic efficiency for CO production at various applied potentials. (c) CO partial current density at various applied potentials. (d) Tafel plots for CO production on various electrodes. (e) TOF of Ni/Cu–N–C compared with those of the state-of-the-art atomically dispersed metal catalysts. (f) Current–time response and the corresponding Faradaic efficiency for CO production on NiCuN_6_ at a fixed potential of −0.7 V. Reproduced with permission from ref (313). Copyright 2022 American Chemical Society.

In addition, binuclear bimetallic sites show promising performances for electrocatalytic CO_2_ reduction. Binuclear NiFe and NiCu sites embedded in N-doped carbon matrix have been reported as efficient catalysts for reduction of CO_2_ to CO, although the proposed atomic structures of the binuclear sites are different, according to the structural characterizations by AC-STEM and EXAFS.^313,314^ In the case of NiFe sites, it is claimed that the two metal atoms coordinate with six N atoms via forming two bridged Ni–N–Fe bonding. In the case of NiCu sites, the proposed structure consists of Ni and Cu atoms bonded with six N atoms, but in a nonbridged configuration. The different structural features of the binuclear bimetallic sites could be caused by the different electronic properties of Fe and Cu atoms, as implied by the theoretical calculations. In both works, the formation of binuclear bimetallic sites contributes the facile formation of *COOH intermediates, which is generally considered as the rate-determining step for electrocatalytic CO_2_ reduction. It occurs by modulating the electronic structures of the Ni atom with a neighboring Cu/Fe atom, resulting in enhanced faradic efficiency for CO production than the single-atom metal catalysts at low overpotentials, as shown in Figure 33. The remarkable advantages of binuclear bimetallic sites over the single-atom sites for electrocatalytic reductio of CO_2_ to CO have also been found in other combinations such as CuCo and NiIn sites, although there are some differences in the proposed structures of the binuclear sites.^315,316^

Besides the promotion effect ascribed to the electronic modification, the synergy of two metal atoms for CO_2_ activation could also be a plausible explanation for the enhanced activity for electrocatalytic CO_2_ reduction. Inspired by the active site of CO dehydrogenase (CODH),^317^ a metalloporphyrin-based mimic has been synthesized based on the combination of Zn and Fe metalloporphyrin complex. The cooperativity between the two metal atoms was demonstrated by the enhanced performance in reduction of CO_2_ to CO in comparison with the mononuclear analogue.^318^ It is proposed that the activation of CO_2_ is proceeded through the adsorption of CO_2_ on both metal centers. The synergistic effects of binuclear bimetallic sites for electrocatalytic CO_2_ reduction have also been observed in other combinations.^319,320^ The studies based on well-defined molecular complexes provide fundamental understanding on the atomic structures of the active sites in binuclear bimetallic sites and could be translated into the studies based on binuclear sites embedded in carbon matrix.

However, none of the above-mentioned binuclear bimetallic sites can achieve the production of C2+ products (such as ethylene, ethanol etc.), although these C2+ products are commonly observed in CO_2_ reduction reactions with Cu and Co nanoparticles.^321,322^ One plausible explanation could be the absence of domains with multiple metal atoms in the binuclear sites for C–C coupling reactions to form C2+ products. In this regard, the combination of binuclear bimetallic sites and Cu nanoparticles may lead to superior performance for reduction of CO_2_ to C2+ products via a tandem catalytic process because the CO_2_ to CO and CO to C2+ products can proceed separately.^323^

The contribution of the two competing reactions (CO_2_ reduction and H_2_ evolution reaction) at the cathode region can be tuned to give syngas with desired CO_2_/H_2_ ratio. For example, ZnLa bimetallic sites supported on N-doped carbon show tunable activity for electrocatalytic reduction of CO_2_ to CO and the ratio of CO/H_2_ in the resultant product can be modulated via tuning the number of Zn and La sites because the Zn and La sites tend to produce CO and H_2_, respectively.^324^

### 5.8 Other Potential Applications of Binuclear Bimetallic Sites

Limited by the availability of catalysts comprising binuclear bimetallic sites, the applications of this type of materials are not as abundant as bimetallic nanoclusters and nanoparticles, up to now. However, there are some examples showing promising catalytic performance of monometallic binuclear sites.^325^ For instance, binuclear Ir species supported on Fe_2_O_3_ show better activity than isolated Ir atoms and Ir nanoparticles for photocatalytic water oxidation reaction.^326^ Binuclear Ir species supported on SiO_2_ and MgO show activity for hydrogenation of ethylene.^327^ Besides, the binuclear Fe species stabilized on C_3_N_4_ exhibit remarkable activity and selectivity for epoxidation of epoxidation of *trans*-stilbene, while isolated Fe atom and Fe nanoparticles are barely inactive.^328^ The unique catalytic behavior of binuclear Fe species is also reflected for oxidation of CH_4_ in the presence of CO and H_2_O_2_, giving to the production of acetic acid.^329^ The use of monometallic binuclear sites for alkane activation reactions have been shown with binuclear Cu and Ga sites, which are stabilized in the micropores of zeolite structures.^330,331^ More examples can be found with the application of monometallic binuclear sites for electrocatalytic reactions, such as the binuclear Co and Fe sites embedded in N-doped carbon for oxygen evolution reaction and oxygen reduction reaction.^332,333^ The above-mentioned experimental works have also shown the advantages of monometallic binuclear sites over isolated metal atoms or nanoparticles. The catalytic performances could be further improved by substituting the monometallic binuclear sites by bimetallic binuclear sites and the choice of the bimetallic combination could be inferred by theoretical calculations.^25,334−336^ This strategy has already been practiced in many theoretical works for screening in the structural configurations of the potential binuclear bimetallic sites. Especially, by employing the calculations based on machine-learning strategies, the efficiency of theoretical screening will be greatly promoted.

The choice of support for binuclear sites has remarkable impacts on their catalytic properties. For example, metallic Pt_2_ clusters can be stabilized in the microporous channels of MOF structure, and these binuclear species give remarkable activity for low-temperature water–gas shift reaction.^337^ The electronic properties of the Pt_2_ clusters are different to those supported on inorganic solid carriers, which are positively charged.^338^ Translating this lesson to binuclear bimetallic sites, screening suitable host for the binuclear sites and establishing the structure–reactivity relationship will be critical for developing binuclear bimetallic sites with novel catalytic properties.

For developing novel supported binuclear bimetallic catalysts, one may achieve that goal by translating the knowledge and fundamental understandings obtained with enzymatic and homogeneous systems. This paradigm could be quite applicable when the target reactions are those dealing with organic molecules, because the rate-limiting steps of the target reaction could be quite similar to those catalyzed by enzymes or molecular metal complexes. By mimicking the composition and structural features of the corresponding enzymes or metal complexes, one could develop active and selective catalysts based on binuclear bimetallic sites. For instance, in conventional the Sonagashira couple reactions, it usually requires the use of both Pd and Cu complex for achieving high reaction efficiency, because Cu is generally considered as a promotor metal for Pd.^339^ It has been shown that bimetallic PdCu nanoparticles have shown advantages over monometallic Pd and Cu particles.^340^ However, the leaching of nanoparticulate Pd and Pd-based nanoparticles in C–C coupling reactions is widely observed, therefore, the nature of the active sites in the bimetallic system remains unknown. It is found that the use of bimetallic PdCu complex to maximize the synergy of Pd and Cu metal sites and the reaction mechanism in the elementary steps, such as the formation of aryl/acetylide species and C–C elimination.^341^ Following these works, it is interesting to develop binuclear PdCu species stabilized on solid carriers such as carbon-based materials or porous matrix for C–C coupling reactions.

In some catalysts comprising monometallic binuclear sites, the chemical states of the two metal atoms could be different, caused by their different coordination environment. For instance, the binuclear Cu sites supported on PdTe nanowires are proposed to be paired Cu^0^–Cu^*x*+^ species. The metallic Cu^0^ accounts for adsorption of CO_2_ while the positively charged Cu^x+^ accounts for the adsorption of H_2_O, resulting in high activity and selectivity for reduction of CO_2_ to CO.^342^ Taking into account the possible scenarios, in which the same type of metal atoms at neighboring sites show different chemical states, the synergy of the two metal atoms in different chemical states may work like two atoms of different elements. This type of binuclear structures could commonly exist in solid catalysts comprising atomically dispersed 3d transition metals (Fe, Co, Cu, etc.), because these metals can exhibit flexible transitions between different chemical states.^343^

In recent years, we have witnessed the renaissance of electrocatalysis for organic synthesis^344,345^ and the design of efficient solid electrocatalysts for organic reactions has been one of the frontiers in catalysis community, especially in the field of biomass conversion.^346,347^ Considering the use of bimetallic molecular complexes in numerous organic synthesis procedures, it is logical to expect that binuclear bimetallic sites supported on carbon could be promising catalytic materials for organic transformations.

### 5.9 Perspectives

In most of the catalytic reactions discussed above, monometallic sites can already catalyze the transformations, although the use of binuclear bimetallic sites may perform better. More attractive examples for extending the potential of binuclear bimetallic sites will be the ones that can only be achieved with the combination of two metal atoms while a single metal atom is not sufficient. In this sense, there is much space for the development of binuclear bimetallic sites for tandem catalytic processes, in which each of the metal atom accounts for at least one elementary step. In this sense, the synergy between the two metal atoms will determine the catalytic performance of the whole process.

The identification of mechanism of binuclear bimetallic sites is still a very challenging task, because the adsorption sites for the substrate molecules and potential dynamic structural transformations of the binuclear species are very difficult to be unveiled. As a consequence, many of the literature works rely on theoretical calculations to understand the reaction mechanism. A potential approach to acquire molecule-level insights on the reaction mechanism is to use the organometallic complexes to mimic the corresponding binuclear bimetallic species and the reaction mechanism based on the well-defined organometallic complexes, which can be studied by well-established tools and methods (NMR, X-ray diffraction etc.), can provide direct insights on the counterpart species supported on solid carrier. This paradigm has been practiced with the application of model metal complexes to study the functional mechanism of metalloenzymes, such as the employments of binuclear Fe complex for mimicking NO reductase and FeAl binuclear bimetallic complex for activation of CO_2_.^348,349^

The examples discussed in this section are mostly focused on binuclear bimetallic sites stabilized on solid carriers, especially for those bimetallic sites embedded in N-doped carbon matrix. For those species, their coordination environments are largely constrained by the metal–support interaction. However, binuclear metal entities can be produced in a highly dynamic manner under reaction conditions, as demonstrated with the formation of binuclear Cu sites in CHA zeolite under the conditions for NH_3_–SCR reaction.^350,351^ The solvation effect of NH_3_ on Cu species drive the migration of Cu species across the cages of CHA zeolite and the dimerization of Cu–NH_3_ complex are responsible for catalyzing the NH_3_–SCR reaction. Taking into account the reactant-induced dynamic structural transformation of atomically dispersed species on solid carriers, the dynamic formation of binuclear bimetallic sites could probably be present in the supported bimetallic catalysts, which to the best of our knowledge have not been reported yet. This possibility deserves to be checked in future works related to the zeolite-supported bimetallic catalysts.

## 6 Catalytic Applications of Bimetallic Nanoclusters

In comparison with binuclear bimetallic sites, the high atomicity of bimetallic nanoclusters leads to distinct geometric and electronic structures, which will cause different patterns in terms of the interaction between the metal sites and the substrate molecules and thus different catalytic properties. In this section, we will discuss the catalytic applications of bimetallic nanoclusters for a variety of reactions and the structure–reactivity relationships.

### 6.1 Bimetallic Nanoclusters for CO Oxidation

**Figure 34:**
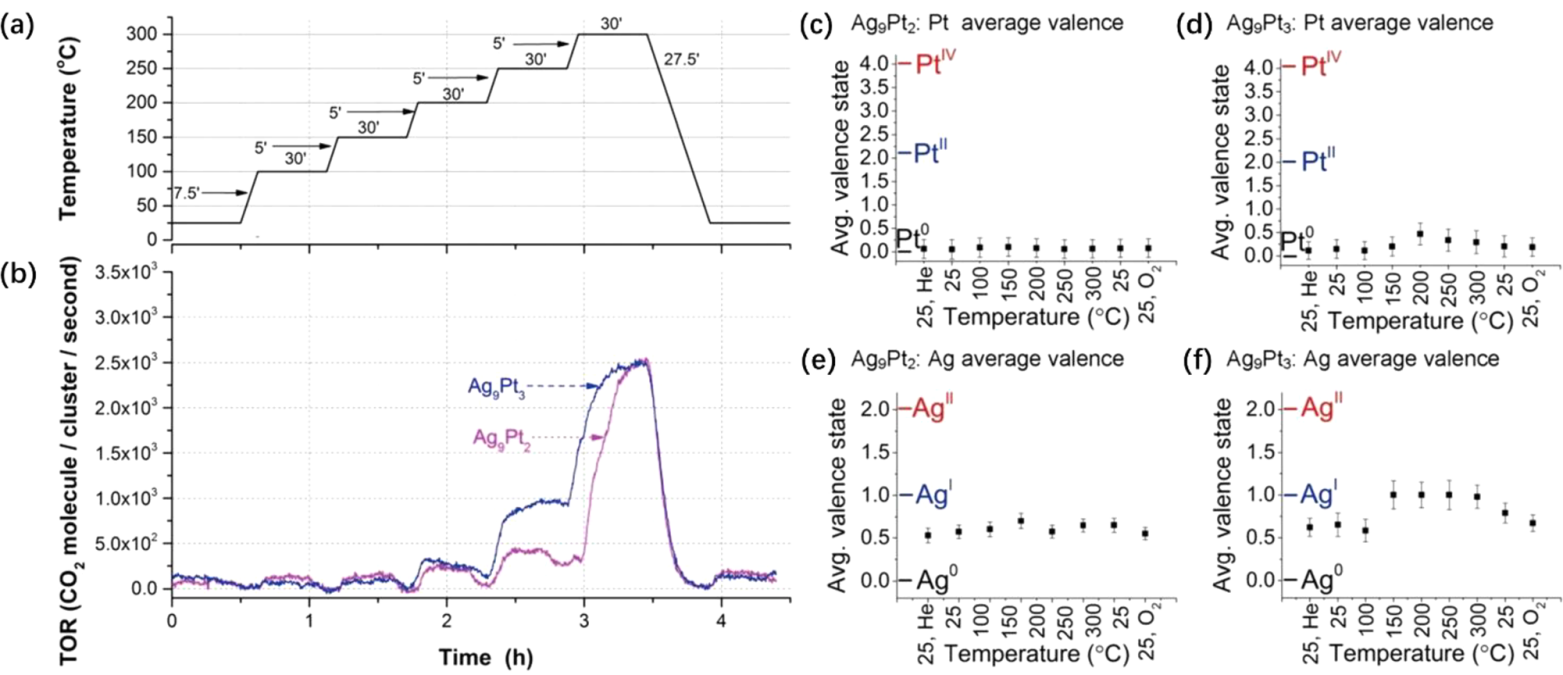
Catalytic performance and chemical states of AgPt bimetallic clusters for CO oxidation reaction. (a) The profile of the reaction temperature and (b) the normalized formation rates of CO_2_ on Ag_9_Pt_2_ (pink) and Ag_9_Pt_3_ (blue) clusters. (c–f) The average valence states of platinum and silver in alumina-supported bimetallic Ag_9_Pt_2_ and Ag_9_Pt_3_ clusters during CO oxidation. Reproduced with permission from ref (83). Copyright 2018 Wiley-VCH.

As one of the most important model reactions, CO oxidation has been frequently employed as the probe reaction to study the catalytic behavior and physicochemical properties of supported bimetallic clusters. For instance, the Ag_9_Pt_2_ and Ag_9_Pt_3_ clusters generated by the size-selected method (a physical method for generation of metal clusters with precise composition as model catalysts but with low yields^84^) have been tested for CO oxidation reaction and the catalytic results indicate that Ag_9_Pt_3_ clusters are more active than the Ag_9_Pt_2_ clusters at 200–250 °C (see Figure 34a,b).^83^ The composition-dependent reactivity of AgPt clusters is ascribed to the favorable activation of O_2_ and CO at Ag and Pt atoms, respectively. The difference in reactivity could be associated with the chemical states of the Ag and Pt species in the two types of AgPt clusters, in which the more active Ag_9_Pt_3_ show a higher number of oxidized species than the Ag_9_Pt_2_ clusters (see Figure 34c–f).

Using ligand-protected bimetallic clusters as a model catalyst, the introduction of Cu into Au_25_ clusters can slightly promote the activity for CO oxidation while the doping with Ag causes negative effects.^352^ Theoretical calculations indicate that the promotion of the second metal (Cu) to Au clusters could be associated with the formation of oxidized Cu species, which enhance the adsorption of CO and activation of O_2_.^353^ However, this observation is controversial to the promotion effect observed with bimetallic AuAg clusters supported on zeolite (pure-silica ITQ-2). This discrepancy could be related to the different reaction temperatures of the catalytic tests (<200 °C versus >200 °C).^354^ Besides, due to the higher reaction temperatures, the sintering of AuAg clusters on zeolite is also observed during the catalytic tests for CO oxidation reaction due to the removal of the thiolate ligands. This evolution behavior of bimetallic clusters has also been observed with bimetallic AuPd clusters supported on TiO_2_, which evolve into PdAu nanoparticles during the CO oxidation reaction.^355^

The advantages of bimetallic clusters over monometallic clusters for CO oxidation are shown in the CeO_2_-supported PtCu clusters.^356^ Generated by ALD, subnanometer Pt clusters are deposited on the (220) facets of Cu-doped CeO_2_ nanorods, forming interfacial PtCu sites on the surface of CeO_2_. The resultant bimetallic PtCu clusters are more active than the isolated Pt atoms and Pt clusters, owing to the unique interfacial structure for facilitating the adsorption and activation of CO and O_2_.

### 6.2 Bimetallic Nanoclusters for Oxidation Reactions

In natural photosynthesis systems, the active site for water oxidation reaction is a bimetallic cluster made by Ca and Mn (CaMn_4_O_5_ cluster), which can activate H_2_O and produce O_2_ in Photosystem II.^357^ Although the detailed mechanism of the water oxidation reaction in the Photosystem II is still not fully elaborated, the extraction of electrons from H_2_O and the charge transfer process between the metal atoms within the CaMn_4_O_5_ cluster is the key.^358^ This lesson is also reflected in the catalytic oxidation reactions based on supported bimetallic nanoclusters.

**Figure 35:**
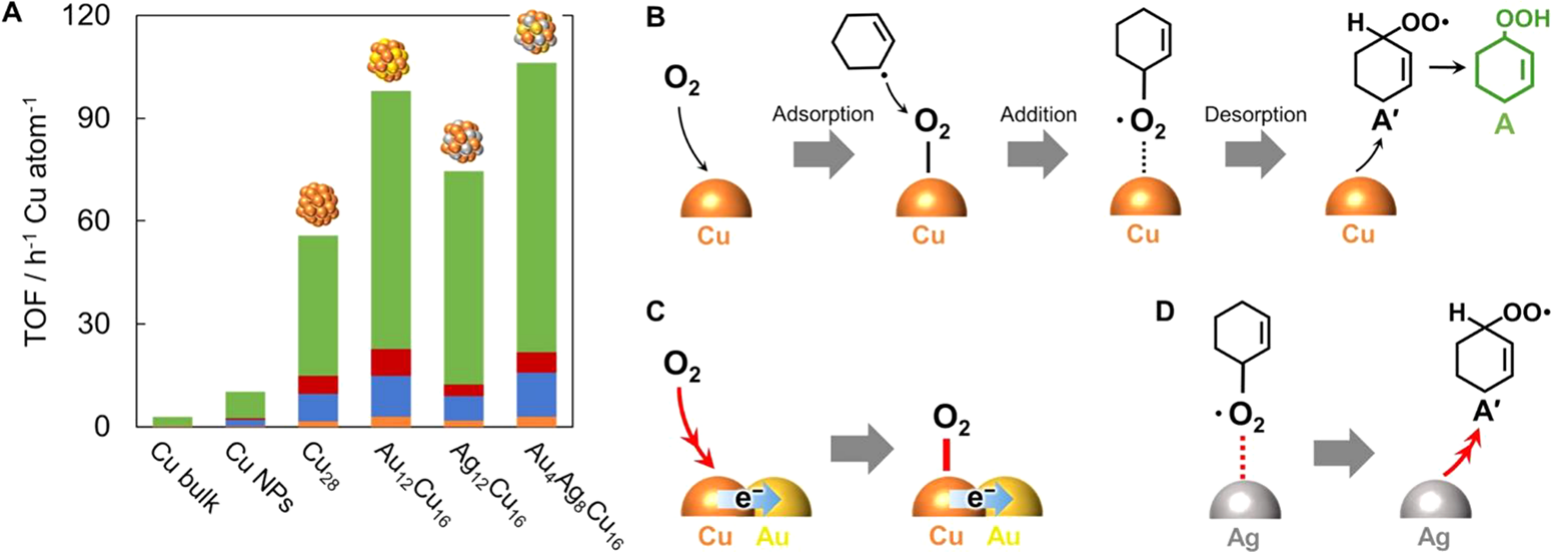
Bimetallic nanoclusters for oxidation of cyclohexene. (A) Turnover frequencies per Cu atom for a 1 h reaction of cyclohexene oxidation catalyzed by subnanometer clusters, nanoparticles, and bulk containing Cu. products 2-cyclohexenyl hydroperoxide (green), 2-cyclohexen-1-one (red), 2-cyclohexen-1-ol (blue), and 1,2-epoxycyclohexane (orange). (B) The proposed reaction mechanism on Cu atoms involves the adsorption of O_2_, the addition of cyclohexene radical, and the desorption of a cyclohexene peroxy radical. (C,D) Proposed reaction mechanisms promoted by the ligand effect in the Au–Cu alloy system and the ensemble effect in the Ag–Cu alloy system. Reproduced with permission from ref (359). Copyright 2020 Wiley-VCH.

For instance, the catalytic activity of Cu clusters is greatly promoted by doping of Au or Ag for oxidation of cyclohexene to 2-cyclohexenyl hydroperoxide (see Figure 35). The charge transfer from Cu to metals to Au is claimed as the reason for the enhanced reactivity of CuAu bimetallic clusters, while the desorption of the product (2-cyclohexenyl hydroperoxide) is facilitated at a higher degree than that on Au and Cu, leading to the promoted activity of CuAg clusters.^359^ The profound impacts of the addition of a single atom to a metal cluster on the catalytic performance of selective oxidation reaction is shown with the superior performances of Au_23_Pd_1_ clusters over monometallic Au_24_ clusters for oxidation of benzyl alcohol to benzoic acid, because the Pd atom serves as the active site for hydride elimination from the α-carbon of benzyl alcohol.^360^

It should be noted that none of the above-mentioned reported catalysts based on bimetallic clusters surpasses the classic supported Au nanoparticles for CO oxidation reaction, while in the case of oxidation of organic molecules, metal clusters are advantageous over the conventional nanoparticulate catalysts. One possible explanation could be the difference in the rate-determining step for the two reactions. For CO oxidation, CO adsorption and O_2_ activation are usually considered the critical steps, while for oxidation of hydrocarbon compounds, the C–H activation is generally considered as the rate-determining step.^361,362^ In this sense, it will be interesting to further explore the catalytic performance of Au-based and Pd-based bimetallic clusters for a variety of oxidation reactions, such as selective oxidation of propylene to propane oxide, oxidation of aromatics to diphenyl compounds, oxidation of thiophenols to disulfide compounds, etc., because these reactions have been reported to be catalyzed by the monometallic clusters/nanoparticles.^363−365^

### 6.3 Bimetallic Nanoclusters for Water–Gas Shift Reaction

In supported metal catalysts, the exposed facets of the support for hosting the metal particles will determine the structure of the metal–supporter interface, which is considered as major active sites for CO oxidation.^366^ The reactivity of bimetallic clusters is also dependent on the surface structure of the solid carrier. The PdCu clusters formed on the (100) facets of CeO_2_ are more active than those formed on (111) facets, which are proposed to be related to the smaller sizes of PdCu clusters generated on (100) facets and the higher number of oxygen vacancy sites.^367^ According to the study with Pd/CeO_2_ catalyst for CO oxidation, sintering of Pd atoms to nanoclusters or nanoparticles may occur due to the CO-induced migration of Pd atoms on the surface of CeO_2_.^368^ In this regard, more insights into the structural features of PdCu bimetallic nanoclusters could be obtained by performing *operando* characterizations.

**Figure 36:**
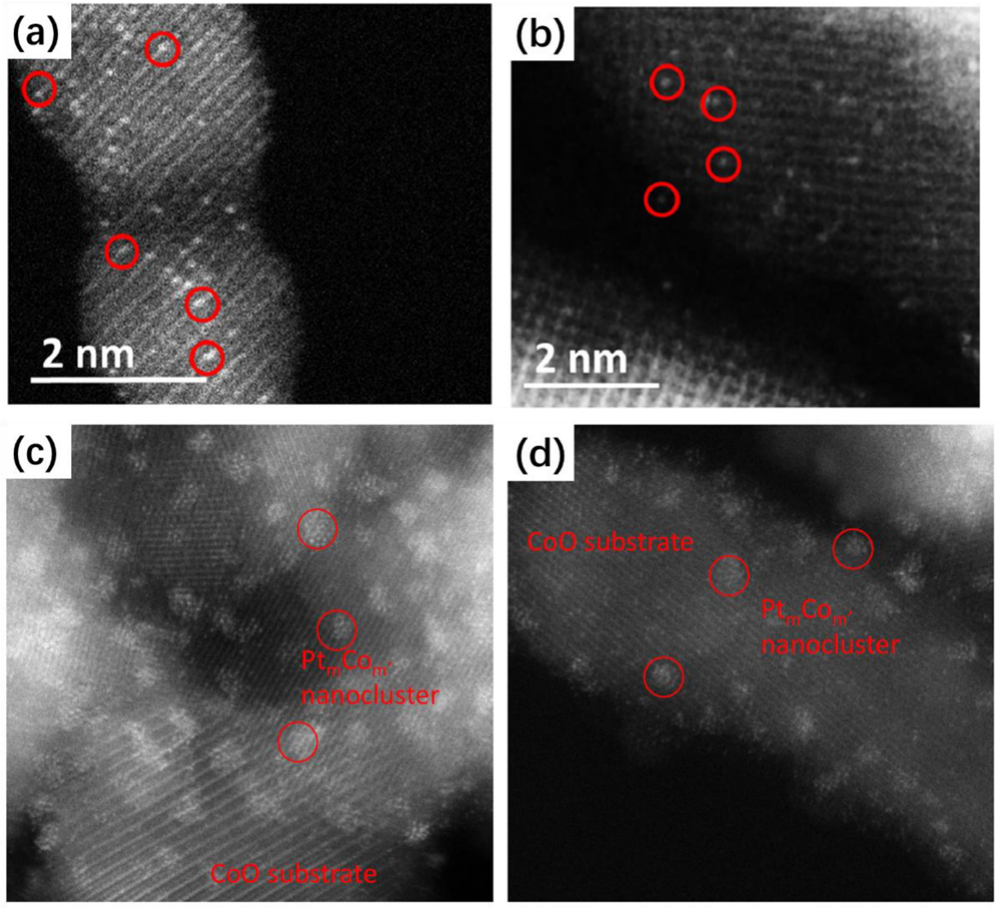
HAADF-STEM images of Pt species supported on Co_3_O_4_. (a,b) The fresh catalyst comprises isolated Pt atoms supported on Co_3_O_4_ nanorods. (c,d) Bimetallic PtCo clusters formed on Co_3_O_4_ nanorods formed from the isolated Pt atoms on Co_3_O_4_. Reproduced with permission from ref (109). Copyright 2013 American Chemical Society.

Indeed, the structural transformation of Pt atoms under water–gas shift reaction conditions has been observed with Pt supported on Co_3_O_4_. As shown in Figure 36a,b, isolated Pt atoms supported by Co_3_O_4_ remain to be atomically dispersed after exposure to water–gas shift reaction mixture up to 200 °C, but PtCo nanoclusters are formed at higher temperatures (≥280 °C) due to the sintering of Pt atoms and partial reduction of the support (see Figure 36c,d).^109^ More interestingly, the PtCo nanoclusters generated under reaction conditions are more active than the pristine Pt atoms (as indicated by lower apparent activation energy and higher TOFs), showing the advantages of bimetallic nanoclusters over isolated metal atoms.

### 6.4 Bimetallic Nanoclusters for Hydrogenation Reactions

Hydrogenation reactions are one of the major applications of supported bimetallic catalysts in practical processes, which are mostly achieved with bimetallic nanoparticles in current industrial processes.^369^ By replacing nanoparticles with nanoclusters, the utilization efficiency of metals can improve significantly and the bimetallic nanoclusters may show catalytic reactivities different to nanoparticulate counterparts.

According to theoretical calculations, a series of models of Co-based bimetallic nanoclusters with the configuration of M_1_-Co*_n_* (M = transition metals) are constructed to study how the composition of bimetallic clusters can influence the activation of N_2_.^370,371^ The metal d orbital electrons and the intrinsic first ionization energy are used to describe the electronic features of the bimetallic clusters, which are further correlated with the adsorption energy of N_2_. The calculation results suggest that the PdCo_4_ configuration is a promising candidate for ammonia synthesis reaction because of its charge buffer capacity of Pd atom and the complementary role of Co in the activation of N≡N bond and the subsequent hydrogenation steps. In terms of the experimental works, the superior performances of RuCo bimetallic nanoclusters and nanoparticles show promising performances over other Ru-based and Co-based catalysts for ammonia synthesis.^372,373^ There are discrepancies between the theoretical modeling and experimental studies that could be caused by the different particle sizes of the bimetallic entities and the support.

Taking mesoporous silica as the support, bimetallic nanoclusters can be generated and then stabilized on the solid carrier via the decomposition of the organometallic precursors, allowing the formation of bimetallic nanoclusters with narrower size distributions and more uniform compositions than the conventional coimpregnation method.^374,375^ For instance, Cu_4_Ru_12_C_2_ clusters are prepared by using (Ru_6_C(CO)_16_Cu_2_Cl)_2_(PPN)_2_ (PPN: bis(triphenylphosphanyl)iminium) as the precursor and mesoporous silica (MCM-41) as the porous support for hosting the clusters.^376^ The resultant bimetallic RuCu nanoclusters show good performance for selective hydrogenation of 1,5,9-cyclododecatriene to cyclododecene.

**Figure 37:**
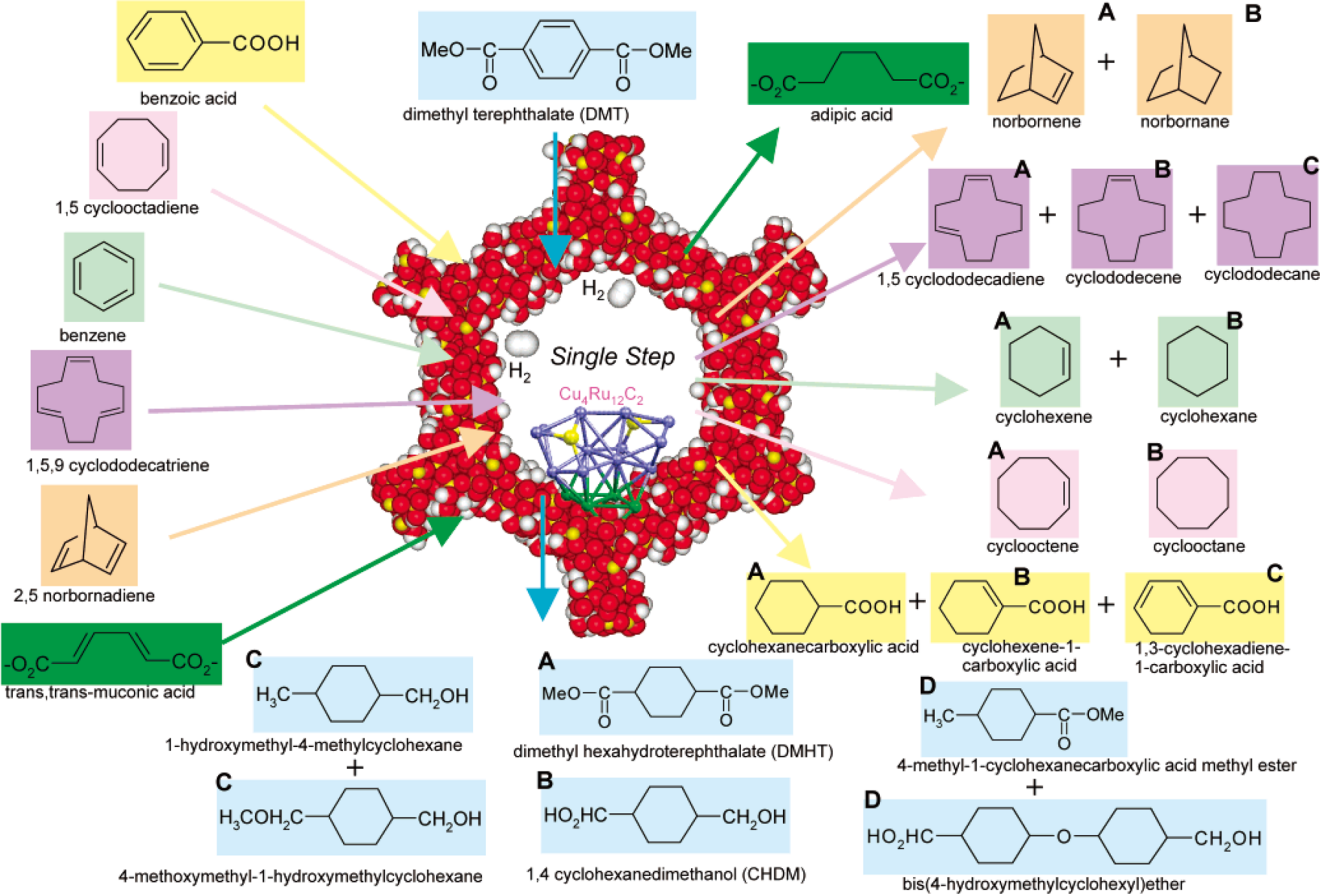
Single-step hydrogenation of some key organic compounds using highly active and selective anchored, bimetallic nanoparticle catalysts (Cu_4_Ru_12_C_2_, in this case). Note: A, B, C, and D correspond to the products as listed in Table 1 (under “product distribution”). Reproduced with permission from ref (374). Copyright 2003 American Chemical Society.

Both the activity and selectivity of the bimetallic nanoclusters can be modulated by tuning the composition as summarized in Figure 37 and Table 1. For example, the Pd_6_Ru_6_ shows higher activity than the Ru_6_Sn clusters for hydrogenation of 1,5-cyclooctadiene but majorly gives the complete hydrogenation product, while the Ru_6_Sn gives exclusively the semihydrogenation product. Such effects have also been demonstrated in other reactions involving the hydrogenation of C=C and −COOCH_3_ groups. According to the lessons learned from the studies carried out with bimetallic nanoparticles, the adsorption properties of the intermediate products on nanoclusters are influenced by the electronic properties of the metal species, which are also directly associated with the composition. The catalytic consequences of hydrogenation reactions with bimetallic clusters can also be associated with the intrinsic hydrogenation capability of different elements. In the case of hydrogenation of benzoic acid, the Ru_10_Pt_2_ clusters show very high selectivity to cyclohexanecarboxylic acid, while the Cu_4_Ru_12_ give the formation of cyclohexene-1-carboxylic acid and 1,3-cyclohexadiene-1-carboxylic acid, which could be ascribed to the much higher hydrogenation capability of Pt than Cu, resulting in the formation of fully hydrogenated products on Ru_10_Pt_2_ clusters and the partially hydrogenated products on Cu_4_Ru_12_ clusters.

**Table 1:** Single-Step Hydrogenations of Cyclic Polyenes and Aromatics with Supported Bimetallic Clusters

							product distribution (mol %)			
bimetallic clusters	substrate	solvent	time (h)	temp (K)	conv (mol %)	TOF (h^–1^)	A	B	C	D
Pd_6_Ru_6_	1,5-cyclooctadiene		8	353	36.9	2012	15.7	84.5		
Ru_6_Sn					11.7	1980	100			
Cu_4_Ru_12_					11.5	690	70.4	29.2		
Ag_4_Ru_12_					9.0	465	57.3	42.5		

Pd_6_Ru_6_	1,5,9-cyclododecatriene		8	373	64.9	5350		11.7	88.5	
Ru_6_Sn					17.2	1940	17.2	82.4		

Pd_6_Ru_6_	2,5-norbornadiene		8	333	76.4	11176	24.7	75.1		
Ru_6_Sn					51.4	10210	88.6	11.3		

Ru_5_Pt_1_	benzene		6	353	26.2	2625	8.7	91.2		
Ru_10_Pt_2_					27.6	1790	24.9	75.2		
Pd_6_Ru_6_					58.7	3216		100		
Ru_6_Sn					17.5	953	11.7	88.1		
Cu_4_Ru_12_					11.7	480		100		

Ru_5_Pt_1_	dimethyl terephthalate	C_2_H_5_OH	4	373	7.5	155	58.7	33.5		6.9
Ru_10_Pt_2_					23.3	714	42.6	52.3		
Pd_6_Ru_6_			8	373	15.5	125	22.5	4.2		74.2
Ru_6_Sn					5.3	54	77.2		22.6	
Cu_4_Ru_12_			24	373	14.2	45	25.3	63.2		11.3

Ru_5_Pt_1_	benzoic acid	C_2_H_5_OH	24	373	61.2	167	86.5	13.3		
Ru_10_Pt_2_					78.5	317	99.5			
Pd_6_Ru_6_					44.5	126	61.5	39.2		
Ru_6_Sn					15.9	24	9.0	42.5	48.6	
Cu_4_Ru_12_					21.8	48		79.6	21.2	

In order to further clarify the detailed structural features of supported bimetallic clusters derived from organometallic complexes, the geometric and electronic features should be followed during the catalyst synthesis procedure and also tracked under reaction conditions. With the help of EXAFS, the coordination environment of the metal species can be elucidated, which suggests the rearrangement of the metal atoms in the precursor complexes due to the removal of ligands and formation of bonding between the metal atoms and the support.^377,378^ For instance, Pt_2_Ru_4_ clusters can be formed on Al_2_O_3_ after careful grafting and subsequent decomposition of the bimetallic PtRu complex on Al_2_O_3_, which show interesting catalytic behavior for hydrogenation of ethylene and hydrogenolysis of butane. Taking into account the limited sensitivity of EXAFS characterization, it will be necessary to combine the EXAFS characterization and the state-of-the-art STEM imaging technique to reveal the atomic structures of the bimetallic nanoclusters.

**Figure 38:**
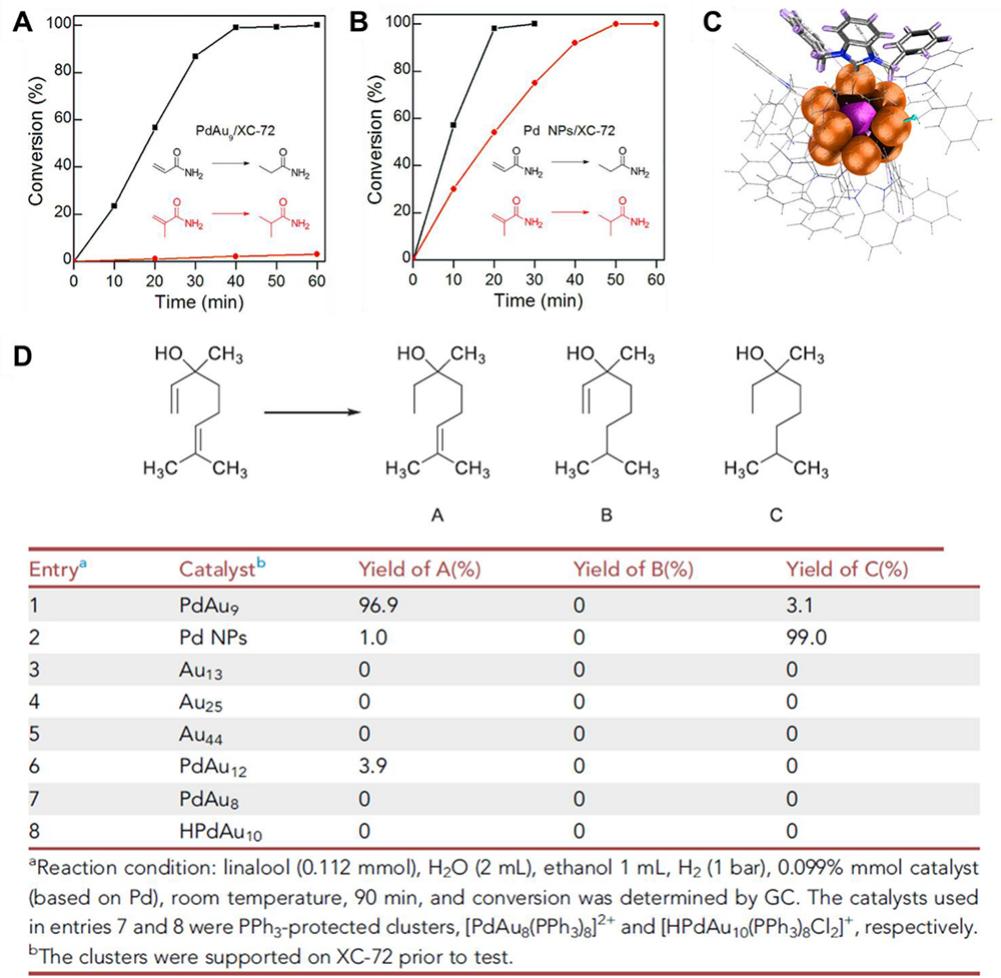
Shape-selective hydrogenation of C=C bonds with PdAu_9_ clusters stabilized by *N*-heterocyclic carbene ligand. (A,B) The catalytic performances of PdAu_9_ clusters and Pd nanoparticles for hydrogenation of acrylamide and methacrylamide. Due to the protection of the organic ligand, PdAu_9_ clusters can only catalyze the hydrogenation of acrylamide because the methacrylamide molecules cannot access the Pd sites. (C) Structure of the PdAu_9_ clusters stabilized by *N*-heterocyclic carbene ligand. The isolated Pt sites are accessible to small molecules. (D) Catalytic performances of different metal catalysts for selective hydrogenation of linalool. The PdAu_9_ clusters are selective for the hydrogenation of the terminal C=C bonds while the Pd nanoparticles give the fully hydrogenated product. Reproduced with permission from ref (380). Copyright 2022 Elsevier.

Currently, the wet-chemistry approach for the synthesis of ligand-protected bimetallic clusters is the most practical and facile way to obtain bimetallic clusters with uniform and well-defined structures. The presence of ligands will block some of the metal sites on the surface and then hinder the catalytic reactions, although the ligands can modulate the product distributions of some hydrogenation reactions.^379^ By choosing the appropriate organic ligand, there could be some free surface sites in the ligand-protected bimetallic nanoclusters, which are available for hydrogenation reactions. By tuning the coordination environment of the bimetallic clusters, the accessibility of the metal sites can be modulated, resulting in shape-selectivity in hydrogenation reactions. As shown in Figure 38, the isolated Pd atom in PdAu_9_ clusters stabilized by *N*-heterocyclic carbene ligand can only be accessed by small alkene molecules and the ligand-protected PdAu_9_ clusters show interesting shape-selective behavior for hydrogenation of the C=C bonds in organic molecules.^380^ Well-defined bimetallic clusters derived from ligand-assisted synthesis are excellent model systems to identify the active sites in the bimetallic catalysts.^381^ However, as mentioned before in the section on synthesis of bimetallic nanoclusters, the stability of the ligand-protected metal clusters, especially their structural integrity during the removal of the ligands is the critical issues that limit their applications in practical applications.

### 6.5 Bimetallic Nanoclusters for Dehydrogenation Reactions

The dehydrogenation of light alkanes in industrial processes relies on the use of supported Pt particles as catalysts. By decreasing the size of Pt particles to subnanometer Pt clusters, the utilization efficiency and intrinsic activity can be greatly improved. Owing to the presence of a large number of undercoordinated Pt sites on the surface of Pt clusters, the addition of Sn can not only improve the stability of Pt clusters against sintering but also suppress the coke deposition on Pt sites due to the modulation of the bonding between Pt and alkane molecules.^382,383^ According to the theoretical calculations, the bonding of alkene (primary product of the alkane dehydrogenation reaction) on the Pt_4_ cluster shows both di-σ- and π-bonded configurations, while the Pt_4_Sn_3_ cluster binds ethylene only in the π configuration. Thus, the further dehydrogenation of alkene is inhibited on bimetallic PtSn clusters, resulting in less coke formation.

**Figure 39:**
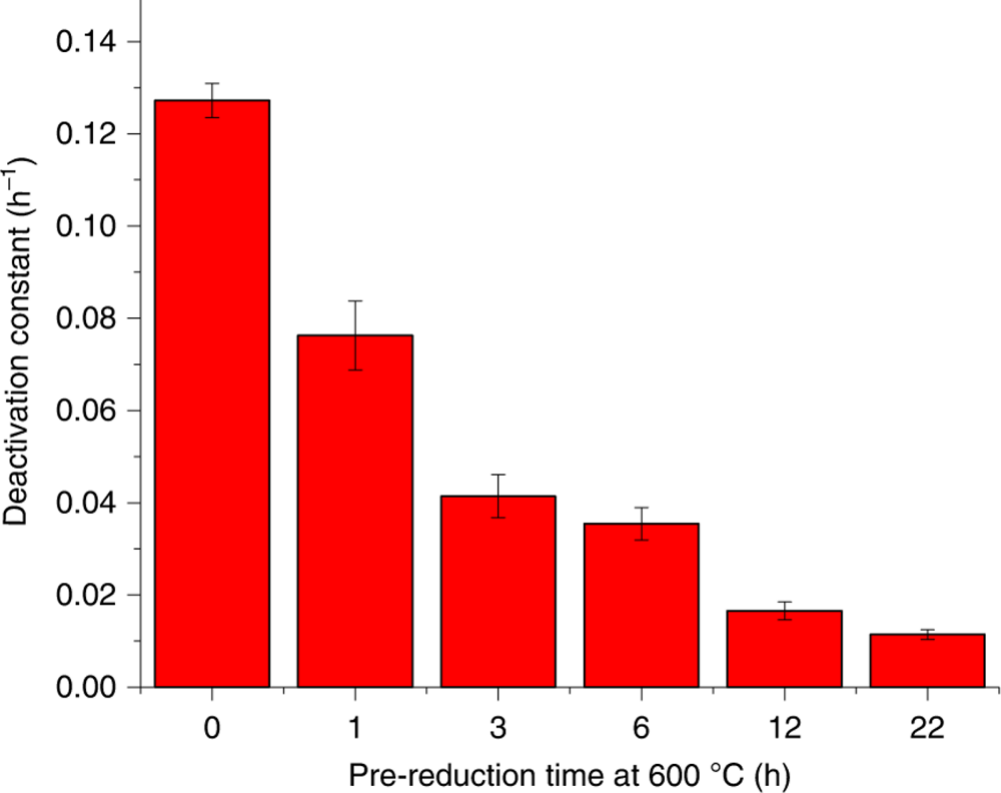
Catalytic performance for the propane dehydrogenation reaction. Reaction conditions: 5 mL min^–1^ propane, 16.5 mL min^–1^ N_2_, 600 °C, 20 mg K-PtSn@MFI-600H_2_ catalyst. The catalyst was reduced in a flow of H_2_ of 35 mL min^–1^ at 600 °C for different times before the dehydrogenation reaction tests. Deactivation constants for the various K-PtSn@MFI catalysts during the propane dehydrogenation reaction at 600 °C. With a longer prereduction treatment, the deactivation of the PtSn bimetallic clusters becomes slower. Reproduced with permission from ref (16). Copyright 2020 Springer Nature Limited.

By confining subnanometer PtSn clusters in zeolites, their stabilization against sintering is greatly improved. More importantly, the bimetallic PtSn clusters confined in the channels of pure silica MFI zeolite show remarkably higher activity than conventional PtSn nanoparticles (>2 nm) for the dehydrogenation of propane to propylene.^384^ The geometric and electronic features of PtSn clusters can be modulated by the pretreatments before the catalytic tests. As shown in Figure 39, with longer prereduction time and more intimate contact between Pt and Sn, is formed as visualized by the quasi in situ STEM measurements.^16^ Consequently, the initial selectivity to propylene is improved, and thanks to the suppressed breaking of C—C bonds the deactivation of the catalyst is also alleviated because of the less coke formation. The advantages of using zeolite as the support for stabilizing Pt-based bimetallic nanoclusters have been demonstrated with other Pt-based bimetallic nanoclusters and in other zeolites besides MFI-type structure.^104,105,385,386^ The morphology of the zeolite support and the location of the promotor metals have significant influences on the particle size distributions and stability of the Pt species, inferring the importance to precisely control the structures of metal-zeolite catalysts, especially the location and the anchoring sites of the metal species in the microporous structure.

Nevertheless, the chemical composition of the bimetallic nanoclusters derived from the combination of a noble metal (such as Pt, Pd, Rh, etc.) and a non-noble metal (such as Sn, Zn, In, etc.) could be further optimized for achieving high utilization efficiency of noble metals. For instance, by planting isolated Pt sites in the Zn/ZSM-5 support, it is claimed that PtZn_*n*_ clusters with one Pt atom surrounded by several Zn atoms are generated in the silanol nests of ZSM-5 and the resultant PtZn*_n_* clusters can efficiently catalyze the dehydroaromatization of ethane due to the synergy between the bimetallic nanoclusters and the acid sites in ZSM-5.^387^ The concept should be extended to other combination of noble and non-noble metals for the preparation of subnanometer bimetallic clusters with low loadings of noble metals but high activities for conversion of light alkanes to olefins and aromatics.

Introducing a small amount of oxidant into the reaction feed can shift the thermodynamic equilibrium by consuming the H_2_, which is called oxidative dehydrogenation of alkanes to alkenes. Bimetallic nanoclusters such as PtSn and PdCu prepared by size-selected method show promising activity for oxidative dehydrogenation of propane to propylene.^388,389^ It should be noted that these small clusters may suffer irreversible structural transformation under the reaction conditions. The presence of oxidant (O_2_) and reactant (alkane) may cause more severe sintering of the metal species in comparison to the reductive atmosphere used in the direct dehydrogenation of propane to propylene. Indeed, the sintering of small PtSn clusters normally occurs during the high-temperature reaction–regeneration cycles because the Pt species have a stronger tendency for migration on the solid carrier than in a reductive atmosphere as a consequence of the formation of volatile PtO_2_.^17,390,391^

To constrain the mobility of Pt species under the reaction conditions for oxidative dehydrogenation of alkanes, the interaction between Pt and the support should be promoted. The choice of CeO_2_ as the support for Pt-based alloy nanoparticles shows that not only the sintering of Pt species but also the coke deposition is greatly suppressed, which is attributed to the strong interaction between Pt and CeO_2_ and the oxygen releasing ability of the CeO_2_ support for the removal of the coke.^392^ Following this strategy, it will be interesting to decrease the size of the Pt-based alloy nanoparticles down to the subnanometer regime to form Pt-based bimetallic or even multimetallic nanoclusters, which could be even more active than the nanoparticulate counterparts.

Beside light alkanes, long-chain alkanes such as C6–C8 alkanes can also be subjected to the dehydrogenation reaction, which will lead to the production of aromatics via the dehydroaromatization process. In the industrial naphtha reforming process, bimetallic Pt–Re catalyst supported on Al_2_O_3_ can effectively convert the hydrocarbon mixture of linear alkanes and cyclic alkanes into aromatics.^393^ Fundamental studies implied that the active sites are probably tiny PtRe nanoclusters with low atomicity (less than 10 atoms). Due to the very broad application of PtRe/Al_2_O_3_ catalysts in petrochemical industry, the catalyst’s structures and catalytic properties have been intensively studied decades ago, as reflected in the prior literature.^394^ However, limited by the resolution/sensitivity of the characterization tools, the atomic structures of the PtRe bimetallic species are not fully revealed yet.^395^ In our opinion, it will be of great interest to employ the newly developed electron microscopy and spectroscopy techniques to revisit the structures of the industrial PtRe/Al_2_O_3_ catalysts or even the optimized PtReSn/Al_2_O_3_, which may bring new insights/inspirations for designing advanced catalysts for hydrocarbon processing.^396,397^

The industrial dehydroaromatization process for converting *n*-hexane into benzene relies on the use of Pt/KL catalyst, which comprises Pt nanoclusters in the 12MR channel of KL zeolite.^398^ The introduction of a cocatalyst metal (such as Fe, Co, etc.) into Pt/KL catalyst to form Pt-based bimetallic nanoclusters are reported to be effective to alleviate the catalyst deactivation and suppress the side reactions in dehydroaromatization of *n*-heptane and *n*-octane.^399,400^ Taking into account the works of zeolite-confined metal clusters for dehydrogenation of light alkanes, further exploration of other Pt-based bimetallic nanoclusters (such as PtSn, PtGe nanoclusters, etc.) and precise control of the composition and location of the nanoclusters in KL zeolite structure may lead to the generation of advanced catalysts for dehydroaromatization reactions.

### 6.6 Bimetallic Nanoclusters for Organic Reactions

For numerous organic reactions, the combination of two different metals may show much higher performances than the monometallic catalyst due to the synergy of the two metals for activating the reactants and completing the catalytic cycle.^234^ Bimetallic nanoclusters based on organometallic complexes show interesting catalytic properties for various organic reactions. For instance, VIII group metals are widely used in carbonylation reactions. Specially, bimetallic nanoclusters based on VIII group metals (such as PdCo, RhCo clusters) are active for homologation of methanol for production of Me_2_O, AcOH, AcOMe, and MeCH(OMe)_2_.^401,402^ The activity and product distribution are greatly dependent on the composition of the bimetallic clusters, which could be caused by the impacts of the clusters’ structural features on the activation of methanol and the C–C coupling step.

**Figure 40:**
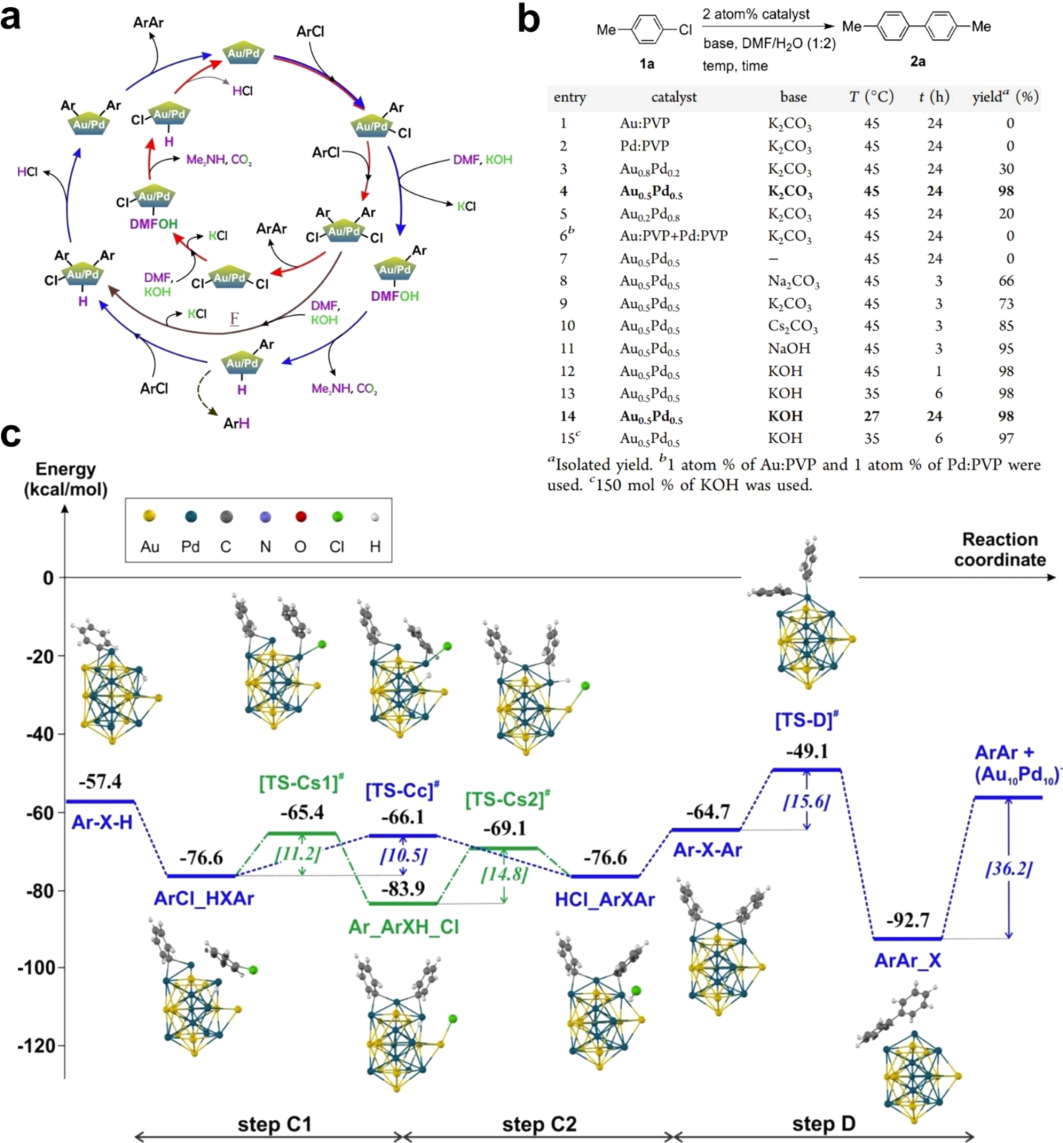
Bimetallic AuPd nanoclusters for Ullmann coupling reaction. (a) Proposed two reaction pathways for Ullmann coupling of ArCl on AuPd nanoclusters: pathway I (outer blue cycle) and pathway II (inner red cycle). (b) Catalytic results of Ullman coupling of 4-chlorotoluene with different metal catalysts. Bimetallic AuPd nanoclusters stabilized by poly(*N*-vinylpyrrolidone) (PVP) show excellent yield, while the monometallic catalysts are inactive. (c) Energy profile for the oxidative addition of the second ArCl to the reductive elimination of biphenyl in the pathway I. (a,c) Reproduced with permission from ref (404). Copyright 2019 Wiley-VCH. (b) Reproduced with permission from ref (403). Copyright 2012 American Chemical Society.

In addition to the bimetallic cluster catalysts derived from the organometallic approach, bimetallic nanoclusters prepared by wet-chemistry methods. For instance, AuPd nanoclusters stabilized by poly(*N*-vinylpyrrolidone) (PVP) are shown to be efficient catalysts for Ullmann coupling reaction while the monometallic clusters are inactive (see Figure 40).^403^ Mechanistic study based on theoretical calculations indicate that the Pd ensembles in the AuPd nanoclusters are responsible for the oxidative addition of Ar–Cl) and the role of Au is to improve the stability of the Pd ensembles.^404^ The synergy of bimetallic nanoclusters for organic reactions is observed with other reactions, such as hydrosilylation of internal alkynes.^405^ It should be noted that the AuPd nanoclusters stabilized by PVP basically show disordered structures with random distribution of Au and Pd. In order to gain further insights on the atomic structures of the active sites in bimetallic nanoclusters, the ligand-protected bimetallic nanoclusters with well-defined structures can be employed as the model catalysts for elucidating the optimum local structures of the Pd sites.

It has been shown with a variety of organic reactions that metal clusters made by a few atoms serve as the real active sites, which are formed under reaction conditions from the metal precursors.^227,406−408^ The metal clusters could be formed due to the interaction between the precursors (in the form of molecular complex or metal nanoparticles) and the reactants/solvents/ligands. In this sense, beside the direct use of bimetallic nanoclusters as the catalysts for organic reactions, the bimetallic clusters may also be formed during the organic transformations catalyzed by two metals. For instance, Pd and Cu are usually employed together for Sonagashira reaction to construct C–C bonds, and the critical role of Pd clusters for C–C coupling reaction has been verified by numerous studies.^409,410^ Bimetallic PdCu clusters could be produced, which need to be carefully verified by electron microscopy and spectroscopy techniques.

In the above examples, the bimetallic nanoclusters are either coordinated by ligands or polymers, which limits their stability and recyclability for practical applications. In principle, by immobilizing bimetallic nanoclusters on solid carrier should effectively improve the stability of the nanoclusters, although their catalytic performances may be affected by the support. For instance, nitrogen-rich mesoporous carbon is shown to be a superior support for hosting AuPd nanoclusters as the catalysts for Ullmann coupling reaction.^411^ In the future works, it will be interesting to explore the suitable support for bimetallic nanoclusters for the target organic reactions and develop appropriate methodologies for stabilizing the nanoclusters on the supports.

### 6.7 Bimetallic Nanoclusters for Photocatalytic Reactions

**Figure 41:**
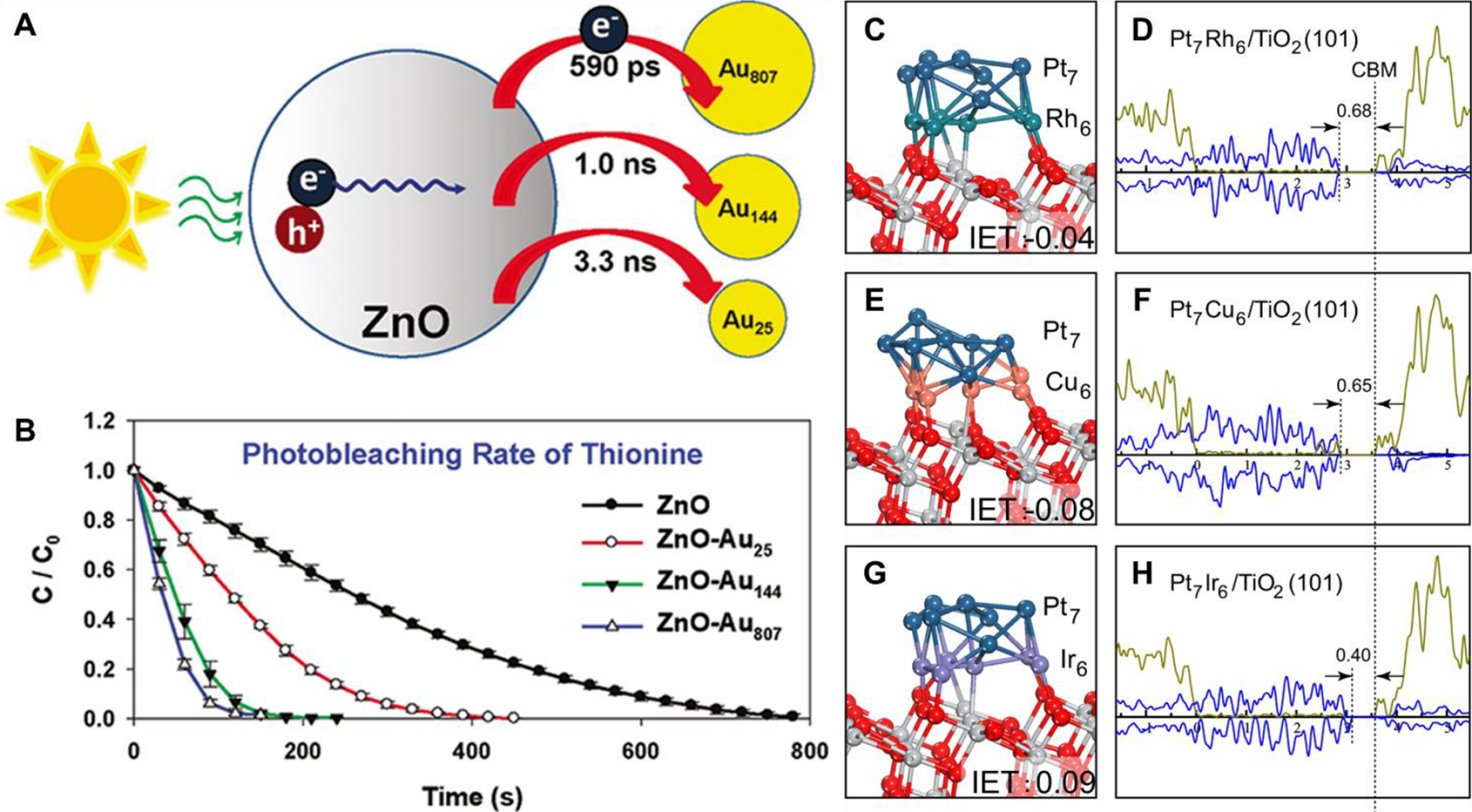
(A) Schematic illustration of charge-transfer between ZnO nanoparticle and Au particles with different sizes. The kinetic curves of photocatalytic degradation of thionine (a model dye molecule) is also presented to show the kinetic of the recombination of photogenerated electrons and holes in ZnO nanoparticles. (B) With faster charge-transfer process between Au particles and ZnO nanoparticles, the photocatalytic degradation of thionine will be faster. (C–H) Calculated structures, IET energies, and density of states for bimetallic nanoclusters supported on TiO_2_. (C,D) Pt_7_Rh_6_ cluster on TiO_2_(101) surface, (E,F) Pt_7_Cu_6_ cluster on TiO_2_(101) surface, and (G,H) Pt_7_Ir_6_ cluster on TiO_2_(101) surface. The valence band edge of TiO_2_ is uniformly aligned to zero in DOS, and the vertical dot lines indicate the TiO_2_ CBM and the *E*_f_ position, respectively. Calculated IET energies are also given in eV. (A,B) Reproduced with permission from ref (414). Copyright 2011 American Chemical Society. (C–H) Reproduced with permission from ref (415). Copyright 2021 Springer Nature under CC-BY license (https://creativecommons.org/licenses/by/4.0/).

In photocatalytic systems, bimetallic nanoclusters are usually employed as cocatalysts to facilitate the surface reactions of the photogenerated charge carriers with the substrate reactants. At the interface of metal–semiconductor interface, an electronic equilibrium will be established once the contact of metal and semiconductor is formed.^412^ Because the electronic structures of the semiconductor supports are normally fixed, the electronic structures of the metal–semiconductor junction will be largely determined by the bimetallic entities.^413^ As displayed in Figure 41, prior works on monometallic clusters show that the charge-transfer rates between ZnO nanoparticles and Au particles increase when increasing the size of Au particles from ca. 1.0 nm (Au_25_) to ca. 3.5 nm (Au_807_).^414^ Taking into account that the electronic structures of bimetallic nanoclusters depend on both the particle and chemical composition, there will be a larger variation range for the charge-transfer rates between the bimetallic nanoclusters and semiconductor support than that with the monometallic counterparts. Indeed, according to the theoretical calculations, the charge transfer between Pt_13_ cluster and TiO_2_ can be markedly improved by introducing Rh or Cu into the Pt_13_ clusters to form Pt_7_Rh_6_ and Pt_7_Cu_6_ clusters with bilayer structures as cocatalysts for photocatalytic H_2_ evolution reactions.^415^ The theoretical study infer that the first-layer metal atoms, which are located at the interface between bimetallic clusters and TiO_2_, are responsible for extracting the photogenerated elections from the TiO_2_ and then transfer the electrons to the second-layer metal atoms for H_2_ evolution reaction.

In a typical experimental work, bimetallic Au_24_Pt and Au_24_Pd clusters are loaded on BaLa_4_Ti_4_O_15_ as the cocatalyst for photocatalytic water splitting.^416^ The catalytic tests show that the introduction of Pd into Au clusters does not improve the photocatalytic activity but Pt can promote the performance. Interestingly, structural characterizations indicate that Pt is selectively located at the interface between Au_24_Pt cluster and the BaLa_4_Ti_4_O_15_ semiconductor, while Pd is located on the external surface of Au_24_Pd cluster. The structural difference affects the transfer of photogenerated electrons to the metal clusters and thereby influences the efficiency for photocatalytic water splitting. Combining the above-mentioned theoretical and experimental works, we can conclude that modulating the composition of the bimetallic nanoclusters, and the spatial distribution of the metal elements is critical for tuning their photocatalytic behavior.

**Figure 42:**
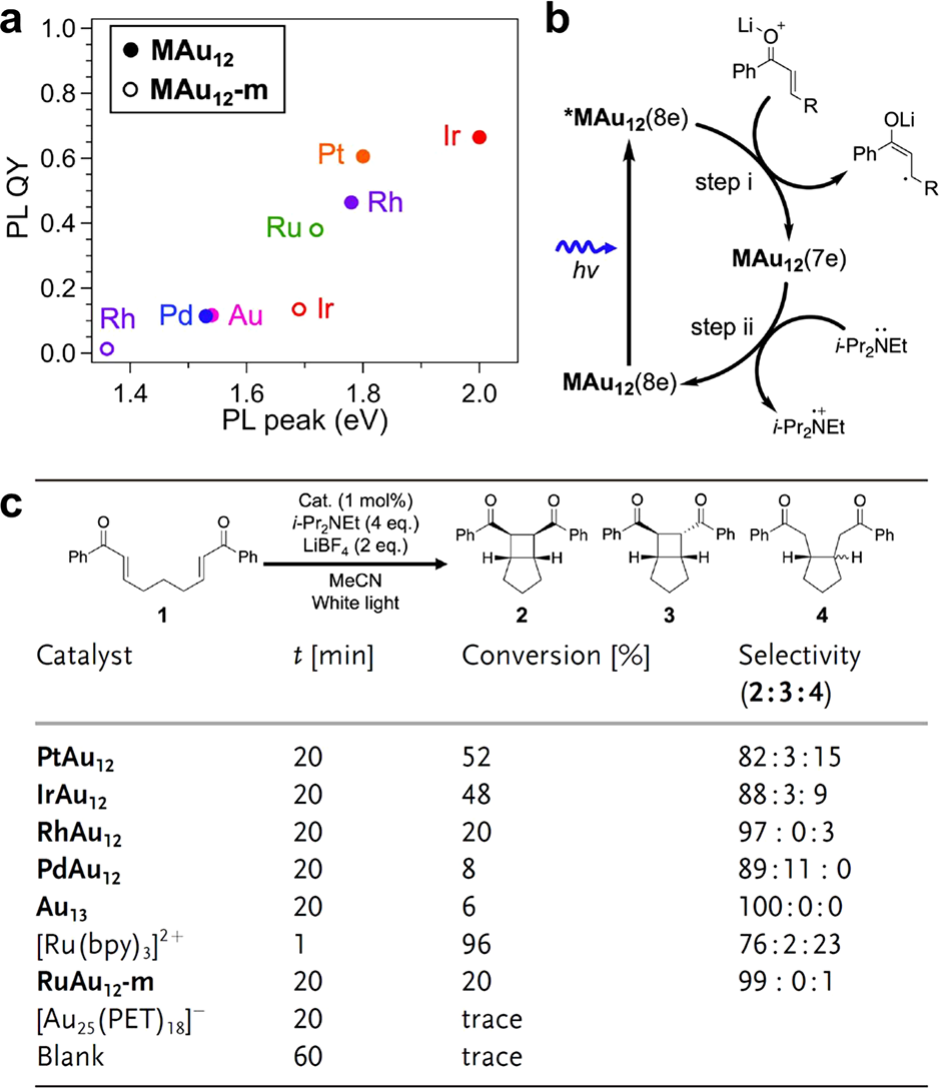
Bimetallic nanoclusters for intramolecular [2 + 2] cycloaddition of bisenone via the oxidative quenching cycle. (a) Plot of the PL QYs of MAu_12_ and MAu_12_-m versus their energy of their PL peak. (b) Proposed photoredox catalytic cycle for MAu_12_. Steps i and ii correspond to the reduction of 1 by *MAu_12_(8e) and the oxidation of *i*-Pr_2_NEt by MAu_12_(7e), respectively. (c) Photoredox catalysis of [2 + 2] bisenone cycloaddition with MAu_12_, [Ru(bpy)_3_]^2+^, RuAu_12_-m, and [Au_25_(PET)_18_]^−^. Reproduced with permission from ref (417). Copyright 2022 Wiley-VCH.

Beyond working as cocatalyst, bimetallic clusters can serve as the photosensitizer for capturing photo energy and then transfer the photogenerated charge carrier (usually electrons) to the reactant for initiating the redox reaction. For example, by using Au-based bimetallic nanoclusters as the photocatalyst, intramolecular [2 + 2] cycloaddition of bisenone is achieved via the oxidative quenching cycle.^417^ By tuning the chemical composition of the bimetallic nanoclusters, the electronic properties are changed, as indicated by the photoluminescence spectra. Consequently, the activity and product distribution in photocatalytic cycloaddition reaction change remarkably with the catalyst’s composition, as summarized in Figure 42.

To improve the charge-transfer efficiency, a promising strategy is to incorporate the bimetallic nanoclusters in a porous matrix, such as metal–organic frameworks, in order to minimize the distance between the photosensitizer and the active sites for photoredox transformations. By trapping the metal clusters in MOF structure, the stability of the ligand-protected bimetallic clusters can be greatly improved.^418^ Moreover, the active sites, which are responsible for the photoredox transformations, can be installed on the node or linker position of the MOF structure, resulting in the formation of composition materials for photocatalytic organic transformations.^419^

### 6.8 Bimetallic Nanoclusters for Electrocatalytic Reactions

**Figure 43:**
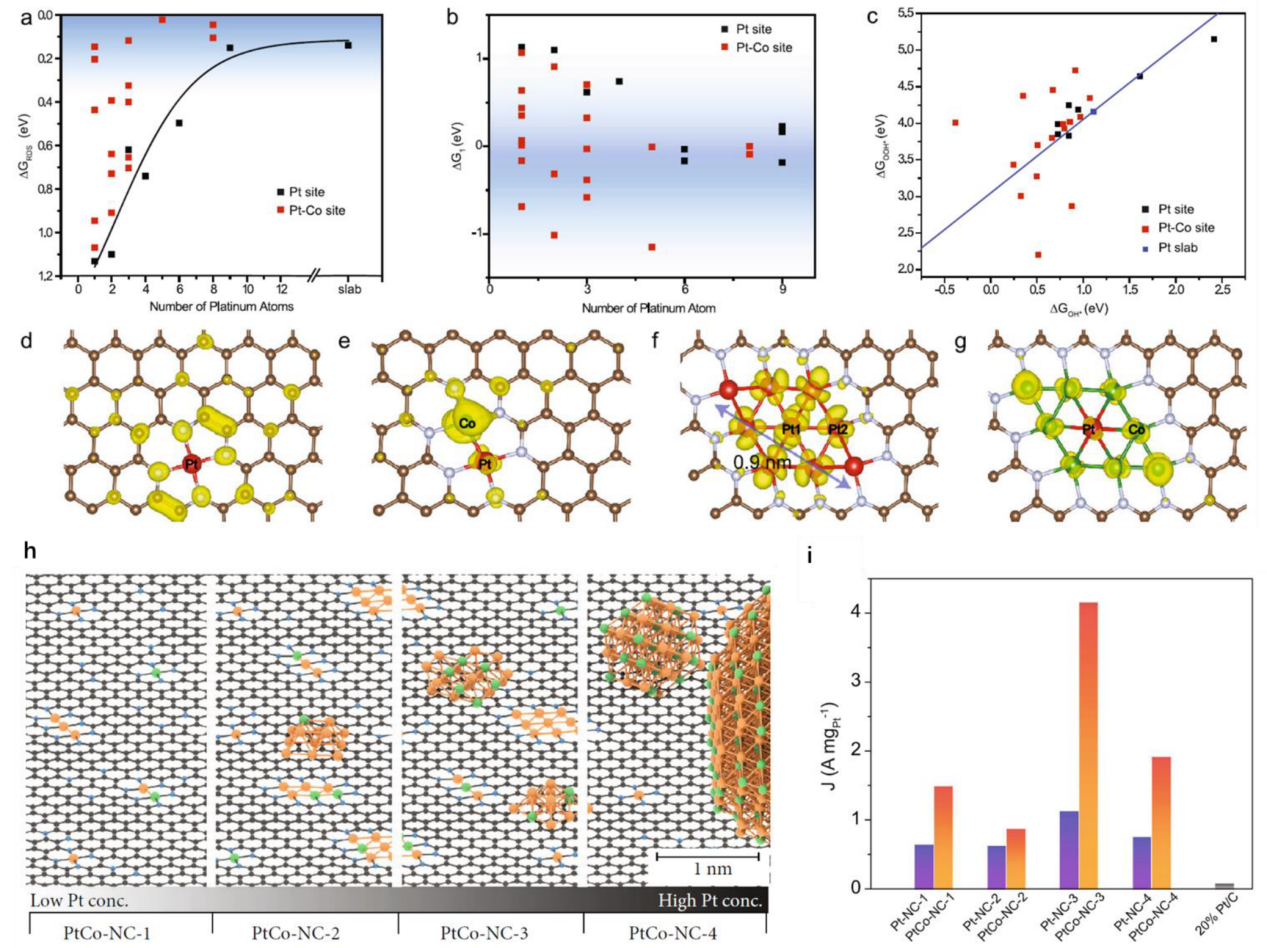
PtCo bimetallic clusters for electrocatalytic ORR. (a–c) Results of DFT calculations of Pt and PtCo clusters of different sizes embedded in a nitrogen-doped carbon matrix. The potential is set to +0.90 V vs RHE. (a) Gibbs free energy of the rate determine step (Δ*G*_RDS_) versus platinum clusters of different sizes (Pt_*x*_). (b) Gibbs free energy of the first-electron reduction (Δ*G*_1_) versus platinum clusters of different sizes (Pt_*x*_). The dark-blue regions in (a) and (b) indicate the range of optimal energy for ORR. (c) Correlation between adsorption free energy of OOH* and OH* intermediates on Pt sites on carbon (black squares), PtCo sites in carbon (red squares), and Pt surface (blue line). (d–g) Wave function module square of selected configurations of Pt and PtCo in carbon near the Fermi level with an isosurface value of 0.001 e/au^3^. (h) Schematic illustration of different types of supported PtCo catalysts on N-doped carbon. The loading of Pt increases gradually from the PtCo-NC-1 sample to the PtCo-NC-4 sample, which causes the growth of the sizes of the Pt species. (i) Electrocatalytic performance in oxygen reduction reaction. Mass activity of the samples and 20% Pt/C at +0.85 V vs RHE. The blue and yellow columns represent the Pt-NC and PtCo-NC samples, respectively. Reproduced with permission from ref (420). Copyright 2020 AAAS under CC-BY license (https://creativecommons.org/licenses/by/4.0/).

In the theoretical works on catalytic properties of bimetallic catalysts, models based on bimetallic clusters with a few atoms are usually constructed to represent the active sites in the catalysts made of bimetallic nanoclusters or nanoparticles. These theoretical studies provide rich knowledge of the influences of geometric and electronic structures on the interaction between the bimetallic nanoclusters and the reactants. For instance, taking the Co–N–C material with isolated Co sites in the N-doped carbon matrix as the support, models for different types of PtCo bimetallic sites are constructed to figure out the most active types of bimetallic sites for electrocatalytic ORR.^420^ As shown in Figure 43, the calculation results show that the activity of Pt clusters with low atomicity (with less than 8 atoms) increases dramatically after the introduction of Co (either doping in the Pt cluster or serving as the anchoring site of the Pt cluster. Guiding by the calculation results, the authors have prepared a series of bimetallic PtCo catalysts with different particle sizes by varying the Pt loading and the catalytic results indicate that Pt clusters with 2–7 Pt atoms and 2–3 Co atoms are the optimum configuration of PtCo clusters for electrocatalytic ORR. The excellent electrocatalytic activity of bimetallic PtCo clusters supported on Co–N–C support is also reflected in hydrogenation evolution reaction.^421^ The structure of the active sites is proposed to be partially oxidized Pt clusters are anchored on the isolated Co sites in the N-doped carbon matrix, due to the interaction between Pt clusters and CoN_4_ sites. The remarkably high activity of PtCo clusters supported on Co–N–C support has also been confirmed by the H_2_–O_2_ membrane electrode assemblies, in which the supported PtCo catalysts with low Pt loading but deliver high mass-transfer-limiting current densities and excellent power performances.^422^

Beside using Co–N–C as the support, bimetallic PtFe catalysts with superior activity for ORR have been prepared by loading a small amount of Pt species on the Fe–N–C support, which deliver excellent activity normalized to the mass of Pt. Detailed structural characterizations show that small bimetallic PtFe nanoparticles with sizes of ∼2 nm are stabilized on the Fe–N–C support, with greatly promoted durability.^423^ In principle, by optimizing the methods for materials synthesis, the size of the bimetallic PtFe nanoparticles may be decreased to subnanometer regime to form bimetallic PtFe nanoclusters, which may further promote the normalized Pt mass activity, or even the specific activity of Pt. Taking into account of the structural diversity of M–N-C materials (the types of M metals and the coordination environment of M metals in the N-doped carbon matrix), there are plenty room for improving the catalytic performances of PtM/M–N–C catalysts for fuel cell applications through optimizing the size of PtM clusters, the bonding between PtM clusters, and the M–N–C support.^424−426^

In additional to carbon-supported noble metal catalysts, bimetallic nanoclusters based on non-noble metals are also promising electrocatalysts. For instance, bimetallic CoFe nanoclusters stabilized in N-doped carbon prepared by the pyrolysis of the mixture of Co and Fe molecular precursors.^427^ The kinetic study indicates that bimetallic CoFe nanoclusters show enhanced rate over the monometallic Co and Fe clusters for binding O_2_. Nevertheless, the bimetallic CoFe nanoclusters also show good performance for electrocatalytic OER, which is ascribed to the formation of bimetallic CoFe oxyhydroxide species under the OER condition, although the detailed structural features of the oxyhydroxide species are not clearly revealed.^428^

Isolated metal sites and binuclear bimetallic sites embedded in N-doped carbon matrix have been reported as active sites for a variety of electrocatalytic reactions. In principle, bimetallic nanoclusters are highly likely to form during the preparation of binuclear bimetallic sites supported on N-doped carbon materials, especially through the pyrolysis method. In this regard, it is important to carry out comparative study on the catalytic properties of binuclear bimetallic sites and bimetallic nanoclusters for a given electrocatalytic reaction. A practical approach to clarify this issue is to prepare the materials by tuning the metal loadings, which will generate a set of bimetallic catalysts with different size distributions, which are suitable for performing kinetic studies.

As discussed in the section of hydrogenation reactions with bimetallic nanoclusters, ligand-protected metal clusters with well-defined structures are excellent model systems to establish the structure–reactivity of bimetallic nanoclusters in electrocatalytic reactions.^429^ As shown in a series of works, the addition of a single metal atom into a metal cluster to form bimetallic clusters will induce remarkable changes in electrocatalytic performances, as proved with Pt-doped Au clusters for electrocatalytic H_2_ evolution,^430,431^ Pt-doped Cu clusters,^432^ and Pd-doped Au clusters for CO_2_ reduction.^433^ For metal clusters with low atomicity, the introduction of a single atom can cause marked changes in the electronic structure, resulting in modification of the electronic structures. For instance, the replacement of one Au with one Pt atom in the Au_38_(SCH_2_Ph*t*Bu)_24_ cluster elevates the relative energy of the highest occupied molecular orbital (HOMO) accompanied by one valence electron loss of Pt_1_Au_37_(SCH_2_Ph*t*Bu)_24_, in comparison with Au_38_(SCH_2_Ph*t*Bu)_24_ with 14 electrons. As a result, the electrocatalytic reduction of CO_2_ to CO is increased. Interestingly, if two Pt atoms are doped into the Au clusters, the resultant Pt_2_Au_36_(SCH_2_Ph*t*Bu)_24_ clusters show declined activity due to the narrowed HOMO–LUMO (lowest unoccupied molecular orbital) gap. The profound influences of the chemical compositions of bimetallic nanoclusters are found with other systems, such as the Au-based and Ag-based clusters for electrocatalytic N_2_ reduction and CO_2_ reduction.^434−437^

**Figure 44:**
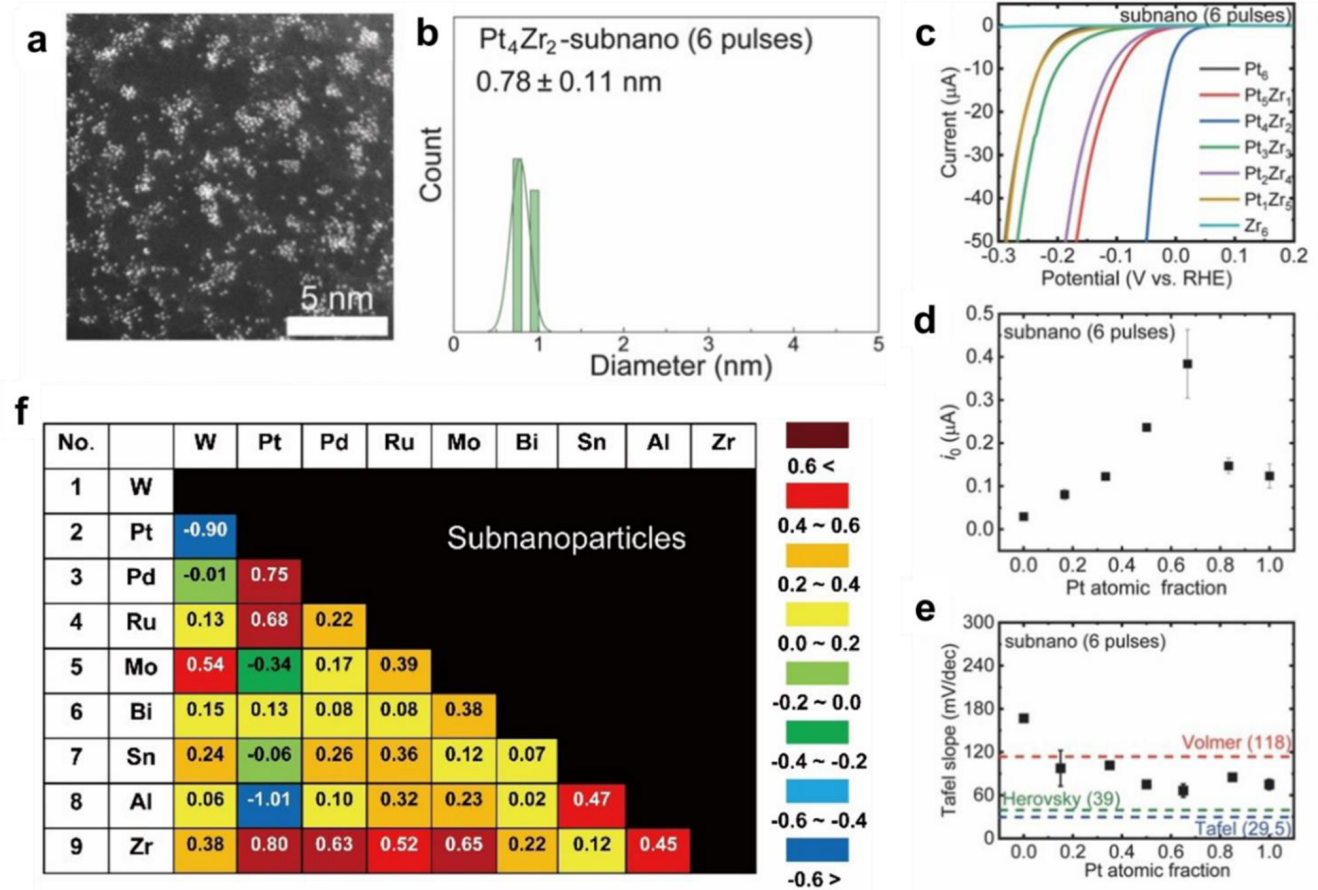
Characterization of PtZr alloy particles prepared from the different number of pulses. (a) ADF-STEM images and (b) size distribution histograms of PtZr bimetallic nanoclusters. (c–e) Comparison of electrocatalytic activity of PtZr bimetallic nanoclusters with different Pt/Zr ratios for the hydrogen evolution reaction (HER). (c) Original linear sweep voltammogram (LSV) curves of PtZr nanoclusters in the aqueous argon-saturated electrolyte (0.05 M H_2_SO_4_). (d) Exchange currents (*i*_0_) of HER derived by the Tafel plots. (e) Tafel slope values of HER. (f) Synergistic effect indexes of various alloy particles for the electrocatalytic HER. HER synergistic index (HSI) was defined by the following formula: HIS = (2*i*_0_(M^1^_*x*_M^2^_*y*_) – *i*_0_(M^1^_*x*_) – *i*_0_(M^2^_*y*_))/2*i*_0_(M^1^_*x*_M^2^_*y*_)), where *i*_0_(M^1^), *i*_0_(M^2^), and *i*_0_(M^1^_*x*_M^2^_*y*_) represent the HER exchange current of single component M^1^, single component M^2^, and bimetallic M^1^_*x*_M^2^_*y*_ (*x* and *y* represent concentrations of M^1^ and M^2^, respectively). Reproduced with permission from ref (441). Copyright 2022 Wiley-VCH.

In order to clarify the impacts of the chemical composition of bimetallic nanoclusters in catalytic reactions, the comparative study should be performed on a series of model catalysts derived from the same sample preparation method. Following this concept, bimetallic PtZr nanoclusters with different Pt/Zr ratios, and particle sizes are generated by controlling the pulses of arcplasma deposition and then tested for electrocatalytic HER.^438^ As shown in Figure 44, the Pt_4_Zr_2_ sample (prepared by four pluses of Pt and two pulse of Zr deposition) comprising PtZr nanoclusters of ∼0.8 nm delivers the best activity. The significant promotion effect of Zr to Pt for electrocatalytic HER can be explained by the modification of the electronic feature of Pt, which weakens the adsorption of Pt–H species. Furthermore, the arcplasma deposition method allows to prepare various types of bimetallic nanoclusters by simply change the metal precursors, enabling the generation of a library of bimetallic nanoclusters for rapid screening. As summarized in Figure 44, synergistic effect indexes of various alloy particles for the electrocatalytic HER are calculated to determine whether the combination of two metal elements in a bimetallic nanocluster is beneficial for HER. Some the results shown in the table are consistent with the literature works on bimetallic catalysts, such as the excellent activity achieved with the PtRu and WMo catalysts.^439,440^ However, results controversial to the prior works are also found with the bimetallic nanocluster library, which could be caused by the different structures of the bimetallic nanoclusters in comparison to the catalysts in reported works.

### 6.9 Perspectives

From the structural point of view, bimetallic nanoclusters can be considered as transition materials between binuclear bimetallic sites and bimetallic nanoparticles. As reflected in the number of related publications, in comparison to the intensively studied field of bimetallic nanoparticles and the emerging field of binuclear bimetallic sites, the research efforts devoted to bimetallic nanoclusters are not as many as the other two fields in recent years. Taking into account the unique catalytic properties of monometallic clusters and their significant advantages over isolated metal atoms and nanoparticles in some important catalytic reactions, bimetallic nanoclusters deserve more attention from the catalysis community. On one hand, to address the distinct reactivity of bimetallic clusters, the future research efforts should be focused on the catalytic properties of subnanometer bimetallic clusters with less than 20 atoms because these entities show very different geometric and electronic structural features compared to binuclear sites and bimetallic nanoparticles. On the other hand, special attention should be paid to the systems in which bimetallic clusters may be formed under reaction conditions due to the structural evolution of binuclear bimetallic sites or bimetallic nanoparticles. In these cases, although the bimetallic nanoclusters are not present in the starting catalysts, they may serve as the real working species, which could be probably overlooked. To resolve this issue, operando studies are encouraged to be performed with the catalysts comprising binuclear bimetallic sites or bimetallic nanoparticles to identify to working active sites in those catalytic materials.

## 7 Catalytic Applications of Bimetallic Nanoparticles

Owing to the industrial interests of supported bimetallic catalysts, an enormous amount of work on the synthesis, characterizations and attempts to establish the structure–reactivity relationships of bimetallic nanoparticles has been carried out, and it has been reviewed and discussed in a number of review articles.^112,442−447^ In this section, we will highlight the progress achieved during the past decade on the catalytic applications of bimetallic nanoparticles. For each type of representative bimetallic nanostructures, we will start with discussions on their geometric and electronic structures and then the impacts of the unique structural features on their catalytic properties will be shown.

### 7.1 Structural Features of Various Bimetallic Nanostructures

**Figure 45:**
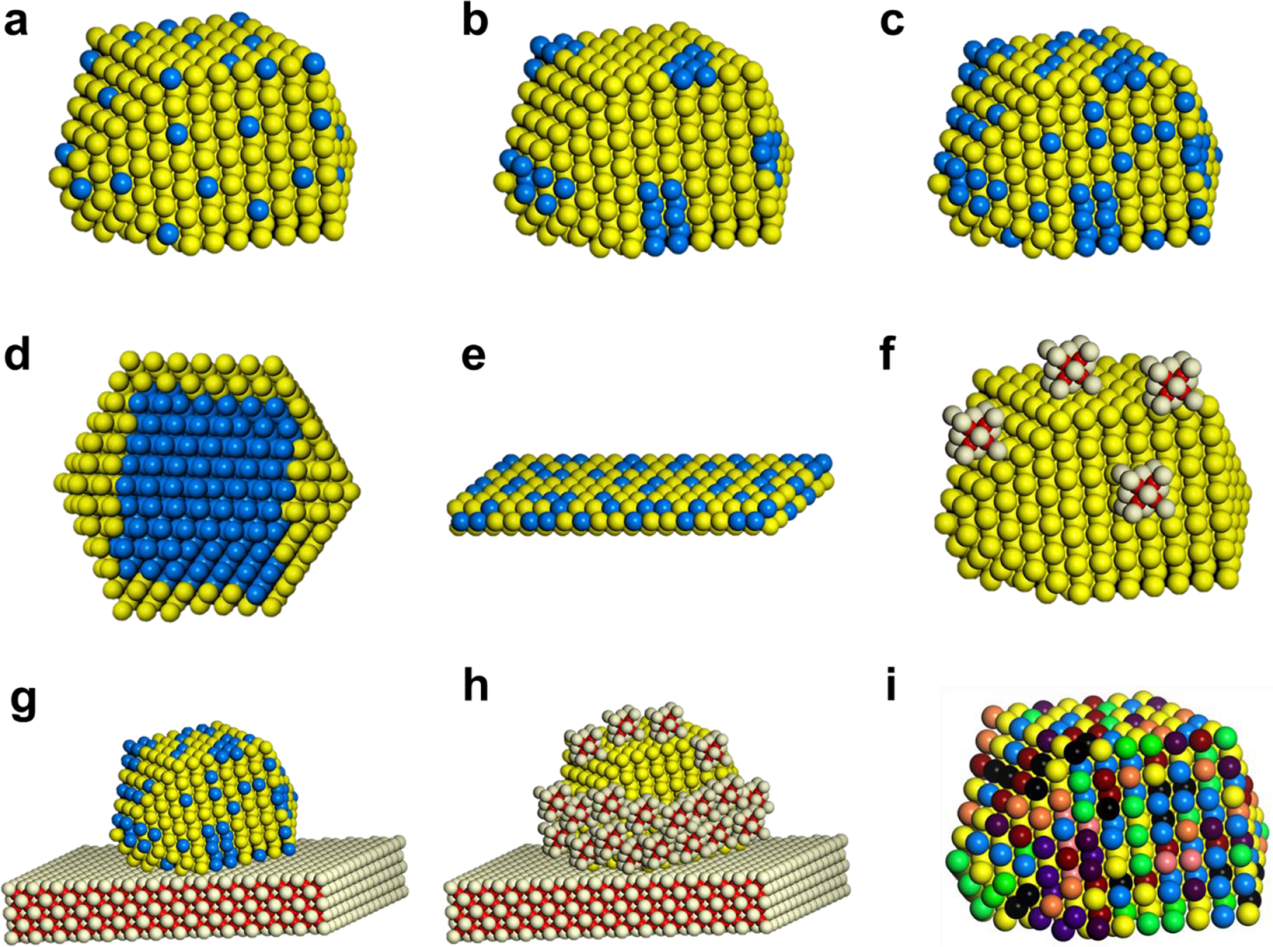
Illustrations of various types of bimetallic nanostructures. (a) Single-atom alloy, (b) bimetallic nanoparticle with local ensembles, (c) alloy nanoparticle with random distribution, (d) core–shell bimetallic nanoparticles, (e) two-dimensional bimetallic structure, (f) metal-oxide composite structure, (g) supported bimetallic nanoparticles, (h) bimetallic structure formed by strong-metal support interaction, and (i) high-entropy alloy nanoparticles.

The larger particle sizes of bimetallic nanostructures than binuclear bimetallic sites and bimetallic nanoclusters cause the higher structural diversity of bimetallic nanostructures than the subnanometer counterparts. As shown in Figure 45, depending on the spatial distribution of the two metal elements, the bimetallic nanoparticles can be generally categorized into the following types: single-atom alloy, bimetallic nanoparticle with local ensembles, bimetallic nanoparticle with random spatial distribution, and core–shell nanoparticle. Moreover, by decreasing the size of the nanoparticles to form low-dimensional bimetallic structures, a large number of undercoordinated surface sites will be exposed in the two-dimensional bimetallic structures (ultrathin nanosheets) and one-dimensional structures (ultrathin nanowires). Besides the combination of two metallic components to form bimetallic particles, the combination of a monometallic particle and a metal oxide component leads to the formation of metal-oxide interfacial structures, which may show distinct properties than the bimetallic particles due to the electronic interaction between the metallic particles and the oxide patches. The structural complexity will be further elevated when the number of involved metal elements is increased from 2 (bimetallic particles) to above 5 (high-entropy alloy particles). Because of the structural diversity, the structure–reactivity relationships of bimetallic nanostructures could be largely different from case to case. For each case, the structural characteristics need to be resolved by comprehensive characterization studies and catalytic studies based on a series of comparative catalysts are usually necessary for elucidating the active sites in the bimetallic nanostructures.

### 7.2 Catalytic Applications of Single-Atom Alloys

**Figure 46:**
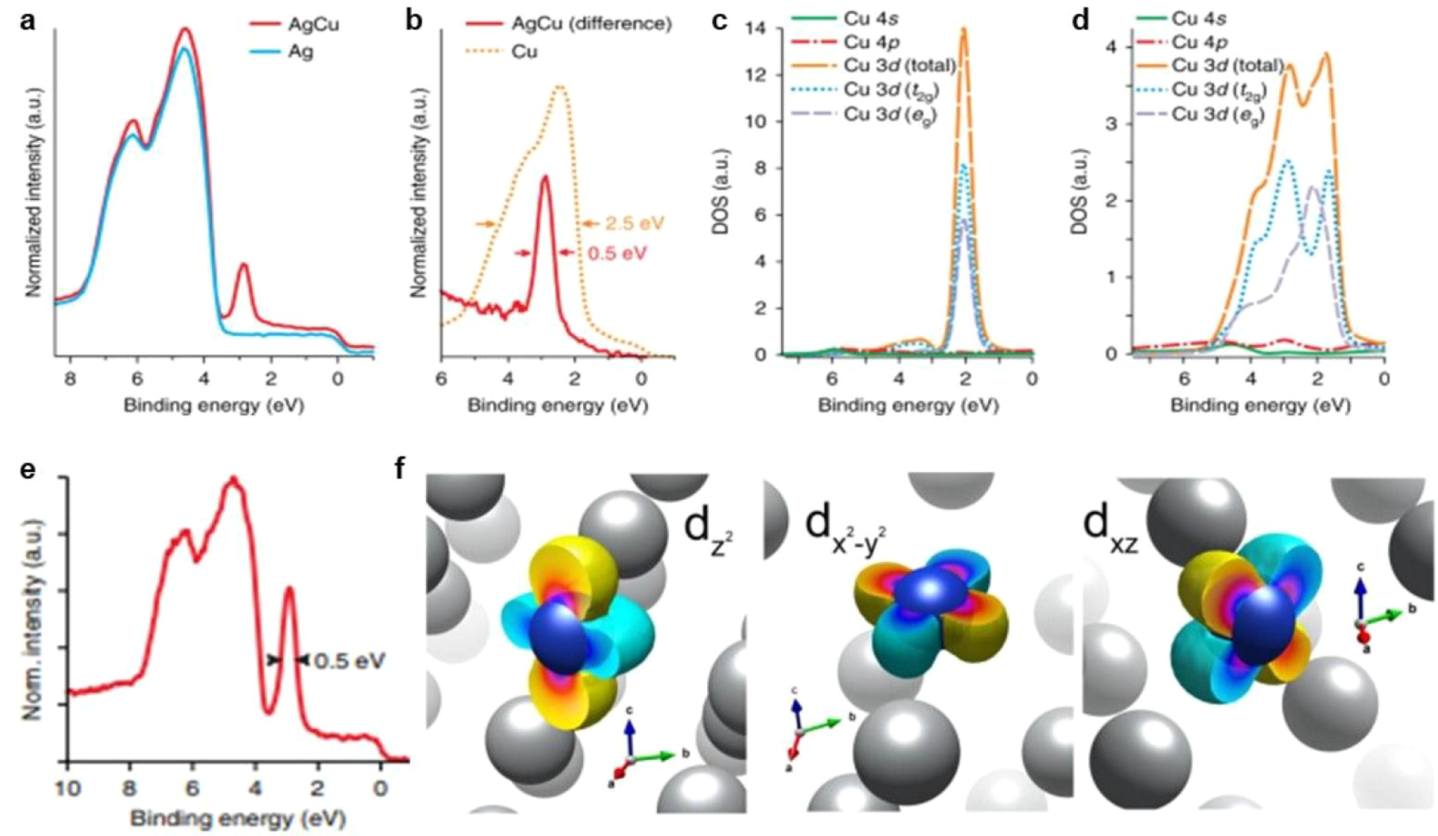
Electronic features of single-atom alloy catalyst. (a) Measured valence photoemission spectra (*hv* = 150 eV) of an AgCu alloy that contained 0.3 at. % Cu and metallic Ag. (b) Difference spectrum of AgCu and Ag, plotted with a Cu reference spectrum. (c) Calculated Cu-based projected density of states (DOS) of Ag_31_Cu_1_. (d) Calculated Cu-based projected DOS of pure bulk Cu. (e) The valence photoemission spectrum of Ag_99.5_Cu_0.5_ measured under methanol reforming conditions. (f) Calculated Cu 3d wave functions of Ag_31_Cu_1_. Reproduced with permission from ref (448). Copyright 2022 American Chemical Society.

In conventional bimetallic nanoparticles, there are regions with local segregation in the form of ensembles with a few atoms. By diluting the percentage of one metal element to a very low level (≤3 at. %), the atoms of one metal element are completely separated by the other metals, resulting in the isolation of metal atoms in a metallic matrix.^116^ As shown in Figure 46, the isolated Cu atoms in Ag matrix show a narrow d-band projected density of state (pDOS), while the pure Cu particle gives a broad one.^449^ Interestingly, the electronic features of the isolated Cu atom in Ag matrix is quite similar to the that of the free Cu atom, which is ascribed to the mismatch of the Ag 4d and Cu 3d states. The sharpness and degeneracy of the Cu 3d states in the single-atom AgCu alloy cause the different bonding with O atom in comparison with pure Cu particle because the isolated Cu atom in Ag matrix can interact with O atom through π bond, while the Cu atom in Cu particle has weaker interaction with O atom.

In other single-atom alloy systems, the electronic structure of the isolated metal atom is greatly altered by the matrix due to the electron transfer between the two metals. For instance, the Pd atoms in the Cu matrix are more electron-rich than the Pd atoms in the monometallic Pd particle, and the isolated Pd atoms show lower adsorption energies of H species, which is beneficial for electrocatalytic H_2_ evolution reaction.^451^ In another example, the electronic properties of the Pt matrix are modified by isolated Ni atoms, resulting in the decrease of the hydrogen-binding energy to the nearly optimal HER activity region.^452^ Moreover, the influence of isolated Ni atoms to Pt matrix is also reflected in the adsorption energy of CO, which facilitate the conversion of CO to CO_2_ in electrocatalytic oxidation of alcohol.

Bimetallic nanoparticles based on single-atom alloys have shown great potential in selective hydrogenation reactions in comparison with traditional alloy nanoparticles with local segregations. By diluting an active metal in a metallic matrix with low intrinsic activity, the selectivity for hydrogenation reactions can be greatly improved, because the side reactions caused by the segregated active metal atoms can be avoided due to the formation of single-atom alloys.^115,116^ For example, by introducing a very small amount of Pt or Pd in to Cu or Au nanoparticles, highly active and selective single-atom alloy catalysts have been prepared for a variety of selective hydrogenation reactions, such as hydrogenation of alkynes to alkenes, hydrogenation of 1,3-butadiene to butenes, hydrogenation of unsaturated aldehydes to unsaturated alcohols, and selective hydrogenolysis of glycerol to 1,2-propanediol.^110,453−455^ The formation of a single-atom alloy not only suppress the undesired side products from overhydrogenation reactions but also greatly improve the utilization efficiency of noble metals.

**Figure 47:**
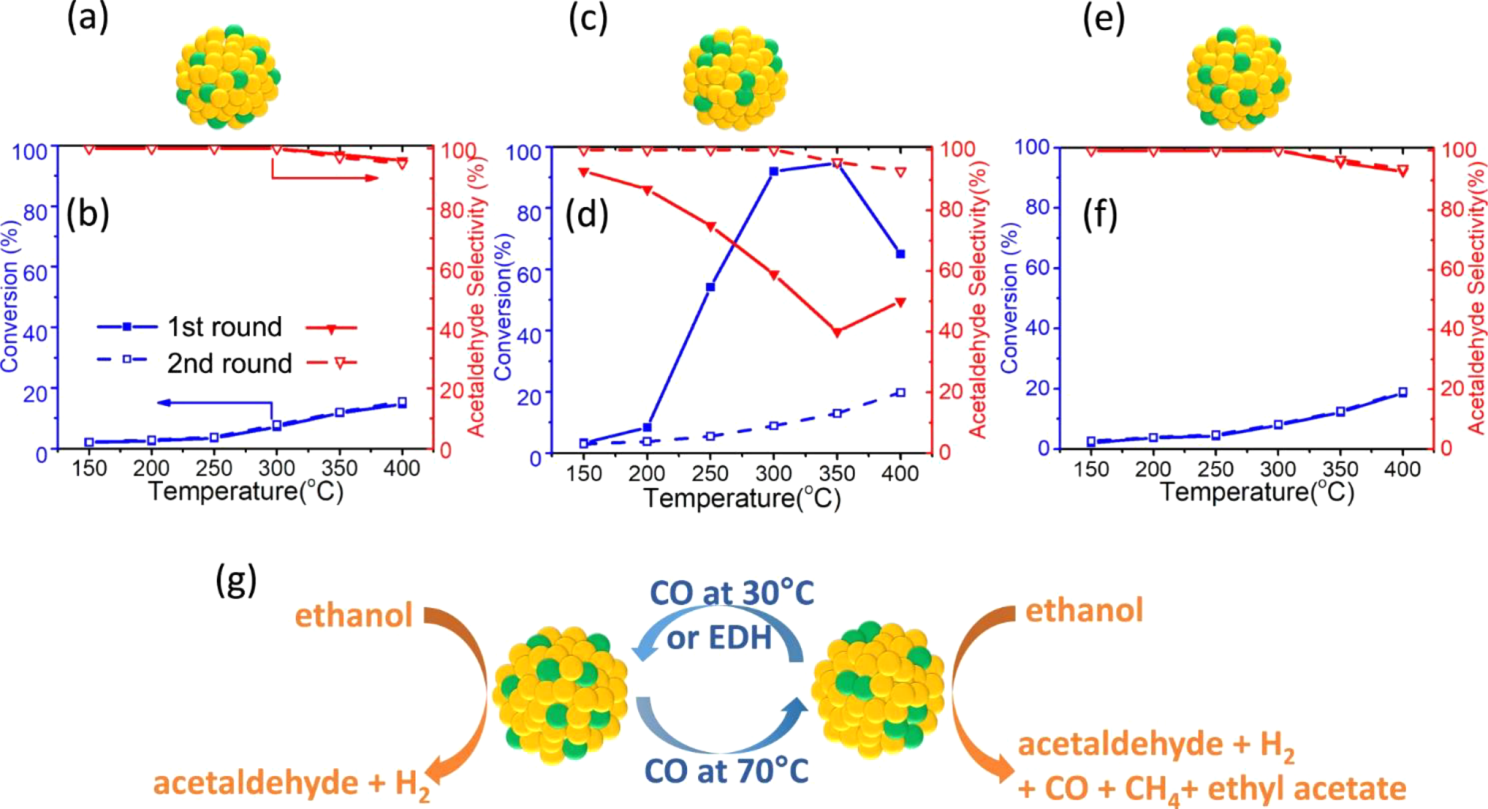
Single-atom PdAu alloys for ethanol dehydrogenation reaction. Structure (a,c,e) and catalytic performance (b,d,f) of a Pd_0.02_Au_0.98_/SiO_2_ sample after (a,b) no CO treatment, (c,d) CO at 30 °C for 30 min, then 70 °C for 30 min, which causes Pd to form clusters, and (e,f) CO treatment at 30 °C for 30 min, then 70 °C for 30 min, then 30 °C for 1 h, which causes Pd to aggregate and then redisperse back into atoms. (g) Schematic illustration of the structural transformation between single-atom PdAu alloy and bimetallic PdAu particles with Pd ensembles. The structural transformation is driven by CO treatment. The solid line shows the first-round ethanol dehydrogenation reaction from 150 to 400 °C, and the dotted line is the second-round reaction. Reproduced with permission from ref (450). Copyright 2021 Springer Nature under CC-BY license (https://creativecommons.org/licenses/by/4.0/).

The advantages of single-atom alloy catalysts have also been demonstrated in other reactions. For instance, for dehydrogenation of propane to propylene and dehydrogenation of ethanol to aldehyde,^450,456^ the isolated metal sites can selectively activate the C–H bonds while the deep dehydrogenation can be suppressed due to the less favorable binding of the primary products on isolated metal sites than that on sites with local segregation. As shown in Figure 47, isolated Pd atoms dispersed in Au matrix are highly active, and selective catalysts for dehydrogenation of ethanol to acetaldehyde while the presence of Pd ensembles will cause the formation a variety of products.^450^ Reversible structural transformation may occur under reaction conditions because the exposure of single-atom PdAu alloys to CO will cause the migration of Pd atoms to form Pd ensembles, which is not favorable for selective dehydrogenation reaction. Besides, isolated Ru atoms embedded in the matrix of Cu nanoparticles exhibit excellent activity for photocatalytic dry reforming of CH_4_ by serving the sites for transferring the hot electrons generated in Cu nanoparticles to reactants and then facilitating the activation of C–H bonds in CH_4_ and C–O bonds in CO_2_.^457^ Once local segregation of Ru atoms is present in the bimetallic RuCu nanoparticles, the undesired deep dehydrogenation of CH_4_ will occur, resulting lower selectivity to syngas (CO and H_2_) and also faster catalyst deactivation in comparison to the single-atom RuCu alloy particles.

From the fundamental point of view, the single-atom alloy catalysts allow description of the electronic structures of the metal centers with well-defined models. This advantage permits the establishment of the structure–reactivity correlation and the fast screening of potential catalytic materials for the target reactions, especially with the help of calculation methods based on machine learning.^458^ From the practical point of view, by diluting noble metals into a non-noble metal matrix for the generation of single-atom alloy, the normalized activity of the noble metal species can be greatly improved. However, such catalysts may still show low efficiency in the practical catalytic processes because the low metal loadings and insufficient yield of desired products on solid catalysts with limited mass or volume is the critical criteria. To overcome this limitation, more efficient synthesis methods are needed for the generation of dense single-atom alloy sites on the solid carrier.

### 7.3 Ensemble Effects in Bimetallic Nanoparticles

The ensemble effects in bimetallic catalysts proposed by Wolfgang Sachtler indicate the necessity of the formation of ensembles of metal atoms for performing the specific reaction.^460^ In other words, for such a type of a reaction, local segregation is required to form the ensemble of a few metal atoms for activation of the substrate molecules.

The size of the metal ensembles can affect the reaction pathways for a given catalytic reaction. For instance, the formation of Au–Pt interface can greatly promote the activity for electrocatalytic oxidation HCOOH, as indicated by the two-order magnitude enhancement in activity observed with Au@Pt and Pt@Au structure in comparison to the bare Au and Pt surface.^461^ From a mechanistic point of view, the oxidation of HCOOH may involves two pathways: the dehydrogenation pathway for production of formate as the intermediate and the dehydration pathway for production of CO as intermediate.^462^ Theoretical calculations indicate that the dehydrogenation pathway can occur with isolated Pt atom in the Au matrix, while the dehydration pathway is favorable on Pt ensemble with at least three Pt atoms.^463^ These insights are consistent with the experimental works, in which the Pt/Au ratios in the bimetallic nanoparticles have significant impacts on the catalytic properties for electrocatalytic oxidation of formic acid.^464^

**Figure 48:**
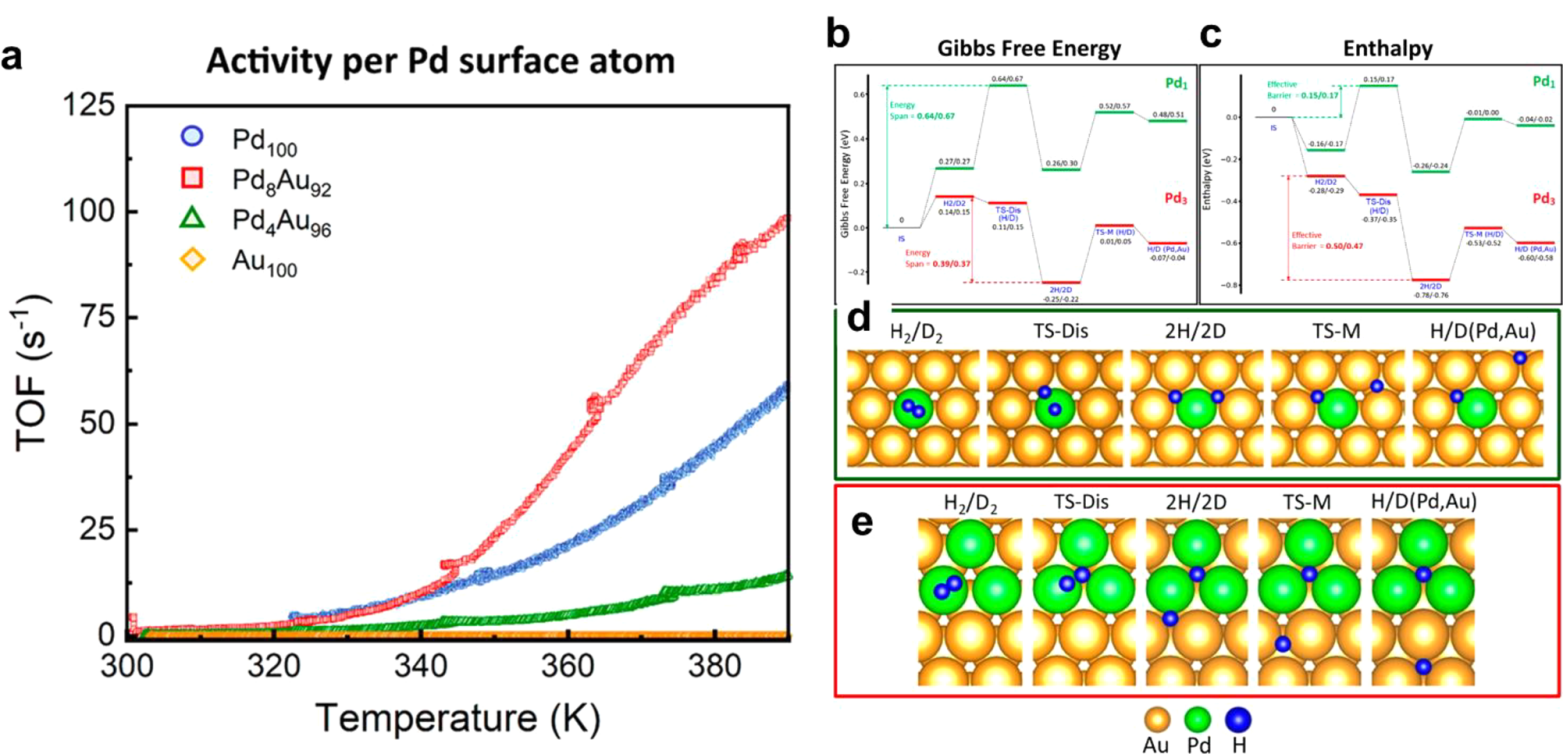
Ensemble effect in AuPd catalyst for isotopic exchange of H_2_ and D_2_. (a) Turnover frequency of Pd_100_Au_0_, Pd_8_Au_92_, Pd_4_Au_96_, and Pd_0_Au_100_ as a function of reaction temperature. The TOF for Pd_100_Au_0_, Pd_8_Au_92_, and Pd_4_Au_96_ is expressed in HD molecules converted per Pd surface atom per second. For Au_100_, the TOF is expressed per Au surface atom. A decahedral particle shape with Au(111) surface with a metal composition equal to the bulk Au/Pd ratio was assumed. (b–e) Density functional theory (DFT) calculations of H_2_ and D_2_ adsorption, dissociation, H migration, and recombination on a Pd single atom and on a Pd trimer ensemble in a Au(111) surface. (b) Gibbs free energy and (c) enthalpy profiles for Pd_1_ (green) and Pd_3_ (red) ensembles (in eV for *T* = 363.15 K and *P* = 0.2 bar). Free energy and enthalpy values for intermediates and transition states are provided both for H_2_ (left number) and D_2_ (right number) dissociation. The Gibbs free energy spans are indicated by a double-end arrow: H_2_/D_2_ dissociation from the gas phase to the dissociation transition state for Pd_1_ (0.64/0.67 eV). The reverse pathway corresponds to recombination of adsorbed H atoms to the H_2_/D_2_ molecular adsorption state (0.39/0.37 eV) for Pd_3_. (d,e) Schematics showing the different intermediates and transition states on Pd_1_Au and Pd_3_Au sites. Reproduced with permission of ref (459). Copyright 2021 American Chemical Society.

The importance of formation of Pd ensembles with multiple Pd atoms has also been suggested by theoretical study with PdCu alloy nanoparticles.^465^ Pd ensembles with Pd site on the external surface and at the subsurface region are the most active configuration of activation of H_2_, which is ascribed to the gradual shift of d-band centers to the Fermi level and the electron transfer between Pd ensemble and Cu matrix. As shown in Figure 48, the superior activity of metal ensembles over isolated metal sites for H_2_ activation is also demonstrated with bimetallic PdAu nanoparticles.^459,466^ With the ensembles of a few atoms, the reaction rates for dissociation of H_2_ will be greatly promoted. The capability for activation of small molecules such as H_2_ and O_2_ will influence their catalytic properties in selective oxidation and hydrogenation reactions. In this regard, the structural features of metal ensembles in bimetallic nanoparticles can affect the chemoselectivity. For example, bimetallic PdAg nanoparticles with diluted Pd in Ag matrix have been found as efficient catalysts for selective hydrogenation of acetylene to ethylene.^467^ In the experimental works, although Pd is highly diluted in Ag matrix, the optimum configuration of Pd site is still unclear. Theoretical calculations indicate that the isolated Pd sites and Pd dimers show low selectivity to ethylene due to the overhydrogenation reaction while the Pd trimers exhibit high selectivity to ethylene.^468^

The ensemble effects are not always beneficial for promoting the catalytic performances, as indicated by the catalytic behaviors of bimetallic AuPd nanoparticles, which are excellent catalyst for some reactions (such as oxidation of benzyl alcohol) but exhibit lower activities than the monometallic ones in some other reactions (such as CO oxidation and water–gas shift reaction).^13^ By rational design of a supported bimetallic Au–Pd/C and Au@Pd/C catalyst with separated Au and Pd domains, the catalytic performances for oxidation of 5-hydroxymethylfurfural in aqueous alkaline solution are enhanced because the Au and Pd sites can perform the dehydrogenation and O_2_ reduction steps, respectively.^469^ However, the optimum size of the metal ensembles for selective oxidation of alcohols require further investigations and the reasons why the bimetallic nanoparticles deliver lower performances than the monometallic particles for CO oxidation and water–gas shift reaction are unknown.

In the above examples, the ensembles are already formed in the starting catalyst. Taking into account the structural transformation of bimetallic nanoparticles under reaction conditions, ensembles could be generated in bimetallic nanoparticles as the consequences of the metal-reactant/product interaction. These phenomena have been observed with numerous systems, such as the formation of Pd domains in AuPd nanoparticles driven by the CO adsorption,^470^ formation of Ni-rich surface domains in CuNi nanoparticles under CO_2_ hydrogenation conditions,^471^ reversible formation of RhPd and RhPt nanoparticles under NO+CO reaction conditions,^472^ Pd-rich domains in PtPd bimetallic nanoparticles for methane combustion,^473^ Pd-rich domains in PdW bimetallic nanoparticles for electrocatalytic oxygen reduction,^474^ and Sn-rich domains in PtSn bimetallic nanoparticles for electrocatalytic methanol oxidation.^475^ Under certain conditions, the migration of the metal species may occur between the metal nanoparticles. For instance, the mixture of monometallic Pt and Au nanoparticles will transform into AuPt alloy nanoparticles during continuous potential cycling in electrocatalytic oxidation of formic acid, which deliver much higher activity than the monometallic nanoparticles.^476^

Understanding the size and coordination environment of the ensembles in the bimetallic nanoparticles are very helpful to elaborate the structure–reactivity relationships. A key scientific issue to clarify the ensemble effects is the structural characterizations of the ensemble species in bimetallic nanoparticles (in terms of the size and geometric features), which causes difficulties to establish the structure–reactivity relationships. The progress in this direction relies on the development of advanced characterization techniques (such as high-resolution TEM imaging and analysis technique) to quantitatively resolve the number and quality of various sites in a bimetallic nanoparticle. Nevertheless, fundamental studies based on well-defined surface or crystals can also bring very helpful insights, which can be used to correlated with the results obtained with practical catalysts based on bimetallic nanoparticles.^477^

### 7.4 Liquid Bimetallic Catalysts

**Figure 49:**
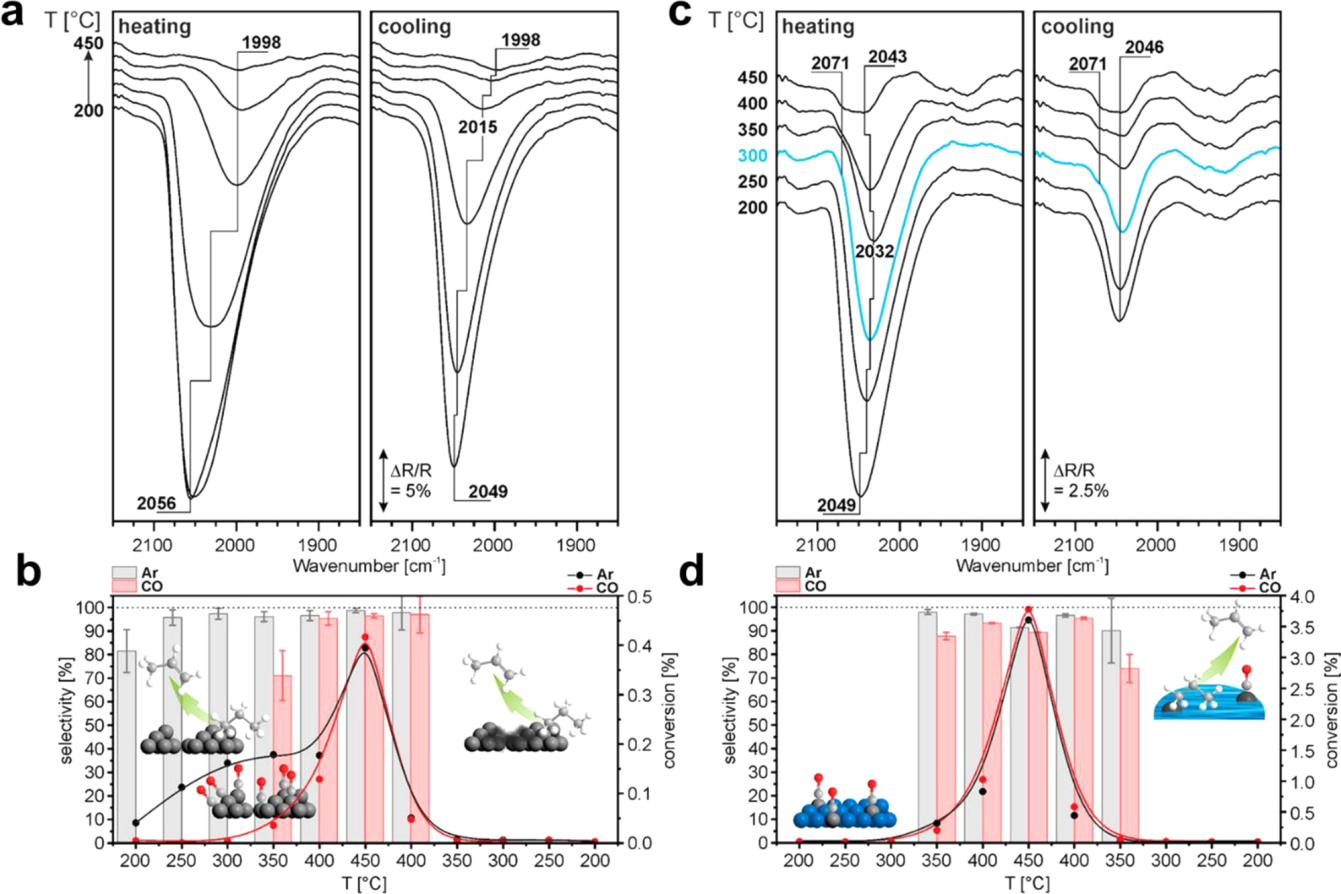
Spectroscopy and catalytic study of catalytically active liquid metal solutions for propane dehydrogenation reaction. (a) Operando DRIFT spectra recorded on the Pt/Al_2_O_3_ sample at selected temperatures. (b) Conversion of propane and selectivity toward propene for the Pt/Al_2_O_3_ sample. (c) Operando DRIFT spectra recorded on the Ga_37_Pt_1_/Al_2_O_3_ supported catalytically active liquid metal solutions sample at selected temperatures. (d) Conversion of propane and selectivity toward propene for the Ga_37_Pt_1_/Al_2_O_3_ sample. Reproduced with permission of ref (478). Copyright 2019 American Chemical Society.

Liquid bimetallic nanoparticles are emerging catalytic materials because they can combine some advantages of conventional heterogeneous catalysts (such as easy separation and high stability) and homogeneous catalysts (electronic-steric control due to the formation of isolated metal sites in a liquid metal matrix).^479^ For instance, when Ga/Pd ratio is higher than 10 and the temperature is above 400 °C, liquid bimetallic alloy will be formed. Following the phase diagram, a series of Ga-rich PdGa bimetallic catalysts have been prepared by galvanic replacement reaction between liquid Ga particles and (NH_4_)_2_PdCl_4_, giving the formation of spherical PdGa particles (Ga/Pd atomic ratios ≥10) on porous glass.^480^ The reactivity of the Ga-rich PdGa particles has been tested for dehydrogenation of butane to butenes, in which the reactivity of PdGa samples with diluted Pd species show remarkably higher activity and stability than the monometallic Pd/SiO_2_ catalyst and PdGa catalyst in the form of solid alloy. The liquid PdGa alloy catalysts even show superior performance in comparison with the classic Cr/Al_2_O_3_ and Pt/Al_2_O_3_ for alkane dehydrogenation reaction. Detailed structural characterizations show that the isolated noble metal atoms (such as Pd, Pt, and Rh) diluted in liquid Ga matrix will be highly mobile.^478^ As suggested by in situ CO-IR spectroscopy and structural characterizations, the noble metal atoms can move between surface and bulk in a highly dynamic manner.^481,482^ The alkane molecule can be converted to alkene through dehydrogenation reaction on the surface noble metal atoms, and the noble metal atoms can subsequently migrate to bulk of Ga matrix, leading to the desorption of the alkene product. Interestingly, as shown in Figure 49, the isolated Pt atoms in the Ga matrix are resistant to CO-poisoning during propane dehydrogenation reaction while the conventional Pt/Al_2_O_3_ catalyst will be partially deactivated by CO due to the strong adsorption of CO on Pt nanoparticles.^478^ This feature infers that supported liquid alloy catalysts may have potential applications for selective hydrogenations with resistance to poison impurities.

In the above-mentioned works related to supported liquid nanoparticles, the synthesis and catalytic applications usually involve the treatments at temperatures above 300 °C. The formation of liquid bimetallic nanoparticles is feasible at low temperature by diluting a very low amount of Pt in the liquid Ga nanoparticles.^483^ Pt species exist as isolated sites in the liquid Ga particles and show high mobility in the liquid Ga particles, which are highly active for electrocatalytic oxidation of methanol at 70 °C. One explanation for unique reactivity of the highly mobile Pt atoms in liquid Ga nanoparticles is the steady renewal of the catalytic metal centers at the interface in comparison to the fixed metal–support interface in conventional heterogeneous metal catalysts. The highly dynamic structures of the soluble metal species in the liquid metal enables the high utilization efficiency of the noble metals and the stability against deactivation under reaction conditions.

It can be anticipated that, in order to control the migration behavior of the Pt atoms in liquid metal particles, the size and morphology of the liquid Ga nanoparticles need to be controlled.^484^ Beside the use of Ga as the “solvent” for another metal element, other metals with low melting point such as In, Sn, and GaIn alloy are also potential candidate materials for preparation of liquid bimetallic particles.^485^ In the above-mentioned works, the noble metal atoms (Pt and Pd) are proposed as isolated sites in the liquid matrix for a series of reaction. However, the presence of metal ensembles formed by a few noble metal atoms is not completely excluded according to the characterization results, and the role of the metal ensembles in the catalytic reactions is unclear.

Although the conventional bimetallic particles usually do not show such highly dynamic and mobile features as the liquid alloy particles at ambient conditions, the mobility of the bimetallic nanoparticles could be underestimated under reaction conditions (e.g., high temperature, high pressure, and in the presence of reactants), especially when the sizes of the bimetallic nanoparticles are below 2 nm. The knowledge and insights derived from the studies with supported liquid bimetallic particles could be translated to understand the dynamic structures of bimetallic nanoparticles under reaction conditions. For instance, combination of spectroscopic characterization and multiscale theoretical modeling indicate that the isolated of Pt sites and the mobility of surface Pt and Ga atoms are key features that account for the high activity of supported PtGa nanoparticles (∼1 nm) for propane dehydrogenation reaction.^486^

### 7.5 Core–Shell Bimetallic Nanoparticles

In numerous studies based on model surface systems, the electronic structures of a monolayer metallic surface will be greatly influenced by the subsurface metal and therefore affect the catalytic properties of the surface monolayers.^487,488^ For instance, the d-band centers can be modulated by varying the subsurface 3d metals from Ti to Ni, leading to the variation of the binding energy of probe molecules such as ethylene, water and CO.

By translating the knowledge accumulated in surface systems to practical systems based on nanoparticulate systems, numerous catalysts based on core–shell bimetallic nanoparticles have been designed and prepared. For example, the reactivity for electrocatalytic H_2_ evolution reaction is improved by decreasing the thickness of the Pt shell in Pd@Pt core–shell nanoparticles, which is ascribed to the modification of the electronic structure of the Pt overlayers caused by the charge transfer from Pd core to Pt overlayers.^489^ In the case of Pd@Pt core–shell nanoparticles formed by deposition Pt overlayers on Pd icosahedra, the structure with three Pt overlayers is found to be the most active configuration for electrocatalytic oxygen reduction reaction.^490^ The advantages of core–shell bimetallic nanoparticles over the alloy nanoparticles have also been demonstrated with Au@Co structure for the hydrolytic dehydrogenation of ammonia borane and Pd@Pt core–shell nanoparticles encapsulated in MOF matrix for CO oxidation.^491,492^

Not only activity, but also selectivity can also be altered by the thickness of overlayers. For instance, the thickness of Pd overlayers on Au nanorods can greatly influence the selectivity for selective hydrogenation of 1,3-butadiene to butenes.^493^ With a thicker overlayer of Pd on Au nanorods, the selectivity to the butenes would be lower. Such an effect has also been observed with Pt overlayers on Au nanoparticles for selective hydrogenation of nitroaromatics.^494^ These works imply that the optimum thickness of the metal layers in core–shell nanoparticles could be dependent on the target reaction, which could be further associated with the thickness-dependent electronic features of the metal overlayers. Usually, the optimum thickness of the shell composition would be ≤3 atomic layers, because thicker overlayers will cause physicochemical properties similar to the bulk metal.

In the above examples, the metallic core is fully covered by another metallic shell. However, if the loading of the shell metal is low, core–shell type structures with submonolayer coverage will be formed. Theoretical calculations suggest that the edge and terrace sites of the Ni patches deposited on Pt(111) surface can efficiently catalyze the ammonia decomposition reaction.^495^ This study infers the importance to control the morphology and domain size of the submonolayer structure on a metal substrate. It has been shown with Pd@Au nanoparticles that Pd dimers supported on Au particles will greatly improve the activity for electrocatalytic reduction of CO_2_ to CO, because Pd dimers can efficiently activate CO_2_ and mitigate the deactivation problem caused by strong adsorption of CO.^496^ By tip-enhanced Raman spectroscopy, the activated H species can be spilled over to the Au substrate and the distance of the H spillover can be as far as ∼20 nm.^497^ Actually, this concept has already been practiced in an early work, in which the catalytic performance of Au/TiO_2_ for chemoselective hydrogenation of nitroaromatics can be greatly enhanced by depositing a very small amount of Pt species (e.g., 100 ppm Pt in the total weight of the Au/TiO_2_ catalyst) on Au particles.^12^

### 7.6 Metal Nanoparticles Modified with Surface Oxide Domains

The bimetallic nanoparticles are supported on solid carriers, but the reversible configuration, in which the metal nanoparticles are modified with another metal oxide species, has also been observed in numerous systems. Such structures are usually formed between noble metals and oxyphilic metal elements. For instance, the deposition of a monolayer of oxide/hydroxide species (such as FeOx and Ni(OH)_2_) on a Pt surface can generate highly active interfacial sites for CO oxidation reaction.^500,501^ The metallic surface and metal-oxide interface are responsible for activation of CO and O_2_, respectively. Controlling the chemical states of the surface oxide domains/layers is critical for maintaining high activity because overoxidation or over-reduction may cause the loss of metal-oxide interfacial sites.^502^

**Figure 50:**
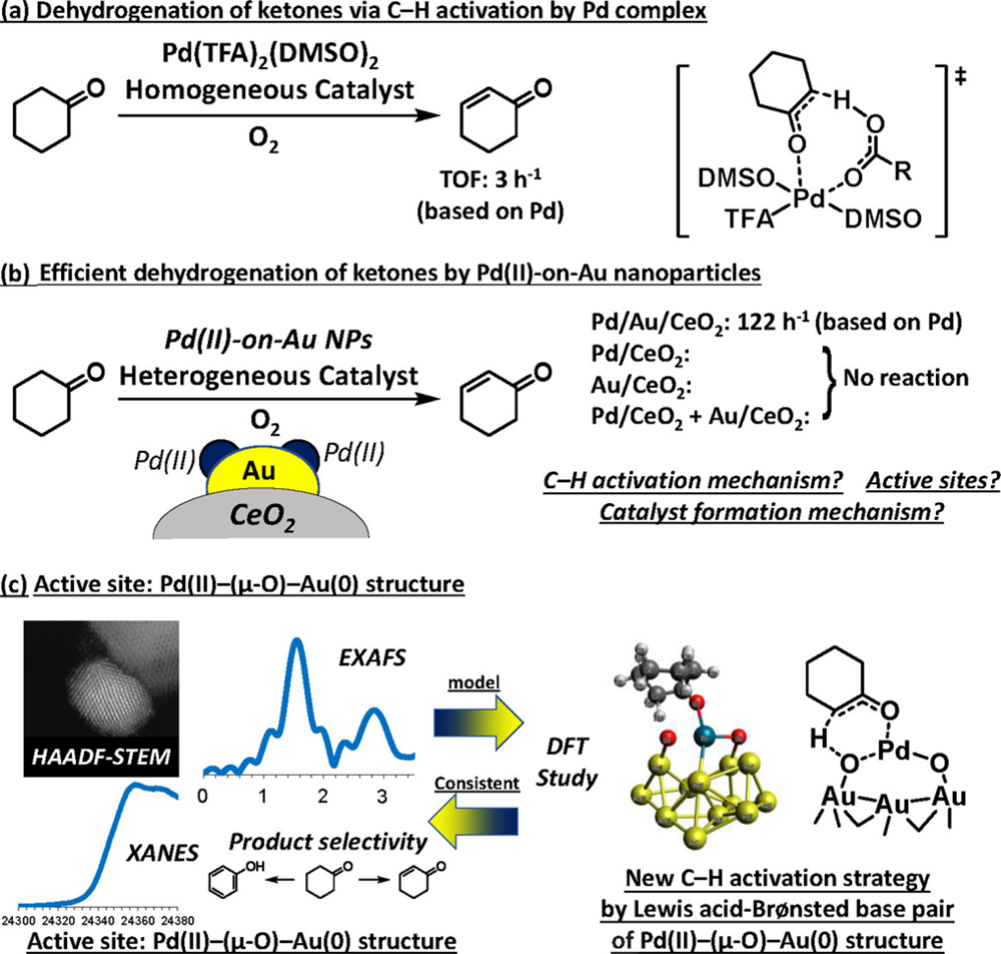
Homogeneous or heterogeneous catalysts for dehydrogenation of cyclohexanone. (a) Pd complex, (b) Au nanoparticles modified with Pd(II) species supported on CeO_2_ as heterogeneous catalyst for dehydrogenation of cyclohexanone. (c) Identification of the structure of the active sites in bimetallic Pd–Au/CeO_2_ catalyst. Reproduced with permission from ref (498). Copyright 2022 American Chemical Society under CC-BY license (https://creativecommons.org/licenses/by/4.0/).

The dehydrogenation of cyclohexanone is greatly enhanced by modifying the surface of Au nanoparticles with Pd(II) species, because the Pd(II) sites can activate the C–H bonds and the metallic Au surface can facilitate the β-H elimination step (see Figure 50).^498,503,504^ Interestingly, the synergy of Au and Pd cannot be achieved with AuPd bimetallic nanoparticles, because the rate-limiting step, activation of C–H bonds, is not favorable on bimetallic AuPd surface.

One difference between core–shell bimetallic structures and metal-oxide interfacial sites is the chemical states of the oxidized species which can serve as the Lewis acid sites, leading to the generation of bifunctional catalysts. This synergy is reflected with the bimetallic Ru-ReOx/SiO_2_ catalyst developed for production of secondary alcohols from 1,2-alkanediol by C–O hydrogenolysis.^505^ The ReOx species located on the metallic Ru particles can activate the −OH group in 1,2-alkanediol and then facilitate the breaking of C–O bonds with the help of activated H species on Ru particles. Metallic Re particles are inactivate for this reaction because of the loss of Lewis acidity. The synergy between metallic and oxide component for a variety of reactions dealing with derivatives from biomass because those catalytic transformations usually involve the hydrogenolysis or hydrodeoxygenation reactions.^506−508^

**Figure 51:**
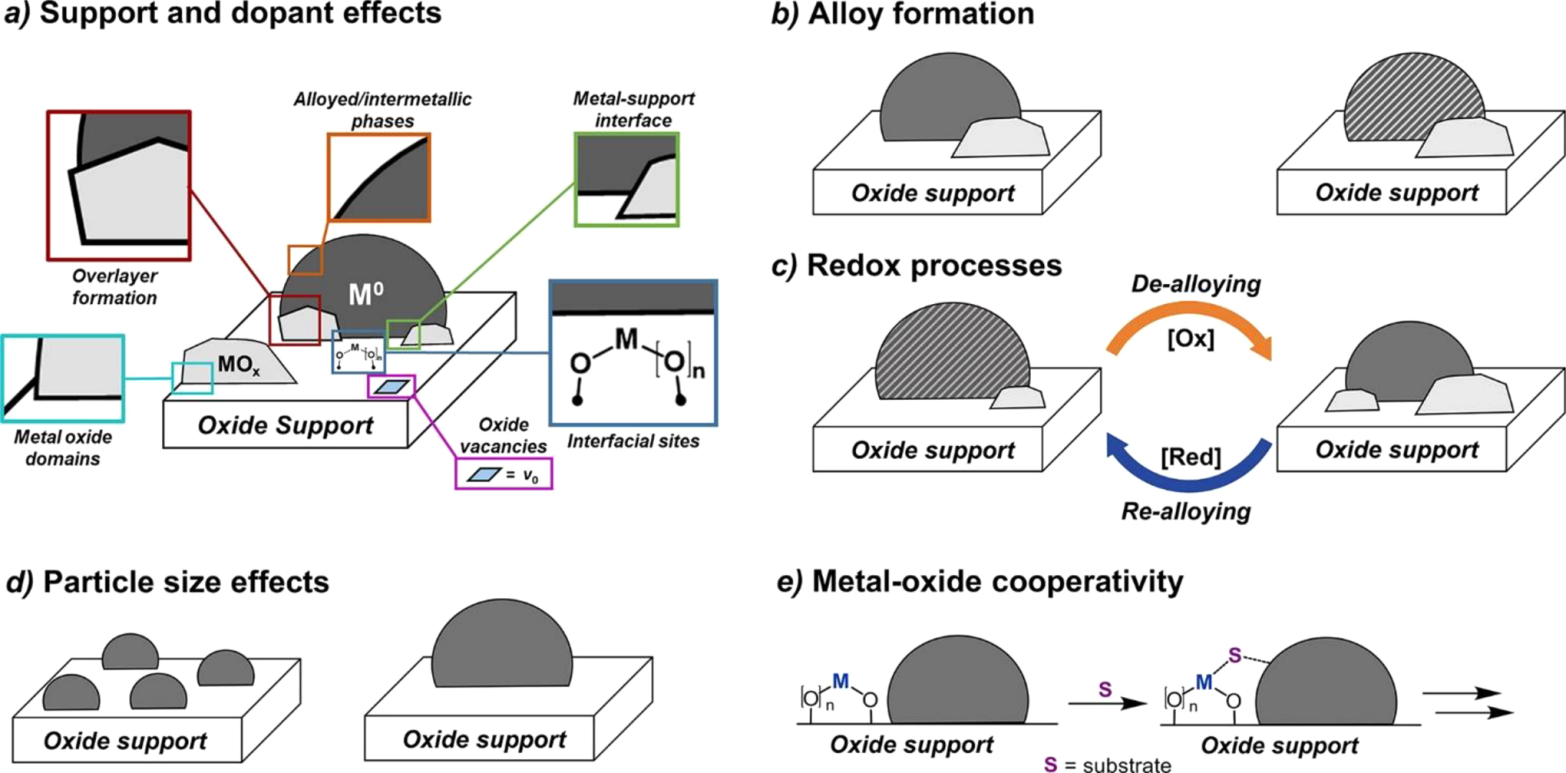
(a) Representation of a range of support and dopant effects. (b) Schematic of nonalloyed and alloyed nanoparticles. (c) Dealloying and realloying as an example of redox processes under reaction conditions. (d) Particle size effects and divergent reactivity. (e) Synergistic activation of a substrate at the metal–support interface. Reproduced with permission from ref (499). Copyright 2021 American Chemical Society.

As shown in Figure 51, the structural features of the metal-oxide interfacial structures are greatly influenced by various parameters and for different types of metal-oxide interfaces, the catalytic properties could be largely different.^499^ Some fundamental issues related to the metal-oxide bimetallic sites are still not clear in those reported systems, such as the optimal size of each component and the atomic structures of the metal-oxide interface. On one hand, researchers may employ advanced characterization techniques to address the detailed structures of the metal-oxide bimetallic sites. On the other hand, preparation of metal-oxide model catalysts with well-defined structural features will be very helpful to elucidate the nature of the active sites.^509^ The latter approach includes the construction of well-defined metal-oxide interfacial structures based on surface organometallic chemistry and construction of model system in surface science studies.^499,510^

### 7.7 Bimetallic Nanoparticles Derived from Metal–Support Interaction

The strong metal–support interaction (SMSI) has profound implications in the catalytic properties of supported metal catalysts. In the classic model of SMSI, the metal particles will be partially or fully covered by the overlayers from the support under high-temperature treatments. The detailed characterizations have revealed that, beside the TiOx overlayers, PtTi alloy particles are formed in Pt/TiO_2_ catalyst due to the SMSI, according to the in situ TEM, EXAFS, EELS, and CO-IR results.^511−513^ The morphological characterization results show that, the PtTi alloy structures are only formed on the surface of the Pt nanoparticles and probably exhibit the thickness of less than three atomic layers. The formation of TiOx overlayers is proposed as a key consequence caused by the SMSI and then has profound influences on the behavior of Pt/TiO_2_ for chemoselective hydrogenation reactions.^514^ In this sense, it will be important to elucidate the impacts of TiOx overlayers and the Pt–Ti alloy on the catalytic properties.

**Figure 52:**
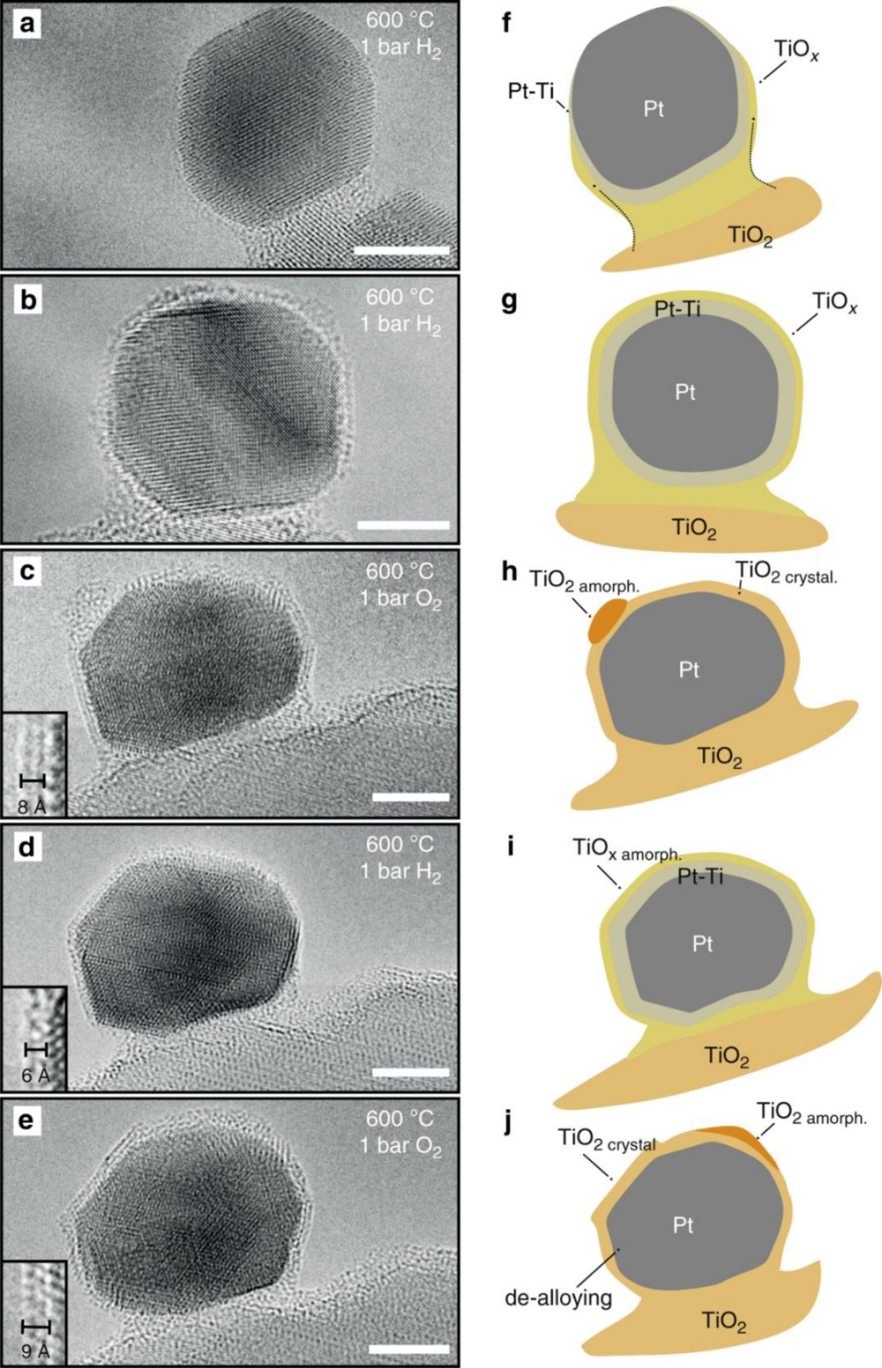
Evolution and dynamic structural changes of the overlayer in SMSI. A platinum particle on a titania support in the first exposure to H_2_ at 600 °C (a,b) and the subsequent atmosphere change to O_2_ at 600 °C (c), a switch to H_2_ (d) and then a switch to O_2_ again (e), and interpretation of the phenomena based on the combined results of in situ transmission electron microscopy, in situ X-ray photoemission spectroscopy, and in situ powder X-ray diffraction (f–j). Insets for c–e show a magnified image of the overlayer structure observed. Scale bar is 5 nm. Reproduced with permission from ref (511). Copyright 2020 Springer Nature under CC-BY license (https://creativecommons.org/licenses/by/4.0/).

Although the SMSI could be reversible in Pt/TiO_2_ catalysts, the atomic structure of Pt–TiO_2_ interface and the morphology of Pt nanoparticles will also change during the consecutive reduction–oxidation treatments, as shown by the in situ TEM images.^511^ As shown in Figure 52, the ultrathin PtTi alloy structures may also evolve with the treatments, which could explain the variation of the catalytic performance of Pt/TiO_2_ catalyst observed in some catalytic studies as well as other TiO_2_-supported metal catalysts.^515,516^ It should be noted that, the TiO_2–*x*_ overlayers shown in the in situ TEM study are not fully removed by the exposure to O_2_ atmosphere, which is different from that observed in ex situ studies and could be related to the pressure gap between in situ TEM study and ex situ studies.^517,518^

The formation of alloy nanoparticles induced by SMSI has also been observed in Pt/CeO_2_ catalyst, in which the Pt–Ce bonding is formed after exposure to CH_4_ at 975 °C.^519^ The formation of Pt–Ce bonding could be understood as the reduction of CeO_2_ support, and it requires higher temperature than the formation of Pt–Ti bonding in Pt/TiO_2_ catalyst, which should be related to the reducibility of the oxide support. It is shown that, SMSI can occur after reduction by H_2_ at 975 °C, while the reduction at 550 °C cannot trigger the SMSI in Pt/CeO_2_.^520^ Formation of Pt-based alloy nanoparticles is facilitated with Pt supported on two-dimensional transition metal carbides (MXenes), as demonstrated with PtNb alloy nanoparticles formed at 350 °C and PtTi alloy nanoparticles formed at 550 °C after reduction treatment by H_2_.^521,522^ The unique electronic properties of the MXene supports could be the reason accounting for the easy formation of these alloy nanoparticles. The Pt-based alloy nanoparticles supported on MXenes show promising performances for several reactions, such as water–gas shift, electrocatalytic H_2_ evolution, and alkane dehydrogenation reactions.

Another typical case involving the formation of bimetallic sites is the Cu–Zn system for methanol synthesis from CO/CO_2_ hydrogenation. There are still many debates on the active sites as reflected in the literature, and the CuZn alloy phase is one of the proposed active sites for methanol synthesis.^523,524^ By tuning the grazing incidence angle of in situ X-ray photoelectron spectroscopy measurements, it is possible to detect the electronic features of the surface Cu and Zn species. The results show that the presence of CO will contribute the reduction and migration of Zn species to form Zn–Cu alloy, and the surface structure is greatly dependent on the atmosphere.^525^

### 7.8 Low-Dimensional Bimetallic Structures

The particle size effects of metal catalysts have been intensively studied in heterogeneous catalysis and the particle sizes of the metal species indeed have great impacts on the catalytic consequences in enormous structure-sensitive reactions.^526^ However, the majority of the studies on particle size effects are based on spherical or nearly spherical particles. In the case of low-dimensional bimetallic structures in the form of ultrathin nanosheets (2D) or nanowires (1D), the unique geometric structural features can bring novel catalytic properties different to three-dimensional nanoparticles and nanoclusters.

One-dimensional bimetallic nanowires with average diameters below 1 nm have been prepared as efficient catalysts for electrocatalytic oxygen reduction and hydrogenation oxidation reactions.^527,528^ The abundant (111) facets in the ultrathin Pt-based bimetallic nanowires are proposed to be responsible for the high activities. This concept has also been shown with the two-dimensional PdMo nanosheets, whose ultrathin structures also provide abundant exposed (111) facets.^136^ The PdMo nanosheets with thickness below 1 nm show remarkably high activity and excellent stability for electrocatalytic oxygen reduction and oxygen evolution reactions. Electron transfer from Mo atoms to Pd atoms is suggested by the DFT calculations, which shifts the center of the band toward negative energy relative to the monometallic Pd nanosheet. The downshift of the d-band center relieves the overbinding of oxygen on the surface, resulting in an optimal oxygen adsorption energy. The superior electrocatalytic performance of bimetallic nanosheets has also been observed with PdFe nanosheets for reduction of N_2_, which is attributed to the efficient N_2_ activation via N_2_-to-Fe σ-donation on the isolated Fe sites in the Pd matrix.^529^

The unique catalytic properties of isolated metal atoms and subnanometer metal clusters can be ascribed to their molecule-like electronic structures.^530^ Novel catalytic properties distinct to conventional nanoparticulate materials usually appear when the sizes of metal entities are decreased to ≤0.7 nm (corresponding to the size of a M_13_ cluster or species with lower atomicity). In this regard, if the diameter/thickness of ultrathin one-dimensional/two-dimensional bimetallic structures can be further decreased to below 0.5 nm, which corresponds to 1–2 atomic layers, a more profound difference between these ultrathin materials and conventional small nanoparticles could be observed, and these low-dimensional bimetallic structures may show catalytic applications beyond the scope of bimetallic nanoparticles.^531^

### 7.9 Bifunctional Catalysts Based on Bimetallic Nanoparticles

**Figure 53:**
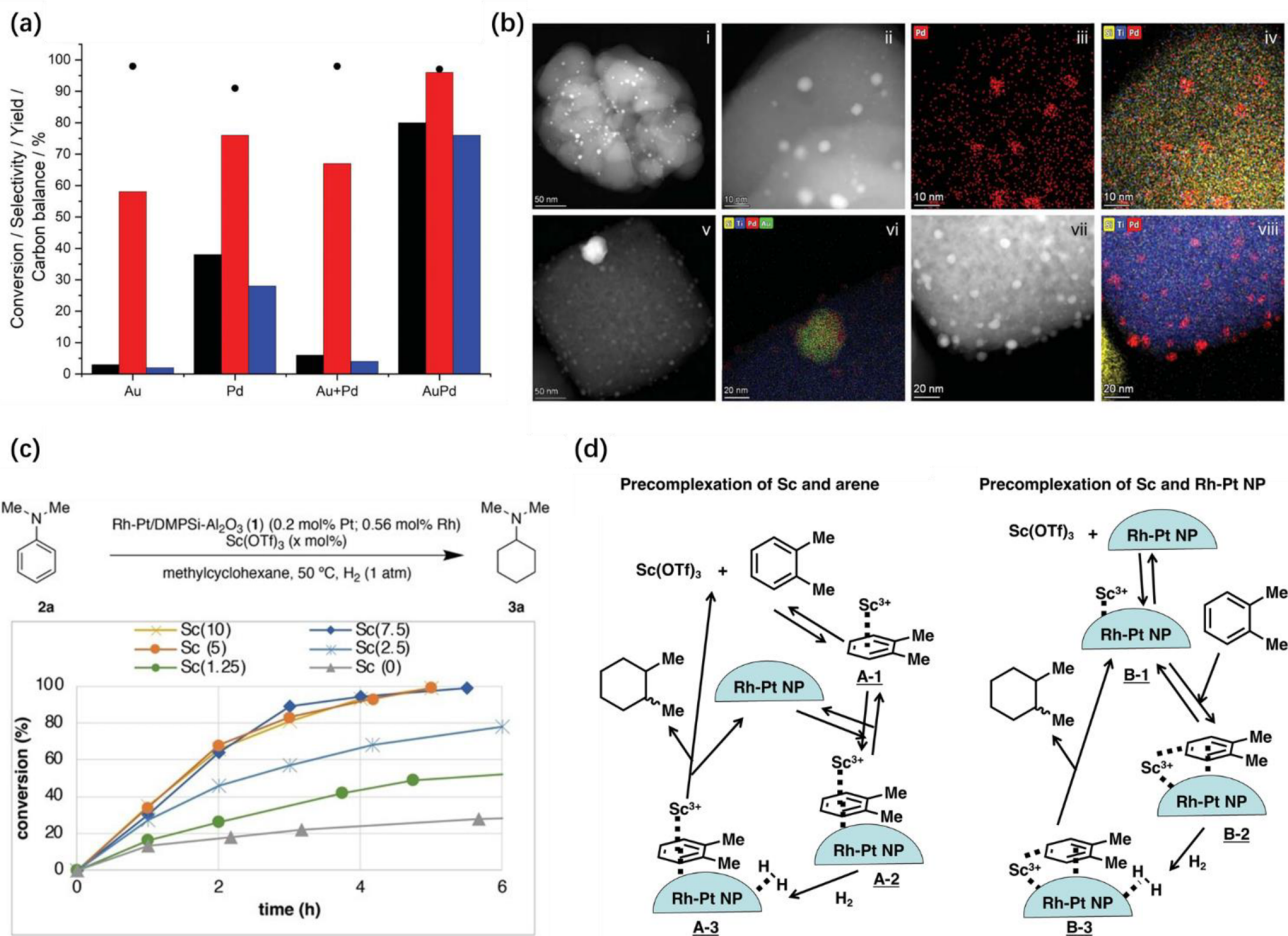
Bifunctional catalysts based on bimetallic nanoparticles. (a) Catalytic activity as a function of the Pd precursor and heat treatment regime. (b) Structural characterization of the fresh AuPd/TS-1 sample. (i) Lower and (ii) higher magnification HAADF-STEM images of the titanosilicate majority component. (iii) XEDS map showing Pd metal attachment only and absence of Au on TS-1. (iv) XEDS overlay map showing Si (yellow), Pd (red), and low-concentration Ti (blue). (c) Hydrogenation of arenes with supported RhPt nanoparticles and Lewis acid as cocatalyst. Reaction profiles with series of Sc(OTf)_3_ loading. (d) Two proposed reaction mechanisms. One is based on precomplexation of Sc^3+^ and arene, while the other one is based on precomplexation of Sc^3+^ and RhPt nanoparticles. (a,b) Reproduced with permission from ref (532). Copyright 2022 AAAS. (c,d) Reproduced with permission from ref (533). Copyright 2022 Wiley-VCH.

Bifunctional catalysts are widely used catalysts in numerous processes, in which the two types of active sites (metal sites for redox reactions and acid/base sites for acid–base reactions) work in a synergistic way for catalytic reactions involving multiple steps.^534,535^ The combination of bimetallic nanoparticles and acid/basic sites can enable the reactions that a solo type of functional component is not sufficient to achieve or significantly promote its performance. The dispersion of Pt in beta and USY zeolite can be improved by addition of Pd to form bimetallic PtPd nanoparticles.^536^ Consequently, a better balance between the metallic sites and the acid sites can be achieved for isomerization and hydrocracking of heptane than the monometallic Pt-zeolite counterparts. The advantage of bifunctional catalysts has also been demonstrated in cascade process involving the formation of metastable intermediates. As presented in Figure 53a,b, by loading bimetallic AuPd nanoparticles on TS-1 zeolite, a cascade transformation from ketone to oxime can be achieved with the AuPd/TS-1 bifunctional catalyst, in which the AuPd nanoparticles are responsible for synthesis of H_2_O_2_ from H_2_ and O_2_ while the TS-1 zeolite provides acid sites for cyclohexanone ammoximation.^532^

Bifunctional catalysts based on bimetallic particles and acid/basic sites are also excellent catalysts for hydrogen-transfer reactions because the bimetallic nanoparticles and acid/basic sites can work in a synergistic manner for the dehydrogenation, intermediate formation, and hydrogenation elementary steps.^537^ The participation of both the metallic component and the support in hydrogen-transfer catalysis are demonstrated with monometallic catalysts supported on oxide.^538,539^ The use of bimetallic nanoparticles instead of monometallic nanoparticles can further promote the activities. For instance, the N-alkylation of primary amides is achieved by the cooperative catalysis based on AuPd bimetallic nanoparticles and homogeneous Lewis acid (Ba(OTf)_2_).^540^ The bimetallic AuPd nanoparticles supported on TiO_2_ and Al_2_O_3_ give excellent activity for amination of alcohols and ketones.^541,542^

Another way to form bifunctional catalytic system is to introduce homogeneous complex to work together with the bimetallic nanoparticles by serving as the acid/basic functional component. For example, as shown in Figure 53c,d, the combination of Sc(OTf)_3_ and RhPt nanoparticles are efficient bifunctional catalyst for hydrogenation of arenes.^543^ The role of Sc^3+^ is proposed to enhance the adsorption of arene on RhPt nanoparticles and facilitate the transfer of activated H species from RhPt nanoparticles to the aromatic ring. The synergy between bimetallic nanoparticles and Lewis acid sites has also been shown with AuPd nanoparticles and Mg^2+^ for N-alkylation of primary amides with primary alcohol.^540^ The AuPd nanoparticles account for the hydrogenation transfer (dehydrogenation of alcohol to aldehyde and subsequent hydrogenation of imine intermediate), while the Lewis acid sites account for the condensation between primary amide and aldehyde.

The spatial distribution of the two functional components in bifunctional catalysts has profound influence on the catalytic performances, as shown with metal-zeolite catalysts for hydrocarbon processing.^544^ In principle, there should also be an optimized spatial distribution of the two types of active sites in the integrated catalyst based on bimetallic nanoparticles and acid/basic sites. It can also be expected that the controllable preparation of such catalytic materials will be more challenging than the monometallic systems.

### 7.10 Bimetallic Nanoparticles with Chiral Structural Features

**Figure 54:**
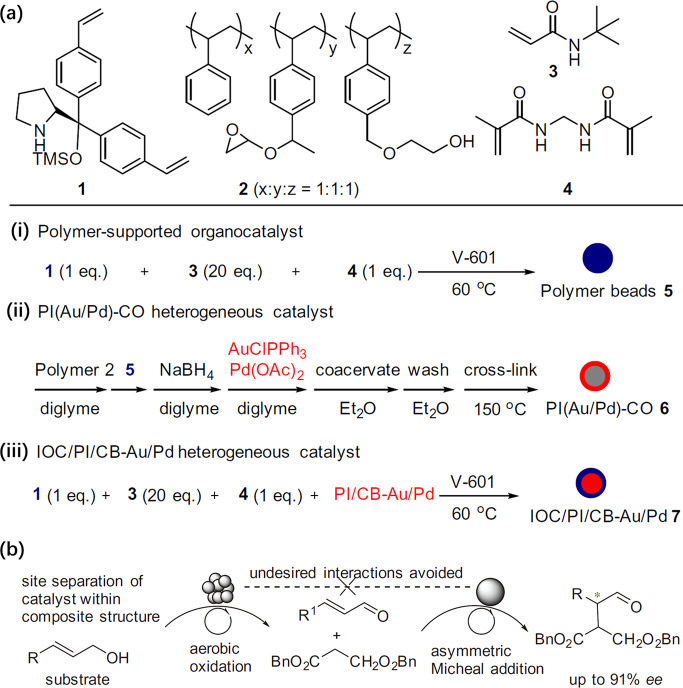
Bimetallic nanoparticles embedded in chiral polymers for enantioselective catalysis. (a) Synthesis of the polymer support with chiral microenvironment for accommodation of bimetallic AuPd nanoparticles. (b) Heterogeneous bifunctional catalysts for the cascade aerobic oxidation of alcohol and C–C bond formation through asymmetric Michael addition. Reproduced with permission from ref (545). Copyright 2013 Royal Society of Chemistry.

Carrying out enantioselective reactions with solid catalysts is one of most desirable goals of the catalysis community. Although great efforts have been dedicated to this topic and some success has been made with ligand-modified supported metal catalysts for enantioselective hydrogenation reactions, the reaction scope and performances are still far behind the well-established homogeneous systems.^546^ One plausible reason that limits the performance of ligand-modified metal nanoparticles could be the flexible conformation of the chiral template molecules absorbed on the surface, resulting in a less-ordered microenvironment.^547^ One strategy to improve the order degree of the chiral microenvironment, bimetallic nanoparticles are embedded in chiral polymers and have shown good performances for a variety of enantioselective C–C bond-forming reactions, such as asymmetric 1,4-addition of arylboronic acids with α,β-unsaturated carbonyl compounds^548^ and cascade oxidation of alcohol to conjugated aldehyde and Micheal addition reaction.^545^ The collaborative function of bimetallic nanoparticles and chiral polymer requires the proper control of the spatial distribution of the two functional components. As shown in Figure 54, the AuPd nanoparticles need to be located on the external surface of the polymer beads while the chiral organocatalysts should be located in the inner layer, as shown in panel (ii) in Figure 54a. If the spatial distribution is reversed (shown in panel (iii) in Figure 54a), the AuPd nanoparticles will be poisoned by the amine groups in the organocatalysts, resulting catalyst deactivation.

Using chiral molecule as template, bimetallic nanostructures with chiral features at nanometer scale can be generated via the chiral-imprinted route. For instance, mesoporous PtIr alloy structures with chiral cavities are synthesized by electrodeposition method using the combination of surfactant and chiral molecule as template.^549^ Good enantioselectivity has been achieved with the chiral PtIr alloy structure for electrocatalytic hydrogenation of acetophenone to phenylethanol. Furthermore, the chiral PtIr structure show much higher enantiomeric excess than the Pt structure, which could be associated with the higher structural stability of PtIr than Pt and therefore a more well-defined chiral structure at atomic scale.

Another practical approach for achieving enantioselective catalysis is to employ porous framework with ordered chiral microenvironment as host for bimetallic nanoparticles. This concept has been demonstrated with metal complexes encapsulated in chiral MOF structures but not shown with bimetallic nanoparticles.^550^ Considering the suitable reactions for bimetallic nanoparticles, encapsulation of bimetallic nanoparticles in chiral MOFs could be a class of promising recyclable catalysts for enantioselective hydrogenation and oxidation reactions.

Besides, the combination of bimetallic nanoclusters or nanoparticles with chiral zeolites can provide even better stability than the MOF structure, although it has not been achieved yet to date.^551,552^ Moreover, zeolite catalysts with specially modified structures can be prepared by imprinting the transition states of the target reactions by using the mimics as organic-structure directing agents.^553^ If the above two concepts are combined, it would lead to the formation of supported metal catalysts confined in well-defined chiral environment with customed surrounding environment for catalyzing enantioselective reactions.

### 7.11 High-Entropy Alloy Particles for Catalysis

**Figure 55:**
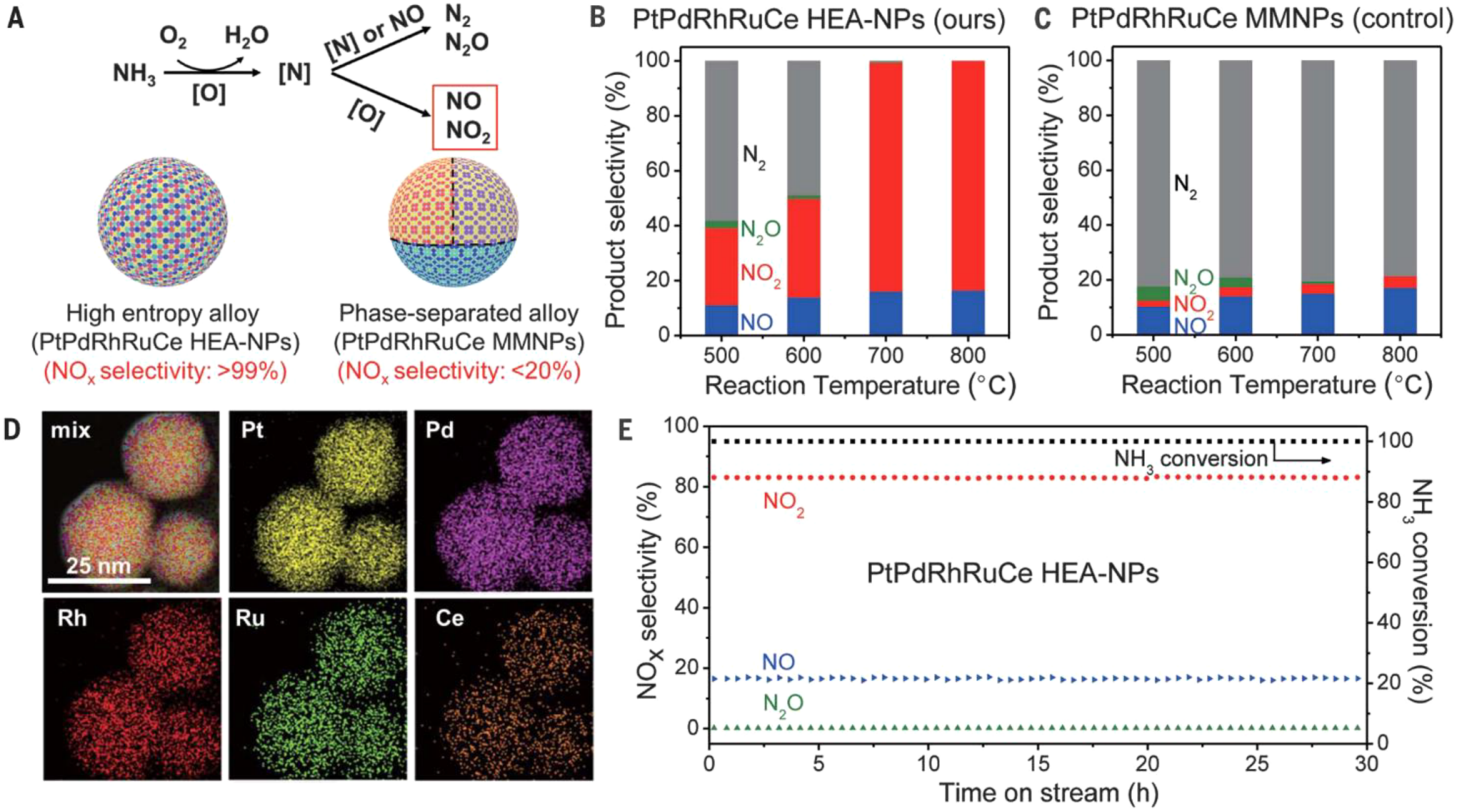
Catalytic performance of quinary high-entropy alloy particles (PtPdRhRuCe) for ammonia oxidation. (A) Reaction scheme for the ammonia oxidation process as well as the structural and performance differences between the PtPdRhRuCe high-entropy alloy particles synthesized by CTS and the control sample (phase-separated PtPdRhRuCe) by wet impregnation. (B,C) Temperature-dependent product distribution and conversion of NH_3_ for PtPdRhRuCe high-entropy alloy particles and phase-separated PtPdRhRuCe particles, respectively. (D) STEM elemental maps for PtPdRhRuCe HEA-NPs. (E) The stability test of PtPdRhRuCe high-entropy alloy particles at 700 °C. Reproduced with permission from ref (554). Copyright 2018 AAAS.

The above-mentioned works have demonstrated that various structural parameters can influence the catalytic properties of bimetallic nanoparticles. It can be anticipated that the complexity in terms of structure–reactivity relationship will be greatly elevated if more metal elements are introduced.^555^ Thanks to the developments in synthesis of high-entropy alloy nanoparticles, the exploration of the catalytic applications of these materials are emerging topics in catalysis community.^556,557^ The advantages of high-entropy alloy catalysts have been proved with various types of reactions, such as decomposition of ammonia,^558^ oxidation of ammonia to NOx,^554^ combustion of methane^559^, and electrocatalytic oxidation of methanol and ethanol.^560,561^ As shown in Figure 55, the high-entropy alloy catalyst shows much better selectivity to desired products for oxidation of ammonia to NOx than the conventional phase-separated multimetal nanoparticles prepared by conventional method. The PtPdRhRuCe nanoparticles remain stable for over 30 h at 700 °C under the conditions for ammonia oxidation reaction. In comparison to the well-established PtPdRh catalysts used in the industrial process, the addition of Ru and Ce can inhabit the segregation of the noble metals and alleviate the sintering.

**Figure 56:**
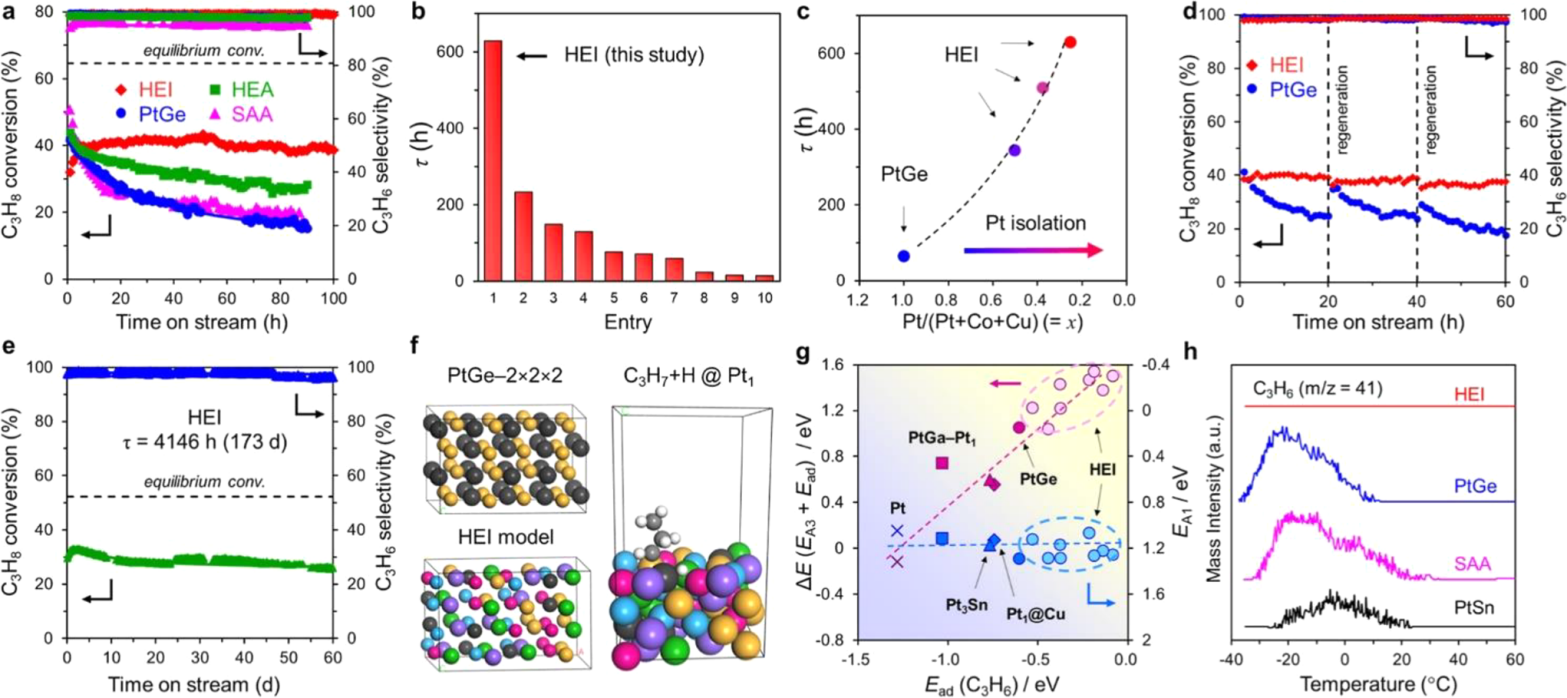
High-entropy intermetallic catalysts for propane dehydrogenation. (a) Catalytic performances of the PtGe, high-entropy intermetallic (HEI), single-atom alloy, and high-entropy alloy for PDH reaction at 600 °C without cofeeding H_2_. (b) Mean catalyst life (τ = *k*_d_^–1^) of reported catalysts and high-entropy intermetallic catalyst in PDH without co-fed H_2_. (c) Relationship between mean catalyst life (τ = *k*_d_^–1^) and the degree of Pt isolation represented by *x* as the Pt/(Pt + Co + Cu) molar ratio in the PtGe and HEI catalyst. (d) Reusability of PtGe and HEI catalyst in PDH at 600 °C after repeated regeneration processes. (e) Long-term stability test of the HEI in PDH at 600 °C with cofeeding H_2_ (catalyst: 150 mg, C_3_H_8_/H_2_/He = 2.5:1.3:3.7 mL min^–1^, WHSV = 2.0 h^–1^). (f) Model structure of the HEI (left bottom) derived from PtGe (left top) for DFT calculations. An example of the HEI slab model for PDH: C_3_H_7_ + H at a Pt_1_ site (right). (g) Relationship between *E*_ad_ (C_3_H_6_) and Δ*E* or *E*_A1_ for various Pt-based surfaces. (h) C_3_H_6_-TPD for Pt-based catalysts (adsorption temperature: −35 °C). Reproduced with permission from ref (570). Copyright 2022 American Chemical Society,.

The increasing number of publications on catalytic applications of high-entropy alloy nanoparticles calls for a unified understanding on the advantages of high-entropy alloy nanoparticles over the conventional monometallic and bimetallic catalysts. This goal could be achieved by translating the knowledge and experiences accumulated in previous studies with the bimetallic systems to the more complicated systems. It is well established that PtM bimetallic particles (M = Sn, Ga, In, Zn, etc.) can greatly prolong the lifetime of the Pt catalyst for dehydrogenation of light alkanes to alkenes by suppressing the coke deposition.^243^ The introduction of a third metal (such as Pb) into the bimetallic PtGa nanoparticles leads to further improvement in the catalyst’s lifetime because the Pb atoms can bond with the Pt atoms surface of PtGa alloy particles and then transform the Pt ensembles into isolated Pt sites, which are highly active and selective for transforming propane into propylene but not active for cleavage of the C–H bonds in propylene, thus suppressing the coke deposition.^562^ The catalyst’s lifetime can be further extended by employing high-entropy PtCoCuGeGaSn alloy nanoparticles supported on SiO_2_ as the catalysts, in which the Pt sites are claimed separated by the transition and main-group metals.^563^ As shown in Figure 56, the catalyst’s lifetime increases with the degree of Pt isolation represented by the loading of Pt in the high-entropy alloy particles. The DFT calculations and the temperature-programmed desorption experiment results suggest that the adsorption of propylene on the high-entropy alloy particles is almost completely suppressed, which contributes the exceptionally high stability.

Some theoretical calculation studies propose that the electronic features of high-entropy alloys are greatly different to the monometallic or bimetallic particles. However, the calculation results are not convincing enough to be correlated with the experimental results, because the realistic structures of high-entropy alloy particles are too complicated to be modeled. To have a reliable description on the electronic features of high-entropy alloy particles, a practical approach is to employ machine-learning strategies to accelerate the calculation process.^564^ This strategy usually employ the use of reactivity descriptor (such as the adsorption energy of the reactant or intermediate on the catalyst’s surface) as the indicator for fast screening and the choice of the descriptor relies on the fundamental understanding on the reaction mechanism of the target reaction.^565,566^ In this sense, the knowledge accumulated with bimetallic systems can be translated to the high-entropy alloys to facilitate the exploration of a large number of potential candidate materials.

In order to further explore the potentials of catalysts based on high-entropy alloys, the methodologies for materials characterizations developed for monometallic and bimetallic nanoparticles need to be adjusted or even to be reinvented. For instance, there are no well-established methods/techniques for measuring the number of exposed surface sites, the spatial distribution of the elements, and the atomic structures of the high-entropy alloys. Besides considering the theoretically infinite configurations of high-entropy alloy catalysts in terms of their composition, it will be necessary to employ high-throughput techniques to efficiently explore the synthesis, characterization, and catalytic applications of these materials in both theoretical and experimental approaches.^567,568^ Nevertheless, to achieve a unified understanding on the structure–reactivity relationships in high-entropy alloy catalysts, the structural features and catalytic performances should be extracted from the experimental results and classified properly in order to build a database for in-depth analysis.^569^

From a mechanistic point of view, there should be an optimum active site in the high-entropy alloys for the target catalytic reaction, although there are numerous types of sites in those particles. In this sense, the percentage of the useful metal atoms contributing to the formation of the real working active sites in the whole catalyst could be low. Therefore, employing advanced characterization techniques and performing rigorous kinetic studies are crucial to identify the active sites which make the major contribution, and thereafter the rational design of multimetallic catalysts would be possible.

### 7.12 Perspectives

In this section, we have witnessed the diversity of the structural features of bimetallic nanocatalysts and the structure–reactivity relationship proposed in the literature works have greatly enriched our knowledge and fundamental understanding on the catalytic properties of the emerging bimetallic nanostructures. For a specific reaction, various types of bimetallic nanostructures may show activity for the desired transformations and some inconsistence can be found with different literature works in terms of the nature of the active sites. These discrepancies hinder the formation of a unified understanding on the structure–reactivity relationship and rational design of bimetallic and multimetallic nanocatalysts. To improve the efficiency of research activity toward understanding the nature of the active sites, we will propose a general workflow for elucidating the plausible active sites in bimetallic catalysts in the next section of this review.

## 8 Perspectives

From the above discussions, it appears that heterogeneous bimetallic catalysts can offer many possibilities in the fields of catalysis, more specifically, in the field of electrocatalysis. In this section, we will present perspectives on the future developments of heterogeneous bimetallic catalysts and more broadly, the future directions of heterogeneous catalysis research.

### 8.1 Precise Synthesis of Bimetallic Sites

#### 8.1.1 Precise Synthesis of Subnanometer Bimetallic Sites

Because the geometric structures of the bimetallic sites have a profound influence on their catalytic performance, the precise control of the structural features determines the homogeneity of the active sites in the final catalyst. In principle, synthesis methodologies for binuclear bimetallic sites and bimetallic nanoclusters with well-defined structures have been established. However, these methods usually require the use of organic ligands for the stabilization of the tiny metal species, which would normally block the active sites. Grafting the binuclear sites or bimetallic nanoclusters on solid carriers and employing appropriate treatments may liberate the metal atoms from the blockage by the ligands. However, during the removal of the organic ligands, sintering of the binuclear sites or bimetallic nanoclusters becomes a critical issue that has not been properly solved yet in many systems. To mitigate the sintering issues, the choice of the solid carrier is critical for providing anchoring sites to limit the mobility of the bimetallic entities. Besides, synergistic effects between the bimetallic nanoclusters and the support may lead to novel catalytic properties than the unsupported nanoclusters.

Another approach for constructing uniform binuclear bimetallic sites and bimetallic nanoclusters on solid carriers is the use of crystalline microporous materials as the host for the tiny bimetallic entities, as mentioned before in this review. We would like to emphasize here that MOF structures will be excellent supports for accommodating subnanometer bimetallic entities because the nodes and linkers provide well-defined sites for the generation of the bimetallic sites through controllable chemical transformations. In the case of taking zeolites as the support, the structural rigidity of the zeolite framework allows for the generation of highly stable bimetallic sites, but it remains a great challenge to precisely control the coordination environment of the metal species in the zeolite structure. The coordination environment of metal species located inside the pores/cavities of zeolites is not as clearly identified as the situation with the metal catalysts supported on MOFs, especially for the noble metal species stabilized in zeolites. In particular, the anchoring sites of noble metal species and the mechanism for stabilization of subnanometer metal clusters inside the zeolite structure need to be elucidated.

#### 8.1.2 Precise Synthesis of Bimetallic Nanostructures

In the case of bimetallic nanostructures, the control of their geometric structures with atomic-level precision is challenging due to the presence of hundreds of atoms in a single bimetallic nanoparticle. From a practical point of view, the synthesis of bimetallic nanoparticles with high uniformity in terms of the chemical composition and the spatial distribution of the metal elements in the particles is crucial for approaching the “ideal” solid catalysts with only one type of active site. In a first attempt, this goal may be achieved by preparing single-atom alloy nanoparticles in which the isolated metal atoms are selectively located in specific sites in the matrix metal. In most of the reported single-atom alloy particles, the spatial distributions of the isolated metal atoms are not precisely controlled. It can be expected that the coordination environment and electronic features of the isolated metal atoms could be dependent on their location in the matrix. To address such impacts, one may use metal nanocrystals with regularly exposed facets as the matrix and then deposit isolated metal sites on the specific facet of the metal nanocrystals for the generation of single-atom alloy structures with well-defined geometric features.^100^

For many bimetallic nanoparticles, the active sites are associated with the metal ensembles. However, the optimum structural features of the metal ensembles are elusive to be elucidated because of the challenges in the preparation of a series of model catalysts with different sizes of metal ensembles. Besides the size of the metal ensembles, the surrounding environment of the metal ensembles, whose impacts on the catalytic performances have barely been studied, can be a determinant. The clarification of the above-mentioned structure–reactivity relationships calls for the improvement in the precision of the synthesis methodologies for the generation of bimetallic catalysts with uniform and well-defined structures.

### 8.2 Quantitative Measurements of the Bimetallic Entities

Currently, state-of-the-art characterization techniques are able to directly measure the particle sizes of the bimetallic entities on solid carriers by advanced electron microscopy techniques and then provide information on the coordination environment of the bimetallic species by spectroscopy tools. However, the information from microscopy techniques is highly local, while the spectroscopy characterization results usually give an overall estimation of the whole sample. Taking into account that in the vast majority of supported bimetallic catalysts, several types of bimetallic entities are simultaneously present, a reliable structure–reactivity relationship needs to be established based on the quantification of the number of each type of bimetallic entity. However, to the best of our knowledge, there is no general characterization technique for achieving that goal, which requires a combination of electron microscopy and spectroscopy techniques.^571,572^ This strategy has been shown with the combination of in situ TEM and in situ X-ray absorption spectroscopy for characterizing the evolution of supported Pt catalyst, which allows giving a more reliable description of the contribution of different types of Pt species in the whole catalyst.^573^

The determination and quantitative measurements of the active sites will be critical for revealing the nature of the active sites and modeling them, in high-entropy alloy nanoparticles, which are emerging catalytic materials in recent years. In principle, there should be an optimized active site for the target reaction in the high-entropy alloy nanoparticles. However, due to the structural complexity, the number of the optimized sites should be low because the exposed metal sites are randomly distributed, showing highly disordered structures. In this sense, there should be plenty of room for improving the catalytic performances of high-entropy alloy nanoparticles if the catalytically relevant sites can be identified, modeled, and then generated in a rational way.

### 8.3 Synergy between Different Types of Metal Entities

Fundamental understandings of the nature of the active sites in supported metal catalysts will reveal the advantages and limitations of each type of metal entity. For a given reaction, the catalytic cycle usually invokes several critical elementary steps, which may require different types of active sites, as demonstrated with the broad applications of bifunctional catalysts made by metallic and acid/basic sites and in the case of supported metal catalysts based on metal entities with different particle sizes.

**Figure 57:**
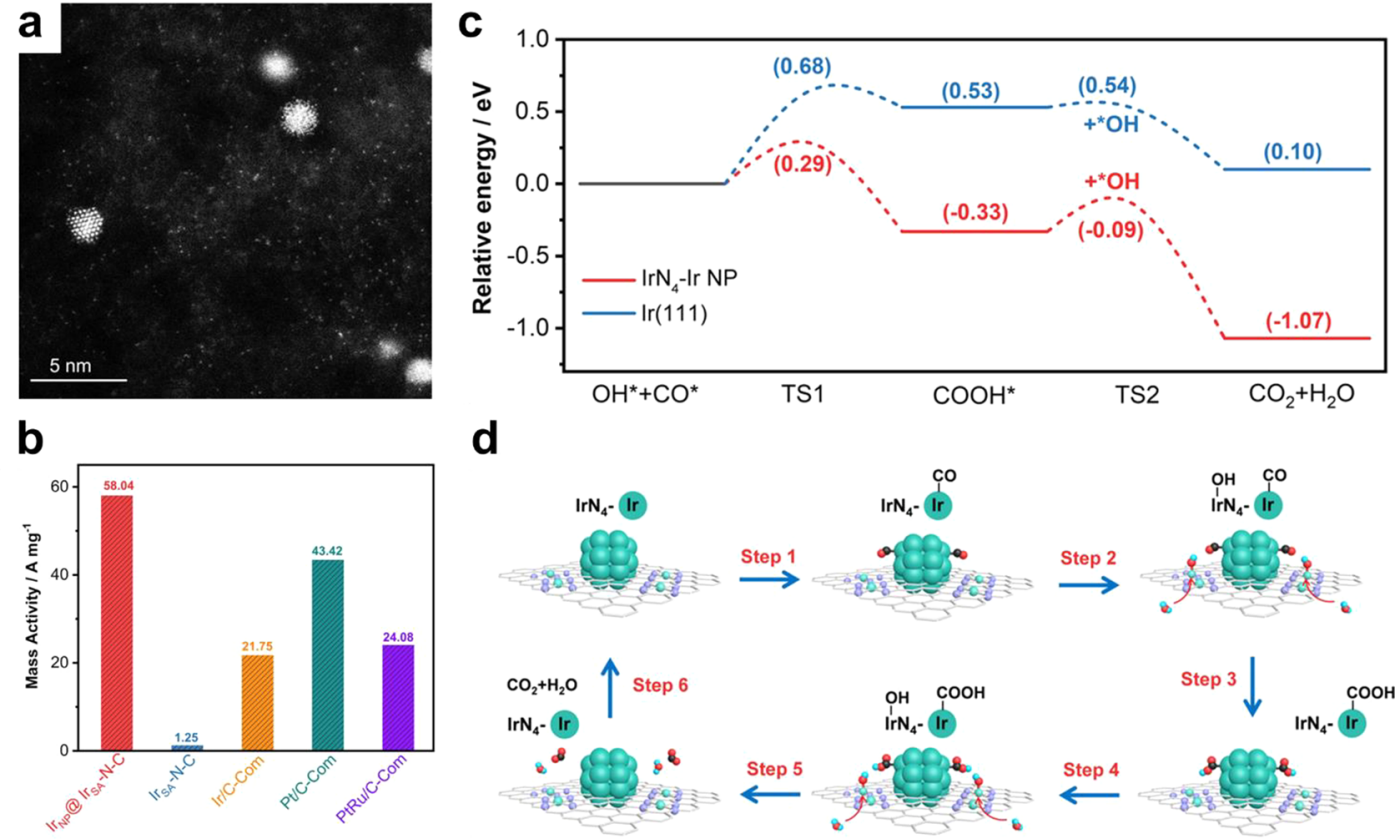
Combination of Ir nanoparticles and isolated Ir atoms for the electrocatalytic H_2_ oxidation reaction. (a) High-resolution HAADF-STEM image of Ir_NP_@Ir_SA_-N-C, confirming the coexistence of single Ir atoms and Ir nanoparticles. (b) The metal mass activities of different catalysts at 0.6 V in H_2_-O_2_ proton-exchange membrane fuel cells. (c) The free energy profiles of the removal of CO on IrN_4_-Ir_NP_ and Ir(111). (d) Schematic illustration of the synergistic effect mechanism between IrN_4_ sites and Ir nanoparticles. Ir, N, C, O, and H atoms are shown in light green, light purple, black, red, and light blue, respectively. Reproduced with permission from ref (574). Copyright 2021 Wiley-VCH.

The combination of different metal entities as active sites has been demonstrated to be an effective strategy for promoting the overall performances of some reactions. For instance, to mitigate the deactivation of the anode catalyst in proton-exchange membrane fuel cells by CO, an Ir/C catalyst comprising the mixture of Ir nanoparticles and isolated Ir atoms has been prepared, in which the isolated Ir atoms can facilitate the oxidation of CO adsorbed on Ir nanoparticles. As shown in Figure 57, the poisoning of Ir nanoparticles by CO is greatly suppressed and the superior performance of Ir nanoparticles for H_2_ oxidation reaction can be fully exhibited.^574^ According to theoretical calculations, active *OH species are formed due to the cleavage of the H–O bond in H_2_O at the isolated Ir atoms embedded in the N-doped carbon matrix, which can further react with the CO adsorbed on Ir nanoparticles to oxidize the CO into CO_2_. In this regard, the isolated Ir atoms should be located at the adjacent sites of the Ir nanoparticles in order to achieve the synergy between the two types of Ir entities. In another example, the combination of isolated Fe atoms and Fe clusters supported on N-doped carbon can boost the performance of isolated Fe atoms by placing Fe clusters at the neighboring sites, because the electronic features of the FeN_4_ sites are influenced by the Fe clusters, resulting in a lower energy barrier for the formation of *OH species on the FeN_4_ sites.^575^

The advantages of combining metal entities of different sizes have been shown in thermal catalytic reactions, such as the combination of isolated Rh atoms and Rh nanoparticles for dehydrogenation of cyclohexanol to phenol^576^ and the combination of isolated Pd atoms and Pd nanoparticles for hydrogenation of ketone and aldehyde compounds.^577^ In the case of Rh catalyst, the spatial intimacy of the isolated Rh atoms, and Rh nanoparticles seem not as critical as the situation in the above-mentioned supported Ir catalyst because the intermediates can transport from one site to another site for completing the catalytic transformation. In the case of Pd catalyst, the role of Pd nanoparticles is claimed to be associated with the dissociation of H_2_, and the activated H species will be transferred to the isolated Pd atoms, where the carbonyl groups are adsorbed, for performing the hydrogenation of the ketone and aldehyde compounds. Therefore, the isolated Pd atoms should be located at the neighboring sites of Pd nanoparticles in order to maximize the efficiency of the H transfer process. In an example of Rh/CeO_2_ system comprising isolated Rh atoms and Rh clusters supported on CeO_2_, the production of ethanol from syngas is ascribed to the synergy of Rh atoms and Rh clusters for CO hydrogenation and C–C coupling reaction, respectively.^578^

**Figure 58:**
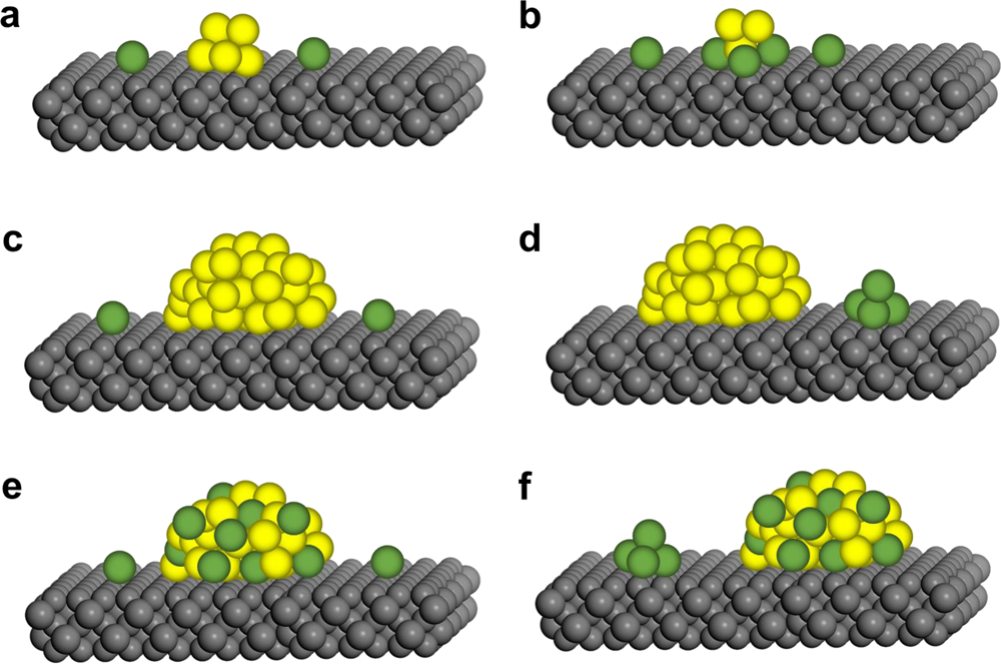
Schematic illustrations of supported bimetallic catalysts based on the combination of metal entities with different sizes. (a) Combination of isolated metal atoms and metal clusters, (b) combination of metal atoms and bimetallic clusters, (c) combination of metal nanoparticles and metal atoms, (d) combination of metal nanoparticles and metal clusters, (e) combination of bimetallic nanoparticles and metal atoms, and (f) combination of metal clusters and bimetallic nanoparticles.

The above-mentioned examples are based on the combination of monometallic clusters/nanoparticles and isolated metal atoms. In principle, the synergistic effects of atomically dispersed metal species and agglomerated metal entities (clusters and nanoparticles) should also work for bimetallic systems. For instance, as illustrated in Figure 58, a mixture of bimetallic clusters/nanoparticles and isolated metal atoms (one of the metal elements in the bimetallic clusters/nanoparticles) can be constructed by loading the bimetallic entities onto a solid carrier comprising isolated metal atoms. This concept has already been demonstrated with the PtFe nanoparticles on metal–N-C support, in which the isolated metal atoms in the support can greatly promote the durability of PtFe and PtCo alloy nanoparticles by suppressing the leaching and sintering.^579,580^

### 8.4 Unified Understanding of the Active Sites

Although the size of bimetallic entities can vary greatly from the subnanometer regime (such as binuclear sites and tiny bimetallic clusters) to the nanometric regime, the active site that is directly involved in the catalytic cycle should be a highly local area. For the vast majority of the reactions catalyzed by heterogeneous metal catalysts, the substrate molecules should interact with a few atoms (probably less than 10 atoms) at the active site, including the atoms from the metal entities and from the support. In this sense, for a specific reaction, the active site in supported bimetallic catalysts comprising different bimetallic entities may share a common structural feature. However, for a given reaction, there are always arguments in the reported works in terms of the nature of the active sites, which precludes the formation of a unified description on the active sites. Some of the different conclusions may result from the discrepancies in the experimental conditions, and some of the contradictive viewpoints may originate from the mistakes in characterizing the “real” active sites.

In the past decade, we have witnessed a very rapid growth of the number of publications in all the fields of catalysis research (spanning from homogeneous to heterogeneous and enzymatic catalysis). On one hand, a large number of new publications will certainly enrich our knowledge in catalysis and provide many information/insights on new synthesis methodologies for catalyst preparation, new techniques for catalyst characterizations, and interesting catalytic properties of new materials. On the other hand, the appearance of publications without strict quality control may cause confusion or even mislead to other researchers, which hinders the formation of a reliable and unified understanding of the active sites in solid catalysts for a target reaction. Unified understandings on the nature of the active sites rely on rigorous design and careful execution of the experiments and adequate data analysis.^581^ For instance, in order to clarify the optimum size of metal entities for a specific reaction, it is recommended to prepare a series of supported metal catalysts by a same method but modulate the particle size distributions by tuning a few synthesis parameters (the less the better). Otherwise, some unknown factors such as impurities in the final solid catalysts could affect the reliability of the comparative studies.

As emphasized along the whole review, the dynamic structural transformations of supported metal catalysts are ubiquitous and the overlooked evolution of the metal entities under reaction conditions may lead to mistakes in identification of the active sites. Such have been frequently encountered with supported monometallic catalysts, and it can be anticipated that such mistakes will more often occur with supported bimetallic catalysts due to the higher degree of structural complexity than the monometallic system. In this regard, it becomes very important to follow the structural features of bimetallic catalysts (morphology, particle size, spatial distribution of the metal elements, etc.) under reaction conditions, or at least simulated reaction conditions. It should be noted that the data derived from the characterizations will provide multiple potential parameters/descriptors for establishing the structure–reactivity correlation, which also relies on distinguishing the critical parameters/descriptors from the spectators.

Correlating the knowledge and lessons generated in different fields of catalysis is helpful for achieving unified understandings on the active sites. For instance, dehydrogenation of alkanes into alkenes can be catalyzed by homogeneous and heterogeneous catalysts.^582,583^ The homogeneous catalysts rely on the modification of the coordination environment (usually through the modification of the ligand/solvent) of the metal centers, while the heterogeneous catalysts rely on the tuning the size and composition of the metal entities, as well as the near and the next nearest neighbors. It is then of interest to compare the working mechanism of homogeneous and heterogeneous systems and then figure out the similarities and dissimilarities for the same type of reaction. This approach will be especially helpful for acquiring new insights when combining the available theoretical calculations and characterization techniques.

### 8.5 New Research Paradigm for Developing New Solid Catalysts

Since the establishment of the fundamental concepts in catalysis in the 19th century, heterogeneous catalysis has evolved into an interdisciplinary field, in which scientists with different backgrounds spanning from chemistry to materials science and engineering are attracted to tackle the challenges in fundamental or practical perspectives.^584^ During the 20th century, the tremendous progress in heterogeneous has made great impacts on the chemical industry, which shapes our current energy landscape and human society. The vast majority of the concepts, tools, and methodologies of heterogeneous catalysis are established on the research activities in dealing with substances from fossil energy resources. Nevertheless, catalysis and the chemistry principles are the same regardless of the origin of the reactants. Therefore, catalysis will continue to serve as an indispensable role in converting renewable energy into chemicals and materials for a sustainable world. For instance, delocalized production of chemicals via electrochemical processes are considered as a promising approach to utilize the renewable electricity produced from solar and wind energy, although there are many technical problems remaining to be solved. Taking into account of the advantages of thermal catalytic processes in scaling up, the coupling of the electrochemical and thermal processes for converting feedstocks (such as CO_2_, N_2_, H_2_O, biomass, plastic waste, etc.) into value-added products may improve the overall efficiency and facilitate the realization of these systems. Developing coupled catalytic systems require the combination of knowledge from different disciplines. For instance, one needs to learn the principles of thermal catalysis and electrochemistry in order to develop a hybrid catalytic system based on the coupling of thermal and electrochemical processes.^585−589^ For instance, the use of high-temperature solid oxide fuel cells and membrane reactor heavily depend on the integration of design of electrochemical devices and application of solid catalysts.^590,591^

The coupling of different expertise for developing novel catalytic systems can also be achieved by introduction of other external excitations into the conventional thermal reaction systems. For instance, the combination of light irradiation and heating gives to the realization of photothermal catalytic system, and the introduction of light irradiation on the solid catalysts can lower the energy barrier of the activation of the reactants and modulate the intermediates formed on the catalyst’s surface, leading to superior performances or distinct product distributions compared to the conventional thermal systems.^322,592,593^ However, the contribution of light irradiation and the mechanism of the synergy of light irradiation and thermal heating in the catalytic cycles are not clearly elucidated.^594^ The situation is similar with other hybrid catalytic systems assisted by external fields, such as the electric-field-assisted reactions and magnetic-field-assisted reactions, which requires further studies to reveal the fundamental mechanisms, especially the reaction pathways in the hybrid catalytic systems.^595−597^

Owing to the advances in the synthetic modification of enzymes, the synthesis of enzyme mimicking molecules, and genetic modification of cells to produce new enzymes, will open more opportunities for manufacturing chemicals from biomass. No doubt that enzymatic and chemo-enzymatic processes will add more potential to the catalysis field, and the combination of enzymes and solid catalysts to form hybrid systems will enable novel catalytic transformations which are not feasible via conventional routes.^598−601^ As always, we expect major advances within the interphase of the different confluent fields and this progress will bring new insights and inspirations to future developments of catalysis science and technology.

Finally, the rapid developments of software and hardware tools based on artificial intelligence and machine learning are also bring new opportunities to catalysis research. The information extraction based on natural language processing offer the possibility to summarize the useful information from the literature and further analysis based on machine learning will guide the design of experiments for materials synthesis.^602^ This strategy has already been demonstrated with the development of solid catalysts for selective oxidation reactions and the synthesis of zeolite catalysts with novel organic structure-directing agents.^603−605^ Moreover, the automated high-throughput techniques can greatly promote the screening of the catalytic materials for a target reaction, and the data analysis based on artificial intelligence can dramatically decrease the time of catalyst optimization.^606−608^ The combination of machine learning and theoretical calculations will greatly promote the efficiency of modeling the atomic structures of complex solid catalysts (such as zeolites and bimetallic nanoclusters/nanoparticles), which provide new insights on the elementary steps (diffusion to the catalyst’s surface, adsorption, surface reaction, and desorption) on solid catalysts.^609,610^
